# Transcriptome-Based Analysis of Kidney Gene Expression Changes Associated with Diabetes in OVE26 Mice, in the Presence and Absence of Losartan Treatment

**DOI:** 10.1371/journal.pone.0096987

**Published:** 2014-05-14

**Authors:** Radko Komers, Bei Xu, Yi Fu, Aaron McClelland, Phillip Kantharidis, Amit Mittal, Herbert T. Cohen, David M. Cohen

**Affiliations:** 1 Division of Nephrology and Hypertension, Department of Medicine, Oregon Health & Science University, Portland, Oregon; 2 Portland V. A. Medical Center, Portland, Oregon, United States of America; 3 Nephrology Section, Boston University School of Medicine, Boston, Massachusetts, United States of America; 4 JDRF Danielle Alberti Memorial Centre for Diabetes Complications, Diabetes Division, Baker IDI Heart and Diabetes Institute, Melbourne, Australia; Universtiy of Maryland Schoool of Medicine, United States of America

## Abstract

Diabetes is among the most common causes of end-stage renal disease, although its pathophysiology is incompletely understood. We performed next-generation sequencing-based transcriptome analysis of renal gene expression changes in the OVE26 murine model of diabetes (age 15 weeks), relative to non-diabetic control, in the presence and absence of short-term (seven-day) treatment with the angiotensin receptor blocker, losartan (n = 3–6 biological replicates per condition). We detected 1438 statistically significant changes in gene expression across conditions. Of the 638 genes dysregulated in diabetes relative to the non-diabetic state, >70% were downregulation events. Unbiased functional annotation of genes up- and down-regulated by diabetes strongly associated (*p*<1×10^−8^) with terms for oxidative stress and for endoplasmic reticulum stress/protein folding. Most of the individual gene products up- or down-regulated with diabetes were unaffected by losartan treatment; however, of the gene products dysregulated in diabetes and influenced by losartan treatment, the vast majority of changes were in the direction of amelioration rather than exacerbation of the diabetic dysregulation. This group of losartan-protected genes associated strongly with annotation terms for endoplasmic reticulum stress, heat shock proteins, and chaperone function, but not oxidative stress; therefore, the losartan-unaffected genes suggest avenues for additional therapeutic opportunity in diabetes. Interestingly, the gene product most highly upregulated by diabetes (>52-fold), encoded by the cationic amino acid transporter *Slc7a12*, and the gene product most highly downregulated by diabetes (>99%) – encoded by the “pseudogene” *Gm6300* – are adjacent in the murine genome, are members of the SLC7 gene family, and are likely paralogous. Therefore, diabetes activates a near-total genetic switch between these two paralogs. Other individual-level changes in gene expression are potentially relevant to diabetic pathophysiology, and novel pathways are suggested. Genes unaffected by diabetes alone but exhibiting increased renal expression with losartan produced a signature consistent with malignant potential.

## Introduction

Diabetic nephropathy is one of the most serious microvascular complications of diabetes mellitus. Diabetic nephropathy is the leading cause of renal failure in industrialized countries, necessitating renal replacement therapy in affected individuals at enormous socioeconomic cost [1], [2]. Metabolic derangements and genetic factors conspire in susceptible diabetic patients to initiate and perpetuate nephropathy. Kidney cells exposed to the diabetic dysmetabolic milieu respond with altered gene expression, and with characteristic functional and structural changes. Renal hemodynamic changes, enhanced cell growth, and extracellular/mesangial matrix (ECM) production contribute to structural changes in glomeruli, tubules, and interstitium that lead to the development of proteinuria, decline in kidney function, and ultimately renal failure [1], [3].

Several studies have used global expression-based approaches to address genome-wide changes in RNA abundance, and to define novel insights into – and novel avenues for investigation in – the pathophysiology of diabetic renal disease [4]–[7]. Previous efforts have focused on chip-based strategies, the advantages of which have included economy and standardization of reagents (e.g., commercially manufactured chips). Transcriptome-based approaches utilize “next-generation” (i.e., high-throughput) sequencing of RNA transcripts after conversion to cDNA. An advantage of this approach is the lack of reliance upon previously identified genes and transcripts; therefore, it can complement hybridization-based strategies by being more comprehensive and less biased. For any given transcript mapping to a gene, expression level is standardized to the length of the transcript and the total number of mapped reads in the sample; this facilitates analytic consolidation of technical and biological replicates, and enables the direct comparison of expression across multiple experimental conditions with nucleotide-level precision.

In this study we applied the RNA-Seq method to identify transcriptome-wide changes in renal gene expression at an early stage of nephropathy in diabetic OVE26 mice, relative to nondiabetic FVB (background) controls. As an established murine model of type 1 diabetes, OVE26 mice develop morphologic and structural changes characteristic of human diabetic nephropathy [8]. We also assessed the impact of short-term angiotensin AT1 receptor blockade (losartan treatment) on the renal transcriptome in diabetic mice.

## Methods

### OVE26 Murine Model of Diabetes

Studies were conducted in the previously characterized OVE26 line, a murine model of Type 1 diabetes (T1D) [8]. OVE26 mice express a chicken calmodulin minigene under control of the rat insulin II promoter; they develop hyperglycemia within 24 h of birth secondary to decreased pancreatic insulin secretion [9]. OVE26 mice exhibit severe albuminuria and, at later stages, renal structural changes resembling human diabetic nephropathy [8], [10]. Male OVE26 mice on the FVB background and control FVB mice were obtained from the Jackson Laboratories. At age 15 weeks, diabetic mice were randomized to receive either angiotensin receptor blocker (ARB) losartan (20 mg/kg in drinking water for 7 days) or vehicle alone. After completion of the treatment period, diabetic mice and age-matched FVB non-diabetic control mice were placed in metabolic cages for urine collection for determination of urinary albumin (Albuwell, Exocell, Philadelphia, PA) and creatinine (Biovision, Milpitas, CA). The following day the mice were anesthetized with i.p. injection of Inactin (100 mg/kg body weight), and blood samples were obtained from the abdominal aorta (for determinations of HBA1c as marker of long-term glycemic control). At that time, the kidneys were removed, decapsulated, snap-frozen in liquid nitrogen, and stored at −80°C. A sagittal section of the right kidney was immersed in formalin for processing for histological evaluation on PAS-stained sections. These procedures were approved by the Institutional Animal Care and Use (Sub)Committee of the Research and Development Committee of the Portland VA Medical Center.

### RNA Preparation and Next-generation Sequencing

Total cellular RNA was isolated from mouse kidney using TriZol reagent (Invitrogen) in accordance with the manufacturer’s directions, and submitted to Otogenetics Corporation (Norcross, GA USA) for RNA-Seq assays. Briefly, the integrity and purity of total RNA were assessed using Agilent Bioanalyzer and OD260/280. 1–2 µg of cDNA was generated using Clontech SmartPCR cDNA kit (Clontech Laboratories, Inc., Mountain View, CA USA; Catalog# 634925) from 100 ng of total RNA, and adaptors were removed by digestion with Rsa*I*. This method uses low cycle number PCR to preferentially amplify poly(A^+^) RNA via a modified oligo(dT) primer. Resultant cDNA was fragmented via sonication (Covaris, Inc., Woburn, MA USA), profiled via Agilent Bioanalyzer, and subjected to Illumina library preparation using NEBNext reagents (New England Biolabs, Ipswich, MA USA; Catalog# E6040). The quality, quantity, and size distribution of the Illumina libraries were determined using an Agilent Bioanalyzer 2100. The libraries were then submitted for Illumina HiSeq2000 sequencing as per the manufacturer’s recommendations. Paired-end 90 or 100 nucleotide (nt) reads were generated, checked for data quality using FASTQC (Babraham Institute, Cambridge, UK), and subjected to data analysis using the DNAnexus platform (DNAnexus, Inc, Mountain View, CA USA) or the platform provided by Center for Biotechnology and Computational Biology (University of Maryland, College Park, MD USA) as previously described [11]. Transcript-level quantitation was in accordance with the DNAnexus White Paper (version 1.1, April 19, 2010) on RNA-Seq/3SEQ Transcriptome Based Quantification. Statistical analysis at the transcript level was performed using Cufflinks 2.0.2. Cufflinks (http://cufflinks.cbcb.umd.edu/) assembles transcripts, estimates their abundances, and tests for differential expression and regulation in RNA-Seq samples (Laboratory for Mathematical and Computational Biology, UC Berkley; Institute of Genetic Medicine, Johns Hopkins University; and the California Institute of Technology). Data were visualized in genomic context within the web-based DNAnexus viewer. All significant changes at the level of CDS are shown in **Table S26** in **File S2**. For all analyses, the terms up- and down-regulation are used to indicate increased or decreased expression (respectively), relative to another experimental group; these terms are not meant to convey an assessment that such changes are adaptive or maladaptive.

Samples designations were Ot3449–Ot3460. Group 1 (non-diabetic control) is samples Ot3449–Ot3454 (CK-1 through CK-6); Group 2 (DM+LOS) is samples Ot3455–Ot3457 (CK-7 through CK-9); and Group 3 (DM alone) is samples Ot3458–Ot3460 (CK-10 through CK-12).

### Real-time PCR

Total RNA (5 µg) was used to generate cDNA with the SuperScript III First-Strand Synthesis System (Invitrogen); product (3 µl) was amplified with TaqMan Universal PCR Master Mix (Applied Biosystems) on a StepOne Plus platform (Applied Biosystems). Comparisons were made using the ΔΔCt method [12] where a VIC-based probe set directed against 18S rRNA and run in parallel served as an internal control. Assays were not multiplexed; apart from internal controls, all probe sets were FAM-based. For one of the six non-diabetic samples, there was no amplification product for any of the probe sets; these data were excluded. Although C_t_ can not be directly compared across multiple probe sets (in contrast to data utilizing a single probe set), the average C_t_ for the high-expressing condition (non-diabetic or DM) for each probe set is shown below in brackets after the probe designation. Probe sets (Invitrogen) were as follows: **Gm6300_1**, Mm03949676_m1 [23.30]; **Gm6300_2**, Mm03949677_m1 [23.44]; **Slc7a12_1**, Mm00499866_m1 [21.87]; **Slc7a12_2**, Mm01283157_m1 [17.91]. Exons and introns spanned by each probe set are shown in the relevant figure (see Results). Data were expressed as mean +/− SEM for n = 3–5 biological replicates (samples), with 2 technical replicates per sample (i.e., individual samples assayed in duplicate).

### Immunoblotting

Kidney tissue was homogenized in lysis buffer with protease inhibitors, and separated via PAGE, transferred to PVDF membrane, immunoblotted, and analyzed densitometrically as previously described [13]. Primary antibody was directed against mouse renin (R&D Systems, Inc., Minneapolis, MN; cat. #AF4277; 1∶1000). To confirm equal lane loading, membranes were stripped and re-analyzed for actin expression. Only two of six FVB control samples were run owing to constraints of the gel comb in mini-format (maximum eight samples); this approach was used so that all lanes could appear on the same exposure of the same film. The two FVB control samples were selected at random. Additional primary antibodies were obtained from Santa Cruz Biotechnology. Blots were subjected to densitometric analysis and normalized to actin control.

### Functional Annotation of Gene-level Data

Functional annotation (i.e., pathways-based analysis) was performed using the DAVID Bioinformatics Resources 6.7 (National Institute of Allergy and Infectious Diseases, NIAID, NIH; http://david.abcc.ncifcrf.gov/home.jsp [14]. Gene symbols were uploaded to the web interface and background was set to the entire Mus musculus genome. Default annotation dictionaries were used (definitions of abbreviations follow); these included annotation terms for disease states (OMIM_disease); functional categories (COG ONTOLOGY, SP_PIR keywords, and UP_SEQ features); gene ontology (GOTERM_BP_FAT, GOTERM_MF_FAT, and GOTERM_CC_FAT); pathways (BBID, BIOCARTA, and KEGG_PATHWAY); and protein domains (SMART, INTERPRO, and PIR superfamily). Where indicated in Results, tissue expression (UP_TISSUE) was also considered. For these databases, definitions and URLs are as follows: GO terms, The Gene Ontology (http://www.geneontology.org/GO.indices.shtml); COG, Clusters of Orthologous Groups of proteins, Phylogenetic classification of proteins encoded in complete genomes (http://www.ncbi.nlm.nih.gov/COG/index.html); OMIM, Online Mendelian Inheritance in Man, An Online Catalog of Human Genes and Genetic Disorders (http://www.omim.org/); SP-PIR, keywords derived from the SwissProt and Protein Information Resource datasets (e.g., http://www.uniprot.org/docs/keywlist); BBID, List of Keywords - Biological Biochemical Image Database (bbid.irp.nia.nih.gov/bbidkeyword.html); BioCarta, pathways annotations (http://www.biocarta.com/); KEGG: Kyoto Encyclopedia of Genes and Genomes - GenomeNet (http://www.genome.jp/kegg/pathway.html); SMART, Simple Modular Architecture Research Tool (http://smart.embl-heidelberg.de/); INTERPRO, The Integrated Resource of Protein Families, Domains and Sites (http://mips.helmholtz-muenchen.de/genre/proj/FGDB/Search/Catalogs/searchCatfirstIpr.html); PIR superfamily, the Protein information Resource’s non-overlapping clustering of UniProtKB sequences into a hierarchical order to reflect their evolutionary relationships (http://pir.georgetown.edu/pirsf/); and UP_TISSUE, the Universal Protein Resource (UniProt) tissue annotation (http://www.uniprot.org/manual/tissue_specificity).

### Gene Expression Analysis using the Cancer Genome Atlas (TCGA) Database

To compare gene expression between ccRCCs and their normal counterpart, the ccRCC patient database available at TCGA was downloaded (https://tcga-data.nci.nih.gov/tcga/) for further analysis. This database includes gene expression profiling (GEP) of ccRCCs and corresponding normal tissues from 66 patients. GEP was performed using the Illumina HiSeq RNA platform. These data were further collated and processed using Significance Analysis of Microarrays of BRB (Biometric Research Branch) Array tools (http://linus.nci.nih.gov/BRB-ArrayTools.html) at the significance of p<0.001 and FDR<0.001. The individual significance of selected genes was also determined using the Student’s t-Test (p<0.05).

## Results

### Metabolic Parameters and Renal Histological Appearance

**Figure 1:**
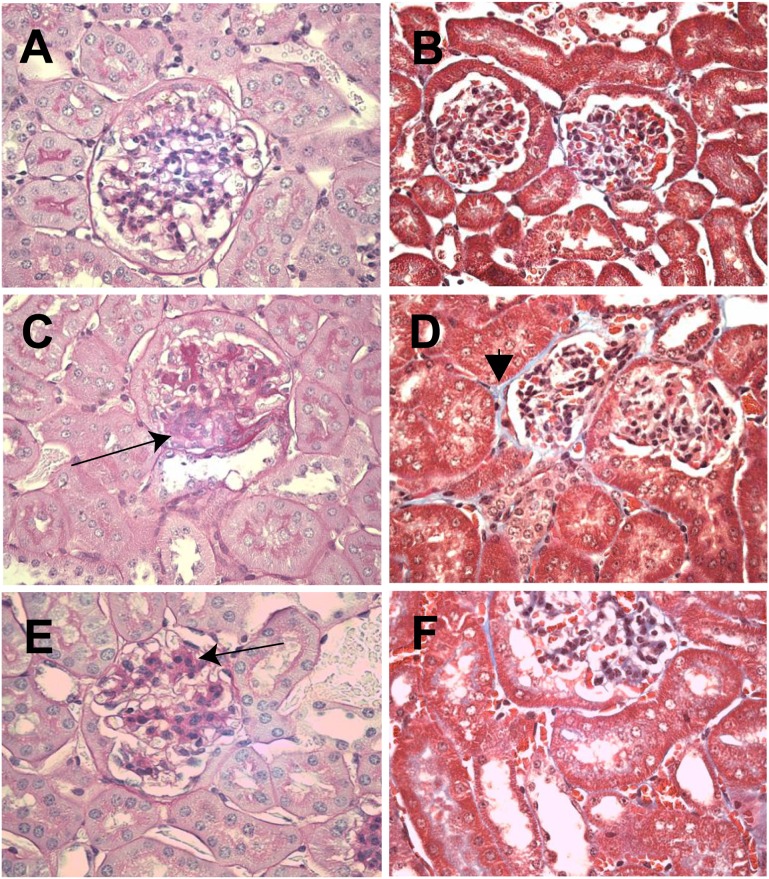
Representative renal histological findings from control FVB mice and from untreated and treated diabetic OVE26 mice. Shown are PAS- (**A, C, E**) and trichrome- (**B, D, F**) stained sections from control FVB mice (**A, B**) from vehicle-treated diabetic OVE26 mice (**C, D**), and from losartan-treated OVE26 mice (**E, F**). In contrast to non-diabetic mice which did nor show any renal pathology, diabetic mice displayed diffuse mesangial expansion (**C, E; arrow**). Tubulointerstitial fibrosis was not detectable at this stage of DM; however, trichrome blue stain was detected in the interstitial space in OVE26 mice (e.g., **D, arrowhead**), suggesting early accumulation of extracellular matrix material.

The OVE26 mice (both vehicle- and losartan-treated) weighed less (**Table 1**), but exhibited a kidney weight to body weight ratio that was ∼60% greater than non-diabetic control mice. Glycated hemoglobin (HbA1c) was elevated in treated and untreated OVE26 groups. Albuminuria (measured as urinary albumin to creatinine ratio) was markedly elevated in the vehicle-treated OVE26 mice (1240+/−690 mg/mmol); it was substantially decreased with losartan treatment (158+/−33 mg/mmol), although not fully to non-diabetic levels (60+/−20 mg/mmol). Renal histology was examined in non-diabetic FVB mice and diabetic OVE26 mice (**Figure 1**). In contrast to non-diabetic (non-diabetic) mice, which did not show any renal pathology, diabetic mice displayed diffuse mesangial expansion by PAS staining (**Figure 1C**). Tubulointerstitial fibrosis was not detectable at this stage of diabetes; however, trichrome blue staining was evident in the interstitial space in OVE26 mice (**Figure 1D**), consistent with early accumulation of extracellular matrix. Losartan treatment for seven days did not influence the diabetic renal histology (**Figure 1E,F**).

**Table 1:** Physical and metabolic characteristics in OVE26 and non-diabetic FVB mice.

Group	BWT [g]	LKW [g]	KW/BWT ratio [%]	HBA1c [%]	ACR [mg/mmol]
**FVB**	29±2	0.25±0.01	0.86±0.05	3.1±0.2	60±20
**OVE26-V**	20±1†	0.28±0.02	1.39±0.08†	6.1±0.3†	1236±685†
**OVE26-L**	22±1†	0.31±0.02#	1.40±0.04†	5.6±0.1†	158±33* #

*p<0.05,

† p<0.01 vs. FVB;

# p<0.05 vs. OVE26-V.

### Data Format and Validation

Individual transcript-level data from each RNA-Seq experiment were aligned with the mm9 murine reference genome. Within each sample, gene expression level was quantitated as FPKM (fragments per kilobase of transcript per million mapped reads), permitting normalization across samples within an experimental condition. Statistical comparison was then performed between groups at the level of the individual transcript using Cufflinks (http://cufflinks.cbcb.umd.edu/). Therefore, although n = 3–6 individual biological replicates per group, for relatively abundant transcripts with large differences in expression level, *p*-values for expression changes were vanishingly small (e.g., <10^−10^).

**Figure 2:**
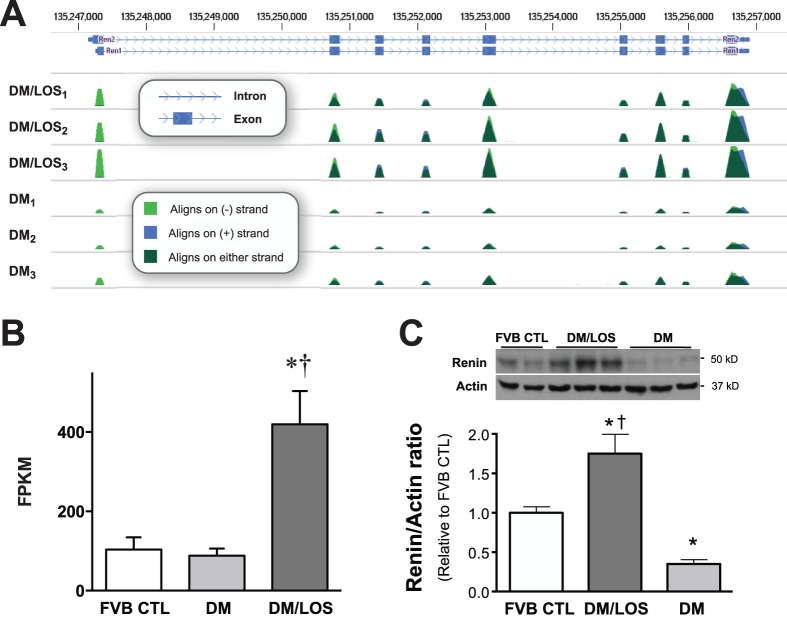
Data format and assay validation. Depiction of data alignment with canonical mouse genome at the *Ren1* (renin) locus showing the effect of losartan treatment on renin expression. **A**. Data from individual biological replicates (numbered as subscripts 1 through 3 for conditions DM and DM/LOS) are shown. Peaks (dark green) correspond to transcripts aligning with exons (blue boxes at top) within the *Ren1* gene; peak height reflects transcript abundance. At this resolution, individual transcript mappings are not discernible. **B**. Graphical depiction of data reflecting normalized renin mRNA abundance under each of the three experimental conditions. (Note that raw data for CTL are not shown in **A**.) These data are consistent with effective angiotensin-receptor blockade in the losartan-treated diabetic mice. **C**. Renal expression of renin protein in lysates prepared from kidneys of non-diabetic FVB mice and in OVE26 diabetic mice with (DM/LOS) or without (DM) treatment with losartan. Densitometric data are presented as renin to actin (loading control) ratios expressed, relative to FVB control (means ± SD). Representative blot is shown in the inset. *p<0.05 vs. FVB non-diabetic control; †p<0.01 vs. OVE26 (diabetic state).

An example of data visualization is shown in **Figure 2A**. Sequence reads align with exons but not introns, consistent with the transcriptome-based analysis. As anticipated with pharmacological angiotensin-receptor blockade, expression of renin (Ren1) mRNA was markedly upregulated (greater peak amplitude and area under curve). Quantitation at the individual transcript level is shown in **Figure 2B**; losartan treatment resulted in a greater than four-fold increase in renin mRNA expression relative to FVB control and to the untreated diabetic state. Increased expression at the level of renin protein was similar (at ∼five-fold) for losartan treatment relative to untreated diabetic state; however, the difference relative to non-diabetic control was more modest (approximately 75% increase). These data serve as an internal validation of this approach, and constitute a genetic signature of effective blockade of the renin-angiotensin-aldosterone system in the losartan-treated mice.

### Global Expression Patterns among the Three Experimental Groups

**Figure 3:**
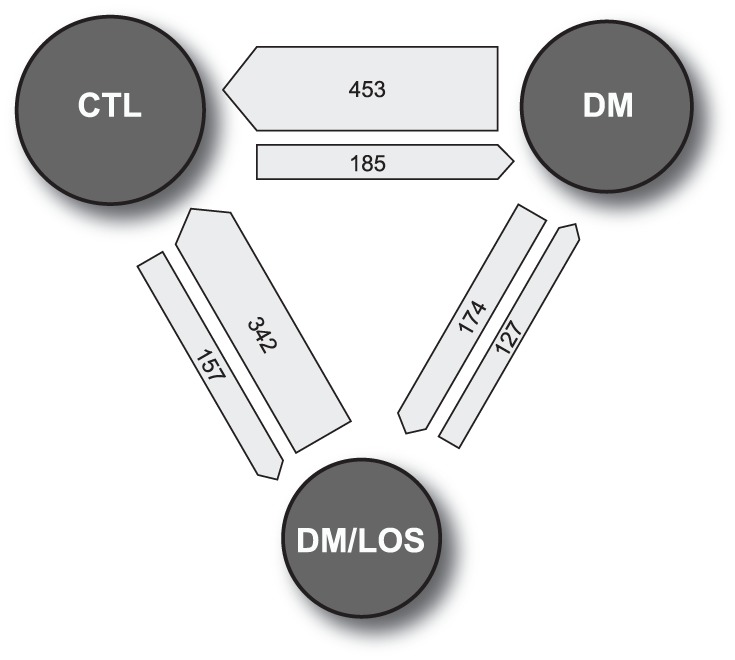
Gene expression changes by condition. Shown are statistically significant changes in gene expression between pairs of experimental conditions (CTL, non-diabetic state; DM, diabetic; DM/LOS, diabetes + losartan treatment). The **area** of the circle representing each condition is proportional to the total number of significant expression changes involving that condition (e.g., 453+185+342+157 = 1137 total for CTL). The **width** (thickness) of each arrow is proportional to the number of changes represented, and the **direction** of the arrow points to the condition in which expression is greater. For example, there were 453 gene products for which renal mRNA expression was greater under control conditions than in the diabetic state (or, re-stated, 453 gene products for which expression was lower in the diabetic state than in the non-diabetic state). The smallest number of differences was seen in the DM *vs*. DM/LOS dyad and the greatest number in the CTL *vs.* DM dyad.

There were 1438 significant differences in gene expression between any two experimental conditions (**Figure 3**). (**Table S26** in **File S2** is a list of all of the gene expression changes.) The most changes occurred between the non-diabetic control and diabetic mice, and the least between diabetes and diabetes + losartan (**Figure 3**). Of the 638 genes differentially regulated in the kidneys of diabetic *vs*. control mice, 185 were upregulated (**Table S1** in **File S1**) and 453 were downregulated (**Table S2** in **File S1**) with diabetes. Of the 301 genes differentially regulated at the RNA level in diabetic *vs.* losartan-treated diabetic mice, 174 were upregulated with treatment and 127 were downregulated with treatment (**Tables S9 and S10**, respectively, in **File S1**; see below). There were also 499 expression differences between control and treated diabetic mice – ∼22 percent fewer differences than were noted between control and diabetic mice.

**Figure 4:**
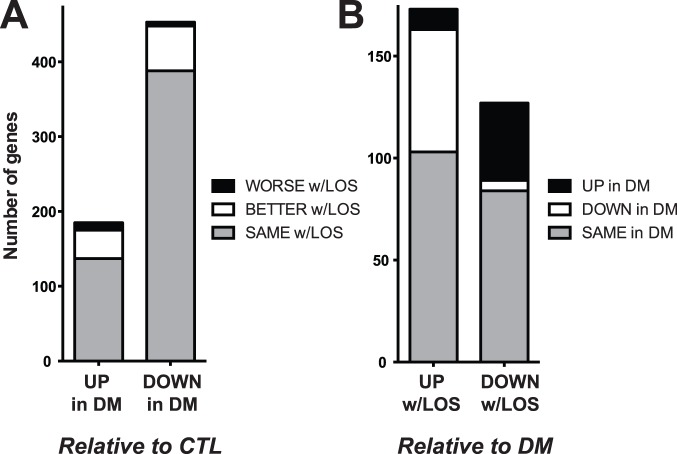
Diabetic changes in renal gene expression and the effect of losartan treatment. **A**. Effect of losartan treatment upon diabetes-associated changes in renal gene expression. Number of changes (UPregulation and DOWNregulation) in renal gene expression with diabetes, relative to control, grouped by the impact of losartan treatment. For most diabetic gene changes, there was no effect of losartan (SAME w/LOS; gray bars). Of the diabetic gene changes affected by losartan treatment, nearly all ameliorated the diabetic change (BETTER w/LOS; white bars) whereas very few exacerbated the change (WORSE w/LOS; black bars). **B**. Effect of diabetes upon gene expression changes associated with losartan treatment. Number of changes (UPregulation and DOWNregulation) in renal gene expression with losartan-treated diabetes (w/LOS), relative to untreated diabetes, assessed based upon the effect of diabetes alone. Most of the genes regulated by losartan were unaffected by diabetes alone (gray bars). These represent potential off-target effects of losartan treatment. However, of the genes regulated positively or negatively by losartan and also up- or down-regulated in diabetes, the vast majority of the losartan-associated changes ameliorated (white DOWN bar in UP column, and black UP bar in DOWN column) rather than exacerbated the change.

The impact of losartan on diabetes-associated expression changes was next assessed in a global fashion (**Figure 4**). In light of the salutary effect of angiotensin receptor blockade upon diabetic kidney disease [15], it was hypothesized that losartan treatment would associate with amelioration or restoration of diabetes-induced aberrant gene expression (i.e., in the direction of the control baseline). Considering only genes upregulated by the diabetic state (**Figure 4A**; left-most bar), 38 of these genes were significantly decreased with losartan (i.e., restored toward control; **Table S3** in **File S1**) whereas only 10 of these upregulated-in-diabetes genes exhibited a statistically significant further increase with losartan treatment (**Table S4** in **File S1**). Importantly, the majority of the diabetes-upregulated genes (137) were unaffected by losartan treatment (**Table S5** in **File S1**) – and may represent potential opportunities for future therapies (see Discussion). Re-stated, they may reflect elements of the diabetic kidney phenotype that are potentially unprotected by losartan treatment.

Of the 453 genes exhibiting downregulated renal expression in diabetes, a similar pattern of treatment-associated amelioration of the diabetic expression phenotype emerged (**Figure 4A**; right-most bar). Specifically, whereas 60 of these downregulated genes showed at least partial reversal/restoration toward control expression levels following treatment (**Table S6** in **File S1**), only 5 genes were further downregulated with losartan (**Table S7** in **File S1**). Also, as was the case with genes upregulated in diabetes, the majority (388) of these downregulated genes were unaffected by losartan treatment (**Table S8** in **File S1**), suggesting additional avenues for therapeutic intervention.

An analogous approach was taken to assess treatment-associated changes in renal gene expression, relative to the untreated diabetic state (**Figure 4B**). Expression of 173 genes was upregulated by losartan treatment in diabetes (relative to untreated diabetes; **Table S9** in **File S1**); of these 60 had been downregulated in the diabetic state whereas only 10 had been upregulated. Similarly, of the 127 genes downregulated by losartan (relative to untreated diabetes; **Table S10** in **File S1**), only five were already downregulated with diabetes whereas 38 had been upregulated with diabetes.

In aggregate, these data indicate that: 1) of the genes with renal expression influenced by the diabetic state, the majority are unaffected by early losartan treatment; and 2) of the diabetes-associated changes in renal gene expression that are impacted by losartan treatment, the great majority are in the direction of restoration of basal (control) levels of gene expression. Although correlative, this is consistent with the protective effect of angiotensin-receptor blockade. These specific patterns can be used to inform inquiries into the downstream mechanism(s) of action of renin-angiotensin-aldosterone system inhibition at the level of the kidney parenchyma, and to identify additional treatment opportunities targeting pathways perturbed in diabetes but unaffected by existing therapy (i.e., angiotensin-receptor blockade).

### Genes with Upregulated Kidney mRNA Expression in Diabetes

Statistically significant upregulations in renal gene expression in diabetes relative to the non-diabetic state ranged from a modest 61% increase to a 52-fold increase. The latter was for the product of the *Slc7a12* gene, the kidney-specific solute carrier family 7 cationic amino acid transporter, y+ system member 12 (see below). All genes exhibiting log_2_-fold change >2 (i.e., > four-fold increase in expression with diabetes) are shown in **Table 2**. Specific examples are discussed below and in the Discussion. A complete list of all genes significantly upregulated in diabetes is shown in **Table S1** in **File S1**. It is important to point out the role of controlling for multiple comparisons in the reporting of statistical significance for this data set.

**Table 2:** All genes exhibiting significantly upregulated renal expression (>4-fold increased) with diabetes (OVE26) relative to control (FVB).

gene_id	Control	DM	fold_change	p_value	gene_name
Slc7a12	0.23	11.99	52.33	0.00000000	solute carrier family 7 (cationic amino acid transporter, y+ system), member 12
Bhmt	0.42	10.29	24.46	0.00000000	betaine-homocysteine methyltransferase
Mosc1	0.05	1.15	21.27	0.00184219	similar to MOSC domain-containing protein 1, mitochondrial; MOCO sulphurase C-terminal domain containing 1
Gm10639	4.16	84.55	20.32	0.00000000	predicted gene 10639
Gsta2	32.14	500.32	15.57	0.00000000	glutathione S-transferase, alpha 2 (Yc2)
Rab30	1.39	14.75	10.64	0.00000000	RAB30, member RAS oncogene family
Gsta1	0.46	4.67	10.22	0.00025003	glutathione S-transferase, alpha 1 (Ya)
Kynu	1.20	10.34	8.60	0.00000000	kynureninase (L-kynurenine hydrolase)
Prlr	0.14	0.93	6.42	0.00000004	prolactin receptor
Bbox1	0.95	6.11	6.40	0.00000012	Butyrobetaine (gamma), 2-oxoglutarate dioxygenase 1 (gamma-butyrobetaine hydroxylase)
Aldh1a1	1.84	10.91	5.92	0.00000000	aldehyde dehydrogenase family 1, subfamily A1
Ugt2b34	0.38	2.08	5.43	0.00001060	UDP glucuronosyltransferase 2 family, polypeptide B34
Aldh1a7	2.07	11.04	5.33	0.00000000	aldehyde dehydrogenase family 1, subfamily A7
Cirbp	3.64	19.18	5.27	0.00000000	cold inducible RNA binding protein
Gbp8	0.68	3.35	4.94	0.00000161	Guanylate-binding protein 8
Cyp2c44	1.31	6.32	4.82	0.00000005	cytochrome P450, family 2, subfamily c, polypeptide 44
Stra6	0.34	1.61	4.73	0.00042375	stimulated by retinoic acid gene 6
Cpb2	0.49	2.23	4.58	0.00133766	carboxypeptidase B2 (plasma)
Acot3	0.96	4.22	4.40	0.00000033	acyl-CoA thioesterase 3
Txnip	21.14	91.96	4.35	0.00000000	thioredoxin interacting protein
Ell3	4.75	20.58	4.34	0.00000000	elongation factor RNA polymerase II-like 3
Angptl3	1.56	6.54	4.20	0.00006180	angiopoietin-like 3
Sgk1	31.46	130.30	4.14	0.00000000	serum/glucocorticoid regulated kinase 1
Lrat	0.15	0.62	4.13	0.00176822	lecithin-retinol acyltransferase (phosphatidylcholine-retinol-O-acyltransferase)
Gucy1a3	0.75	3.01	4.02	0.00000002	guanylate cyclase 1, soluble, alpha 3

### Genes Exhibiting Down-regulated Kidney mRNA Expression in Diabetes

Genes exhibiting log_2_-fold change <−2 (i.e., >75% reduction in expression) are shown in **Table 3**. Significant downregulation in expression with diabetes ranged from a modest 37% decrease to>99% decrease for Gm6300, the murine solute carrier family 7 (cationic amino acid transporter, y+ system), member pseudogene (see below). Some gene products were expressed in the non-diabetic state but were completely absent from the vehicle-treated (*Mup3, Ahsg, Mug1, Uox, Mup10, Mup21*) or losartan-treated (*Serpina3k, Mup3, Mug1, Mup9, Mup17, Uox, Ucp1, Mup21, LOC100048884*) diabetic kidney, so fold-induction could not be determined (**Table S26** in **File S2**). Many of the downregulated genes are stress-response genes of the heat shock protein family (see Discussion). This is perhaps unexpected, given that the hypertonic stress associated with systemic hyperglycemia should upregulate expression of heat shock proteins [16]; however, a glucose-mediated osmotic diuresis in the setting of uncontrolled diabetes may “wash out” the medullary concentration gradient and reduce medullary tonicity. Specific downregulated genes potentially relevant to diabetic pathophysiology are discussed below and in the Discussion). A complete list of all genes significantly downregulated in diabetes is shown in **Table S2** in **File S1**.

**Table 3:** All genes exhibiting significantly DOWNregulated (>75%) renal expression with diabetes (OVE26) relative to control (FVB) (n = 453).

gene_id	Control	DM	% decrease	p_value	Gene_Name
Gm6300	4.40	0.03	99.29	0.00000000	predicted gene 6300
Rmrp	16.48	0.36	97.81	0.00242795	RNA component of mitochondrial RNAase P
Ucp1	1.54	0.04	97.72	0.00026946	uncoupling protein 1 (mitochondrial, proton carrier)
Hspa1a	1.05	0.03	97.57	0.00004660	heat shock protein 1B; heat shock protein 1A; heat shock protein 1-like
Cyp2b10	2.64	0.15	94.18	0.00000003	cytochrome P450, family 2, subfamily b, polypeptide 10
Ctxn3	10.28	0.73	92.87	0.00000000	cortexin 3
Acsm3	418.73	31.57	92.46	0.00000000	acyl-CoA synthetase medium-chain family member 3
Srd5a2	8.20	0.70	91.51	0.00000000	steroid 5 alpha-reductase 2
Slc22a7	283.54	24.77	91.26	0.00000000	solute carrier family 22 (organic anion transporter), member 7
Tc2n	1.03	0.10	90.33	0.00010489	tandem C2 domains, nuclear
Cldn9	1.35	0.14	89.86	0.00023920	claudin 9
Sdf2l1	7.00	0.74	89.45	0.00000000	stromal cell-derived factor 2-like 1
Creld2	31.83	3.50	88.99	0.00000000	cysteine-rich with EGF-like domains 2
Angptl7	42.26	4.71	88.85	0.00000000	angiopoietin-like 7
Mtmr7	2.22	0.26	88.36	0.00000096	myotubularin related protein 7
Lipo1	1.51	0.18	88.28	0.00000009	lipase, member O1
Hspa1b	1.47	0.19	87.33	0.00000015	heat shock protein 1B; heat shock protein 1A; heat shock protein 1-like
Bcl6	2.81	0.38	86.29	0.00000000	B-cell leukemia/lymphoma 6
Gm15348	1.13	0.15	86.24	0.00000015	predicted gene 15348
Il34	23.18	3.21	86.14	0.00000000	interleukin 34
Anxa13	8.12	1.17	85.60	0.00000000	annexin A13
Slco1a1	208.21	30.70	85.26	0.00000000	solute carrier organic anion transporter family, member 1a1
Manf	52.95	7.81	85.24	0.00000000	mesencephalic astrocyte-derived neurotrophic factor
Slc22a28	32.21	4.83	84.99	0.00000000	solute carrier family 22, member 28
Serpinh1	8.62	1.37	84.05	0.00000000	serine (or cysteine) peptidase inhibitor, clade H, member 1
Mdk	16.92	2.72	83.91	0.00000000	Midkine
Ces2b	5.00	0.82	83.66	0.00000000	Carboxyesterase 2B
Hspa5	500.40	82.59	83.50	0.00000000	heat shock protein 5
Odc1	14.72	2.45	83.34	0.00000000	Ornithine decarboxylase, structural 1
Gm853	14.88	2.50	83.21	0.00000000	predicted gene 853
Cndp2	567.62	99.93	82.39	0.00000000	CNDP dipeptidase 2 (metallopeptidase M20 family)
C1qtnf3	196.68	34.72	82.35	0.00000000	C1q and tumor necrosis factor related protein 3
Cyp2j13	101.54	18.27	82.01	0.00000000	cytochrome P450, family 2, subfamily j, polypeptide 13
Rtp3	2.46	0.44	81.91	0.00000001	receptor transporter protein 3
Hspb1	2.47	0.45	81.90	0.00057488	heat shock protein 1
Slc22a30	195.67	35.43	81.89	0.00000000	solute carrier family 22, member 30
Gm13498	5.80	1.10	81.05	0.00000000	predicted gene 13498
C4a	1.55	0.30	80.84	0.00000000	Complement component 4A (Rodgers blood group)
Apoa1	2.49	0.48	80.55	0.00100158	apolipoprotein A–I
Cyp24a1	12.72	2.49	80.45	0.00000000	cytochrome P450, family 24, subfamily a, polypeptide 1
Rpl3l	1.78	0.35	80.14	0.00191032	ribosomal protein L3-like
Lpl	180.65	37.27	79.37	0.00000000	lipoprotein lipase; similar to Lipoprotein lipase precursor (LPL)
Gm2016	1.32	0.28	78.85	0.00039848	predicted gene 2016
Chordc1	50.01	10.64	78.72	0.00000000	cysteine and histidine-rich domain (CHORD)-containing, zinc-binding protein 1
B4galt5	5.92	1.27	78.52	0.00000000	UDP-Gal: betaGlcNAc beta 1,4-galactosyltransferase, polypeptide 5
Cpe	15.64	3.41	78.21	0.00000000	carboxypeptidase E; similar to carboxypeptidase E
Hsph1	21.69	4.75	78.12	0.00000000	heat shock 105 kDa/110 kDa protein 1
Hsd17b13	2.95	0.65	77.92	0.00204276	hydroxysteroid (17-beta) dehydrogenase 13
Hsp90ab1	775.83	172.91	77.71	0.00000367	heat shock protein 90 alpha (cytosolic), class B member 1
Gm5662	3.05	0.69	77.49	0.00000041	predicted gene, EG435337
Ldhd	206.41	46.61	77.42	0.00000000	lactate dehydrogenase D
Slc9a8	22.70	5.17	77.20	0.00000000	solute carrier family 9 (sodium/hydrogen exchanger), member 8
Ceacam2	8.15	1.91	76.58	0.00000004	carcinoembryonic antigen-related cell adhesion molecule 2
Gusb	16.22	3.84	76.31	0.00000000	glucuronidase, beta
Hsp90aa1	35.94	8.62	76.03	0.00000000	Heat shock protein 90, alpha (cytosolic), class A member 1
Sec14l3	3.18	0.77	75.76	0.00000000	SEC14-like 3 (S. cerevisiae)
Hspa8	399.77	97.00	75.74	0.00000000	similar to heat shock protein 8; heat shock protein 8
Tmem169	1.94	0.47	75.73	0.00000070	transmembrane protein 169
Dnajb1	16.82	4.12	75.53	0.00000000	DnaJ (Hsp40) homolog, subfamily B, member 1
Rhox6	2.83	0.70	75.37	0.00126947	reproductive homeobox 6
Znrf1	14.94	3.72	75.11	0.00000000	zinc and ring finger 1

### A Genetic Switch Involving Cationic Amino Acid Transporter Genes

**Figure 5:**
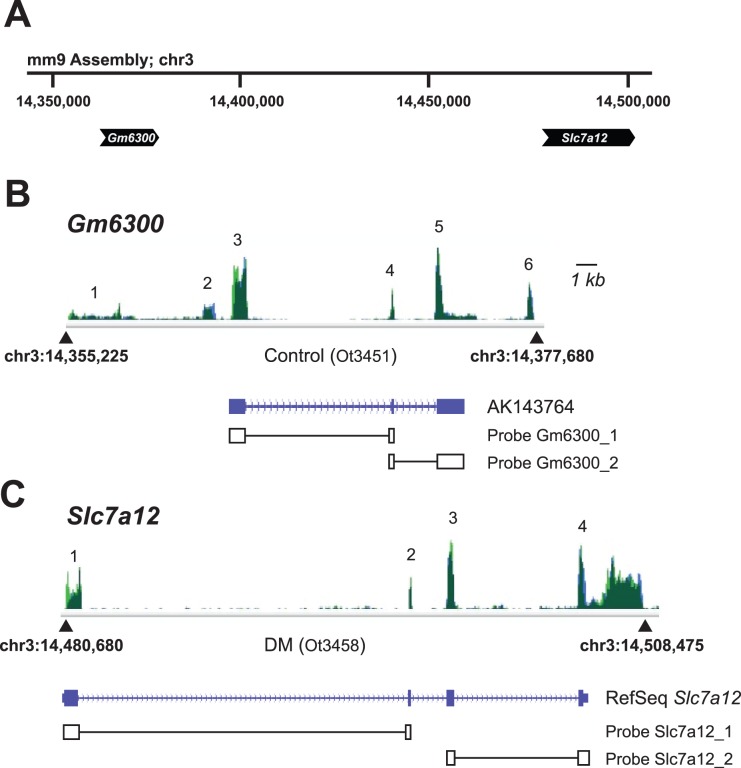
“Switching” expression from Gm6300 to Slc7a12 with induction of diabetes. RNA-Seq expression data for *Slc7a12* and for the “pseudogene” *Gm6300*, mapped on the mm9 murine genome assembly. The *Gm6300* pseudogene shares homology with *Slc7a12* and is ∼125 kb upstream of *Slc7a12* on chr3 (**A**); they appear to be paralogs. **B**. RNA-Seq expression data (green) for *Gm6300* are shown for Control condition (representative sample Ot3451). Also shown are mm9 (chromosome 3) genomic coordinates, and clone AK143764 (the only known cDNA or EST matching the RefSeq *Gm6300* gene; see Results). **C**. Depicted are the expressed regions of *Slc7a12* (in green; between chr3∶14,480,680–14,508,475) from a representative tracing of RNA-Seq transcripts from diabetic mouse kidney (sample Ot3458). Also shown is the exonic structure of the canonical *Slc7a12* reference sequence (RefSeq). For **B** and **C**, note the alignment of expressed transcripts with the predicted exons. Not shown, there was essentially no *Slc7a12* expression under Control conditions, and no *Gm6300* expression under diabetic conditions (see text and Figure 6). For the canonical gene sequences AK143764 and *Slc7a12*, exons are indicated by blue boxes, introns by an intervening blue line; CDS (in contrast to UTR) is designated by the taller blue boxes. Intron-spanning real-time PCR primer pairs (two primer/probe sets per gene, designated “_1” and “_2”) are diagrammed and are used for generating data in Figure 6. The scale bar denotes 1 kb for **B** and **C**. Exons are labeled 1 through 6 (**B**) and 1 through 4 (**C**), based upon mapped RNA-Seq reads.

The most upregulated (*Slc7a12*) and most downregulated (*Gm6300*) gene products in the diabetic state derive from adjacent and seemingly paralogous loci (**Figure 5A**); this is consistent with a “switch” from expression of the *Gm6300* “pseudogene” under non-diabetic control conditions to expression of the *Slc7a12* gene in the diabetic state. The pseudogene resides ∼125 kb “upstream” of *Slc7a12* (**Figure 5A**), shares sequence homology with the latter (see below), and appears to have been derived from it. However, there is only ∼70–80% homology at the nucleotide level across the subset of expressed regions (inferred exons; data not shown) such that this is highly unlikely to represent an alignment artifact within the DNAnexus analysis platform. Murine *Gm6300* corresponds to RefSeq NR_033591.2 and had been assigned the following provisional description: “Mus musculus predicted gene 6300 (Gm6300), non-coding RNA; Entrez Gene: 622229.” This is representative of the richness of detail that can be achieved with the RNA-Seq transcriptome-based approach and its lack of reliance upon pre-defined genes or genomic regions of interest.

The genomic region spanned by transcripts mapping to the *Gm6300* pseudogene (chr3∶14,355,250–14,377,700 in the mm9 murine genome assembly) under control conditions (**Figure 5B**) was aligned with the *Slc7a12* genomic region (chr3∶14,480,700–14,508,500) to which transcripts mapped under diabetic conditions (**Figure 5C**); regions of fairly high homology (up to 80% at the nucleotide level) were detected. A single murine cDNA reported in the NCBI databases maps to *Gm6300* (sequence ID: AK143764, cloned from a neonatal library); however, it reflects the use of fewer putative exons than we observed (exons 1, 2, and 3 of this clone appear to correspond to exons 3, 4, and 5 of the putative *Gm6300* transcript in the present study; **Figure 5B**). Furthermore, the putative exons of *Gm6300* are conserved across a wide range of vertebrate species – from orangutan to *Gallus* (UCSC Genome Browser; Multiz Alignments of 30 Vertebrates track; data not shown). The SLC7A12 protein is also known as Asc-2 ([17]; not to be confused with the transcription factor of the same name) and XAT1 [18]. The 3′-most 1.5-kb of the deduced *Gm6300* cDNA (exons 3, 4, 5, and 6) was 70–80% identical at the nucleotide level to the 5′-most 1.5 kb of the deduced *SLC7A12* transcript (reading in the same direction; exons 1, 2, 3, and part of exon 4).

In the EST Profile public domain utility (at http://www.ncbi.nlm.nih.gov), *SLC7A12* is reported to exhibit kidney-restricted expression; however, additional ESTs were detected in samples derived from liver. *Gm6300* ESTs were detected in mRNA derived from kidney and spleen.

**Figure 6:**
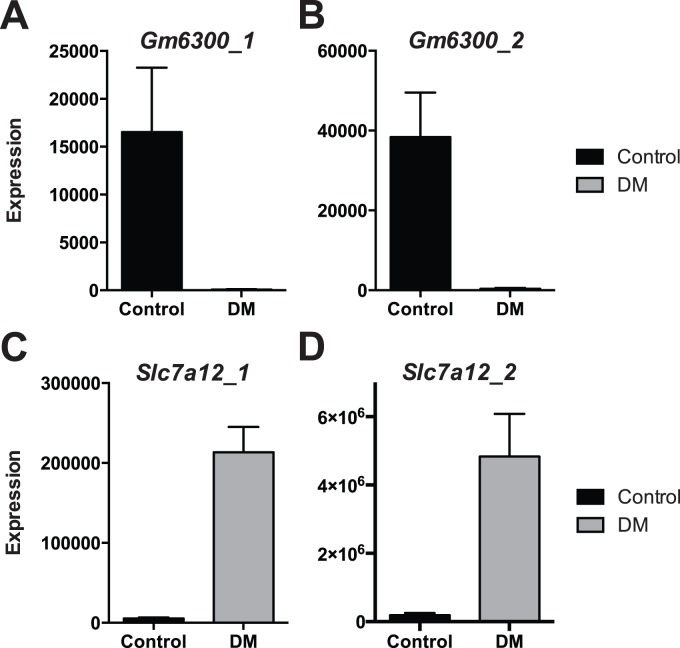
Differential expression of Gm6300 and Slc7a12 under control and diabetic conditions. Real-time PCR-based mRNA expression level (relative to 18S internal control) of *Gm6300* (**A, B**) and *Slc7a12* (**C, D**) using each of two different primer/probe sets per gene, and interrogating different putative expressed exons separated by an intervening intron. Intron-spanning amplicons are mapped in Figure 5. Probe sets (Invitrogen) were as follows: **Gm6300_1**, Mm03949676_m1; **Gm6300_2**, Mm03949677_m1; **Slc7a12_1**, Mm00499866_m1; **Slc7a12_2**, Mm01283157_m1. Data are expressed as mean +/− SEM for n = 3–5 biological replicates, with 2 technical replicates per sample (i.e., individual samples assayed in duplicate). Consistent with RNA-Seq data, expression of *Gm6300* was reduced by 99.5% (**A**) or 99% (**B**), whereas expression of *Slc7a12* was increased 39-fold (**C**) or 27-fold (**D**) with diabetes, depending upon probe set.

We used real-time PCR to confirm the diabetes-associated changes in renal expression of these paralogs (**Figure 6**). Via two different probe sets spanning intervening introns (i.e, specific for expression of a spliced transcript), abundant expression of *Gm6300* was evident under Control conditions, and diabetes was associated with a 99–99.5% (depending upon probe set) reduction in expression. In similar fashion, and again via two different intron-spanning probe sets, only low-level expression of *Slc7a12* was evident under Control conditions, and diabetes was associated with a 27–39-fold increase in transcript abundance. These data closely corroborated the RNA-Seq-based whole-transcriptome data.

In aggregate, these data indicate that: 1) *Gm6300* is likely not a pseudogene in this model; 2) *Gm6300* is transcribed in murine kidney only under control conditions; 3) the *Gm6300* primary transcript is spliced; and 4) the adjacent (and likely “parental”) *Slc7a12* gene is only transcribed in kidney in the diabetic state. Diabetes induced a near-total genetic “switch” from one paralogous gene product to another.

### A Module of Water-balance-associated Genes Affected by Diabetes and/or Losartan Treatment

**Figure 7:**
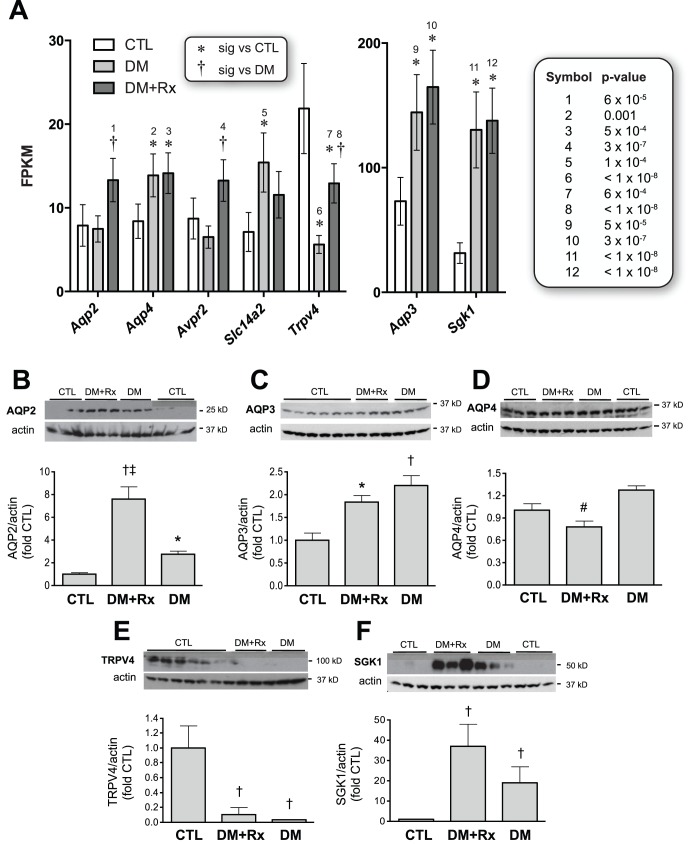
Effect of experimental conditions upon water-balance genes. A. A module of mammalian water balance genes was affected under one or more experimental conditions in the present model. Depicted genes are instrumental in water handling in the distal nephron, or in central osmosensing. Data are represented as FPKM (i.e., expression normalized within-sample). Depending upon gene product, data are scaled to relatively low (left panel) or high (right panel) maximal expression. Several diabetes-associated changes are unaffected by losartan treatment (*Aqp4*, *Aqp3*, *Sgk1*), presumably owing to an inability of losartan to correct osmotic derangement associated with hyperglycemia. In contrast, the diabetes-associated down-regulation in renal *Trpv4* expression is partially reversed with losartan treatment. *P*-values for the depicted differences (relative to CTL or to DM) are shown in the right panel and are keyed to numbers over the significance symbols. **B** through **E**. Immunoblots of protein expression (AQP2, AQP3, AQP4, TRPV4, and SGK1) in whole-kidney lysates prepared from FVB mice (“CTL”) and from losartan-treated (“DM+Rx”) or vehicle-treated (“DM”) OVE26 diabetic mice. Densitometry data, normalized to actin expression level, are shown graphically below the immunoblots for each protein. Significance levels are as follows: *p<0.5 and †p<0.01 relative to CTL (FVB) mice; #p<0.05 and ‡p<0.01 relative to untreated OVE26 diabetic mice (DM).

Unmitigated hyperglycemia increases plasma osmolality, and can also decrease renal medullary osmolality via an accompanying osmotic diuresis. We identified a relatively large number of genes relevant to water balance that were affected by diabetes and/or losartan treatment (**Figure 7**). These gene products are either instrumental in regulating water handling in the distal nephron (AQP2, AQP3, AQP4, SLC14A2, SGK1) or are putative constituents of the central and/or renal osmosensing mechanism (TRPV4). The diabetes-associated upregulation in renal expression of most of these gene products was unaffected by losartan treatment; this is consistent with the lack of a hypoglycemic effect with losartan therapy. Interestingly, the marked downregulation in renal TRPV4 expression observed in the diabetic state was significantly ameliorated by losartan treatment. We attempted to corroborate these findings at the protein level via immunoblotting. The directional changes across the experimental groups relative to non-diabetic FVB control mice were generally preserved for AQP2, AQP3, TRPV4, and SGK1; however, the pattern seen with AQP4 mRNA was not recapitulated at the protein level (**Figure 7**).

### Candidate Diabetes-associated Gene Products in the OVE26 Model

**Figure 8:**
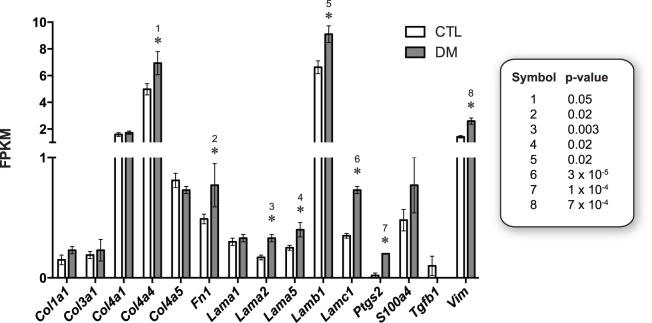
Candidate diabetes-associated gene products in the OVE26 model. Owing to the high significance threshold arising from the multiple comparisons-informed transcriptome-wide statistical approach, a number of expected diabetes candidate genes did not achieve significance at the transcriptome level in the present model, and were not represented in the functional annotation-based analysis. Raw expression data were queried for fifteen candidate genes and tested for nominal significance (P<0.05) via *t*-test (see text). Eight of the 15 gene products – particularly members of the laminin gene family – were upregulated in the OVE26 kidneys (relative to control kidneys). *P*-values for the depicted differences (relative to CTL or to DM) are shown in the right panel and are keyed to numbers over the significance symbols. *S100a4* is also known as *Fsp1*, and *Ptgs2* (prostaglandin-endoperoxide synthase 2, or prostaglandin G/H synthase and cyclooxygenase) is also known as cyclooxygenase-2 (COX-2).

The absence of a number of known diabetes-associated genes from the list of gene products upregulated by diabetes in the present model was unexpected, especially in light of the incipient changes seen by light microscopy (**Figure 1**). We speculated that the high threshold arising from the multiple comparisons-informed transcriptome-wide statistical approach accounted for this discrepancy. It was hypothesized that a number of these gene products would be modestly upregulated in the OVE26 kidneys, and would be expressed at a relatively low level (i.e., represented by comparatively few sequencing reads in the data sets). Expression data for 15 such genes relevant to the development of glomerulosclerosis, tubulointerstitial fibrosis, and microinflammation were examined and tested for nominal significance (P<0.05) via *t*-test. Eight of the 15 gene products – particularly members of the laminin gene family – were upregulated in the OVE26 kidneys (relative to non-diabetic control kidneys; **Figure 8**). As anticipated, most were expressed at low levels (FPKM<<1) and/or exhibited modest upregulation. These data underscore the ability of the present approach to corroborate prior findings in experimental diabetes, while highlighting the stringent requirements for achieving statistical significance in this model.

### Functional Annotation Analysis of Diabetic Changes in Kidney Gene Expression

An unbiased functional annotation-based analysis was performed to evaluate the totality of renal gene expression changes associated with the diabetic state or with losartan treatment. The DAVID platform [14] was used to identify over-representation of gene ontology terms (i.e., GO terms) among differentially regulated genes; terms were assigned to the domains of biological process, molecular function, or cellular compartment. Additional annotation terms (see Methods) were derived from the KEGG and other functional databases in the DAVID Bioinformatics Resource 6.7 online platform (National Institute of Allergy and Infectious Diseases (NIAID), NIH; http://david.abcc.ncifcrf.gov/home.jsp). Functional annotation data can be evaluated in terms of statistical significance (p-value) for the association with the annotation term, and in terms of the fold enrichment of annotation term-associated genes in a given sample. Owing to large differences in the numbers of genes assigned to each annotation term (and across uploaded gene lists), the two will not necessarily track in parallel. A number of biological terms were overrepresented among the genes dysregulated in diabetes; those with the lowest p-values are summarized below.

In the initial analysis, all diabetes-associated changes in gene expression were considered in aggregate (i.e., both up- and down-regulation events – which we termed dysregulation events; **Tables S1 and S2** in **File S1**). This was done because gene symbols mapping to an annotation term may be either up- or down-regulated in association with that keyword, biological process, etc. In our analysis, the top annotation keywords (all with p<10^−8^) for diabetes-associated changes in kidney gene expression were: the SP PIR keywords endoplasmic reticulum, stress response, oxidoreductase, chaperone, and nadp; the cellular compartment GO terms endoplasmic reticulum, endoplasmic reticulum part, vesicular fraction, and microsome; the biological process GO term oxidation reduction; and the KEGG pathway drug metabolism. Nearly all top hits relate to ER stress or oxidative stress. The full list of 474 annotations with p-value<0.1 is shown in **Table S11** in **File S1**. The results of this and other pathway analyses receive further attention in the Discussion, and the results of all functional annotation analyses are summarized in **Table 4**.

**Table 4:** *Summary of gene expression comparisons and corresponding functional annotations* (see text for details of individual analyses).

Comparison	Number of genes	List of gene symbols	Number of annot. terms	List of annot. terms	Annotation findings
Upregulated in diabetes relative to control	185	S-1	244	S-12	Oxidative stress
Downregulated in diabetes relative to control	453	S-2	338	S-13	ER stress and oxidative stress
Dysregulated in diabetes relative to control	638	S-1/S-2	474	S-11	ER stress and oxidative stress
Dysregulated in diabetes relative to control, and ameliorated with losartan treatment	98	S-3/S-6	52	S-14	ER, heat shock proteins, chaperones, unfolded protein response
Dysregulated in diabetes relative to control, and unaffected by losartan treatment	525	S-5/S-8	219	S-15	Oxidative stress
Dysregulated in diabetes relative to control, and exacerbated by losartan treatment	15	S-4/S-7	N/A	N/A	N/A [too few genes for meaningful analysis]
Upregulated in diabetes relative to control, and ameliorated with losartan treatment	38	S-3	52	S-16	No consistent pattern
Upregulated in diabetes relative to control, and unaffected by losartan treatment	137	S-5	ND	ND	ND
Upregulated in diabetes relative to control, and exacerbated with losartan treatment	10	S-4	N/A	N/A	N/A [too few genes for meaningful analysis]
Downregulated in diabetes relative to control, and ameliorated with losartan treatment	60	S-6	70	S-17	ER, heat shock proteins, chaperones, unfolded protein response
Downregulated in diabetes relative to control, and unaffected by losartan treatment	388	S-8	ND	ND	ND
Downregulated in diabetes relative to control, and exacerbated by losartan treatment	5	S-7	N/A	N/A	N/A [too few genes for meaningful analysis]
Upregulated in losartan treatment relative to untreated diabetes	173	S-9	193	S-22	ER, heat shock proteins, stress
Downregulated in losartan treatment relative to untreated diabetes	127	S-10	45	S-23	ER, heat shock proteins, stress
Dysregulated in losartan treatment relative to untreated diabetes	300	S-9/S-10	209	S-24	ER, heat shock proteins, stress
Unaffected by diabetes, and increased by losartan	103	S-18	89	S-20	Top hits are cancer-related with marginal significance
Unaffected by diabetes, and decreased by losartan	84	S-19	40	S-21	steroid/nuclear hormone receptor

A brief synopsis of the annotation findings is shown in the last column. For these analyses, dysregulation encompasses both up- and down-regulation of expression (see text).

“S-” refers to the Supplementary Table number (i**n File S1**) depicting the list of genes or results of functional annotation.

Genes up- and down-regulated with diabetes were then evaluated independently. Achieving the highest significance for annotation terms among genes upregulated in diabetic kidney (relative to the non-diabetic state), and expressed relative to the murine whole-genome background, were the following: KEGG pathways for drug metabolism and for metabolism of xenobiotics by cytochrome P450; the INTERPRO terms Glutathione S-transferase (alpha class), Glutathione S-transferase (N-terminal), and Glutathione S-transferase (C-terminal); the molecular function GO term glutathione transferase activity; the SP_PIR keyword oxidoreductase; and the biological process GO terms vitamin metabolic process, oxidation reduction, and response to toxin. Full details for the 244 annotation terms are shown in **Table S12** in **File S1**. Nearly all top hits relate to oxidative stress. As a validation of this pathway-based approach, a separate test for tissue specificity (UP_TISSUE) among the upregulated gene products was most significant for kidney among all tissues (p = 4×10^−9^).

Annotation terms achieving the highest significance among genes *down*regulated in diabetic kidney include the SP_PIR keywords for endoplasmic reticulum, stress response, chaperone, and oxidoreductase; the cellular compartment GOTERMs endoplasmic reticulum, endoplasmic reticulum part, endoplasmic reticulum lumen, melanosome, and pigment granule; and the biological process GO term protein folding. Full details of the 338 annotation terms are shown in **Table S13** in **File S1**. Most top hits are in the domains of ER stress and oxidative stress.

### Effects of Losartan on Diabetes-associated Changes in Renal Gene Expression

To explore the impact of early angiotensin receptor blockade on the spectrum of genes altered in the diabetic kidney, we compared kidney gene expression in diabetic mice without treatment to their counterparts receiving losartan. Compared with kidneys from the untreated diabetic mice, kidneys from losartan-treated diabetic mice showed 174 upregulated genes (**Table S9** in **File S1**) and 127 downregulated genes (**Table S10** in **File S1**).

Of the genes upregulated by losartan in diabetes (relative to untreated diabetes), functional annotation emphasized terms related to the ER, heat shock proteins, the unfolded protein response, and stress response(s) (**Table S22** in **File S1**). Of genes downregulated by losartan in diabetes (relative to untreated diabetes), functional annotation failed to emphasize a consistent pattern (**Table S23** in **File S1**). When all losartan-associated gene changes (relative to untreated diabetes) were considered in aggregate, functional annotation (**Table S24** in **File S1**) was similar to that seen with only the losartan-upregulated genes – driven by ER stress-related terms (e.g., heat shock proteins), but not oxidative stress.

A direct comparison of expression levels in diabetic kidney in the presence and absence of losartan treatment fails to account for the effect of diabetes relative to the non-diabetic (control) state; for example, it can not be determined whether a losartan-associated expression change occurs in the direction of amelioration or exacerbation of a diabetes-induced expression change. Therefore, losartan-associated expression changes were examined in the context of the effect of diabetes alone. The majority of genes with up- or down-regulated expression in response to diabetes were unaffected by losartan treatment (**Figure 3A**). Top hits in pathway analysis showed the following annotation terms: the KEGG pathway, drug metabolism; the SP PIR keyword, oxidoreductase; the InterPro keywords, glutathione S-transferase (alpha class), glutathione S-transferase (N-terminal), glutathione S-transferase (C-terminal), and lyase; the biological process GO term, oxidation reduction; the molecular function GO term, glutathione transferase activity; and the cellular compartment GO term, microsome. The full list of 219 annotations is shown in **Table S15** in **File S1**. These terms strongly relate to oxidative stress; as alluded to earlier, they collectively represent potential targets for additional and/or novel therapies.

We turned our attention to the subset of genes dysregulated in diabetes (i.e., either up- or down-regulated expression) *and* for which expression is partially or completely normalized with losartan treatment. There was remarkable functional uniformity among the most highly significant annotation terms for this group of 98 genes. The top hits in pathway-based analysis were: the SP_PIR keywords stress response and chaperone; the cellular compartment GO term endoplasmic reticulum lumen; the INTERPRO terms heat shock protein 70, heat shock protein Hsp70, heat shock protein 70 (conserved site); the molecular function GO terms protein domain specific binding and unfolded protein binding; and the PIR_SUPERFAMILY term chaperone HSP70. Therefore, losartan served to normalize the aberrant expression of ER stress-related gene products in diabetic kidney. The complete list of 52 annotation terms is shown in **Table S14** in **File S1**. Next, dysregulated genes were independently analyzed as upregulated or downregulated genes. The complete list of 38 genes upregulated in diabetic kidney and ameliorated – partially or completely – with losartan treatment is found in **Table S3** in **File S1**, with functional annotation results in **Table S16** in **File S1**. Of genes downregulated in diabetic kidney, losartan resulted in relative normalization of expression of 60 genes (listed in **Table S6** in **File S1**). Functional annotation is shown in **Table S17** in **File S1**, where terms relate predominantly to endoplasmic reticulum stress (heat shock proteins, chaperone function, etc.).

Most genes upregulated or downregulated in the diabetic kidney (relative to control) were unaffected by losartan treatment (**Tables S5** and **S8**, respectively, in **File S1**), and some were ameliorated with losartan (see above); however, a small subset of genes exhibited further exacerbation of dysregulation (i.e., additional up- or down-regulation) with losartan treatment. These included five genes further down-regulated with losartan (**Table S7** in **File S1**), and ten genes further upregulated with losartan (**Table S4** in **File S1**), relative to the diabetic state alone. For these groups, the gene number was too small to permit reliable functional annotation.

Lastly, we examined the effect of losartan on genes that were *not* affected by diabetes. Of the 103 genes unaffected by diabetes alone but exhibiting increased expression with diabetes plus losartan (**Table S18** in **File S1**), a fibrinogen chain (the gamma chain, encoded by *Fgg*) was among the most upregulated. This effect was unexpected given the role of fibrinogen as an inflammatory marker and cardiovascular risk factor in diabetes [19]. As previously mentioned, expression of renin was also markedly increased in the losartan-treated diabetic mice relative to untreated diabetic mice, consistent with effective inhibition of the RAS system in this model (**Figure 2**). In aggregate, these genes regulated by losartan but unaffected by diabetes could represent “off-target” effects, or effects related to potential side effects.

Unexpectedly, functional annotation on this gene list revealed a number of annotation terms potentially related to cancer: among the top nine hits of 89 reported annotation terms (of which some are likely not significant) were the p53 signaling pathway, bladder cancer, regulation of cell cycle, and response to radiation (**Table S20** in **File S1**). Conflicting data have emerged from epidemiological studies on the role of ARBs in cancer risk (reviewed in: [20]); there is no known mechanism through which such an effect may be exerted. Functional annotation on the list of genes unaffected by diabetes but down-regulated in the losartan-treated diabetic state (**Table S19** in **File S1**) did not reveal a similar signature (**Table S21** in **File S1**). The annotation analysis does not consider the direction of gene regulation and this is pivotal for ascertaining whether the upregulation events constitute a (putative) risk or protective signature vis-à-vis malignancy. Therefore, we used the EMBL-EBI Expression Atlas (http://www.ebi.ac.uk/gxa/) to query the direction of expression of each corresponding human gene (*CDKN1A*, *CCND1*, *MDM2*, *TIMP3*, *MYC*, *BTC*, *OBFC2A*, and *THBD*) in the malignancy signature derived from these annotation terms across a panel of human cancer-based expression profiles. Most gene products were upregulated in most cancers except for *CDKN1A* and *OBFC2A*. Importantly, for six of the eight genes, data were available for bladder cancer; all six (*CDKN1A*, *CCND1*, *MDM2*, *TIMP3*, *MYC*, and *THBD*) were strongly upregulated in that tumor. There were no data in this repository for renal cell carcinoma.

To ascertain whether these losartan-upregulated gene products associated with renal cell carcinoma, expression of the eight-gene signature was investigated in clear-cell renal cell carcinoma (ccRCC) through The Cancer Genome Atlas ([21]; http://cancergenome.nih.gov/). Expression at the RNA level was compared in ccRCC versus normal tissue (66 samples of each). Of the eight genes, five were significantly overexpressed in ccRCC relative to normal tissue: *CDKN1A* (p21) (1.6 fold); *CCND1* (cyclin D1) (5.4 fold); *MDM2* (1.7 fold); *MYC* (2.5 fold); and *OBFC2A* (1.7 fold). One was nominally overexpressed (*THBD*; 1.2-fold), whereas two of the genes were decreased in ccRCC: *TIMP3* (0.7 fold); and *BTC* (0.27 fold). Data and p-values are shown in **Table S25** in **File S1**. The increase in gene expression in kidney cancer versus normal kidney tissue for genes *MYC*, *CCND1*, *MDM2* and *CDKN1A* was also confirmed using the Oncomine database (www.oncomine.org). The other four genes were not clearly different in this analysis in Oncomine.

## Discussion

To our knowledge, this report is the first next-generation sequencing-based transcriptome analysis of renal gene expression in experimental diabetes. We tested 3–6 biological replicates per condition in a rigorous statistical approach to discern significant differences in expression at the RNA level. There was close correspondence among data within each experimental group (e.g., **Figure 2A, B**), which facilitated the detection of 1438 instances of differential gene expression across conditions. Effective blockade of the angiotensin receptor was pharmacologically achieved, based upon the marked upregulation in renin mRNA seen with losartan treatment (**Figure 2A, B**).

Among the broad patterns observed, it was notable that the most expression differences occurred between the non-diabetic and diabetic kidney; unexpectedly, however, over 70% of the diabetes-associated changes reflected downregulation and not upregulation in gene expression. The fewest expression differences occurred between the diabetic and treated-diabetic groups. Interestingly, most diabetes-associated changes in gene expression were unaffected by losartan treatment; however, of the diabetic changes impacted by losartan, the vast majority (87%) were ameliorating (i.e., tending to normalize or restore expression toward non-diabetic control levels) rather than exacerbating. This is consistent with the known salutary effect of angiotensin receptor blockade in diabetic kidney disease [15]. Diabetes-associated gene expression changes that are insensitive to losartan may represent novel opportunities for directed therapy. Importantly, annotation terms and/or biochemical processes most over-represented in these therapeutic “lacunae” relate primarily to oxidative stress. Therefore, the inability of losartan’s known antioxidant properties (e.g., [22]) to fully reverse the genetic hallmarks of oxidative stress in the present example of diabetic nephropathy suggest additional opportunities among even known target pathways.

Unexpectedly, losartan treatment affected expression of nearly 200 genes that were seemingly unaffected by diabetes alone (**Figure 3B**). While some or many of these expression differences may simply represent the effect of a statistical threshold applied across the RNA products of many thousands of genes, it is conceivable that a subset of these genes may reflect “off-target” effects of losartan independent of a diabetic protective effect. They may represent a consequence of losartan treatment in general, or an effect of losartan that is only manifest in the diabetic state. Alternatively, some members of this gene subset may reflect the renal consequences of losartan effects at extra-renal sites (cardiovascular, etc.); this is discussed further below (**Impact of losartan treatment**).

Strikingly, the most highly upregulated and the most highly downregulated two transcripts in diabetic kidney were encoded by adjacent genes that are likely paralogous – arising through gene duplication in the vertebrate lineage or earlier. The kidney tubule amino acid transporter Slc7a12 was expressed only in the diabetic kidneys, whereas the upstream paralog, Gm6300, was expressed only in non-diabetic kidneys; the ratio in each instance was 50–100∶1. Although this is highly unlikely to represent an artifact – based upon inspection of individual transcripts and the relatively modest sequence similarity across the paralogs – the role of this “switch” in the pathogenesis of, or response to, diabetic kidney disease remains uncertain. Slc7a12 (Asc-2 or XAT1) is a little-studied kidney-specific member of the SLC7 family of cationic and L-type amino acid transporters [17], [18]. Members of the SLC7 family can deliver L-arginine to promote nitric oxide synthesis [23]. The role of this process in the oxidative stress of diabetes is unclear. A member of this SLC7 gene family was implicated as a susceptibility locus for diabetic nephropathy in an ethnic Malay population [24]. The molecular basis for the paralog switching is of interest; possibilities include transcriptional regulation, mRNA stability, and/or epigenetic mechanisms. The nature of the local stimulus is also obscure – whether it is osmotic, hyperglycemic, or a response to oxidative stress or another stimulus.

### Upregulated Genes in OVE26 Kidney

Manual curation of the most strongly upregulated genes in diabetic kidney revealed genes previously linked to the pathophysiology of diabetic nephropathy or microvascular complications. Serum/glucocorticoid regulated kinase 1 *(Sgk1)* was increased 4.1 fold. SGK1 mediates diabetes-induced fibronectin production by tubular cells in vitro [25] and *Sgk1* knock-out mice made diabetic are relatively protected from the development of renal structural changes [26]. Upregulation of *Sgk1* can enhance sodium reabsorption in diabetic kidneys. Interestingly, increased *Sgk1* expression in our OVE26 model coincided with upregulation of *Stk39* (also known as SPAK, 2.38 fold); STK39 operates downstream of SGK1 in the tubular control of sodium reabsorption [27]. Moreover, increased *Sgk1* expression in diabetes appears to be kidney-specific [4]. A panel of genes involved in defense against oxidative stress was also upregulated, including the NAD(P)H dehydrogenase, quinone 1 (*Nqo1,* 3.0 fold), in parallel with genes coding for three glutathione S-transferase alpha isoforms (*Gsta1–3;* 10.2, 15.6, 3.3 fold, respectively). Insulin-like growth factor binding protein 1 (*Igfbp1;* 3.8 fold increased) is overexpressed in early-stage STZ-induced diabetes in rats, and is involved in the early renal hypertrophic response [28]. Epoxide hydrolase 1– the product of *Ephx1* (increased 3.6 fold) – is protective of nephropathy via its EET arachidonic acid metabolites [29]. CTGF (increased 2.8-fold here) was earlier implicated in the pathogenesis of nephropathy [30].

Our analysis also revealed highly upregulated genes not previously described in the context of diabetic complications, but rather suggested in a variety of clinical and experimental studies as putative contributors to cardiovascular pathophysiology or cardiovascular risk. Considering the parallels in processes operating in cardiovascular pathophysiology and in the pathogenesis of microvascular diabetic complications, these genes could be relevant in diabetic renal pathophysiology.

Kynureninase (*KYNU*) was upregulated 8.6-fold with DM; this enzyme catalyses conversion of the tryptophan metabolite kynurenin into L- alanine and anthranilate. Metabolomic profiling of serum biomarkers detected elevated levels of kynurenine in diabetic patients with kidney involvement relative to those without kidney involvement [31]. In addition, increased formation of kynurenines might contribute to development of metabolic syndrome via their apoptotic, neurotoxic, and pro-oxidative effects [32].

Thioredoxin-interacting protein (*Txnip,* 4.4-fold upregulated) has been implicated in the pathogenesis of diabetic nephropathy [33], and may mediate the ROS-dependent adverse effects of hyperglycemia on mesangial cells in vitro [34]. Angiopoietin-like 3 (*Angptl3,* 4.1-fold upregulated) is involved in lipid metabolism. Angptl3 mutations – and lower levels of the protein – cause familial combined hypolipidemia [35]. Polymorphisms in *Angptl3* associate with higher triglyceride levels and may represent a cardiovascular risk factor [36]. Particularly relevant to diabetic nephropathy, *Angptl3* is expressed on podocyte foot processes, and its increased expression correlates with their effacement. Angptl3 also plays a role in podocytic motility, in glomerular permeability, and in regulation of nephrin expression [37]. In addition, expression of a closely related angiopoetin-like gene (*Angptl4*) was also upregulated in OVE26 kidney.

### Downregulated Genes in OVE26 Kidney

Several genes with expression levels among those most dramatically reduced in diabetic kidney (≥67%) may be linked to the pathogenesis of complications in diabetes. Markedly reduced expression of uncoupling protein-1 (*Ucp-1,* 98% reduction in expression) is consistent with diabetes-induced mitochondrial dysfunction [38]. *Bcl6* (86% reduction in expression) is an antiapoptotic pro-survival gene. And the enzyme ornithine decarboxylase 1 (*Odc1,* 84% reduction in expression) was noted by Thompson, Blantz, and coworkers to be upregulated in diabetes and potentially contributed to aberrant renal growth and hyperfiltration [39]. A handful of genes are expressed in non-diabetic kidney but are completely absent in vehicle and/or losartan-treated diabetes (Table 26 in File S2), precluding their placement in a quantitative ranking. These include the major urinary protein (*Mup*) family members, *Mup3, Mup9, Mup10, Mup17*, and *Mup21*, as well as *Uox* (urate oxidase). This small list of gene symbols matches annotation terms for allergens, and for pheromone and odorant binding (data not shown). There are several dozen murine *Mup* genes and pseudogenes [40]. MUP-family proteins are hypothesized to convey pheromones from the circulation to the urine, and may regulate glucose metabolism [41]. *Mup1*– which lacked differential expression in the present study – is reduced in the liver and/or the circulation in murine models of obesity [42], [43] and caloric restriction [44], [45]. Hepatic overexpression of MUP1 protein or exogenous administration of purified MUP1 improved glycemic control in mouse models of diabetes [42], [43].

Other downregulated genes associate with diabetic renal pathophysiology by indirect evidence. Cortexin 3 (*Ctxn3*, 93% reduction in expression) – originally isolated from goat renal cortex – is a cardioprotective endogenous activator of eNOS that may play a role in the pathogenesis of hypertension [46]. Cortexin-3 expression was decreased in diabetic kidney, which also exhibits impaired endothelial NO generation. C1q/tumor necrosis factor-related protein-3 (*C1qtnf3*, also known as *Crtp*-*3,* 5.6 fold), a paralog of adiponectin, regulates glucose metabolism and innate immunity, and exerts anti-inflammatory and antifibrotic properties in several cell types [47]. Therefore, cortexin-3 downregulation in the diabetic kidney might contribute to these two major mechanisms of injury operative in diabetic nephropathy. Patients with metabolic syndrome display significantly higher levels of C1q/tumor necrosis factor-related protein-3, which correlate with cardiometabolic risk [48].

### Prior Genome-wide Investigations of Diabetes

A number of prior investigations have adopted an unbiased and/or whole-genome analysis of expression patterns in the context of diabetic kidney disease; most used DNA microarray analysis. Microarray studies are limited to the detection of transcripts hybridizing to a pre-designed – and generally commercial – array, whereas the present RNA-Seq approach entails direct sequencing of each transcript. This permits mapping to any site in the genome and is highly quantitative at the level of the individual transcript. It also enables the capture of subtle differences at the transcript level (e.g., novel transcriptional start sites, consequences of genetic polymorphisms, and evidence for RNA editing). Furthermore, transcriptome sequencing is nimble; as more is learned about the human genome, each new iteration of a “reference” genome results in more informative mapping of RNA sequence information.

Epstein and coworkers used microarray analysis to assess changes in renal gene expression at 2, 4, and 8 months of age in OVE26 mice [5]. At the age of 4 months (i.e., akin to the present studies), upregulation of inflammatory genes – such as C3 component of complement – was seen. In a subsequent study in the same model, the mice were subjected to uninephrectomy to accelerate renal injury [6]. The spectrum of genes with altered expression encompassed several proinflammatory pathways and those involved in renal fibrosis and glomerulosclerosis. In contrast, the present analysis did not reveal significant enrichment of proinflammatory or profibrotic genes. This difference may, in part, arise from our multiple comparison-informed statistical model, minimizing Type I errors at the expense of some Type II errors. In addition, this group’s analysis emphasized the evolution of gene expression differences from an early to a later timepoint. Importantly, however, altered expression of individual genes that might associate with a diabetes-induced pro-inflammatory state was observed in the present model, including RAGE ligand *s100A6* [49], *Txnip* [50], *Fas, C4a* [51], *CD55* [52], and *Gbp-8* [53]), as well as with glomerulosclerosis and tubulointerstitial fibrosis (*Ctgf*; [54], [55]).

It is important to point out that in addition to a different transcriptome profiling method (microarray), the Epstein studies also used a different bioinformatic algorithm than the present study. There is no ideal or uniformly accepted statistical approach for aggregating observed gene-level differences into broad biological inferences. RNA-Seq with biological replicates may prove more sensitive because the analysis is conducted at the level of the individual transcripts (see above). A detailed discussion of the advantages and disadvantages of biological functional annotation platforms is beyond the scope of this manuscript (e.g., [56]); however, these methods generally rely upon the same or similar catalogs of externally curated annotation terms.

A recent study by Jaffa et al [7] compared global renal gene expression 6 months after streptozotocin induction of diabetes in bradykinin receptor-knockout and wild-type mice. Comparison of the wild-type control and diabetic mice, relevant to the present analysis, showed reduced expression of *Cckar* [7], as in the present study, along with *Odc1* and *C1qtnf3*. *Cckar* was recently identified as a protective factor in diabetic kidney [57].

Knoll et al. [4] compared transcriptome profiles in renal cortex (and other tissues) in streptozotocin-treated rats two weeks after induction of diabetes. Consistent with the present study, they noted increased expression of *Sgk1* in diabetic kidneys, as described in greater detail above [4]. This group also observed altered expression of a range of thiol-related genes, and enrichment of genes involved in antioxidant defenses in the kidney (see below).

### Themes Emerging from the Functional Annotation-based Analysis

In the present study, functional annotation-based analysis of genes differentially expressed in diabetic kidney revealed two dominant themes: 1) dysregulation of genes associated with oxidative stress (e.g., the annotation terms for oxidoreduction, GST proteins, NADP, etc.); and 2) dysregulation of genes associated with ER stress (e.g., the annotation terms for ER compartment, chaperone function, heat shock proteins, etc.).

Although upregulation of genes coding for enzymes directly involved in ROS production was not detected, there was markedly reduced expression of *Ucp1* (Uncoupling protein 1– mitochondrial, proton carrier) with diabetes. Importantly, this is consistent with enhanced mitochondrial ROS production. Ucp1 is pivotal to maintaining a low voltage gradient across the mitochondrial membrane, which becomes elevated upon exposure to the diabetic milieu. Accordingly, overexpression of Ucp1 in endothelial cells blunted high-glucose-induced mitochondrial superoxide production, and expression of RAGE and calgranulins [38]. Other dysregulated oxidative stress-related genes include *Nqo1, Gsta* family members, and *Hmox1.* These genes, along with *Ephx1, Dnajc3,* and *Dnajb11,* are regulated by Nf-E2-related factor 2 (Nrf2) [58], [59]. Nrf2 is a transcription factor exhibiting increased expression and function in response to oxidative stress. Activated Nrf2 binds to antioxidant response elements (AREs) in the genes encoding enzymes essential for protection from oxidative stress [59]. Interestingly, not all Nrf2-responsive genes were upregulated in diabetic kidney in the present model: *Hmox1* expression was decreased and may reflect an incomplete compensatory response to DM-induced oxidative stress. Increased expression of Nrf2-dependent genes has been shown in diabetic tissues [59], and is among the reputed mechanisms of action for the potential nephroprotectant, bardoxolone [60].

Further supporting the primacy of oxidative stress in diabetic kidney, we observed dysregulation of many genes in the large cytochrome P450 (*Cyp*) family; these can act as a tissue source of ROS production [61]. The protein *Cyp4a* is required for 20-HETE generation from arachidonic acid. This lipid figures prominently in cardiovascular and renal pathophysiology; for example, it upregulates NADPH oxidase in podocytes and promotes their apoptosis in OVE26 mice [62]. Within the Cyp4a subfamily we detected increased expression of polypeptide 31 (*Cyp4a31*, 2.3 fold) and decreased expression of polypeptides −12a and −12b (*Cyp4a12a*, *Cyp4a12b*); the net effect vis-à-vis ROS generation is unclear. Adding further complexity, one of the most highly upregulated gene products was *Ephx1*. *Ephx1* is Nrf2-responsive and degrades epoxyeicosatrienoic acids (EETs). EET compounds are arachidonic acid metabolites generated by CYP family members; in contrast to 20-HETEs, they are nephroprotective [63]. Using the *Ephx1* knockout mice, Chen et al have demonstrated a beneficial role for this gene product in experimental diabetic nephropathy [29].

The second broad theme to emerge is the dysregulated renal expression in diabetes of genes matching annotation terms for ER cellular compartment, ER stress, and chaperones. Under physiological conditions, correct protein folding is ensured via a combination of molecular chaperones, foldases, and lectins [64]. Improperly folded proteins are targeted for degradation. When unfolded proteins accumulate, ER stress ensues. ER stress activates the “unfolded protein response,” an integrated signal transduction pathway that transmits information about net protein folding status in the ER to the nucleus and the cytosol to help restore ER homeostasis. This pathway is essential for protecting the cell from environmental and metabolic stressors. ER stress and induction of the unfolded protein response (i.e., upregulation of expression of ER stress-associated gene products) occurs in renal cells exposed to hyperglycemia in vitro; the phenomenon has also been observed in kidneys from experimental diabetic models and in kidneys from diabetic patients [65]–[67]. Studies in rodent models of diabetes have documented increased expression of renal ER stress markers, and activation or posttranslational modification of mediators of the unfolded protein response (BiP*/Hspa5,* Chop/Gadd153/*Ddit3*; [66], [67]). Some reports linked these changes to diabetes-induced apoptosis of kidney cells [66]. Lindenmeyer et al showed upregulation of Chop/Gadd153/*Ddit3*, BiP/*Hspa5*, Hypoxia up-regulated 1 (*Hyou1*), calnexin (*Canx*) and *Xbp1* in diabetic kidney via microarray analysis [65]; however, to our knowledge dysregulation of ER-associated genes has not previously been reported as a dominant finding in genome-wide expression studies focusing on diabetic nephropathy.

The present studies in the OVE26 model displayed decreased – not increased – expression of the genes associated with ER stress and the unfolded protein response, relative to the non-diabetic mice, as evidenced by the prevailing annotation terms and inspection of the gene lists. It’s not possible to conclude whether the downregulated expression of chaperones and ER stress-associated proteins in diabetic kidney is an adaptive or maladaptive response. However, downregulation of most of the genes in this domain is suggestive of a diabetes-induced defect or even collapse of the unfolded protein response, potentially accompanied by unchecked accumulation of unfolded proteins. This possibility is further supported by evidence suggesting protective roles of ER stress-associated genes in various pathological conditions [68], [69], including kidney disease [70]; analogous observations are emerging from studies of diabetic retinopathy [71].

In addition, the over-representation of ER stress-related genes among those restored toward control levels by losartan treatment (**Tables S14** and **S17** in **File S1**; see below) – and their relative absence from the gene lists unaffected by losartan treatment (e.g., **Table S15** in **File S1**) – suggests that the downregulation seen with diabetes is pathological. That is, losartan treatment – which almost completely blocked the development of diabetic albuminuria (**Table 1**) – was associated with restoration of expression of these gene products in the direction of control levels. Because losartan did not affect glycemic control (**Table 1**), it would not be expected to impact glycosuria or the resultant osmotic diuresis and the attendant decrease in medullary tonicity. Therefore, although expression of the heat shock protein family of chaperones is under osmotic control [16] and these proteins are abundantly expressed in the hypertonic kidney medulla (reviewed in: [72]), their downregulation with diabetes in the present model is likely independent of any osmotic effects.

### Impact of Losartan Treatment

Most diabetic changes in gene expression were unaffected by losartan, and the majority of losartan-associated changes served to ameliorate rather than exacerbate diabetes-associated changes (**Figure 3**). There was also evidence for specific protective effects of ARB treatment. Functional annotation of losartan-responsive genes showed enrichment in ER lumen cellular components. This, along with inspection of the up- and down-regulated gene lists, suggested that losartan corrected abnormalities in the spectrum of ER stress/unfolded protein response-associated genes. Angiotensin II can stimulate ER stress [73], [74], and ARBs afford protection from ER stress [70], [75]–[77]. Inhibition of the type 1 angiotensin II receptor can ameliorate ER stress in the diabetic kidney, and block ER stress-induced apoptosis in an obstructive model of renal interstitial fibrosis [70]. Moreover, in agreement with the present data, antifibrotic effects of receptor inhibition were associated with upregulation of *Xbp1* [70]. Considering the well established role of AngII in pro-oxidant signaling and ROS generation [22], [77], [78], it is perhaps unexpected that losartan did not exhibit a more universal effect on oxidative stress genes in the present model. It is possible that metabolic derangements in this model were sufficiently severe to drive oxidative stress independent of angiotensin receptor action. Perhaps consistent with this interpretation, losartan treatment failed to prevent the development of microalbuminuria in normalbuminuric type 1 diabetic patients [79]. Other possible explanations include: 1) small (but still meaningful) differences in expression level of oxidative stress-responsive genes between the groups; and 2) unusually low abundance of these transcripts in both groups.

A small subset of genes exhibited further exacerbation of dysregulation (i.e., additional up- or down-regulation) with losartan treatment. One of the five genes further down-regulated with losartan (**Table S7** in **File S1**), frizzled-related protein (*Frzp*), is an endogenous inhibitor of Wnt pathway that has been associated with DM-induced podocytopathy [reviewed in: [80]]. Meprin 1 beta (*Mep1b*), also in this group, is a metalloendopeptidase in the renal proximal tubule brush-border membrane that colocalizes with ACE (angiotensin converting enzyme). Decreased renal expression of *Mep1b* was seen in experimental models of diabetes [81]. Moreover, the level of Mep1b expression is inversely related to the severity of nephropathy in diabetic mice [81]. Furthermore, a Mep1b polymorphism associate with DM risk in Pima Indians [82].

Limited but provocative data related losartan use in this diabetic model to the upregulation of a gene expression signature matching annotation terms for malignancy. This conclusion is tempered by the absence of a non-diabetic losartan study arm. Nonetheless, even if the present losartan gene signature is unique to the diabetic kidney and is not relevant to other ARB indications (e.g., hypertension or congestive heart failure), it’s still potentially meaningful from a public health perspective. Importantly, most of these genes were similarly upregulated in public database profiles for urothelial cancer and for clear-cell renal cell carcinoma. Three of these eight genes encode the well-known oncoproteins MYC, cyclin D1 and MDM2. MYC is a basic helix-loop-helix transcription factor that increases expression of many genes, but particularly those involved in cell proliferation [83]. Cyclin D1 promotes the G1/S transition of the cell cycle and thereby also increases proliferation [84]. MDM2 serves as a ubiquitin ligase for the p53 tumor suppressor [85], which protects genome stability and also negatively regulates the cell cycle. Thus, cells that exhibit increased expression of these three genes would be expected to be proliferative and prone to genomic instability. MYC [86], [87] and cyclin D1 are also well-known contributors in the pathogenesis of ccRCC [88], [89], while MDM2 has been implicated in urothelial cancer [90]. In contrast, CDKN1A, which encodes cyclin-dependent kinase inhibitor p21 that slows the cell cycle, is also overexpressed in losartan-treated diabetic kidney. This may act to counter the proliferative effects of MYC, cyclin D1 and MDM2; however, and concerningly, CDKN1A is also overexpressed in clear-cell renal cell carcinoma relative to normal kidney. Thus, the overall picture with losartan treatment is one that raises suspicion for increased oncogenic potential in the diabetic kidney.

Association of ARB therapy with cancer remains controversial (reviewed in: [20]). In 2010, the FDA issued the following statement: “FDA’s meta-analysis of 31 randomized controlled trials comparing ARBs to other treatment found no evidence of an increased risk of incident (new) cancer, cancer-related death, breast cancer, lung cancer, or prostate cancer in patients receiving ARBs (http://www.fda.gov/Safety/MedWatch/SafetyInformation/SafetyAlertsforHumanMedicalProducts/ucm219185.htm). In June, 2011, the FDA further concluded that “…treatment with an ARB medication does not increase a patientns risk of developing cancer” (http://www.fda.gov/Drugs/DrugSafety/ucm257516.htm). Notably, longitudinal follow-up was limited in these randomized controlled trials for drug efficacy, and these analyses do not specifically address kidney or urothelial cancer. In 2013, a senior regulator at the FDA unsuccessfully sought stronger warnings for cancer risk with this drug class, as reported by the Wall Street Journal (http://online.wsj.com/news/articles/SB10001424127887324682204578515172395384146). A recent comprehensive report on ARB use and cancer noted an adjusted odds ratio of 1.10 for urologic cancer, with confidence intervals of 0.56–2.18 [91]. Intervals this broad fail to exclude a clinically meaningful increased risk for urologic cancer. A meta-analysis of randomized controlled trials of ARB usage detected a significant increase in new cancer risk [92]; importantly, the follow-up intervals for the included studies were only 1.9–4.8 years and results were not reported by cancer site. A more comprehensive analysis of 15 ARB RCTs, conducted by the trialists themselves, makes no mention of kidney or urothelial cancer in their otherwise negative report [93]. Bangalore et al aggregated data from 70 RCTs representing a spectrum of antihypertensives [94]. Although their conclusion “refutes a 5–10% relative increase in the risk of cancer or cancer-related death with the use of ARBs,” they allow that their data “showed a consistent harmful effect of the ACEi and ARB combination on cancer risk” [94]. Again, and importantly, these data reflect the very limited follow-up interval for these clinical trials; with similar follow-up, the risk of even tobacco – with its long latency period (e.g., [95]) – might go undetected. A second meta-analysis on ARB usage failed to detect an increased risk for cancer; however, the subgroup analysis showed an increased risk for kidney cancer and melanoma [96]. In contrast, some animal studies have suggested that ARBs may protect from progression and metastasis of existing kidney and bladder cancer [97], [98]. In a retrospective assessment of 279 patients who underwent resection for known urothelial cancer, ACEi or ARB use associated with improved five-year metastasis-free survival rate [99]. We feel that the present data contribute to the conversation about long-term ARB safety. Although there are no compelling human data to support oncogenic potential of ARB treatment in the kidney or other organ systems, we believe that ongoing vigilance vis-à-vis kidney and urothelial cancer is warranted.

There are a number of important **limitations** to this study. The OVE26 diabetic phenotype – with hyperglycemia present essentially from birth – does not temporally recapitulate the human Type I diabetic phenotype. Nonetheless, the renal histological lesions are notably similar, albeit at more advanced stages of nephropathy than were studied here. The effect of losartan was tested in isolation and not in combination with other antidiabetic therapies (e.g., insulin supplementation); therefore, some or many of the diabetic gene changes unaffected by losartan may represent the renal consequences of hyperglycemia, per se. With the exception of the model-validating experiments with renin and a range of potentially osmotically-responsive gene products (Fig. 7), expression was addressed at the RNA level and not the protein level. Further studies aimed at specific biological processes affected by diabetes – and particularly those that are not ameliorated by angiotensin-receptor blockade – will warrant detailed investigation at the protein level and in other diabetic models. Our use of whole kidney – in contrast to a specific renal tissue, tubule segment, or cell type – reduces sensitivity for changes in tissue specific-transcripts, particularly those of low abundance. The present findings are most relevant to renal tubular cells that comprise ∼90% of renal mass. We adopted this approach for rapidity of sample collection and processing (to minimize mRNA degradation), and to avoid the introduction of confounding by virtue of imprecise or inconsistent selection of the relatively poorly demarcated renal tissue zones (e.g., cortex, outer medulla, inner medulla, etc.). We also elected to omit a losartan-treated non-diabetic group. Emphasis was placed upon achieving sufficient biological replicates to ensure statistically robust conclusions in the non-diabetic vs. diabetic state, and in the treated vs. untreated diabetic state. Future, more targeted studies with less costly methodology will permit use of a 2×2 factorial design. Lastly, the high significance threshold necessitated by our multiple comparisons-informed statistical approach (and designed to minimize Type I errors) increased the likelihood of Type II errors. This was evident when nominal (p<0.05) significance was demonstrated for some gene products previously implicated in diabetic renal disease (Figure 8) but not detected by our whole-transcriptome approach. Lastly, it is important to emphasize that the observed gene expression changes represent associations; it can not be directly inferred whether they reflect adaptive or maladaptive events.

In conclusion, early changes in the kidneys of OVE26 murine model center around dysregulation of genes related to ER stress and oxidative stress; losartan treatment appears to favorably impact the former but not the latter. Of the gene expression changes occurring in diabetes, the impact of losartan tended to be ameliorating rather than exacerbating. The diabetic state activated a genetic switch; the amino acid transporter, *Slc7a12*, was newly expressed and its adjacent paralog, *Gm6300*, was almost completely shut off. And lastly, the over-representation of cancer-associated annotation terms among genes unaffected by diabetes but upregulated by diabetes plus losartan – while speculative – warrants further study.

## Supporting Information

File S1 **Tables S1 through S25 are combined in this file. Tables S1 through S24 include the lists of gene names and their gene functional annotations for the between-group comparisons discussed in the text.** **Table 4**, appearing within the manuscript itself and not in the supporting information, provides a key to the supplementary table(s) supporting each conclusion. **Table S25** is not indexed in **Table 4**; it shows expression of genes matching cancer-related annotation terms (from **Table S24**) in clear-cell renal cell carcinoma (ccRCC) and in adjacent normal tissue in human tumor/kidney samples from The Cancer Genome Atlas KCC database (http://cancergenome.nih.gov/).
(DOCX)

File S2 **Table S26, the list of all 1438 gene expression differences exhibiting pair-wise statistical significance between experimental groups.** Data are presented as: gene ID; locus; sample_1 (experimental group of sample 1); sample_2 (experimental group of sample 2); check status (data comparison quality control); value_1 (FPKM value for sample_1); value_2 (FPKM value for sample_2); log2fold change; test_stat; p_value; and q_value for the comparisons, and statistical significance. Note that “sample” in this table refers to aggregated data for that entire experimental condition (i.e., n = 3–6 biological replicates).
(XLS)
