# Supplementary material for: Transcriptome-Based Analysis of Kidney Gene Expression Changes Associated with Diabetes in OVE26 Mice, in the Presence and Absence of Losartan Treatment
Source: PLoS One. 2014 May 14;9(5):e96987. doi: 10.1371/journal.pone.0096987 (PMC4020814; doi:10.1371/journal.pone.0096987)
Supplement: File S1 — Tables S1 through S25 are combined in this file. Tables S1 through S24 include the lists of gene names and their gene functional annotations for the between-group comparisons discussed in the text. Table 4 , appearing within the manuscript itself and not in the supporting information, provides a key to the supplementary table(s) supporting each conclusion. Table S25 is not indexed in Table 4 ; it shows expression of genes matching cancer-related annotation terms (from Table S24) in clear-cell renal cell carcinoma (ccRCC) and in adjacent normal tissue in human tumor/kidney samples from The Cancer Genome Atlas KCC database (http://cancergenome.nih.gov/). (DOCX) [file pone.0096987.s001.docx]

**Suppl. Table S1** All genes exhibiting significantly upregulated renal expression with diabetes (OVE26) relative to control (FVB) (n = 185)

| **gene_id** | **Control** | **DM** | **fold_change** | **p_value** | **gene_name** |
| --- | --- | --- | --- | --- | --- |
| Slc7a12 | 0.23 | 11.99 | 52.33 | 0.00000000 | solute carrier family 7 (cationic amino acid transporter, y+ system), member 12 |
| Bhmt | 0.42 | 10.29 | 24.46 | 0.00000000 | betaine-homocysteine methyltransferase |
| Mosc1 | 0.05 | 1.15 | 21.27 | 0.00184219 | similar to MOSC domain-containing protein 1, mitochondrial; MOCO sulphurase C-terminal domain containing 1 |
| Gm10639 | 4.16 | 84.55 | 20.32 | 0.00000000 | predicted gene 10639 |
| Gsta2 | 32.14 | 500.32 | 15.57 | 0.00000000 | glutathione S-transferase, alpha 2 (Yc2) |
| Rab30 | 1.39 | 14.75 | 10.64 | 0.00000000 | RAB30, member RAS oncogene family |
| Gsta1 | 0.46 | 4.67 | 10.22 | 0.00025003 | glutathione S-transferase, alpha 1 (Ya) |
| Kynu | 1.20 | 10.34 | 8.60 | 0.00000000 | kynureninase (L-kynurenine hydrolase) |
| Prlr | 0.14 | 0.93 | 6.42 | 0.00000004 | prolactin receptor |
| Bbox1 | 0.95 | 6.11 | 6.40 | 0.00000012 | Butyrobetaine (gamma), 2-oxoglutarate dioxygenase 1 (gamma-butyrobetaine hydroxylase) |
| Aldh1a1 | 1.84 | 10.91 | 5.92 | 0.00000000 | aldehyde dehydrogenase family 1, subfamily A1 |
| Ugt2b34 | 0.38 | 2.08 | 5.43 | 0.00001060 | UDP glucuronosyltransferase 2 family, polypeptide B34 |
| Aldh1a7 | 2.07 | 11.04 | 5.33 | 0.00000000 | aldehyde dehydrogenase family 1, subfamily A7 |
| Cirbp | 3.64 | 19.18 | 5.27 | 0.00000000 | cold inducible RNA binding protein |
| Gbp8 | 0.68 | 3.35 | 4.94 | 0.00000161 | Guanylate-binding protein 8 |
| Cyp2c44 | 1.31 | 6.32 | 4.82 | 0.00000005 | cytochrome P450, family 2, subfamily c, polypeptide 44 |
| Stra6 | 0.34 | 1.61 | 4.73 | 0.00042375 | stimulated by retinoic acid gene 6 |
| Cpb2 | 0.49 | 2.23 | 4.58 | 0.00133766 | carboxypeptidase B2 (plasma) |
| Acot3 | 0.96 | 4.22 | 4.40 | 0.00000033 | acyl-CoA thioesterase 3 |
| Txnip | 21.14 | 91.96 | 4.35 | 0.00000000 | thioredoxin interacting protein |
| Ell3 | 4.75 | 20.58 | 4.34 | 0.00000000 | elongation factor RNA polymerase II-like 3 |
| Angptl3 | 1.56 | 6.54 | 4.20 | 0.00006180 | angiopoietin-like 3 |
| Sgk1 | 31.46 | 130.30 | 4.14 | 0.00000000 | serum/glucocorticoid regulated kinase 1 |
| Lrat | 0.15 | 0.62 | 4.13 | 0.00176822 | lecithin-retinol acyltransferase (phosphatidylcholine-retinol-O-acyltransferase) |
| Gucy1a3 | 0.75 | 3.01 | 4.02 | 0.00000002 | guanylate cyclase 1, soluble, alpha 3 |
| Igfbp1 | 0.64 | 2.44 | 3.83 | 0.00166825 | insulin-like growth factor binding protein 1 |
| Cyp27b1 | 3.22 | 12.30 | 3.82 | 0.00000000 | cytochrome P450, family 27, subfamily b, polypeptide 1 |
| Gc | 5.59 | 20.60 | 3.69 | 0.00000000 | group specific component |
| Upp2 | 14.54 | 52.16 | 3.59 | 0.00000000 | uridine phosphorylase 2 |
| Ephx1 | 10.38 | 36.99 | 3.56 | 0.00000000 | epoxide hydrolase 1, microsomal |
| Ifi27l2a | 5.08 | 17.98 | 3.54 | 0.00044300 | interferon, alpha-inducible protein 27 like 2A |
| Lypd6 | 0.47 | 1.66 | 3.52 | 0.00022139 | LY6/PLAUR domain containing 6 |
| Lgals4 | 3.89 | 13.54 | 3.48 | 0.00000007 | Lectin, galactose binding, soluble 4 |
| Gm129 | 3.73 | 12.95 | 3.48 | 0.00000014 | predicted gene 129 |
| Angptl4 | 8.19 | 28.34 | 3.46 | 0.00000000 | angiopoietin-like 4 |
| Gsto1 | 2.90 | 10.01 | 3.45 | 0.00000323 | glutathione S-transferase omega 1 |
| March10 | 0.69 | 2.37 | 3.43 | 0.00006280 | Membrane-associated ring finger (C3HC4) 10 |
| Hacl1 | 20.95 | 71.13 | 3.39 | 0.00000000 | 2-hydroxyacyl-CoA lyase 1 |
| Mcm5 | 0.37 | 1.22 | 3.34 | 0.00129129 | minichromosome maintenance deficient 5, cell division cycle 46 (S. cerevisiae) |
| Pdk4 | 1.74 | 5.68 | 3.27 | 0.00000002 | pyruvate dehydrogenase kinase, isoenzyme 4 |
| Gsta3 | 27.66 | 90.40 | 3.27 | 0.00000000 | glutathione S-transferase, alpha 3 |
| Akr1c12 | 3.56 | 11.28 | 3.17 | 0.00000167 | aldo-keto reductase family 1, member C12 |
| Uroc1 | 1.93 | 6.12 | 3.17 | 0.00000003 | urocanase domain containing 1 |
| Dixdc1 | 0.40 | 1.24 | 3.12 | 0.00010370 | DIX domain containing 1 |
| Acaa2 | 97.90 | 297.70 | 3.04 | 0.00000001 | acetyl-Coenzyme A acyltransferase 2 (mitochondrial 3-oxoacyl-Coenzyme A thiolase) |
| Spp2 | 207.06 | 624.89 | 3.02 | 0.00000000 | secreted phosphoprotein 2 |
| Svopl | 0.62 | 1.85 | 3.00 | 0.00207958 | SV2 related protein homolog (rat)-like |
| Clca1 | 0.50 | 1.50 | 3.00 | 0.00171736 | chloride channel calcium activated 1 |
| Nqo1 | 27.84 | 83.43 | 3.00 | 0.00000000 | NAD(P)H dehydrogenase, quinone 1 |
| Abcb1b | 0.54 | 1.59 | 2.96 | 0.00026065 | ATP-binding cassette, sub-family B (MDR/TAP), member 1B |
| 2310001A20Rik | 12.69 | 37.46 | 2.95 | 0.00000000 | RIKEN cDNA 2310001A20 gene |
| Cp | 3.31 | 9.69 | 2.92 | 0.00000378 | ceruloplasmin |
| Ftcd | 4.89 | 14.19 | 2.90 | 0.00000000 | formiminotransferase cyclodeaminase |
| Dnase1 | 465.28 | 1348.19 | 2.90 | 0.00239405 | deoxyribonuclease I |
| Mt2 | 23.22 | 67.15 | 2.89 | 0.00000003 | metallothionein 2 |
| Mt1 | 367.36 | 1055.89 | 2.87 | 0.00000012 | metallothionein 1 |
| Ctgf | 2.81 | 7.99 | 2.85 | 0.00000050 | connective tissue growth factor |
| Mgmt | 3.06 | 8.63 | 2.82 | 0.00077559 | O-6-methylguanine-DNA methyltransferase |
| Tmem86b | 1.64 | 4.62 | 2.81 | 0.00148775 | transmembrane protein 86B |
| Slc22a29 | 3.36 | 9.39 | 2.80 | 0.00000023 | solure carrier family 22 member 29 |
| Akr1c13 | 9.28 | 25.82 | 2.78 | 0.00000001 | aldo-keto reductase family 1, member C13 |
| Fam134b | 34.79 | 96.73 | 2.78 | 0.00000449 | family with sequence similarity 134, member B |
| Hccs | 1.52 | 4.20 | 2.77 | 0.00004780 | holocytochrome c synthetase |
| Gsta4 | 13.53 | 36.46 | 2.70 | 0.00000002 | glutathione S-transferase, alpha 4 |
| Trf | 6.84 | 18.42 | 2.69 | 0.00000013 | transferrin |
| Elovl6 | 0.95 | 2.56 | 2.69 | 0.00000268 | ELOVL family member 6, elongation of long chain fatty acids (yeast) |
| 8430408G22Rik | 11.69 | 31.25 | 2.67 | 0.00000798 | RIKEN cDNA 8430408G22 gene |
| Xpnpep2 | 1.06 | 2.84 | 2.67 | 0.00006590 | X-prolyl aminopeptidase (aminopeptidase P) 2, membrane-bound |
| 2900092D14Rik | 1.31 | 3.43 | 2.62 | 0.00010381 | RIKEN cDNA 2900092D14 gene |
| Slc6a19 | 61.89 | 161.65 | 2.61 | 0.00000015 | solute carrier family 6 (neurotransmitter transporter), member 19 |
| Lpar2 | 0.52 | 1.35 | 2.60 | 0.00034163 | lysophosphatidic acid receptor 2 |
| Lyplal1 | 63.83 | 165.70 | 2.60 | 0.00000001 | lysophospholipase-like 1 |
| 1300015D01Rik | 1.15 | 2.98 | 2.59 | 0.00008580 | RIKEN cDNA 1300015D01 gene |
| Stc1 | 1.07 | 2.74 | 2.57 | 0.00008460 | stanniocalcin 1 |
| Mpzl2 | 2.58 | 6.62 | 2.56 | 0.00000074 | myelin protein zero-like 2 |
| Fbxo21 | 1.62 | 4.14 | 2.56 | 0.00000482 | F-box protein 21 |
| Ddc | 14.66 | 36.79 | 2.51 | 0.00000002 | dopa decarboxylase |
| Utp20 | 2.85 | 7.11 | 2.50 | 0.00000002 | UTP20, small subunit (SSU) processome component, homolog (yeast) |
| Slc38a3 | 11.17 | 27.81 | 2.49 | 0.00000004 | solute carrier family 38, member 3 |
| Adamts5 | 0.30 | 0.75 | 2.48 | 0.00151242 | A disintegrin-like and metallopeptidase (reprolysin type) with thrombospondin type 1 motif, 5 (aggrecanase-2) |
| Cyp2a5 | 10.84 | 26.50 | 2.44 | 0.00000014 | Cytochrome P450, family 2, subfamily a, polypeptide 5 |
| Gpihbp1 | 7.23 | 17.54 | 2.43 | 0.00010209 | GPI-anchored HDL-binding protein 1 |
| Plk3 | 1.69 | 4.08 | 2.42 | 0.00035196 | polo-like kinase 3 (Drosophila) |
| Cyp2d26 | 33.71 | 81.33 | 2.41 | 0.00000013 | cytochrome P450, family 2, subfamily d, polypeptide 26 |
| Agmo | 2.04 | 4.89 | 2.40 | 0.00021121 | alkylglycerol monooxygenase |
| Amotl1 | 0.69 | 1.66 | 2.40 | 0.00002080 | angiomotin-like 1 |
| Stk39 | 1.71 | 4.08 | 2.38 | 0.00005460 | serine/threonine kinase 39, STE20/SPS1 homolog (yeast) |
| Prim1 | 1.96 | 4.56 | 2.33 | 0.00224158 | DNA primase, p49 subunit |
| Herc3 | 1.52 | 3.55 | 2.33 | 0.00002130 | hect domain and RLD 3 |
| Cyp4a31 | 50.20 | 116.47 | 2.32 | 0.00021699 | Cytochrome P450, family 4, subfamily a, polypeptide 31 |
| Tipin | 3.11 | 7.21 | 2.32 | 0.00104855 | timeless interacting protein |
| Cpm | 1.52 | 3.50 | 2.31 | 0.00001400 | carboxypeptidase M |
| Pttg1 | 6.99 | 16.08 | 2.30 | 0.00185242 | pituitary tumor-transforming gene 1 |
| Pdk2 | 65.68 | 150.89 | 2.30 | 0.00000607 | pyruvate dehydrogenase kinase, isoenzyme 2 |
| Slc28a1 | 3.00 | 6.83 | 2.28 | 0.00001390 | solute carrier family 28 (sodium-coupled nucleoside transporter), member 1 |
| Npl | 17.21 | 39.01 | 2.27 | 0.00000022 | N-acetylneuraminate pyruvate lyase |
| Agpat9 | 2.26 | 5.11 | 2.26 | 0.00008060 | 1-acylglycerol-3-phosphate O-acyltransferase 9 |
| G0s2 | 9.14 | 20.61 | 2.26 | 0.00008970 | G0/G1 switch gene 2 |
| Slc2a5 | 22.64 | 50.96 | 2.25 | 0.00000051 | solute carrier family 2 (facilitated glucose transporter), member 5 |
| Prps2 | 9.76 | 21.91 | 2.25 | 0.00000012 | phosphoribosyl pyrophosphate synthetase 2 |
| Fas | 4.34 | 9.73 | 2.24 | 0.00014058 | Fas (TNF receptor superfamily member 6) |
| Il5ra | 1.35 | 3.01 | 2.23 | 0.00038788 | interleukin 5 receptor, alpha |
| Sh3bp5 | 2.62 | 5.84 | 2.23 | 0.00010001 | SH3-domain binding protein 5 (BTK-associated) |
| BC089597 | 78.25 | 173.67 | 2.22 | 0.00000371 | cis-retinol/3alpha hydroxysterol short-chain dehydrogenase-like precursor |
| Muc20 | 2.99 | 6.61 | 2.21 | 0.00006610 | mucin 20 |
| Slc5a9 | 1.30 | 2.86 | 2.20 | 0.00021668 | solute carrier family 5 (sodium/glucose cotransporter), member 9 |
| Hebp1 | 43.17 | 94.87 | 2.20 | 0.00000061 | heme binding protein 1 |
| 6030446N20Rik | 1.44 | 3.15 | 2.19 | 0.00178948 | RIKEN cDNA 6030446N20 gene |
| Slc5a12 | 39.55 | 86.49 | 2.19 | 0.00010818 | solute carrier family 5 (sodium/glucose cotransporter), member 12 |
| Hddc2 | 7.55 | 16.38 | 2.17 | 0.00100709 | HD domain containing 2 |
| Slc14a2 | 7.12 | 15.43 | 2.17 | 0.00010858 | solute carrier family 14 (urea transporter), member 2 |
| Lass4 | 1.10 | 2.36 | 2.13 | 0.00169018 | LAG1 homolog, ceramide synthase 4 |
| Maoa | 0.98 | 2.09 | 2.13 | 0.00153663 | monoamine oxidase A |
| Acer2 | 1.45 | 3.08 | 2.13 | 0.00029889 | alkaline ceramidase 2 |
| Mgst1 | 53.25 | 113.17 | 2.13 | 0.00000223 | microsomal glutathione S-transferase 1 |
| Tob2 | 1.82 | 3.86 | 2.12 | 0.00014137 | transducer of ERBB2, 2 |
| Fbp2 | 17.50 | 36.82 | 2.10 | 0.00000480 | fructose bisphosphatase 2 |
| Pnpla7 | 3.00 | 6.29 | 2.10 | 0.00001230 | patatin-like phospholipase domain containing 7 |
| Fgg | 5.44 | 11.33 | 2.08 | 0.00043947 | fibrinogen gamma chain |
| Acnat1 | 15.87 | 32.92 | 2.07 | 0.00004740 | Acyl-coenzyme A amino acid N-acyltransferase 1 |
| Acot2 | 10.42 | 21.62 | 2.07 | 0.00003350 | acyl-CoA thioesterase 2 |
| Cdk18 | 3.01 | 6.22 | 2.06 | 0.00010011 | Cyclin-dependent kinase 18 |
| F2r | 1.65 | 3.40 | 2.06 | 0.00087901 | coagulation factor II (thrombin) receptor |
| Fga | 6.88 | 14.15 | 2.06 | 0.00008820 | fibrinogen alpha chain |
| Apob | 0.63 | 1.29 | 2.06 | 0.00011556 | apolipoprotein B |
| Scg5 | 10.32 | 21.11 | 2.05 | 0.00004640 | secretogranin V |
| 3010026O09Rik | 8.58 | 17.42 | 2.03 | 0.00016796 | RIKEN cDNA 3010026O09 gene |
| Fabp3 | 17.73 | 35.88 | 2.02 | 0.00018986 | fatty acid binding protein 3, muscle and heart |
| S100a6 | 13.28 | 26.84 | 2.02 | 0.00035780 | S100 calcium binding protein A6 (calcyclin) |
| Clec2h | 10.40 | 21.00 | 2.02 | 0.00000937 | C-type lectin domain family 2, member h; predicted gene 4698 |
| Tgfbr3 | 2.72 | 5.48 | 2.02 | 0.00001620 | transforming growth factor, beta receptor III |
| Acox2 | 46.72 | 94.10 | 2.01 | 0.00032206 | acyl-Coenzyme A oxidase 2, branched chain |
| Slc26a6 | 2.19 | 4.40 | 2.01 | 0.00064771 | solute carrier family 26, member 6 |
| Anxa1 | 5.72 | 11.45 | 2.00 | 0.00048639 | annexin A1 |
| Folh1 | 13.89 | 27.72 | 2.00 | 0.00003490 | folate hydrolase |
| Tln2 | 3.51 | 6.96 | 1.98 | 0.00000928 | talin 2 |
| Aqp3 | 73.03 | 144.35 | 1.98 | 0.00004910 | aquaporin 3 |
| Eif4ebp3 | 23.77 | 46.80 | 1.97 | 0.00033757 | Eukaryotic translation initiation factor 4E binding protein 3 |
| Klf10 | 10.82 | 21.14 | 1.95 | 0.00001620 | Kruppel-like factor 10 |
| Iyd | 50.96 | 99.37 | 1.95 | 0.00002310 | iodotyrosine deiodinase |
| Prom1 | 5.86 | 11.43 | 1.95 | 0.00024695 | prominin 1 |
| Fam13a | 8.26 | 16.08 | 1.95 | 0.00001590 | family with sequence similarity 13, member A |
| Nupr1 | 59.61 | 115.35 | 1.94 | 0.00002370 | nuclear protein 1 |
| Fmo4 | 5.56 | 10.76 | 1.93 | 0.00034841 | flavin containing monooxygenase 4 |
| Aldoc | 7.82 | 15.12 | 1.93 | 0.00013299 | aldolase C, fructose-bisphosphate |
| Spon1 | 1.15 | 2.21 | 1.93 | 0.00095297 | spondin 1, (f-spondin) extracellular matrix protein |
| Btg2 | 3.63 | 7.00 | 1.93 | 0.00020878 | B-cell translocation gene 2, anti-proliferative |
| Mfsd7c | 5.31 | 10.22 | 1.92 | 0.00005220 | major facilitator superfamily domain containing 7C |
| Txndc16 | 1.68 | 3.18 | 1.90 | 0.00157173 | thioredoxin domain containing 16 |
| Sparcl1 | 3.45 | 6.54 | 1.90 | 0.00034998 | SPARC-like 1 |
| Serping1 | 21.56 | 40.80 | 1.89 | 0.00003070 | serine (or cysteine) peptidase inhibitor, clade G, member 1 |
| Msl3 | 10.58 | 20.02 | 1.89 | 0.00004030 | male-specific lethal 3 homolog (Drosophila) |
| Cbr1 | 11.96 | 22.53 | 1.88 | 0.00035541 | carbonyl reductase 1 |
| Plat | 17.18 | 32.05 | 1.87 | 0.00004630 | plasminogen activator, tissue |
| Fbln5 | 2.54 | 4.73 | 1.86 | 0.00023820 | fibulin 5 |
| Ktn1 | 2.08 | 3.87 | 1.86 | 0.00078958 | kinectin 1 |
| Muc1 | 6.43 | 11.82 | 1.84 | 0.00037584 | mucin 1, transmembrane |
| Lgals3bp | 6.84 | 12.55 | 1.84 | 0.00029349 | lectin, galactoside-binding, soluble, 3 binding protein |
| Nudt7 | 10.04 | 18.34 | 1.83 | 0.00223334 | nudix (nucleoside diphosphate linked moiety X)-type motif 7 |
| Rgs5 | 25.62 | 46.57 | 1.82 | 0.00009730 | regulator of G-protein signaling 5 |
| Akr1d1 | 4.43 | 8.04 | 1.81 | 0.00068558 | aldo-keto reductase family 1, member D1 |
| Iqgap2 | 1.40 | 2.54 | 1.81 | 0.00202874 | IQ motif containing GTPase activating protein 2 |
| Car14 | 93.65 | 168.78 | 1.80 | 0.00220129 | carbonic anhydrase 14 |
| Mogat2 | 15.29 | 27.55 | 1.80 | 0.00017391 | monoacylglycerol O-acyltransferase 2 |
| Klf13 | 3.07 | 5.54 | 1.80 | 0.00031691 | Kruppel-like factor 13 |
| Elmod3 | 10.56 | 18.82 | 1.78 | 0.00025981 | ELMO/CED-12 domain containing 3 |
| Abcd4 | 5.04 | 8.97 | 1.78 | 0.00087339 | ATP-binding cassette, sub-family D (ALD), member 4 |
| Gm16379 | 54.34 | 96.63 | 1.78 | 0.00037510 | predicted gene 16379 |
| Mif | 160.35 | 284.90 | 1.78 | 0.00030086 | macrophage migration inhibitory factor |
| Lrrc66 | 5.54 | 9.84 | 1.78 | 0.00042336 | leucine rich repeat containing 66 |
| Cyp2d22 | 15.59 | 27.58 | 1.77 | 0.00033568 | cytochrome P450, family 2, subfamily d, polypeptide 22 |
| Pex11a | 18.89 | 32.81 | 1.74 | 0.00131294 | peroxisomal biogenesis factor 11 alpha |
| Tmem43 | 5.07 | 8.77 | 1.73 | 0.00111366 | transmembrane protein 43 |
| Mfsd4 | 9.45 | 16.28 | 1.72 | 0.00145223 | major facilitator superfamily domain containing 4 |
| Lgr4 | 13.38 | 23.03 | 1.72 | 0.00071540 | leucine-rich repeat-containing G protein-coupled receptor 4 |
| Sepw1 | 48.90 | 83.36 | 1.70 | 0.00053561 | selenoprotein W, muscle 1 |
| Rpp21 | 62.20 | 105.69 | 1.70 | 0.00076826 | ribonuclease P 21 subunit (human) |
| Lpin2 | 5.18 | 8.61 | 1.66 | 0.00164487 | lipin 2 |
| Aqp4 | 8.41 | 13.88 | 1.65 | 0.00106571 | aquaporin 4 |
| Prodh2 | 23.59 | 38.94 | 1.65 | 0.00110941 | proline dehydrogenase (oxidase) 2 |
| Sfxn2 | 5.30 | 8.73 | 1.65 | 0.00208762 | similar to Sideroflexin-2; sideroflexin 2 |
| Mal2 | 20.13 | 33.12 | 1.65 | 0.00145365 | mal, T-cell differentiation protein 2 |
| Inpp5k | 14.81 | 24.20 | 1.63 | 0.00130234 | inositol polyphosphate 5-phosphatase K |
| Slc6a8 | 9.47 | 15.31 | 1.62 | 0.00136672 | solute carrier family 6 (neurotransmitter transporter, creatine), member 8 |
| Ptrf | 9.79 | 15.78 | 1.61 | 0.00203233 | polymerase I and transcript release factor |

**Suppl. Table S2** All genes exhibiting significantly DOWNregulated renal expression with diabetes (OVE26) relative to control (FVB) (n = 453)

| **gene_id** | **Control** | **DM** | **% decrease** | **p_value** | **Gene_Name** |
| --- | --- | --- | --- | --- | --- |
| Gm6300 | 4.40 | 0.03 | 99.29 | 0.00000000 | predicted gene 6300 |
| Rmrp | 16.48 | 0.36 | 97.81 | 0.00242795 | RNA component of mitochondrial RNAase P |
| Ucp1 | 1.54 | 0.04 | 97.72 | 0.00026946 | uncoupling protein 1 (mitochondrial, proton carrier) |
| Hspa1a | 1.05 | 0.03 | 97.57 | 0.00004660 | heat shock protein 1B; heat shock protein 1A; heat shock protein 1-like |
| Cyp2b10 | 2.64 | 0.15 | 94.18 | 0.00000003 | cytochrome P450, family 2, subfamily b, polypeptide 10 |
| Ctxn3 | 10.28 | 0.73 | 92.87 | 0.00000000 | cortexin 3 |
| Acsm3 | 418.73 | 31.57 | 92.46 | 0.00000000 | acyl-CoA synthetase medium-chain family member 3 |
| Srd5a2 | 8.20 | 0.70 | 91.51 | 0.00000000 | steroid 5 alpha-reductase 2 |
| Slc22a7 | 283.54 | 24.77 | 91.26 | 0.00000000 | solute carrier family 22 (organic anion transporter), member 7 |
| Tc2n | 1.03 | 0.10 | 90.33 | 0.00010489 | tandem C2 domains, nuclear |
| Cldn9 | 1.35 | 0.14 | 89.86 | 0.00023920 | claudin 9 |
| Sdf2l1 | 7.00 | 0.74 | 89.45 | 0.00000000 | stromal cell-derived factor 2-like 1 |
| Creld2 | 31.83 | 3.50 | 88.99 | 0.00000000 | cysteine-rich with EGF-like domains 2 |
| Angptl7 | 42.26 | 4.71 | 88.85 | 0.00000000 | angiopoietin-like 7 |
| Mtmr7 | 2.22 | 0.26 | 88.36 | 0.00000096 | myotubularin related protein 7 |
| Lipo1 | 1.51 | 0.18 | 88.28 | 0.00000009 | lipase, member O1 |
| Hspa1b | 1.47 | 0.19 | 87.33 | 0.00000015 | heat shock protein 1B; heat shock protein 1A; heat shock protein 1-like |
| Bcl6 | 2.81 | 0.38 | 86.29 | 0.00000000 | B-cell leukemia/lymphoma 6 |
| Gm15348 | 1.13 | 0.15 | 86.24 | 0.00000015 | predicted gene 15348 |
| Il34 | 23.18 | 3.21 | 86.14 | 0.00000000 | interleukin 34 |
| Anxa13 | 8.12 | 1.17 | 85.60 | 0.00000000 | annexin A13 |
| Slco1a1 | 208.21 | 30.70 | 85.26 | 0.00000000 | solute carrier organic anion transporter family, member 1a1 |
| Manf | 52.95 | 7.81 | 85.24 | 0.00000000 | mesencephalic astrocyte-derived neurotrophic factor |
| Slc22a28 | 32.21 | 4.83 | 84.99 | 0.00000000 | solute carrier family 22, member 28 |
| Serpinh1 | 8.62 | 1.37 | 84.05 | 0.00000000 | serine (or cysteine) peptidase inhibitor, clade H, member 1 |
| Mdk | 16.92 | 2.72 | 83.91 | 0.00000000 | midkine |
| Ces2b | 5.00 | 0.82 | 83.66 | 0.00000000 | Carboxyesterase 2B |
| Hspa5 | 500.40 | 82.59 | 83.50 | 0.00000000 | heat shock protein 5 |
| Odc1 | 14.72 | 2.45 | 83.34 | 0.00000000 | Ornithine decarboxylase, structural 1 |
| Gm853 | 14.88 | 2.50 | 83.21 | 0.00000000 | predicted gene 853 |
| Cndp2 | 567.62 | 99.93 | 82.39 | 0.00000000 | CNDP dipeptidase 2 (metallopeptidase M20 family) |
| C1qtnf3 | 196.68 | 34.72 | 82.35 | 0.00000000 | C1q and tumor necrosis factor related protein 3 |
| Cyp2j13 | 101.54 | 18.27 | 82.01 | 0.00000000 | cytochrome P450, family 2, subfamily j, polypeptide 13 |
| Rtp3 | 2.46 | 0.44 | 81.91 | 0.00000001 | receptor transporter protein 3 |
| Hspb1 | 2.47 | 0.45 | 81.90 | 0.00057488 | heat shock protein 1 |
| Slc22a30 | 195.67 | 35.43 | 81.89 | 0.00000000 | solute carrier family 22, member 30 |
| Gm13498 | 5.80 | 1.10 | 81.05 | 0.00000000 | predicted gene 13498 |
| C4a | 1.55 | 0.30 | 80.84 | 0.00000000 | Complement component 4A (Rodgers blood group) |
| Apoa1 | 2.49 | 0.48 | 80.55 | 0.00100158 | apolipoprotein A-I |
| Cyp24a1 | 12.72 | 2.49 | 80.45 | 0.00000000 | cytochrome P450, family 24, subfamily a, polypeptide 1 |
| Rpl3l | 1.78 | 0.35 | 80.14 | 0.00191032 | ribosomal protein L3-like |
| Lpl | 180.65 | 37.27 | 79.37 | 0.00000000 | lipoprotein lipase; similar to Lipoprotein lipase precursor (LPL) |
| Gm2016 | 1.32 | 0.28 | 78.85 | 0.00039848 | predicted gene 2016 |
| Chordc1 | 50.01 | 10.64 | 78.72 | 0.00000000 | cysteine and histidine-rich domain (CHORD)-containing, zinc-binding protein 1 |
| B4galt5 | 5.92 | 1.27 | 78.52 | 0.00000000 | UDP-Gal:betaGlcNAc beta 1,4-galactosyltransferase, polypeptide 5 |
| Cpe | 15.64 | 3.41 | 78.21 | 0.00000000 | carboxypeptidase E; similar to carboxypeptidase E |
| Hsph1 | 21.69 | 4.75 | 78.12 | 0.00000000 | heat shock 105kDa/110kDa protein 1 |
| Hsd17b13 | 2.95 | 0.65 | 77.92 | 0.00204276 | hydroxysteroid (17-beta) dehydrogenase 13 |
| Hsp90ab1 | 775.83 | 172.91 | 77.71 | 0.00000367 | heat shock protein 90 alpha (cytosolic), class B member 1 |
| Gm5662 | 3.05 | 0.69 | 77.49 | 0.00000041 | predicted gene, EG435337 |
| Ldhd | 206.41 | 46.61 | 77.42 | 0.00000000 | lactate dehydrogenase D |
| Slc9a8 | 22.70 | 5.17 | 77.20 | 0.00000000 | solute carrier family 9 (sodium/hydrogen exchanger), member 8 |
| Ceacam2 | 8.15 | 1.91 | 76.58 | 0.00000004 | carcinoembryonic antigen-related cell adhesion molecule 2 |
| Gusb | 16.22 | 3.84 | 76.31 | 0.00000000 | glucuronidase, beta |
| Hsp90aa1 | 35.94 | 8.62 | 76.03 | 0.00000000 | Heat shock protein 90, alpha (cytosolic), class A member 1 |
| Sec14l3 | 3.18 | 0.77 | 75.76 | 0.00000000 | SEC14-like 3 (S. cerevisiae) |
| Hspa8 | 399.77 | 97.00 | 75.74 | 0.00000000 | similar to heat shock protein 8; heat shock protein 8 |
| Tmem169 | 1.94 | 0.47 | 75.73 | 0.00000070 | transmembrane protein 169 |
| Dnajb1 | 16.82 | 4.12 | 75.53 | 0.00000000 | DnaJ (Hsp40) homolog, subfamily B, member 1 |
| Rhox6 | 2.83 | 0.70 | 75.37 | 0.00126947 | reproductive homeobox 6 |
| Znrf1 | 14.94 | 3.72 | 75.11 | 0.00000000 | zinc and ring finger 1 |
| 4930502E18Rik | 3.64 | 0.92 | 74.71 | 0.00001840 | RIKEN cDNA 4930502E18 gene |
| Adh1 | 1796.96 | 459.39 | 74.44 | 0.00018104 | alcohol dehydrogenase 1 (class I) |
| Trpv4 | 21.87 | 5.63 | 74.28 | 0.00000000 | transient receptor potential cation channel, subfamily V, member 4 |
| Gm6614 | 3.91 | 1.02 | 74.00 | 0.00000005 | predicted gene 6614 |
| Apoh | 9.73 | 2.55 | 73.80 | 0.00000000 | apolipoprotein H |
| P4ha1 | 9.13 | 2.41 | 73.62 | 0.00000000 | procollagen-proline, 2-oxoglutarate 4-dioxygenase (proline 4-hydroxylase), alpha 1 polypeptide |
| Pcyox1l | 1.06 | 0.28 | 73.39 | 0.00185330 | prenylcysteine oxidase 1 like |
| Slc6a15 | 7.69 | 2.05 | 73.34 | 0.00000000 | solute carrier family 6 (neurotransmitter transporter), member 15 |
| Hsd17b11 | 36.81 | 9.87 | 73.19 | 0.00000000 | hydroxysteroid (17-beta) dehydrogenase 11 |
| Hist1h4i | 11.65 | 3.14 | 73.09 | 0.00001440 | Histone cluster 1, H4i |
| Fmo5 | 75.66 | 20.57 | 72.82 | 0.00000000 | flavin containing monooxygenase 5 |
| Cyp4a12a | 230.09 | 63.36 | 72.46 | 0.00000000 | cytochrome P450, family 4, subfamily a, polypeptide 12a |
| BC021614 | 26.79 | 7.49 | 72.03 | 0.00000000 | cDNA sequence BC021614 |
| Mpv17l | 108.97 | 30.88 | 71.66 | 0.00000000 | Mpv17 transgene, kidney disease mutant-like |
| Apoa4 | 1.81 | 0.51 | 71.63 | 0.00046883 | apolipoprotein A-IV |
| Nlrc4 | 0.96 | 0.27 | 71.61 | 0.00008310 | NLR family, CARD domain containing 4 |
| Ace | 5.90 | 1.69 | 71.32 | 0.00000041 | angiotensin I converting enzyme (peptidyl-dipeptidase A) 1 |
| Atp11a | 40.61 | 11.87 | 70.77 | 0.00000000 | ATPase, class VI, type 11A |
| Tbx10 | 8.78 | 2.58 | 70.57 | 0.00000055 | T-box 10 |
| Irf5 | 2.55 | 0.75 | 70.52 | 0.00001170 | interferon regulatory factor 5 |
| 5730528L13Rik | 2.01 | 0.59 | 70.50 | 0.00066861 | RIKEN cDNA 5730528L13 gene |
| Ly6a | 1128.91 | 334.82 | 70.34 | 0.00000172 | lymphocyte antigen 6 complex, locus A |
| Banp | 4.72 | 1.40 | 70.34 | 0.00000000 | BTG3 associated nuclear protein |
| Ly6e | 152.50 | 45.45 | 70.20 | 0.00000000 | lymphocyte antigen 6 complex, locus E |
| Aacs | 115.32 | 34.92 | 69.72 | 0.00000000 | acetoacetyl-CoA synthetase |
| Slco1a6 | 618.35 | 187.66 | 69.65 | 0.00021190 | solute carrier organic anion transporter family, member 1a6 |
| Dnaja1 | 61.99 | 18.82 | 69.64 | 0.00000552 | DnaJ (Hsp40) homolog, subfamily A, member 1 |
| Hyou1 | 3.61 | 1.10 | 69.49 | 0.00000000 | hypoxia up-regulated 1 |
| Vill | 15.92 | 4.86 | 69.49 | 0.00000000 | villin-like |
| Car4 | 347.15 | 105.99 | 69.47 | 0.00000001 | carbonic anhydrase 4 |
| Inmt | 900.50 | 276.06 | 69.34 | 0.00000425 | indolethylamine N-methyltransferase |
| Ahsa1 | 50.48 | 15.47 | 69.34 | 0.00000000 | AHA1, activator of heat shock protein ATPase homolog 1 (yeast) |
| Myo5a | 3.13 | 0.96 | 69.30 | 0.00000000 | myosin VA |
| Ahsa2 | 7.01 | 2.16 | 69.17 | 0.00000173 | AHA1, activator of heat shock protein ATPase homolog 2 (yeast) |
| Als2cr4 | 48.89 | 15.17 | 68.98 | 0.00000000 | amyotrophic lateral sclerosis 2 (juvenile) chromosome region, candidate 4 |
| Sh3tc1 | 1.94 | 0.61 | 68.76 | 0.00000020 | SH3 domain and tetratricopeptide repeats 1 |
| Slco1a5 | 50.45 | 15.84 | 68.59 | 0.00000000 | solute carrier organic anion transporter family, member 1a5 |
| Amacr | 151.87 | 48.09 | 68.34 | 0.00000004 | alpha-methylacyl-CoA racemase |
| 9030425E11Rik | 1.00 | 0.32 | 67.96 | 0.00097106 | RIKEN cDNA 9030425E11 gene |
| Slc17a3 | 218.46 | 70.62 | 67.67 | 0.00008770 | solute carrier family 17 (sodium phosphate), member 3 |
| Hsf1 | 3.49 | 1.15 | 67.14 | 0.00000448 | heat shock factor 1 |
| Stip1 | 59.67 | 19.76 | 66.88 | 0.00000000 | stress-induced phosphoprotein 1 |
| Ncoa5 | 3.23 | 1.07 | 66.82 | 0.00000023 | nuclear receptor coactivator 5 |
| Cyp4a12b | 145.31 | 48.27 | 66.78 | 0.00000001 | cytochrome P450, family 4, subfamily a, polypeptide 12B |
| Lrp12 | 1.75 | 0.58 | 66.75 | 0.00000363 | low density lipoprotein-related protein 12 |
| Ces1f | 584.77 | 194.58 | 66.73 | 0.00006810 | Carboxylesterase 1F |
| Erc2 | 2.78 | 0.93 | 66.59 | 0.00000000 | ELKS/RAB6-interacting/CAST family member 2 |
| Car9 | 2.57 | 0.86 | 66.43 | 0.00008290 | carbonic anhydrase 9 |
| Tmem45b | 69.08 | 23.31 | 66.25 | 0.00000000 | transmembrane protein 45b |
| Rlf | 1.66 | 0.56 | 65.98 | 0.00000014 | rearranged L-myc fusion sequence |
| Hsd11b1 | 102.39 | 34.97 | 65.84 | 0.00000002 | hydroxysteroid 11-beta dehydrogenase 1 |
| Nipal2 | 4.61 | 1.58 | 65.81 | 0.00000000 | NIPA-like domain containing 2 |
| Rhpn1 | 1.65 | 0.57 | 65.76 | 0.00061725 | rhophilin, Rho GTPase binding protein 1 |
| Osta | 151.83 | 52.13 | 65.66 | 0.00000001 | organic solute transporter alpha |
| Il13ra1 | 9.82 | 3.37 | 65.65 | 0.00000000 | interleukin 13 receptor, alpha 1 |
| Kif12 | 10.69 | 3.67 | 65.63 | 0.00000000 | kinesin family member 12 |
| Sel1l | 8.27 | 2.85 | 65.59 | 0.00000000 | sel-1 suppressor of lin-12-like (C. elegans) |
| Slc16a14 | 12.52 | 4.32 | 65.46 | 0.00000000 | solute carrier family 16 (monocarboxylic acid transporters), member 14 |
| Cckar | 14.14 | 4.92 | 65.21 | 0.00000000 | cholecystokinin A receptor |
| Pi4ka | 14.96 | 5.21 | 65.15 | 0.00000001 | phosphatidylinositol 4-kinase, catalytic, alpha polypeptide |
| Me1 | 33.99 | 11.89 | 65.01 | 0.00000000 | Malic enzyme 1, NADP(+)-dependent, cytosolic |
| Hmgcr | 6.43 | 2.25 | 65.01 | 0.00000000 | 3-hydroxy-3-methylglutaryl-Coenzyme A reductase |
| Cmtm6 | 25.08 | 8.82 | 64.82 | 0.00000000 | CKLF-like MARVEL transmembrane domain containing 6 |
| Gm6788 | 146.11 | 51.60 | 64.68 | 0.00000000 | predicted gene 6788 |
| Gm5069 | 1.39 | 0.49 | 64.54 | 0.00018614 | predicted gene 5069 |
| Dynll1 | 70.93 | 25.25 | 64.40 | 0.00000000 | Dynein light chain LC8-type 1 |
| Hsp90b1 | 228.87 | 82.07 | 64.14 | 0.00000700 | heat shock protein 90, beta (Grp94), member 1 |
| Itpk1 | 1.49 | 0.54 | 64.04 | 0.00042138 | inositol 1,3,4-triphosphate 5/6 kinase |
| Tmco3 | 27.88 | 10.05 | 63.96 | 0.00000001 | transmembrane and coiled-coil domains 3 |
| Ifrd2 | 27.54 | 9.97 | 63.79 | 0.00000000 | interferon-related developmental regulator 2 |
| Tlcd2 | 5.89 | 2.15 | 63.44 | 0.00006620 | TLC domain containing 2 |
| Akr1c14 | 182.24 | 66.76 | 63.37 | 0.00000295 | aldo-keto reductase family 1, member C14 |
| Tnfrsf12a | 25.55 | 9.38 | 63.30 | 0.00112551 | tumor necrosis factor receptor superfamily, member 12a |
| Pdia6 | 200.85 | 73.83 | 63.24 | 0.00000240 | Protein disulfide isomerase associated 6 |
| Aifm3 | 1.78 | 0.66 | 62.83 | 0.00031932 | apoptosis-inducing factor, mitochondrion-associated 3 |
| Sec24d | 4.07 | 1.53 | 62.43 | 0.00000006 | Sec24 related gene family, member D (S. cerevisiae) |
| Hmox1 | 72.03 | 27.10 | 62.38 | 0.00002480 | heme oxygenase (decycling) 1 |
| Calr | 415.41 | 156.60 | 62.30 | 0.00008050 | calreticulin |
| Gm12824 | 3.64 | 1.37 | 62.23 | 0.00000000 | predicted gene 12824 |
| Esr2 | 1.55 | 0.59 | 62.18 | 0.00028786 | estrogen receptor 2 (beta) |
| Abhd2 | 1.96 | 0.74 | 62.13 | 0.00013098 | abhydrolase domain containing 2 |
| Frzb | 7.83 | 2.97 | 62.05 | 0.00000001 | frizzled-related protein |
| Xbp1 | 17.32 | 6.59 | 61.97 | 0.00000000 | X-box binding protein 1 |
| Vwa2 | 1.05 | 0.40 | 61.94 | 0.00076096 | von Willebrand factor A domain containing 2 |
| Fam132a | 7.35 | 2.80 | 61.94 | 0.00000714 | family with sequence similarity 132, member A |
| Cacybp | 34.88 | 13.39 | 61.60 | 0.00000000 | calcyclin binding protein |
| Dnajb11 | 13.11 | 5.08 | 61.24 | 0.00000001 | DnaJ (Hsp40) homolog, subfamily B, member 11 |
| Serpinf2 | 326.56 | 127.20 | 61.05 | 0.00009820 | serine (or cysteine) peptidase inhibitor, clade F, member 2 |
| Sh3bp2 | 5.36 | 2.11 | 60.71 | 0.00000083 | SH3-domain binding protein 2 |
| Grtp1 | 29.88 | 11.76 | 60.64 | 0.00000000 | GH regulated TBC protein 1 |
| Gas2 | 262.91 | 103.57 | 60.61 | 0.00004530 | growth arrest specific 2 |
| Cd55 | 4.61 | 1.82 | 60.57 | 0.00000267 | CD55 antigen |
| Defb29 | 149.45 | 58.97 | 60.54 | 0.00000000 | defensin beta 29 |
| Tradd | 2.86 | 1.13 | 60.54 | 0.00193080 | TNFRSF1A-associated via death domain |
| Cd36 | 276.82 | 109.93 | 60.29 | 0.00077350 | CD36 antigen |
| Gjb2 | 20.17 | 8.02 | 60.26 | 0.00000000 | gap junction protein, beta 2 |
| Ptpru | 1.04 | 0.41 | 60.25 | 0.00025158 | protein tyrosine phosphatase, receptor type, U |
| Entpd4 | 7.16 | 2.85 | 60.16 | 0.00000004 | ectonucleoside triphosphate diphosphohydrolase 4 |
| Pdia3 | 166.28 | 66.26 | 60.15 | 0.00000784 | protein disulfide isomerase associated 3 |
| Dusp6 | 18.22 | 7.29 | 60.02 | 0.00000000 | dual specificity phosphatase 6 |
| Cidea | 5.33 | 2.13 | 60.00 | 0.00195807 | cell death-inducing DNA fragmentation factor, alpha subunit-like effector A |
| Ncln | 2.58 | 1.03 | 60.00 | 0.00006030 | nicalin homolog (zebrafish) |
| Reep5 | 33.24 | 13.36 | 59.80 | 0.00000001 | receptor accessory protein 5 |
| Acss3 | 2.26 | 0.91 | 59.74 | 0.00078458 | acyl-CoA synthetase short-chain family member 3 |
| Npdc1 | 6.06 | 2.44 | 59.64 | 0.00003180 | neural proliferation, differentiation and control gene 1 |
| Cyp7b1 | 53.59 | 21.69 | 59.52 | 0.00000004 | cytochrome P450, family 7, subfamily b, polypeptide 1 |
| Arhgef16 | 7.63 | 3.09 | 59.48 | 0.00000008 | Rho guanine nucleotide exchange factor (GEF) 16 |
| Ppic | 77.08 | 31.24 | 59.48 | 0.00000000 | peptidylprolyl isomerase C |
| Cml2 | 20.22 | 8.22 | 59.36 | 0.00000001 | camello-like 2 |
| Aspdh | 174.20 | 70.81 | 59.35 | 0.00000004 | aspartate dehydrogenase domain containing |
| Csf1r | 3.73 | 1.52 | 59.11 | 0.00000100 | colony stimulating factor 1 receptor |
| Hspa4l | 4.69 | 1.93 | 58.86 | 0.00000042 | heat shock protein 4 like |
| Tmem176a | 1175.17 | 483.45 | 58.86 | 0.00188075 | transmembrane protein 176A |
| Slc33a1 | 15.64 | 6.45 | 58.77 | 0.00000001 | solute carrier family 33 (acetyl-CoA transporter), member 1 |
| Hnrnpm | 8.97 | 3.76 | 58.12 | 0.00005260 | heterogeneous nuclear ribonucleoprotein M |
| Ubxn10 | 2.02 | 0.85 | 58.10 | 0.00043971 | UBX domain protein 10 |
| Por | 72.87 | 30.57 | 58.05 | 0.00000040 | P450 (cytochrome) oxidoreductase |
| Pdia4 | 123.06 | 51.66 | 58.02 | 0.00000413 | protein disulfide isomerase associated 4 |
| Pzp | 34.71 | 14.64 | 57.82 | 0.00000054 | pregnancy zone protein |
| Prr5 | 12.64 | 5.35 | 57.66 | 0.00000032 | Rho GTPase activating protein 8; proline rich 5 (renal), Arhgap8 |
| Met | 2.40 | 1.02 | 57.61 | 0.00000133 | met proto-oncogene |
| Farsb | 43.84 | 18.59 | 57.59 | 0.00000004 | phenylalanyl-tRNA synthetase, beta subunit |
| Bcl7b | 3.79 | 1.61 | 57.42 | 0.00047528 | B-cell CLL/lymphoma 7B |
| Agps | 7.83 | 3.34 | 57.35 | 0.00000004 | alkylglycerone phosphate synthase |
| Bdh1 | 25.08 | 10.76 | 57.12 | 0.00000398 | 3-hydroxybutyrate dehydrogenase, type 1 |
| Slc25a19 | 40.52 | 17.40 | 57.06 | 0.00000003 | solute carrier family 25 (mitochondrial thiamine pyrophosphate carrier), member 19 |
| Ywhag | 4.10 | 1.77 | 56.87 | 0.00000344 | tyrosine 3-monooxygenase/tryptophan 5-monooxygenase activation protein, gamma polypeptide |
| Kcnk5 | 26.22 | 11.34 | 56.77 | 0.00000016 | potassium channel, subfamily K, member 5 |
| Evc | 4.92 | 2.14 | 56.47 | 0.00000049 | Ellis van Creveld gene homolog (human) |
| 0610012H03Rik | 333.34 | 145.24 | 56.43 | 0.00009370 | RIKEN cDNA 0610012H03 gene |
| Etv1 | 3.88 | 1.69 | 56.37 | 0.00221532 | ets variant gene 1 |
| Tfrc | 5.68 | 2.49 | 56.13 | 0.00000087 | transferrin receptor |
| Ces2c | 58.77 | 25.80 | 56.10 | 0.00000033 | Carboxylesterase 2C |
| Erp29 | 72.41 | 31.79 | 56.10 | 0.00000010 | endoplasmic reticulum protein 29 |
| Mlec | 34.64 | 15.24 | 56.01 | 0.00000272 | malectin |
| Pmepa1 | 1.47 | 0.65 | 55.70 | 0.00043079 | prostate transmembrane protein, androgen induced 1 |
| Klc4 | 5.72 | 2.54 | 55.67 | 0.00001390 | kinesin light chain 4 |
| Ntn1 | 1.37 | 0.61 | 55.37 | 0.00020129 | similar to Netrin-1 precursor; netrin 1 |
| Actg1 | 56.10 | 25.06 | 55.34 | 0.00000191 | Actin, gamma, cytoplasmic 1 |
| Zfp937 | 1.25 | 0.56 | 55.33 | 0.00014407 | Zinc finger protein 937 |
| Pion | 5.34 | 2.39 | 55.26 | 0.00000276 | pigeon homolog (Drosophila) |
| Proc | 222.02 | 99.41 | 55.23 | 0.00022434 | protein C |
| Chtf8 | 1.78 | 0.80 | 55.20 | 0.00176636 | CTF8, chromosome transmission fidelity factor 8 |
| Nt5e | 31.19 | 13.98 | 55.18 | 0.00000089 | 5' nucleotidase, ecto |
| Col6a6 | 1.15 | 0.52 | 55.03 | 0.00027342 | RIKEN cDNA E330026B02 gene |
| Glyctk | 11.34 | 5.11 | 54.95 | 0.00000738 | glycerate kinase |
| Bsnd | 4.80 | 2.17 | 54.82 | 0.00002080 | Bartter syndrome, infantile, with sensorineural deafness (Barttin) |
| Lmbrd2 | 2.28 | 1.03 | 54.77 | 0.00051547 | LMBR1 domain containing 2 |
| Timd2 | 15.48 | 7.01 | 54.75 | 0.00001310 | T-cell immunoglobulin and mucin domain containing 2 |
| St8sia1 | 5.01 | 2.28 | 54.58 | 0.00000033 | ST8 alpha-N-acetyl-neuraminide alpha-2,8-sialyltransferase 1 |
| Erp44 | 21.95 | 9.98 | 54.53 | 0.00000022 | endoplasmic reticulum protein 44 |
| Pecr | 669.43 | 304.83 | 54.46 | 0.00091072 | peroxisomal trans-2-enoyl-CoA reductase |
| Fkbp4 | 46.62 | 21.23 | 54.46 | 0.00000047 | FK506 binding protein 4 |
| Psen1 | 35.59 | 16.21 | 54.45 | 0.00000096 | presenilin 1 |
| Mettl7b | 393.70 | 179.74 | 54.35 | 0.00018602 | methyltransferase like 7B |
| 0610011F06Rik | 395.65 | 181.35 | 54.16 | 0.00001920 | RIKEN cDNA 0610011F06 gene |
| Cish | 12.76 | 5.86 | 54.10 | 0.00005760 | cytokine inducible SH2-containing protein |
| Ubiad1 | 15.94 | 7.32 | 54.07 | 0.00000075 | UbiA prenyltransferase domain containing 1 |
| Dnajc22 | 60.28 | 27.71 | 54.03 | 0.00000096 | DnaJ (Hsp40) homolog, subfamily C, member 22 |
| Cdk2ap2 | 25.37 | 11.67 | 54.00 | 0.00000453 | CDK2-associated protein 2 |
| Mif4gd | 30.22 | 13.92 | 53.95 | 0.00000144 | MIF4G domain containing |
| Atp9a | 4.71 | 2.17 | 53.85 | 0.00000860 | ATPase, class II, type 9A |
| Rab11fip3 | 38.27 | 17.73 | 53.67 | 0.00000264 | RAB11 family interacting protein 3 (class II) |
| Susd2 | 29.76 | 13.80 | 53.61 | 0.00000207 | sushi domain containing 2 |
| Rnf145 | 7.40 | 3.45 | 53.34 | 0.00000307 | ring finger protein 145 |
| Mvd | 8.53 | 3.98 | 53.31 | 0.00002410 | mevalonate (diphospho) decarboxylase |
| Tpt1 | 15.42 | 7.20 | 53.31 | 0.00007550 | Tumor protein, translationally-controlled 1 |
| Tra2a | 3.30 | 1.55 | 53.09 | 0.00186667 | transformer 2 alpha homolog (Drosophila) |
| Gcgr | 4.30 | 2.02 | 52.98 | 0.00049780 | glucagon receptor |
| Hspe1 | 301.96 | 142.00 | 52.97 | 0.00000918 | Heat shock protein 1 (chaperonin 10) |
| Aspg | 5.02 | 2.37 | 52.78 | 0.00004840 | asparaginase homolog (S. cerevisiae) |
| Derl1 | 12.76 | 6.03 | 52.75 | 0.00000097 | Der1-like domain family, member 1; predicted gene 6737 |
| Aup1 | 89.72 | 42.42 | 52.72 | 0.00000358 | ancient ubiquitous protein 1 |
| Cpsf6 | 2.85 | 1.35 | 52.72 | 0.00000860 | cleavage and polyadenylation specific factor 6 |
| Slc39a11 | 7.15 | 3.38 | 52.72 | 0.00010018 | Solute carrier family 39 (metal ion transporter), member 11 |
| Egf | 113.69 | 53.76 | 52.71 | 0.00066596 | epidermal growth factor |
| Gmppb | 8.98 | 4.25 | 52.70 | 0.00050743 | GDP-mannose pyrophosphorylase B |
| Pdk3 | 14.43 | 6.83 | 52.66 | 0.00000239 | pyruvate dehydrogenase kinase, isoenzyme 3 |
| Amdhd2 | 28.84 | 13.65 | 52.66 | 0.00000130 | amidohydrolase domain containing 2 |
| Gm7092 | 58.69 | 27.81 | 52.63 | 0.00000124 | predicted gene 7092 |
| Csrp2 | 179.84 | 85.20 | 52.62 | 0.00000427 | cysteine and glycine-rich protein 2 |
| Scrn2 | 12.34 | 5.88 | 52.39 | 0.00001650 | secernin 2 |
| Rbp1 | 3.75 | 1.79 | 52.37 | 0.00026632 | retinol binding protein 1, cellular |
| Lztr1 | 22.32 | 10.67 | 52.21 | 0.00000153 | leucine-zipper-like transcriptional regulator, 1 |
| Galntl4 | 5.99 | 2.86 | 52.16 | 0.00003380 | UDP-N-acetyl-alpha-D-galactosamine:polypeptide N-acetylgalactosaminyltransferase-like 4 |
| Pdzd3 | 40.81 | 19.54 | 52.11 | 0.00000208 | PDZ domain containing 3 |
| Cyp51 | 65.15 | 31.22 | 52.08 | 0.00008450 | cytochrome P450, family 51 |
| Tuba1c | 16.52 | 7.97 | 51.75 | 0.00000419 | tubulin, alpha 1C; predicted gene 6682 |
| Grwd1 | 4.85 | 2.35 | 51.59 | 0.00047423 | glutamate-rich WD repeat containing 1 |
| Kif20b | 2.99 | 1.46 | 51.15 | 0.00007460 | kinesin family member 20B |
| Rce1 | 11.51 | 5.63 | 51.13 | 0.00004980 | RCE1 homolog, prenyl protein peptidase (S. cerevisiae) |
| Osgin1 | 88.72 | 43.45 | 51.02 | 0.00004510 | oxidative stress induced growth inhibitor 1 |
| Afmid | 70.46 | 34.55 | 50.96 | 0.00002980 | arylformamidase |
| Sypl2 | 1.94 | 0.95 | 50.95 | 0.00230530 | synaptophysin-like 2 |
| Sipa1l1 | 3.68 | 1.81 | 50.89 | 0.00003880 | signal-induced proliferation-associated 1 like 1 |
| Rbm14 | 2.33 | 1.14 | 50.88 | 0.00221120 | RNA binding motif protein 14 |
| Eme2 | 4.99 | 2.47 | 50.48 | 0.00166455 | essential meiotic endonuclease 1 homolog 2 (S. pombe) |
| Hpn | 138.02 | 68.44 | 50.42 | 0.00054415 | hepsin |
| Idi1 | 3.36 | 1.67 | 50.32 | 0.00062862 | isopentenyl-diphosphate delta isomerase; similar to Isopentenyl-diphosphate delta isomerase; predicted gene 7655 |
| Tst | 136.70 | 68.02 | 50.24 | 0.00003820 | thiosulfate sulfurtransferase, mitochondrial |
| Ddo | 18.13 | 9.02 | 50.24 | 0.00000461 | D-aspartate oxidase |
| Hspd1 | 263.59 | 131.36 | 50.16 | 0.00181563 | predicted gene 12141; heat shock protein 1 (chaperonin) |
| Slc9a3r1 | 25.59 | 12.76 | 50.15 | 0.00000563 | solute carrier family 9 (sodium/hydrogen exchanger), member 3 regulator 1 |
| Slc22a12 | 10.14 | 5.06 | 50.14 | 0.00002120 | solute carrier family 22 (organic anion/cation transporter), member 12 |
| Hist1h2bc | 126.12 | 62.90 | 50.13 | 0.00000549 | histone cluster 1, H2bg; histone cluster 1, H2be; histone cluster 2, H2bb; histone cluster 1, H2bc |
| Slc46a3 | 8.75 | 4.37 | 50.08 | 0.00003480 | solute carrier family 46, member 3 |
| Igj | 3.69 | 1.84 | 50.06 | 0.00195283 | immunoglobulin joining chain |
| Vps8 | 5.32 | 2.66 | 50.04 | 0.00001780 | vacuolar protein sorting 8 homolog (S. cerevisiae) |
| Degs2 | 169.20 | 84.75 | 49.91 | 0.00013140 | degenerative spermatocyte homolog 2 (Drosophila), lipid desaturase |
| Irf6 | 5.43 | 2.72 | 49.89 | 0.00002080 | interferon regulatory factor 6 |
| 2300009A05Rik | 25.13 | 12.60 | 49.86 | 0.00035802 | RIKEN cDNA 2300009A05 gene |
| Hnrnpc | 23.66 | 11.88 | 49.79 | 0.00002520 | heterogeneous nuclear ribonucleoprotein C |
| P4hb | 160.10 | 80.59 | 49.66 | 0.00065814 | prolyl 4-hydroxylase, beta polypeptide |
| Ostc | 51.31 | 25.87 | 49.58 | 0.00000806 | oligosaccharyltransferase complex subunit |
| Akap2 | 5.74 | 2.90 | 49.54 | 0.00003770 | A kinase (PRKA) anchor protein 2; paralemmin 2 |
| Rpn1 | 20.74 | 10.49 | 49.42 | 0.00001020 | ribophorin I |
| St13 | 79.49 | 40.26 | 49.35 | 0.00002280 | suppression of tumorigenicity 13 |
| Zfp810 | 10.22 | 5.18 | 49.32 | 0.00001440 | zinc finger protein 810 |
| Necap2 | 7.36 | 3.75 | 49.04 | 0.00022542 | NECAP endocytosis associated 2 |
| Gdi2 | 198.81 | 101.35 | 49.02 | 0.00191274 | guanosine diphosphate (GDP) dissociation inhibitor 2 |
| Spns1 | 7.72 | 3.95 | 48.83 | 0.00004910 | spinster homolog 1 (Drosophila) |
| P4ha2 | 16.40 | 8.40 | 48.79 | 0.00073545 | procollagen-proline, 2-oxoglutarate 4-dioxygenase (proline 4-hydroxylase), alpha II polypeptide |
| Pla2g6 | 10.87 | 5.57 | 48.75 | 0.00209839 | phospholipase A2, group VI |
| Leo1 | 3.62 | 1.86 | 48.63 | 0.00204278 | Leo1, Paf1/RNA polymerase II complex component, homolog (S. cerevisiae) |
| Arhgap15 | 3.11 | 1.60 | 48.48 | 0.00213100 | Rho GTPase activating protein 15 |
| Itih2 | 3.54 | 1.83 | 48.45 | 0.00064678 | inter-alpha trypsin inhibitor, heavy chain 2 |
| Rgl1 | 14.45 | 7.45 | 48.42 | 0.00002040 | ral guanine nucleotide dissociation stimulator,-like 1 |
| Papss1 | 68.33 | 35.27 | 48.38 | 0.00017407 | 3'-phosphoadenosine 5'-phosphosulfate synthase 1 |
| Zbtb20 | 13.79 | 7.12 | 48.35 | 0.00036574 | zinc finger and BTB domain containing 20 |
| Pllp | 7.60 | 3.92 | 48.35 | 0.00027239 | plasma membrane proteolipid |
| Cbs | 68.75 | 35.65 | 48.15 | 0.00035846 | cystathionine beta-synthase |
| Snx31 | 11.86 | 6.15 | 48.11 | 0.00003670 | sorting nexin 31 |
| Dnajc3 | 12.03 | 6.25 | 48.05 | 0.00001650 | DnaJ (Hsp40) homolog, subfamily C, member 3 |
| Slco3a1 | 13.00 | 6.78 | 47.83 | 0.00082514 | solute carrier organic anion transporter family, member 3a1 |
| Ass1 | 239.02 | 124.77 | 47.80 | 0.00101496 | argininosuccinate synthetase 1 |
| Rdh10 | 3.95 | 2.07 | 47.73 | 0.00164695 | retinol dehydrogenase 10 (all-trans) |
| Tmtc4 | 5.08 | 2.66 | 47.63 | 0.00014672 | transmembrane and tetratricopeptide repeat containing 4 |
| Cyb5r3 | 58.05 | 30.40 | 47.63 | 0.00004830 | cytochrome b5 reductase 3 |
| Rhoc | 31.13 | 16.31 | 47.59 | 0.00003620 | ras homolog gene family, member C |
| Usp10 | 26.20 | 13.79 | 47.38 | 0.00003280 | ubiquitin specific peptidase 10 |
| Far1 | 58.64 | 30.87 | 47.36 | 0.00072935 | fatty acyl CoA reductase 1 |
| Rab38 | 8.36 | 4.40 | 47.35 | 0.00058951 | RAB38, member of RAS oncogene family |
| Ascc2 | 7.33 | 3.87 | 47.22 | 0.00013395 | activating signal cointegrator 1 complex subunit 2 |
| Lasp1 | 4.04 | 2.13 | 47.20 | 0.00038189 | LIM and SH3 protein 1 |
| 2810407C02Rik | 16.57 | 8.75 | 47.18 | 0.00002730 | RIKEN cDNA 2810407C02 gene |
| Tcfec | 16.15 | 8.54 | 47.10 | 0.00005530 | transcription factor EC |
| Ddost | 23.83 | 12.61 | 47.09 | 0.00003170 | dolichyl-di-phosphooligosaccharide-protein glycotransferase |
| Atf4 | 20.69 | 10.97 | 46.98 | 0.00003620 | activating transcription factor 4 |
| Mfap3l | 4.63 | 2.45 | 46.94 | 0.00104230 | microfibrillar-associated protein 3-like |
| E030010A14Rik | 93.37 | 49.54 | 46.94 | 0.00054888 | RIKEN cDNA E030010A14 gene |
| Gm15421 | 35.42 | 18.84 | 46.80 | 0.00191339 | Ribosomal protein L22 like 1 pseudogene |
| Gm6900 | 21.84 | 11.64 | 46.70 | 0.00084641 | predicted gene 6900 (Gm6900) pseudogene on chromosome 7 |
| Fcamr | 6.97 | 3.71 | 46.69 | 0.00075233 | Fc receptor, IgA, IgM, high affinity |
| 2610019F03Rik | 5.28 | 2.82 | 46.66 | 0.00064376 | RIKEN cDNA 2610019F03 gene |
| Lman2l | 6.94 | 3.71 | 46.64 | 0.00035284 | lectin, mannose-binding 2-like |
| Chrna4 | 4.29 | 2.29 | 46.57 | 0.00018262 | cholinergic receptor, nicotinic, alpha polypeptide 4 |
| Dhdds | 6.50 | 3.48 | 46.49 | 0.00014553 | dehydrodolichyl diphosphate synthase |
| 1300002K09Rik | 2.43 | 1.30 | 46.40 | 0.00241997 | RIKEN cDNA 1300002K09 gene |
| BC026585 | 330.32 | 177.05 | 46.40 | 0.00182542 | cDNA sequence BC026585 |
| Arf1 | 169.99 | 91.20 | 46.35 | 0.00133725 | predicted gene 5823; ADP-ribosylation factor 1; predicted gene 8230 |
| Tmem30a | 17.06 | 9.17 | 46.27 | 0.00005790 | transmembrane protein 30A |
| Morf4l2 | 34.53 | 18.56 | 46.27 | 0.00024695 | Mortality factor 4 like 2 |
| Pxn | 2.72 | 1.46 | 46.21 | 0.00169566 | paxillin |
| Tmem109 | 43.51 | 23.41 | 46.19 | 0.00006260 | transmembrane protein 109 |
| Trps1 | 4.43 | 2.39 | 46.07 | 0.00004940 | trichorhinophalangeal syndrome I (human); similar to Trps1 protein |
| Cyb561 | 11.13 | 6.02 | 45.96 | 0.00009410 | cytochrome b-561 |
| Rnf24 | 7.44 | 4.02 | 45.95 | 0.00037692 | ring finger protein 24 |
| Txnl1 | 10.82 | 5.85 | 45.89 | 0.00009530 | thioredoxin-like 1 |
| Ppid | 11.72 | 6.34 | 45.89 | 0.00022026 | Peptidylprolyl isomerase D (cyclophilin D) |
| Dock8 | 1.85 | 1.01 | 45.74 | 0.00050617 | dedicator of cytokinesis 8 |
| Ninj1 | 54.57 | 29.61 | 45.73 | 0.00006020 | ninjurin 1 |
| Tmem205 | 166.04 | 90.17 | 45.69 | 0.00012054 | transmembrane protein 205 |
| Nudt8 | 24.88 | 13.51 | 45.68 | 0.00042904 | nudix (nucleoside diphosphate linked moiety X)-type motif 8 |
| Cyp2a4 | 63.82 | 34.70 | 45.63 | 0.00012065 | cytochrome P450, family 2, subfamily a, polypeptide 4 |
| Eif5a | 248.31 | 135.12 | 45.58 | 0.00177426 | eukaryotic translation initiation factor 5A |
| Itgb8 | 3.97 | 2.16 | 45.57 | 0.00101421 | integrin beta 8 |
| Ces2d-ps | 10.67 | 5.81 | 45.55 | 0.00027139 | Carboxylesterase 2D, pseudogene |
| Ndfip1 | 20.06 | 10.94 | 45.46 | 0.00085973 | Nedd4 family interacting protein 1 |
| Slc22a2 | 67.60 | 36.88 | 45.45 | 0.00022363 | solute carrier family 22 (organic cation transporter), member 2 |
| Reps2 | 2.28 | 1.25 | 45.38 | 0.00039561 | RALBP1 associated Eps domain containing protein 2 |
| Cml5 | 202.50 | 110.65 | 45.36 | 0.00042621 | camello-like 5 |
| Ggct | 10.30 | 5.63 | 45.32 | 0.00100253 | gamma-glutamyl cyclotransferase |
| Zfp101 | 3.92 | 2.15 | 45.23 | 0.00192423 | zinc finger protein 101 |
| Zpld1 | 4.05 | 2.22 | 45.19 | 0.00221447 | zona pellucida like domain containing 1 |
| Grb7 | 27.68 | 15.20 | 45.08 | 0.00007840 | growth factor receptor bound protein 7 |
| Arf4 | 100.98 | 55.47 | 45.06 | 0.00054128 | ADP-ribosylation factor 4 |
| Snx6 | 140.42 | 77.17 | 45.04 | 0.00108523 | similar to sorting nexin 6; sorting nexin 6 |
| Pigt | 7.16 | 3.93 | 45.02 | 0.00042300 | Phosphatidylinositol glycan anchor biosynthesis, class T |
| Tmem39a | 3.90 | 2.14 | 45.00 | 0.00176628 | transmembrane protein 39a |
| Ccdc6 | 32.68 | 18.03 | 44.84 | 0.00067193 | coiled-coil domain containing 6 |
| Chmp6 | 27.81 | 15.45 | 44.44 | 0.00012846 | chromatin modifying protein 6 |
| Mrpl44 | 28.34 | 15.85 | 44.10 | 0.00014571 | mitochondrial ribosomal protein L44 |
| Psmd7 | 43.41 | 24.27 | 44.09 | 0.00012690 | proteasome (prosome, macropain) 26S subunit, non-ATPase, 7 |
| Ran | 16.46 | 9.21 | 44.08 | 0.00013824 | RAN, member RAS oncogene family |
| Sec23b | 51.79 | 28.97 | 44.07 | 0.00059426 | similar to SEC23B; SEC23B (S. cerevisiae) |
| 0610010O12Rik | 458.71 | 256.91 | 43.99 | 0.00086485 | RIKEN cDNA 0610010O12 gene |
| Trmt112 | 20.24 | 11.35 | 43.96 | 0.00078536 | TRNA methyltransferase 11-2 |
| Usp20 | 9.85 | 5.52 | 43.95 | 0.00013643 | ubiquitin specific peptidase 20 |
| Fam195a | 44.98 | 25.22 | 43.94 | 0.00018801 | RIKEN cDNA 9530058B02 gene |
| Ogfod2 | 10.09 | 5.66 | 43.93 | 0.00109070 | 2-oxoglutarate and iron-dependent oxygenase domain containing 2 |
| Mep1b | 59.54 | 33.42 | 43.88 | 0.00052655 | meprin 1 beta |
| Pth1r | 44.24 | 24.85 | 43.84 | 0.00021065 | parathyroid hormone 1 receptor |
| ORF63 | 30.44 | 17.11 | 43.79 | 0.00019757 | open reading frame 63 |
| Ralb | 9.86 | 5.54 | 43.78 | 0.00037027 | v-ral simian leukemia viral oncogene homolog B (ras related) |
| Nudt19 | 46.61 | 26.23 | 43.72 | 0.00025483 | nudix (nucleoside diphosphate linked moiety X)-type motif 19 |
| Dusp11 | 6.60 | 3.72 | 43.67 | 0.00014909 | dual specificity phosphatase 11 (RNA/RNP complex 1-interacting) |
| Epb4.9 | 8.53 | 4.81 | 43.57 | 0.00039461 | erythrocyte protein band 4.9 |
| Galns | 38.64 | 21.82 | 43.52 | 0.00070431 | galactosamine (N-acetyl)-6-sulfate sulfatase |
| Acmsd | 35.01 | 19.78 | 43.51 | 0.00051375 | amino carboxymuconate semialdehyde decarboxylase |
| Abhd3 | 78.38 | 44.29 | 43.49 | 0.00048733 | abhydrolase domain containing 3 |
| Chd3 | 1.63 | 0.92 | 43.43 | 0.00208794 | chromodomain helicase DNA binding protein 3 |
| Ncor1 | 6.52 | 3.69 | 43.43 | 0.00018387 | nuclear receptor co-repressor 1 |
| Zfp830 | 7.12 | 4.04 | 43.28 | 0.00039700 | zinc finger protein 830 |
| Aldh3a2 | 63.85 | 36.24 | 43.24 | 0.00109008 | aldehyde dehydrogenase family 3, subfamily A2 |
| 2410066E13Rik | 23.08 | 13.10 | 43.23 | 0.00020090 | RIKEN cDNA 2410066E13 gene |
| Edem1 | 17.49 | 9.95 | 43.09 | 0.00040138 | ER degradation enhancer, mannosidase alpha-like 1 |
| Nadk | 16.22 | 9.24 | 43.05 | 0.00021777 | NAD kinase |
| Tars | 12.69 | 7.23 | 43.03 | 0.00026716 | threonyl-tRNA synthetase |
| Gcnt2 | 13.34 | 7.61 | 42.93 | 0.00071640 | glucosaminyl (N-acetyl) transferase 2, I-branching enzyme |
| Fads2 | 10.80 | 6.18 | 42.83 | 0.00134932 | fatty acid desaturase 2 |
| Agphd1 | 44.15 | 25.25 | 42.80 | 0.00163870 | RIKEN cDNA C630028N24 gene |
| Gpr137b-ps | 20.29 | 11.62 | 42.75 | 0.00026468 | G protein-coupled receptor 137B, pseudogene |
| 1810046J19Rik | 151.34 | 86.70 | 42.71 | 0.00051091 | RIKEN cDNA 1810046J19 gene |
| Tardbp | 11.89 | 6.81 | 42.70 | 0.00227804 | predicted gene 13886; TAR DNA binding protein |
| Uap1 | 24.18 | 13.86 | 42.67 | 0.00025494 | UDP-N-acetylglucosamine pyrophosphorylase 1 |
| Mbd1 | 10.69 | 6.15 | 42.45 | 0.00052133 | methyl-CpG binding domain protein 1 |
| Sgta | 28.23 | 16.25 | 42.44 | 0.00025967 | small glutamine-rich tetratricopeptide repeat (TPR)-containing, alpha |
| Opa3 | 6.13 | 3.53 | 42.41 | 0.00218185 | optic atrophy 3 (human) |
| Coq9 | 109.88 | 63.32 | 42.37 | 0.00109567 | coenzyme Q9 homolog (yeast) |
| Ndrg2 | 56.98 | 32.90 | 42.26 | 0.00161853 | N-myc downstream regulated gene 2 |
| D4Ertd22e | 11.99 | 6.92 | 42.26 | 0.00158014 | DNA segment, Chr 4, ERATO Doi 22, expressed |
| Srpk1 | 13.92 | 8.07 | 42.05 | 0.00039191 | serine/arginine-rich protein specific kinase 1 |
| St6galnac2 | 66.01 | 38.30 | 41.98 | 0.00085498 | ST6 (alpha-N-acetyl-neuraminyl-2,3-beta-galactosyl-1,3)-N-acetylgalactosaminide alpha-2,6-sialyltransferase 2 |
| Htra1 | 8.48 | 4.93 | 41.85 | 0.00145668 | HtrA serine peptidase 1 |
| Fubp1 | 5.68 | 3.31 | 41.82 | 0.00035915 | far upstream element (FUSE) binding protein 1 |
| Add1 | 8.63 | 5.02 | 41.81 | 0.00098608 | adducin 1 (alpha) |
| Rab1b | 13.36 | 7.78 | 41.77 | 0.00073494 | RAB1B, member RAS oncogene family |
| Acbd4 | 12.60 | 7.35 | 41.69 | 0.00066599 | acyl-Coenzyme A binding domain containing 4 |
| D17Wsu104e | 61.44 | 35.93 | 41.52 | 0.00040847 | DNA segment, Chr 17, Wayne State University 104, expressed |
| Mlxipl | 4.65 | 2.72 | 41.50 | 0.00224209 | MLX interacting protein-like |
| Rangap1 | 5.13 | 3.01 | 41.37 | 0.00245327 | RAN GTPase activating protein 1 |
| Nucb1 | 13.29 | 7.79 | 41.34 | 0.00047329 | nucleobindin 1 |
| Cxcr7 | 23.60 | 13.85 | 41.30 | 0.00050694 | chemokine (C-X-C motif) receptor 7 |
| Nhp2 | 94.93 | 55.74 | 41.28 | 0.00151898 | NHP2 ribonucleoprotein homolog (yeast) |
| Coro1b | 15.35 | 9.04 | 41.09 | 0.00071975 | coronin, actin binding protein 1B |
| Coasy | 42.04 | 24.78 | 41.06 | 0.00073822 | Coenzyme A synthase |
| Tpk1 | 48.86 | 28.84 | 40.97 | 0.00111189 | thiamine pyrophosphokinase |
| Ccnl2 | 7.02 | 4.14 | 40.94 | 0.00222407 | cyclin L2 |
| Ddx41 | 8.83 | 5.21 | 40.92 | 0.00152204 | DEAD (Asp-Glu-Ala-Asp) box polypeptide 41 |
| Snx3 | 53.96 | 31.91 | 40.86 | 0.00056575 | sorting nexin 3 |
| Selm | 23.52 | 13.92 | 40.82 | 0.00192220 | selenoprotein M |
| Slc35b1 | 52.98 | 31.39 | 40.75 | 0.00066301 | solute carrier family 35, member B1 |
| Plau | 25.65 | 15.20 | 40.73 | 0.00065720 | plasminogen activator, urokinase |
| Derl2 | 27.26 | 16.16 | 40.71 | 0.00092113 | Der1-like domain family, member 2 |
| Rabggta | 31.84 | 18.88 | 40.70 | 0.00073484 | Rab geranylgeranyl transferase, a subunit |
| Ush1c | 34.98 | 20.75 | 40.67 | 0.00096944 | Usher syndrome 1C homolog (human) |
| 2810004N23Rik | 17.16 | 10.20 | 40.55 | 0.00129113 | RIKEN cDNA 2810004N23 gene |
| Edf1 | 61.84 | 36.79 | 40.50 | 0.00074409 | predicted gene 11964; endothelial differentiation-related factor 1 |
| Spcs2 | 19.40 | 11.54 | 40.50 | 0.00061465 | signal peptidase complex subunit 2 homolog (S. cerevisiae) |
| Top1 | 10.41 | 6.19 | 40.48 | 0.00063240 | topoisomerase (DNA) I |
| Pcyt2 | 82.17 | 48.98 | 40.40 | 0.00162685 | phosphate cytidylyltransferase 2, ethanolamine |
| Nuak2 | 6.33 | 3.78 | 40.21 | 0.00207691 | NUAK family, SNF1-like kinase, 2 |
| Fam32a | 49.79 | 29.78 | 40.20 | 0.00092839 | RIKEN cDNA 2510049I19 gene |
| Tgm2 | 17.12 | 10.25 | 40.16 | 0.00076461 | transglutaminase 2, C polypeptide |
| Fam173b | 24.09 | 14.42 | 40.15 | 0.00126581 | family with sequence similarity 173, member B |
| H6pd | 17.91 | 10.72 | 40.14 | 0.00101910 | hexose-6-phosphate dehydrogenase (glucose 1-dehydrogenase) |
| Myo7b | 2.68 | 1.61 | 40.07 | 0.00217956 | myosin VIIB |
| Gimap9 | 14.90 | 8.94 | 40.02 | 0.00200146 | GTPase, IMAP family member 9 |
| Tmem64 | 4.88 | 2.93 | 40.00 | 0.00144881 | transmembrane protein 64 |
| Psmd6 | 61.93 | 37.19 | 39.95 | 0.00083534 | proteasome (prosome, macropain) 26S subunit, non-ATPase, 6 |
| Dpp7 | 40.42 | 24.32 | 39.83 | 0.00092259 | dipeptidylpeptidase 7 |
| Krt7 | 31.78 | 19.12 | 39.83 | 0.00088290 | keratin 7 |
| Ccdc107 | 76.43 | 46.01 | 39.80 | 0.00082268 | hypothetical protein LOC622404 |
| Itgb6 | 12.38 | 7.46 | 39.76 | 0.00160111 | integrin beta 6 |
| Arsb | 9.43 | 5.68 | 39.70 | 0.00087360 | arylsulfatase B |
| Igfbp4 | 38.59 | 23.43 | 39.28 | 0.00117834 | insulin-like growth factor binding protein 4 |
| Echdc2 | 35.73 | 21.70 | 39.28 | 0.00102455 | enoyl Coenzyme A hydratase domain containing 2 |
| Treh | 51.53 | 31.37 | 39.12 | 0.00174737 | trehalase (brush-border membrane glycoprotein) |
| Fkbp1a | 24.92 | 15.19 | 39.02 | 0.00115462 | FK506 binding protein 1a |
| Psmd4 | 48.36 | 29.49 | 39.01 | 0.00108740 | proteasome (prosome, macropain) 26S subunit, non-ATPase, 4 |
| Ybx1 | 11.86 | 7.24 | 38.99 | 0.00173364 | predicted gene 6540; predicted gene 11560; similar to nuclease sensitive element binding protein 1; Y box protein 1 |
| Chchd2 | 65.89 | 40.28 | 38.86 | 0.00119326 | Coiled-coil-helix-coiled-coil-helix domain containing 2 |
| Fndc3a | 3.53 | 2.16 | 38.85 | 0.00215292 | fibronectin type III domain containing 3A |
| Lman2 | 13.39 | 8.21 | 38.69 | 0.00125359 | lectin, mannose-binding 2 |
| Cib1 | 95.41 | 58.53 | 38.65 | 0.00135963 | calcium and integrin binding 1 (calmyrin) |
| Ddrgk1 | 48.78 | 30.01 | 38.48 | 0.00137509 | DDRGK domain containing 1 |
| Mrps7 | 33.40 | 20.60 | 38.33 | 0.00142982 | mitchondrial ribosomal protein S7 |
| Clic4 | 9.95 | 6.14 | 38.28 | 0.00143449 | chloride intracellular channel 4 (mitochondrial) |
| Galnt1 | 7.12 | 4.40 | 38.18 | 0.00238450 | UDP-N-acetyl-alpha-D-galactosamine:polypeptide N-acetylgalactosaminyltransferase 1 |
| Mrpl34 | 77.50 | 48.14 | 37.89 | 0.00186273 | mitochondrial ribosomal protein L34 |
| Cux1 | 13.63 | 8.46 | 37.89 | 0.00235965 | cut-like homeobox 1 |
| Wdr18 | 12.90 | 8.09 | 37.29 | 0.00211071 | WD repeat domain 18 |

**Suppl. Table S3** Genes upregulated in untreated diabetes (relative to control) and decreased (i.e., reverted toward control expression level) with losartan treatment (relative to untreated diabetes) (n = 38; alphabetized by gene symbol)

| 1300015D01Rik |
| --- |
| 2900092D14Rik |
| 3010026O09Rik |
| 6030446N20Rik |
| 8430408G22Rik |
| Bbox1 |
| Bhmt |
| Cirbp |
| Cpm |
| Dixdc1 |
| Ell3 |
| Elmod3 |
| Fam134b |
| Fam13a |
| Gm129 |
| Gucy1a3 |
| Herc3 |
| Igfbp1 |
| Il5ra |
| Lass4 |
| Lgals4 |
| Lpar2 |
| Lrrc66 |
| Lypd6 |
| Mfsd7c |
| Mogat2 |
| Pdk4 |
| Pttg1 |
| Sh3bp5 |
| Slc22a29 |
| Slc28a1 |
| Slc5a12 |
| Slc6a19 |
| Slc7a12 |
| Tgfbr3 |
| Txnip |
| Upp2 |
| Xpnpep2 |

**Suppl. Table S4** Genes upregulated in diabetes and further upregulated with losartan treatment (n = 10; alphabetized by gene symbol)

| Cyp27b1 |
| --- |
| Fga |
| Fgg |
| Gc |
| Gm16379 |
| Mgst1 |
| Mt1 |
| Mt2 |
| Npl |
| Plk3 |

**Suppl. Table S5** Genes upregulated in untreated diabetes (relative to control) and unaffected with losartan treatment (relative to untreated diabetes) (n = 137; alphabetized by gene symbol)

| 2310001A20Rik |
| --- |
| Abcb1b |
| Abcd4 |
| Acaa2 |
| Acer2 |
| Acnat1 |
| Acot2 |
| Acot3 |
| Acox2 |
| Adamts5 |
| Agmo |
| Agpat9 |
| Akr1c12 |
| Akr1c13 |
| Akr1d1 |
| Aldh1a1 |
| Aldh1a7 |
| Aldoc |
| Amotl1 |
| Angptl3 |
| Angptl4 |
| Anxa1 |
| Apob |
| Aqp3 |
| Aqp4 |
| BC089597 |
| Btg2 |
| Car14 |
| Cbr1 |
| Cdk18 |
| Clca1 |
| Clec2h |
| Cp |
| Cpb2 |
| Ctgf |
| Cyp2a5 |
| Cyp2c44 |
| Cyp2d22 |
| Cyp2d26 |
| Cyp4a31 |
| Ddc |
| Dnase1 |
| Eif4ebp3 |
| Elovl6 |
| Ephx1 |
| F2r |
| Fabp3 |
| Fas |
| Fbln5 |
| Fbp2 |
| Fbxo21 |
| Fmo4 |
| Folh1 |
| Ftcd |
| G0s2 |
| Gbp8 |
| Gm10639 |
| Gpihbp1 |
| Gsta1 |
| Gsta2 |
| Gsta3 |
| Gsta4 |
| Gsto1 |
| Hacl1 |
| Hccs |
| Hddc2 |
| Hebp1 |
| Ifi27l2a |
| Inpp5k |
| Iqgap2 |
| Iyd |
| Klf10 |
| Klf13 |
| Ktn1 |
| Kynu |
| Lgals3bp |
| Lgr4 |
| Lpin2 |
| Lrat |
| Lyplal1 |
| Mal2 |
| Maoa |
| March10 |
| Mcm5 |
| Mfsd4 |
| Mgmt |
| Mif |
| Mosc1 |
| Mpzl2 |
| Msl3 |
| Muc1 |
| Muc20 |
| Nqo1 |
| Nudt7 |
| Nupr1 |
| Pdk2 |
| Pex11a |
| Plat |
| Pnpla7 |
| Prim1 |
| Prlr |
| Prodh2 |
| Prom1 |
| Prps2 |
| Ptrf |
| Rab30 |
| Rgs5 |
| Rpp21 |
| S100a6 |
| Scg5 |
| Sepw1 |
| Serping1 |
| Sfxn2 |
| Sgk1 |
| Slc14a2 |
| Slc26a6 |
| Slc2a5 |
| Slc38a3 |
| Slc5a9 |
| Slc6a8 |
| Sparcl1 |
| Spon1 |
| Spp2 |
| Stc1 |
| Stk39 |
| Stra6 |
| Svopl |
| Tipin |
| Tln2 |
| Tmem43 |
| Tmem86b |
| Tob2 |
| Trf |
| Txndc16 |
| Ugt2b34 |
| Uroc1 |
| Utp20 |

**Suppl. Table S6** Genes downregulated in untreated diabetes (relative to control) and reverted toward control expression level with losartan treatment (relative to untreated diabetes) (n = 60; alphabetized by gene symbol)

| 0610010O12Rik |
| --- |
| 2810004N23Rik |
| Aacs |
| Actg1 |
| Banp |
| Bcl7b |
| Bsnd |
| Calr |
| Chordc1 |
| Cish |
| Cndp2 |
| Creld2 |
| Cxcr7 |
| Ddo |
| Dnajb11 |
| Dusp11 |
| Dynll1 |
| Edf1 |
| Epb4.9 |
| Fubp1 |
| Gm6788 |
| Gm6900 |
| Grwd1 |
| Hmox1 |
| Hnrnpc |
| Hnrnpm |
| Hsf1 |
| Hsp90aa1 |
| Hspa5 |
| Hspa8 |
| Hsph1 |
| Hyou1 |
| Kif12 |
| Krt7 |
| Leo1 |
| Manf |
| Mettl7b |
| Mif4gd |
| Mrpl34 |
| Ninj1 |
| Nuak2 |
| Osgin1 |
| P4ha1 |
| Prr5 |
| Rhoc |
| Sdf2l1 |
| Sec24d |
| Serpinh1 |
| Sipa1l1 |
| Slc16a14 |
| Slc25a19 |
| Tcfec |
| Tfrc |
| Tgm2 |
| Tnfrsf12a |
| Top1 |
| Tpt1 |
| Trpv4 |
| Tst |
| Ywhag |

**Suppl. Table S7** Genes downregulated in untreated diabetes (relative to control) and further decreased with losartan treatment (relative to untreated diabetes) (n = 5; alphabetized by gene symbol)

| Frzb |
| --- |
| Itih2 |
| Kif20b |
| Mep1b |
| Rnf24 |

**Suppl. Table S8** Genes downregulated in untreated diabetes (relative to control) and unaffected by losartan treatment (relative to untreated diabetes) (n = 388; alphabetized by gene symbol)

| 0610011F06Rik |
| --- |
| 0610012H03Rik |
| 1300002K09Rik |
| 1810046J19Rik |
| 2300009A05Rik |
| 2410066E13Rik |
| 2610019F03Rik |
| 2810407C02Rik |
| 4930502E18Rik |
| 5730528L13Rik |
| 9030425E11Rik |
| Abhd2 |
| Abhd3 |
| Acbd4 |
| Ace |
| Acmsd |
| Acsm3 |
| Acss3 |
| Add1 |
| Adh1 |
| Afmid |
| Agphd1 |
| Agps |
| Ahsa1 |
| Ahsa2 |
| Aifm3 |
| Akap2 |
| Akr1c14 |
| Aldh3a2 |
| Als2cr4 |
| Amacr |
| Amdhd2 |
| Angptl7 |
| Anxa13 |
| Apoa1 |
| Apoa4 |
| Apoh |
| Arf1 |
| Arf4 |
| Arhgap15 |
| Arhgef16 |
| Arsb |
| Ascc2 |
| Aspdh |
| Aspg |
| Ass1 |
| Atf4 |
| Atp11a |
| Atp9a |
| Aup1 |
| B4galt5 |
| BC021614 |
| BC026585 |
| Bcl6 |
| Bdh1 |
| C1qtnf3 |
| C4a |
| Cacybp |
| Car4 |
| Car9 |
| Cbs |
| Ccdc107 |
| Ccdc6 |
| Cckar |
| Ccnl2 |
| Cd36 |
| Cd55 |
| Cdk2ap2 |
| Ceacam2 |
| Ces1f |
| Ces2b |
| Ces2c |
| Ces2d-ps |
| Chchd2 |
| Chd3 |
| Chmp6 |
| Chrna4 |
| Chtf8 |
| Cib1 |
| Cidea |
| Cldn9 |
| Clic4 |
| Cml2 |
| Cml5 |
| Cmtm6 |
| Coasy |
| Col6a6 |
| Coq9 |
| Coro1b |
| Cpe |
| Cpsf6 |
| Csf1r |
| Csrp2 |
| Ctxn3 |
| Cux1 |
| Cyb561 |
| Cyb5r3 |
| Cyp24a1 |
| Cyp2a4 |
| Cyp2b10 |
| Cyp2j13 |
| Cyp4a12a |
| Cyp4a12b |
| Cyp51 |
| Cyp7b1 |
| D17Wsu104e |
| D4Ertd22e |
| Ddost |
| Ddrgk1 |
| Ddx41 |
| Defb29 |
| Degs2 |
| Derl1 |
| Derl2 |
| Dhdds |
| Dnaja1 |
| Dnajb1 |
| Dnajc22 |
| Dnajc3 |
| Dock8 |
| Dpp7 |
| Dusp6 |
| E030010A14Rik |
| Echdc2 |
| Edem1 |
| Egf |
| Eif5a |
| Eme2 |
| Entpd4 |
| Erc2 |
| Erp29 |
| Erp44 |
| Esr2 |
| Etv1 |
| Evc |
| Fads2 |
| Fam132a |
| Fam173b |
| Fam195a |
| Fam32a |
| Far1 |
| Farsb |
| Fcamr |
| Fkbp1a |
| Fkbp4 |
| Fmo5 |
| Fndc3a |
| Galns |
| Galnt1 |
| Galntl4 |
| Gas2 |
| Gcgr |
| Gcnt2 |
| Gdi2 |
| Ggct |
| Gimap9 |
| Gjb2 |
| Glyctk |
| Gm12824 |
| Gm13498 |
| Gm15348 |
| Gm15421 |
| Gm2016 |
| Gm5069 |
| Gm5662 |
| Gm6300 |
| Gm6614 |
| Gm7092 |
| Gm853 |
| Gmppb |
| Gpr137b-ps |
| Grb7 |
| Grtp1 |
| Gusb |
| H6pd |
| Hist1h2bc |
| Hist1h4i |
| Hmgcr |
| Hpn |
| Hsd11b1 |
| Hsd17b11 |
| Hsd17b13 |
| Hsp90ab1 |
| Hsp90b1 |
| Hspa1a |
| Hspa1b |
| Hspa4l |
| Hspb1 |
| Hspd1 |
| Hspe1 |
| Htra1 |
| Idi1 |
| Ifrd2 |
| Igfbp4 |
| Igj |
| Il13ra1 |
| Il34 |
| Inmt |
| Irf5 |
| Irf6 |
| Itgb6 |
| Itgb8 |
| Itpk1 |
| Kcnk5 |
| Klc4 |
| Lasp1 |
| Ldhd |
| Lipo1 |
| Lman2 |
| Lman2l |
| Lmbrd2 |
| Lpl |
| Lrp12 |
| Ly6a |
| Ly6e |
| Lztr1 |
| Mbd1 |
| Mdk |
| Me1 |
| Met |
| Mfap3l |
| Mlec |
| Mlxipl |
| Morf4l2 |
| Mpv17l |
| Mrpl44 |
| Mrps7 |
| Mtmr7 |
| Mvd |
| Myo5a |
| Myo7b |
| Nadk |
| Ncln |
| Ncoa5 |
| Ncor1 |
| Ndfip1 |
| Ndrg2 |
| Necap2 |
| Nhp2 |
| Nipal2 |
| Nlrc4 |
| Npdc1 |
| Nt5e |
| Ntn1 |
| Nucb1 |
| Nudt19 |
| Nudt8 |
| Odc1 |
| Ogfod2 |
| Opa3 |
| ORF63 |
| Osta |
| Ostc |
| P4ha2 |
| P4hb |
| Papss1 |
| Pcyox1l |
| Pcyt2 |
| Pdia3 |
| Pdia4 |
| Pdia6 |
| Pdk3 |
| Pdzd3 |
| Pecr |
| Pi4ka |
| Pigt |
| Pion |
| Pla2g6 |
| Plau |
| Pllp |
| Pmepa1 |
| Por |
| Ppic |
| Ppid |
| Proc |
| Psen1 |
| Psmd4 |
| Psmd6 |
| Psmd7 |
| Pth1r |
| Ptpru |
| Pxn |
| Pzp |
| Rab11fip3 |
| Rab1b |
| Rab38 |
| Rabggta |
| Ralb |
| Ran |
| Rangap1 |
| Rbm14 |
| Rbp1 |
| Rce1 |
| Rdh10 |
| Reep5 |
| Reps2 |
| Rgl1 |
| Rhox6 |
| Rhpn1 |
| Rlf |
| Rmrp |
| Rnf145 |
| Rpl3l |
| Rpn1 |
| Rtp3 |
| Scrn2 |
| Sec14l3 |
| Sec23b |
| Sel1l |
| Selm |
| Serpinf2 |
| Sgta |
| Sh3bp2 |
| Sh3tc1 |
| Slc17a3 |
| Slc22a12 |
| Slc22a2 |
| Slc22a28 |
| Slc22a30 |
| Slc22a7 |
| Slc33a1 |
| Slc35b1 |
| Slc39a11 |
| Slc46a3 |
| Slc6a15 |
| Slc9a3r1 |
| Slc9a8 |
| Slco1a1 |
| Slco1a5 |
| Slco1a6 |
| Slco3a1 |
| Snx3 |
| Snx31 |
| Snx6 |
| Spcs2 |
| Spns1 |
| Srd5a2 |
| Srpk1 |
| St13 |
| St6galnac2 |
| St8sia1 |
| Stip1 |
| Susd2 |
| Sypl2 |
| Tardbp |
| Tars |
| Tbx10 |
| Tc2n |
| Timd2 |
| Tlcd2 |
| Tmco3 |
| Tmem109 |
| Tmem169 |
| Tmem176a |
| Tmem205 |
| Tmem30a |
| Tmem39a |
| Tmem45b |
| Tmem64 |
| Tmtc4 |
| Tpk1 |
| Tra2a |
| Tradd |
| Treh |
| Trmt112 |
| Trps1 |
| Tuba1c |
| Txnl1 |
| Uap1 |
| Ubiad1 |
| Ubxn10 |
| Ucp1 |
| Ush1c |
| Usp10 |
| Usp20 |
| Vill |
| Vps8 |
| Vwa2 |
| Wdr18 |
| Xbp1 |
| Ybx1 |
| Zbtb20 |
| Zfp101 |
| Zfp810 |
| Zfp830 |
| Zfp937 |
| Znrf1 |
| Zpld1 |

**Suppl. Table S9** Genes UPregulated with losartan treatment (relative to untreated diabetes) (n = 174; ranked by fold-induction)

| **gene_id** | **DM** | **DM_LOS** | **fold_change** | **p_value** | **gene name** |
| --- | --- | --- | --- | --- | --- |
| Fgg | 11.33 | 64.10 | 5.66 | 0.00000000 | fibrinogen gamma chain |
| Ren1 | 88.16 | 419.40 | 4.76 | 0.00000000 | renin 1 structural |
| Insc | 0.41 | 1.69 | 4.12 | 0.00000074 | inscuteable homolog (Drosophila) |
| Fam129a | 0.85 | 3.37 | 3.98 | 0.00000000 | family with sequence similarity 129, member A |
| Creld2 | 3.50 | 13.91 | 3.97 | 0.00000000 | cysteine-rich with EGF-like domains 2 |
| Manf | 7.81 | 29.00 | 3.71 | 0.00000000 | mesencephalic astrocyte-derived neurotrophic factor |
| Fgb | 2.36 | 8.49 | 3.60 | 0.00000000 | fibrinogen beta chain |
| Actg1 | 25.06 | 87.46 | 3.49 | 0.00000000 | Actin, gamma, cytoplasmic 1 |
| Hmox1 | 27.10 | 92.66 | 3.42 | 0.00000000 | heme oxygenase (decycling) 1 |
| C4b | 0.30 | 1.02 | 3.34 | 0.00000015 | Complement component 4B (Chido blood group) |
| Nuak2 | 3.78 | 12.20 | 3.23 | 0.00000000 | NUAK family, SNF1-like kinase, 2 |
| Cyp27b1 | 12.30 | 38.54 | 3.13 | 0.00000000 | cytochrome P450, family 27, subfamily b, polypeptide 1 |
| Hspa5 | 82.59 | 258.68 | 3.13 | 0.00000000 | heat shock protein 5 |
| Sh3tc1 | 0.61 | 1.86 | 3.06 | 0.00000001 | SH3 domain and tetratricopeptide repeats 1 |
| Tmem52 | 1.56 | 4.75 | 3.06 | 0.00008970 | transmembrane protein 52 |
| Grem2 | 0.28 | 0.84 | 2.99 | 0.00008470 | gremlin 2 homolog, cysteine knot superfamily (Xenopus laevis) |
| Sdf2l1 | 0.74 | 2.19 | 2.96 | 0.00108756 | stromal cell-derived factor 2-like 1 |
| Prr5 | 5.35 | 15.25 | 2.85 | 0.00000000 | Rho GTPase activating protein 8; proline rich 5 (renal) |
| Gm6788 | 51.60 | 142.77 | 2.77 | 0.00000000 | predicted gene 6788 |
| Dynll1 | 25.25 | 67.01 | 2.65 | 0.00000000 | Dynein light chain LC8-type 1 |
| Ftl1 | 1796.27 | 4733.98 | 2.64 | 0.00000196 | ferritin light chain 1 |
| Krt19 | 1.46 | 3.78 | 2.59 | 0.00002980 | keratin 19 |
| Mcoln3 | 3.78 | 9.69 | 2.56 | 0.00000000 | mucolipin 3 |
| C3 | 1.96 | 4.91 | 2.51 | 0.00000000 | complement component 3 |
| Slc16a6 | 2.45 | 6.12 | 2.50 | 0.00000821 | solute carrier family 16 (monocarboxylic acid transporters), member 6 |
| Serpinh1 | 1.37 | 3.37 | 2.45 | 0.00046534 | serine (or cysteine) peptidase inhibitor, clade H, member 1 |
| Jub | 0.58 | 1.40 | 2.44 | 0.00005440 | ajuba |
| Myc | 0.85 | 2.08 | 2.43 | 0.00069055 | myelocytomatosis oncogene |
| Rcan1 | 35.72 | 86.51 | 2.42 | 0.00000000 | regulator of calcineurin 1 |
| Timp3 | 370.08 | 894.12 | 2.42 | 0.00003310 | tissue inhibitor of metalloproteinase 3 |
| Tchhl1 | 0.98 | 2.37 | 2.42 | 0.00007940 | trichohyalin-like 1 |
| Tnfrsf12a | 9.38 | 22.27 | 2.37 | 0.00141708 | tumor necrosis factor receptor superfamily, member 12a |
| Hsf1 | 1.15 | 2.70 | 2.35 | 0.00006450 | heat shock factor 1 |
| Chordc1 | 10.64 | 24.82 | 2.33 | 0.00000000 | cysteine and histidine-rich domain (CHORD)-containing, zinc-binding protein 1 |
| Smcp | 4.98 | 11.52 | 2.31 | 0.00000329 | sperm mitochondria-associated cysteine-rich protein |
| Trpv4 | 5.63 | 12.91 | 2.29 | 0.00000000 | transient receptor potential cation channel, subfamily V, member 4 |
| Btc | 2.73 | 6.09 | 2.23 | 0.00000004 | betacellulin, epidermal growth factor family member |
| Slc16a14 | 4.32 | 9.63 | 2.23 | 0.00000002 | solute carrier family 16 (monocarboxylic acid transporters), member 14 |
| Mgst1 | 113.17 | 247.54 | 2.19 | 0.00000000 | microsomal glutathione S-transferase 1 |
| Armc6 | 1.53 | 3.33 | 2.18 | 0.00006450 | armadillo repeat containing 6 |
| Obfc2a | 5.17 | 11.14 | 2.16 | 0.00000000 | oligonucleotide/oligosaccharide-binding fold containing 2A |
| Sipa1l1 | 1.81 | 3.89 | 2.15 | 0.00000005 | signal-induced proliferation-associated 1 like 1 |
| Plk3 | 4.08 | 8.53 | 2.09 | 0.00000049 | polo-like kinase 3 (Drosophila) |
| Hyou1 | 1.10 | 2.28 | 2.07 | 0.00001200 | hypoxia up-regulated 1 |
| Fga | 14.15 | 29.24 | 2.07 | 0.00000518 | fibrinogen alpha chain |
| Krt7 | 19.12 | 39.34 | 2.06 | 0.00000001 | keratin 7 |
| Hspa8 | 97.00 | 199.19 | 2.05 | 0.00000026 | similar to heat shock protein 8; heat shock protein 8 |
| Fam107b | 18.28 | 37.27 | 2.04 | 0.00000002 | family with sequence similarity 107, member B |
| Avpr2 | 6.51 | 13.25 | 2.03 | 0.00000030 | arginine vasopressin receptor 2 |
| Leo1 | 1.86 | 3.74 | 2.01 | 0.00009060 | Leo1, Paf1/RNA polymerase II complex component, homolog (S. cerevisiae) |
| Gm6900 | 11.64 | 23.23 | 2.00 | 0.00000876 | predicted gene 6900 |
| Grwd1 | 2.35 | 4.68 | 1.99 | 0.00007650 | glutamate-rich WD repeat containing 1 |
| Tmco4 | 1.83 | 3.65 | 1.99 | 0.00001270 | transmembrane and coiled-coil domains 4 |
| Cdkn1a | 2.96 | 5.90 | 1.99 | 0.00007570 | cyclin-dependent kinase inhibitor 1A (P21) |
| Epb4.9 | 4.81 | 9.58 | 1.99 | 0.00000033 | erythrocyte protein band 4.9 |
| Add3 | 10.65 | 20.81 | 1.95 | 0.00000201 | adducin 3 (gamma) |
| Cpn1 | 1.54 | 3.00 | 1.95 | 0.00125985 | carboxypeptidase N, polypeptide 1 |
| Mettl7b | 179.74 | 348.98 | 1.94 | 0.00000217 | methyltransferase like 7B |
| Tcfec | 8.54 | 16.53 | 1.93 | 0.00000067 | transcription factor EC |
| Actb | 823.51 | 1578.23 | 1.92 | 0.00089699 | actin, beta |
| Gc | 20.60 | 39.39 | 1.91 | 0.00000064 | group specific component |
| Bcl7b | 1.61 | 3.08 | 1.91 | 0.00239752 | B-cell CLL/lymphoma 7B |
| Kif12 | 3.67 | 7.00 | 1.90 | 0.00001320 | kinesin family member 12 |
| H2-Ab1 | 7.56 | 14.34 | 1.90 | 0.00002040 | Histocompatibility 2, class II antigen A, beta 1 |
| Spns2 | 2.22 | 4.18 | 1.89 | 0.00002740 | spinster homolog 2 (Drosophila) |
| Tacstd2 | 12.49 | 23.40 | 1.87 | 0.00000071 | tumor-associated calcium signal transducer 2 |
| Thbd | 1.51 | 2.82 | 1.87 | 0.00012235 | thrombomodulin |
| P4ha1 | 2.41 | 4.46 | 1.85 | 0.00001290 | procollagen-proline, 2-oxoglutarate 4-dioxygenase (proline 4-hydroxylase), alpha 1 polypeptide |
| Slc16a1 | 1.51 | 2.79 | 1.85 | 0.00006520 | solute carrier family 16 (monocarboxylic acid transporters), member 1 |
| Irak2 | 3.72 | 6.86 | 1.85 | 0.00015889 | interleukin-1 receptor-associated kinase 2 |
| Med29 | 1.83 | 3.34 | 1.83 | 0.00107033 | mediator complex subunit 29; predicted gene 9450 |
| 4930402H24Rik | 2.81 | 5.11 | 1.82 | 0.00000740 | RIKEN cDNA 4930402H24 gene |
| Banp | 1.40 | 2.55 | 1.82 | 0.00019941 | BTG3 associated nuclear protein |
| Tpt1 | 7.20 | 13.05 | 1.81 | 0.00032562 | Tumor protein, translationally-controlled 1 |
| Tbc1d1 | 2.77 | 4.99 | 1.80 | 0.00000751 | TBC1 domain family, member 1 |
| Ywhag | 1.77 | 3.18 | 1.80 | 0.00019678 | tyrosine 3-monooxygenase/tryptophan 5-monooxygenase activation protein, gamma polypeptide |
| Nop2 | 8.32 | 14.93 | 1.79 | 0.00000399 | NOP2 nucleolar protein homolog (yeast) |
| 4933407C03Rik | 3.19 | 5.72 | 1.79 | 0.00013261 | RIKEN cDNA 4933407C03 gene |
| Slc7a8 | 21.31 | 38.15 | 1.79 | 0.00000673 | solute carrier family 7 (cationic amino acid transporter, y+ system), member 8 |
| Fmo1 | 472.58 | 843.76 | 1.79 | 0.00136905 | flavin containing monooxygenase 1 |
| Aqp2 | 7.48 | 13.30 | 1.78 | 0.00005870 | aquaporin 2 |
| Lad1 | 3.25 | 5.74 | 1.76 | 0.00006720 | ladinin |
| Eef1e1 | 16.72 | 29.46 | 1.76 | 0.00001660 | eukaryotic translation elongation factor 1 epsilon 1 |
| Cpsf6 | 1.35 | 2.37 | 1.76 | 0.00009480 | cleavage and polyadenylation specific factor 6 |
| Amd1 | 1.93 | 3.40 | 1.76 | 0.00039766 | S-adenosylmethionine decarboxylase 1 |
| Tmem37 | 117.97 | 207.07 | 1.76 | 0.00001560 | transmembrane protein 37 |
| Creg1 | 7.18 | 12.58 | 1.75 | 0.00002260 | cellular repressor of E1A-stimulated genes 1 |
| Sh3tc2 | 4.32 | 7.54 | 1.75 | 0.00001470 | SH3 domain and tetratricopeptide repeats 2 |
| Fam20c | 1.44 | 2.51 | 1.74 | 0.00167640 | family with sequence similarity 20, member C |
| Sec24d | 1.53 | 2.66 | 1.74 | 0.00058635 | Sec24 related gene family, member D (S. cerevisiae) |
| Mdm2 | 3.11 | 5.42 | 1.74 | 0.00011053 | transformed mouse 3T3 cell double minute 2 |
| Hnrnpm | 3.76 | 6.52 | 1.74 | 0.00207467 | heterogeneous nuclear ribonucleoprotein M |
| Gpatch4 | 4.50 | 7.80 | 1.73 | 0.00049355 | G patch domain containing 4 |
| Bsnd | 2.17 | 3.75 | 1.73 | 0.00066280 | Bartter syndrome, infantile, with sensorineural deafness (Barttin) |
| Alas1 | 85.87 | 148.69 | 1.73 | 0.00013945 | aminolevulinic acid synthase 1 |
| Slc22a23 | 2.58 | 4.44 | 1.72 | 0.00002990 | solute carrier family 22, member 23 |
| Lyz2 | 7.86 | 13.50 | 1.72 | 0.00038632 | lysozyme 2 |
| Wsb1 | 10.83 | 18.60 | 1.72 | 0.00021850 | WD repeat and SOCS box-containing 1 |
| Id2 | 642.03 | 1103.03 | 1.72 | 0.00131612 | inhibitor of DNA binding 2 |
| AW555464 | 1.13 | 1.92 | 1.70 | 0.00045131 | expressed sequence AW555464 |
| Plk2 | 2.32 | 3.94 | 1.70 | 0.00090669 | polo-like kinase 2 (Drosophila) |
| Hexa | 8.66 | 14.68 | 1.69 | 0.00006710 | hexosaminidase A |
| Ndrg1 | 215.50 | 364.05 | 1.69 | 0.00163922 | N-myc downstream regulated gene 1 |
| Cndp2 | 99.93 | 167.81 | 1.68 | 0.00023620 | CNDP dipeptidase 2 (metallopeptidase M20 family) |
| Mt1 | 1055.89 | 1768.75 | 1.68 | 0.00074421 | metallothionein 1 |
| Cish | 5.86 | 9.76 | 1.67 | 0.00020242 | cytokine inducible SH2-containing protein |
| Alpl | 68.42 | 113.96 | 1.67 | 0.00025856 | alkaline phosphatase, liver/bone/kidney |
| Slc3a2 | 18.86 | 31.29 | 1.66 | 0.00005650 | solute carrier family 3 (activators of dibasic and neutral amino acid transport), member 2 |
| Ccnd1 | 16.79 | 27.73 | 1.65 | 0.00006410 | cyclin D1 |
| Gna14 | 2.45 | 4.03 | 1.65 | 0.00080975 | guanine nucleotide binding protein, alpha 14 |
| Ppcs | 6.78 | 11.16 | 1.65 | 0.00063909 | phosphopantothenoylcysteine synthetase |
| Tfrc | 2.49 | 4.07 | 1.63 | 0.00034299 | transferrin receptor |
| Gldc | 9.29 | 15.17 | 1.63 | 0.00007990 | glycine decarboxylase |
| Osgin1 | 43.45 | 70.89 | 1.63 | 0.00014870 | oxidative stress induced growth inhibitor 1 |
| Dynll2 | 6.07 | 9.88 | 1.63 | 0.00088358 | dynein light chain LC8-type 2 |
| Gm8615 | 36.95 | 60.06 | 1.63 | 0.00012421 | Glucosamine-6-phosphate deaminase 1 pseudogene |
| Vcl | 2.77 | 4.49 | 1.62 | 0.00027125 | vinculin |
| 0610010O12Rik | 256.91 | 416.19 | 1.62 | 0.00023272 | RIKEN cDNA 0610010O12 gene |
| Slc25a19 | 17.40 | 28.17 | 1.62 | 0.00014663 | solute carrier family 25 (mitochondrial thiamine pyrophosphate carrier), member 19 |
| Scd2 | 2.90 | 4.70 | 1.62 | 0.00024354 | stearoyl-Coenzyme A desaturase 2 |
| Gnpda1 | 12.76 | 20.66 | 1.62 | 0.00011212 | Glucosamine-6-phosphate deaminase 1 |
| Aacs | 34.92 | 56.51 | 1.62 | 0.00050126 | acetoacetyl-CoA synthetase |
| Ltbr | 4.12 | 6.67 | 1.62 | 0.00147267 | lymphotoxin B receptor |
| Ninj1 | 29.61 | 47.86 | 1.62 | 0.00012444 | ninjurin 1 |
| Cd63 | 647.18 | 1044.58 | 1.61 | 0.00211490 | CD63 antigen |
| Fam176a | 7.85 | 12.65 | 1.61 | 0.00046515 | family with sequence similarity 176, member A |
| Edf1 | 36.79 | 59.09 | 1.61 | 0.00027915 | endothelial differentiation-related factor 1 |
| Hnrnpc | 11.88 | 19.07 | 1.61 | 0.00033123 | heterogeneous nuclear ribonucleoprotein C |
| Ncoa7 | 3.81 | 6.12 | 1.61 | 0.00071272 | nuclear receptor coactivator 7 |
| Hsp90aa1 | 8.62 | 13.81 | 1.60 | 0.00018631 | heat shock protein 90, alpha (cytosolic), class A member 1 |
| 2810004N23Rik | 10.20 | 16.26 | 1.59 | 0.00060236 | RIKEN cDNA 2810004N23 gene |
| Ccdc141 | 1.72 | 2.73 | 1.59 | 0.00107580 | RIKEN cDNA 2610301F02 gene |
| Hbxip | 98.28 | 155.35 | 1.58 | 0.00028465 | hepatitis B virus x interacting protein |
| Mif4gd | 13.92 | 21.99 | 1.58 | 0.00077004 | MIF4G domain containing |
| Rps8 | 13.90 | 21.95 | 1.58 | 0.00240406 | Ribosomal protein S8 |
| Tubb2a | 9.61 | 15.17 | 1.58 | 0.00064065 | tubulin, beta 2A |
| Hmgn1 | 12.16 | 19.18 | 1.58 | 0.00078429 | High mobility group nucleosomal binding domain 1 |
| Fubp1 | 3.31 | 5.21 | 1.58 | 0.00034498 | far upstream element (FUSE) binding protein 1 |
| Dhrs3 | 124.47 | 195.61 | 1.57 | 0.00106088 | dehydrogenase/reductase (SDR family) member 3 |
| Mt2 | 67.15 | 105.51 | 1.57 | 0.00040950 | metallothionein 2 |
| Slc25a45 | 12.15 | 19.07 | 1.57 | 0.00040674 | solute carrier family 25, member 45 |
| Eif1 | 32.90 | 51.37 | 1.56 | 0.00033505 | Eukaryotic translation initiation factor 1 |
| Actn4 | 8.93 | 13.91 | 1.56 | 0.00041904 | actinin alpha 4 |
| Tomm5 | 65.50 | 101.88 | 1.56 | 0.00057618 | translocase of outer mitochondrial membrane 5 homolog (yeast) |
| 2810055F11Rik | 17.15 | 26.67 | 1.56 | 0.00042050 | RIKEN cDNA 2810055F11 gene |
| Ddo | 9.02 | 14.03 | 1.55 | 0.00046576 | D-aspartate oxidase |
| Calr | 156.60 | 243.42 | 1.55 | 0.00232882 | calreticulin |
| Tmem82 | 7.91 | 12.29 | 1.55 | 0.00151256 | transmembrane protein 82 |
| Dnajb11 | 5.08 | 7.88 | 1.55 | 0.00234805 | DnaJ (Hsp40) homolog, subfamily B, member 11 |
| Rorc | 18.02 | 27.83 | 1.54 | 0.00047103 | RAR-related orphan receptor gamma |
| Top1 | 6.19 | 9.57 | 1.54 | 0.00058074 | topoisomerase (DNA) I |
| 1600029D21Rik | 33.65 | 51.94 | 1.54 | 0.00061265 | RIKEN cDNA 1600029D21 gene |
| Phf5a | 12.49 | 19.23 | 1.54 | 0.00081028 | PHD finger protein 5A |
| Hsph1 | 4.75 | 7.29 | 1.54 | 0.00108801 | heat shock 105kDa/110kDa protein 1 |
| Rhoc | 16.31 | 25.05 | 1.54 | 0.00134857 | ras homolog gene family, member C |
| Epb4.1l3 | 3.65 | 5.60 | 1.53 | 0.00138629 | erythrocyte protein band 4.1-like 3 |
| H2-D1 | 50.61 | 77.11 | 1.52 | 0.00123999 | histocompatibility 2, D region locus 1 |
| Aup1 | 42.42 | 64.27 | 1.52 | 0.00128156 | ancient ubiquitous protein 1 |
| Tgm2 | 10.25 | 15.52 | 1.51 | 0.00104065 | transglutaminase 2, C polypeptide |
| Tst | 68.02 | 102.55 | 1.51 | 0.00125053 | thiosulfate sulfurtransferase, mitochondrial |
| Cxcr7 | 13.85 | 20.87 | 1.51 | 0.00098695 | chemokine (C-X-C motif) receptor 7 |
| Npl | 39.01 | 58.74 | 1.51 | 0.00100307 | N-acetylneuraminate pyruvate lyase |
| Dap | 15.14 | 22.68 | 1.50 | 0.00158916 | death-associated protein |
| Ncbp2 | 11.61 | 17.36 | 1.49 | 0.00154267 | nuclear cap binding protein subunit 2 |
| Slc25a25 | 19.97 | 29.78 | 1.49 | 0.00175009 | solute carrier family 25 (mitochondrial carrier, phosphate carrier), member 25 |
| Cnnm4 | 6.20 | 9.25 | 1.49 | 0.00215145 | cyclin M4 |
| Gstp1 | 113.85 | 169.53 | 1.49 | 0.00174125 | Glutathione S-transferase, pi 1 |
| Mrpl34 | 48.14 | 71.62 | 1.49 | 0.00189319 | mitochondrial ribosomal protein L34 |
| Gm16379 | 96.63 | 143.57 | 1.49 | 0.00146885 | predicted gene 16379 |
| Mkks | 10.29 | 15.29 | 1.49 | 0.00229181 | McKusick-Kaufman syndrome protein |
| Aldoart2 | 39.79 | 58.79 | 1.48 | 0.00182054 | aldolase 1, A isoform, retrogene 2 |
| Dusp11 | 3.72 | 5.49 | 1.48 | 0.00204121 | dual specificity phosphatase 11 (RNA/RNP complex 1-interacting) |
| Wfdc2 | 124.31 | 183.06 | 1.47 | 0.00238607 | WAP four-disulfide core domain 2 |
| Pdrg1 | 25.73 | 37.70 | 1.47 | 0.00226666 | p53 and DNA damage regulated 1 |

**Suppl. Table S10** Genes DOWNregulated with losartan treatment (relative to untreated diabetes) (n = 127; ranked by fold-induction)

| **gene_id** | **DM** | **DM/LOS** | **%_decrease** | **p_value** | **Gene name** |
| --- | --- | --- | --- | --- | --- |
| Prss35 | 0.69 | 0.03 | 95.47 | 0.00207154 | protease, serine, 35 |
| Ttr | 1.52 | 0.25 | 83.82 | 0.00123952 | transthyretin |
| Gm129 | 12.95 | 2.18 | 83.17 | 0.00000000 | predicted gene 129 |
| Igfbp1 | 2.44 | 0.47 | 80.68 | 0.00000593 | insulin-like growth factor binding protein 1 |
| Dbp | 7.25 | 1.41 | 80.59 | 0.00015921 | D site albumin promoter binding protein |
| Cirbp | 19.18 | 4.36 | 77.28 | 0.00000000 | cold inducible RNA binding protein |
| 8430408G22Rik | 31.25 | 7.53 | 75.90 | 0.00000000 | RIKEN cDNA 8430408G22 gene |
| Kcnj2 | 1.15 | 0.31 | 73.39 | 0.00000003 | potassium inwardly-rectifying channel, subfamily J, member 2 |
| Upp2 | 52.16 | 16.93 | 67.55 | 0.00000000 | uridine phosphorylase 2 |
| Nr1d1 | 9.18 | 3.20 | 65.11 | 0.00000000 | nuclear receptor subfamily 1, group D, member 1 |
| Gem | 2.68 | 0.98 | 63.46 | 0.00000693 | GTP binding protein (gene overexpressed in skeletal muscle) |
| Cyp2j9 | 4.46 | 1.69 | 62.16 | 0.00000059 | cytochrome P450, family 2, subfamily j, polypeptide 9 |
| Il5ra | 3.01 | 1.14 | 62.01 | 0.00000007 | interleukin 5 receptor, alpha |
| Rasa3 | 0.88 | 0.34 | 61.07 | 0.00041711 | RAS p21 protein activator 3 |
| Lrrc29 | 2.40 | 0.94 | 60.85 | 0.00013635 | leucine rich repeat containing 29 |
| Herc3 | 3.55 | 1.40 | 60.49 | 0.00000000 | hect domain and RLD 3 |
| Gatsl3 | 8.63 | 3.45 | 60.09 | 0.00000007 | GATS protein-like 3 |
| 1300015D01Rik | 2.98 | 1.19 | 60.01 | 0.00000031 | RIKEN cDNA 1300015D01 gene |
| Pttg1 | 16.08 | 6.58 | 59.06 | 0.00000406 | pituitary tumor-transforming gene 1 |
| 2900092D14Rik | 3.43 | 1.44 | 58.12 | 0.00000153 | RIKEN cDNA 2900092D14 gene |
| Lypd6 | 1.66 | 0.70 | 58.04 | 0.00005100 | LY6/PLAUR domain containing 6 |
| Car3 | 27.07 | 11.48 | 57.57 | 0.00001600 | carbonic anhydrase 3 |
| Lgals4 | 13.54 | 5.83 | 56.95 | 0.00000012 | Lectin, galactose binding, soluble 4 |
| 3010026O09Rik | 17.42 | 7.57 | 56.54 | 0.00000005 | RIKEN cDNA 3010026O09 gene |
| Ccdc148 | 1.04 | 0.46 | 56.14 | 0.00074385 | coiled-coil domain containing 148 |
| Dixdc1 | 1.24 | 0.55 | 55.98 | 0.00003220 | DIX domain containing 1 |
| Clip4 | 2.15 | 0.95 | 55.74 | 0.00017406 | CAP-GLY domain containing linker protein family, member 4 |
| Lrrc66 | 9.84 | 4.36 | 55.70 | 0.00000000 | leucine rich repeat containing 66 |
| Bbox1 | 6.11 | 2.73 | 55.29 | 0.00000832 | butyrobetaine (gamma), 2-oxoglutarate dioxygenase 1 (gamma-butyrobetaine hydroxylase) |
| Slc6a19 | 161.65 | 73.01 | 54.84 | 0.00000005 | solute carrier family 6 (neurotransmitter transporter), member 19 |
| Bhmt | 10.29 | 4.69 | 54.44 | 0.00000021 | betaine-homocysteine methyltransferase |
| Mfsd9 | 6.04 | 2.77 | 54.22 | 0.00000015 | major facilitator superfamily domain containing 9 |
| Suv39h1 | 2.39 | 1.10 | 54.16 | 0.00004930 | suppressor of variegation 3-9 homolog 1 (Drosophila) |
| Ppm1k | 7.66 | 3.53 | 53.96 | 0.00000000 | protein phosphatase 1K (PP2C domain containing) |
| Fam134b | 96.73 | 44.79 | 53.70 | 0.00000352 | family with sequence similarity 134, member B |
| Slc28a1 | 6.83 | 3.22 | 52.88 | 0.00000037 | solute carrier family 28 (sodium-coupled nucleoside transporter), member 1 |
| Kif20b | 1.46 | 0.71 | 51.50 | 0.00009220 | kinesin family member 20B |
| Txnip | 91.96 | 45.03 | 51.03 | 0.00000690 | thioredoxin interacting protein |
| Gucy1a3 | 3.01 | 1.49 | 50.49 | 0.00000830 | guanylate cyclase 1, soluble, alpha 3 |
| Dnajc12 | 345.75 | 171.43 | 50.42 | 0.00000239 | DnaJ (Hsp40) homolog, subfamily C, member 12 |
| Mfsd7c | 10.22 | 5.09 | 50.21 | 0.00000019 | major facilitator superfamily domain containing 7C |
| Dusp1 | 4.25 | 2.12 | 50.14 | 0.00016805 | dual specificity phosphatase 1 |
| Ppara | 16.60 | 8.36 | 49.63 | 0.00001060 | peroxisome proliferator activated receptor alpha |
| Tcp11l2 | 13.17 | 6.64 | 49.56 | 0.00000037 | t-complex 11 (mouse) like 2 |
| Bcat1 | 11.37 | 5.76 | 49.38 | 0.00000039 | branched chain aminotransferase 1, cytosolic |
| Fnip2 | 3.55 | 1.82 | 48.83 | 0.00000119 | folliculin interacting protein 2 |
| Rhebl1 | 4.74 | 2.43 | 48.82 | 0.00160276 | Ras homolog enriched in brain like 1 |
| Gcom1 | 2.98 | 1.53 | 48.79 | 0.00059002 | GRINL1A complex locus |
| Cndp1 | 31.97 | 16.40 | 48.70 | 0.00000015 | carnosine dipeptidase 1 (metallopeptidase M20 family) |
| Tmem26 | 1.88 | 0.98 | 48.20 | 0.00013254 | transmembrane protein 26 |
| Itih2 | 1.83 | 0.96 | 47.54 | 0.00176092 | inter-alpha trypsin inhibitor, heavy chain 2 |
| Olfm4 | 4.21 | 2.21 | 47.49 | 0.00101137 | olfactomedin 4 |
| Lpar2 | 1.35 | 0.71 | 47.47 | 0.00041993 | lysophosphatidic acid receptor 2 |
| Afm | 12.82 | 6.76 | 47.26 | 0.00000385 | afamin |
| Erbb3 | 4.34 | 2.32 | 46.65 | 0.00002070 | v-erb-b2 erythroblastic leukemia viral oncogene homolog 3 (avian) |
| Mcm10 | 1.83 | 0.99 | 46.19 | 0.00154049 | minichromosome maintenance deficient 10 (S. cerevisiae) |
| Rab43 | 2.97 | 1.61 | 45.91 | 0.00050785 | RAB43, member RAS oncogene family |
| Fam81a | 3.74 | 2.04 | 45.53 | 0.00024817 | family with sequence similarity 81, member A |
| Gpam | 4.53 | 2.47 | 45.35 | 0.00003900 | glycerol-3-phosphate acyltransferase, mitochondrial |
| Cdkn2c | 9.71 | 5.32 | 45.25 | 0.00044120 | cyclin-dependent kinase inhibitor 2C (p18, inhibits CDK4) |
| Slc7a12 | 11.99 | 6.57 | 45.25 | 0.00020387 | solute carrier family 7 (cationic amino acid transporter, y+ system), member 12 |
| Zfand1 | 12.16 | 6.71 | 44.82 | 0.00004580 | zinc finger, AN1-type domain 1 |
| Klf12 | 1.18 | 0.65 | 44.41 | 0.00184722 | Kruppel-like factor 12 |
| Pdk4 | 5.68 | 3.16 | 44.41 | 0.00004980 | pyruvate dehydrogenase kinase, isoenzyme 4 |
| Sh3bp5 | 5.84 | 3.27 | 44.02 | 0.00015089 | SH3-domain binding protein 5 (BTK-associated) |
| Osbpl9 | 100.07 | 56.12 | 43.92 | 0.00015179 | oxysterol binding protein-like 9 |
| Tmem220 | 6.90 | 3.88 | 43.80 | 0.00199321 | transmembrane protein 220 |
| Srd5a1 | 5.70 | 3.21 | 43.68 | 0.00014652 | steroid 5 alpha-reductase 1 |
| 6030446N20Rik | 3.15 | 1.78 | 43.53 | 0.00147280 | RIKEN cDNA 6030446N20 gene |
| Trim7 | 6.25 | 3.53 | 43.47 | 0.00165271 | tripartite motif-containing 7 |
| Tmem174 | 273.66 | 154.75 | 43.45 | 0.00020985 | transmembrane protein 174 |
| Fam13a | 16.08 | 9.16 | 43.04 | 0.00000944 | family with sequence similarity 13, member A |
| Ace2 | 21.22 | 12.15 | 42.74 | 0.00001050 | angiotensin I converting enzyme (peptidyl-dipeptidase A) 2 |
| D4Wsu53e | 14.48 | 8.31 | 42.61 | 0.00011035 | DNA segment, Chr 4, Wayne State University 53, expressed |
| Lass4 | 2.36 | 1.37 | 42.07 | 0.00165507 | LAG1 homolog, ceramide synthase 4 |
| Xpnpep2 | 2.84 | 1.66 | 41.67 | 0.00118885 | X-prolyl aminopeptidase (aminopeptidase P) 2, membrane-bound |
| Ell3 | 20.58 | 12.10 | 41.23 | 0.00005700 | elongation factor RNA polymerase II-like 3 |
| Frzb | 2.97 | 1.75 | 41.15 | 0.00231268 | frizzled-related protein |
| Rdh16 | 72.37 | 42.66 | 41.06 | 0.00009830 | retinol dehydrogenase 16 |
| 4933407H18Rik | 1.67 | 0.99 | 40.92 | 0.00075590 | RIKEN cDNA 4933407H18 gene |
| Asb9 | 29.60 | 17.50 | 40.90 | 0.00007650 | ankyrin repeat and SOCS box-containing 9 |
| Gm11992 | 14.92 | 8.82 | 40.88 | 0.00011799 | predicted gene 11992 |
| Rnf24 | 4.02 | 2.38 | 40.84 | 0.00065806 | ring finger protein 24 |
| D630029K05Rik | 107.04 | 63.49 | 40.68 | 0.00011675 | RIKEN cDNA D630029K05 gene |
| Slco4c1 | 5.93 | 3.52 | 40.66 | 0.00064720 | solute carrier organic anion transporter family, member 4C1 |
| Btg1 | 2.98 | 1.77 | 40.46 | 0.00051425 | B-cell translocation gene 1, anti-proliferative |
| Olfml3 | 6.36 | 3.79 | 40.31 | 0.00171137 | olfactomedin-like 3 |
| Cpm | 3.50 | 2.09 | 40.12 | 0.00034923 | carboxypeptidase M |
| 2210404J11Rik | 7.04 | 4.22 | 40.02 | 0.00050968 | RIKEN cDNA 2210404J11 gene |
| BC024386 | 121.28 | 73.18 | 39.66 | 0.00014150 | cDNA sequence BC023486 |
| Mogat2 | 27.55 | 16.63 | 39.66 | 0.00009190 | monoacylglycerol O-acyltransferase 2 |
| Slc22a29 | 9.39 | 5.68 | 39.51 | 0.00042560 | Solute carrier family 22. member 29 |
| Xrn2 | 18.85 | 11.43 | 39.38 | 0.00007380 | 5'-3' exoribonuclease 2 |
| Vdr | 18.94 | 11.52 | 39.16 | 0.00008740 | vitamin D receptor |
| Slc41a2 | 2.76 | 1.68 | 39.09 | 0.00156018 | solute carrier family 41, member 2 |
| Slc5a12 | 86.49 | 52.87 | 38.87 | 0.00102359 | solute carrier family 5 (sodium/glucose cotransporter), member 12 |
| Sucnr1 | 17.67 | 10.81 | 38.81 | 0.00026427 | succinate receptor 1 |
| Slc8a1 | 1.16 | 0.71 | 38.72 | 0.00041632 | solute carrier family 8 (sodium/calcium exchanger), member 1 |
| C8g | 18.99 | 11.66 | 38.60 | 0.00073360 | complement component 8, gamma polypeptide |
| Tgfbr3 | 5.48 | 3.37 | 38.47 | 0.00020001 | transforming growth factor, beta receptor III |

**Suppl. Table S11** Functional annotation terms associated with genes exhibiting DYSregulation in diabetes relative to control (n = 474; ranked by ascending p-value)

| **Category** | **Term** | **PValue** | **Bonferroni** | **Fold Enrichment** |
| --- | --- | --- | --- | --- |
| SP_PIR_KEYWORDS | endoplasmic reticulum | 8.13E-20 | 3.38E-17 | 3.35 |
| GOTERM_CC_FAT | GO:0005783~endoplasmic reticulum | 1.29E-17 | 3.66E-15 | 2.79 |
| SP_PIR_KEYWORDS | stress response | 1.88E-14 | 7.85E-12 | 12.29 |
| SP_PIR_KEYWORDS | oxidoreductase | 1.55E-13 | 6.46E-11 | 3.06 |
| GOTERM_CC_FAT | GO:0044432~endoplasmic reticulum part | 1.75E-13 | 4.97E-11 | 4.45 |
| GOTERM_BP_FAT | GO:0055114~oxidation reduction | 2.62E-12 | 5.14E-09 | 2.70 |
| GOTERM_CC_FAT | GO:0042598~vesicular fraction | 1.82E-10 | 5.16E-08 | 4.39 |
| GOTERM_CC_FAT | GO:0005792~microsome | 2.20E-09 | 6.26E-07 | 4.22 |
| SP_PIR_KEYWORDS | Chaperone | 3.31E-09 | 1.38E-06 | 4.71 |
| KEGG_PATHWAY | mmu00982:Drug metabolism | 4.86E-09 | 7.05E-07 | 6.34 |
| SP_PIR_KEYWORDS | nadp | 6.47E-09 | 2.69E-06 | 4.76 |
| GOTERM_CC_FAT | GO:0005788~endoplasmic reticulum lumen | 2.07E-08 | 5.89E-06 | 6.34 |
| SP_PIR_KEYWORDS | microsome | 2.16E-08 | 8.97E-06 | 5.53 |
| GOTERM_BP_FAT | GO:0006766~vitamin metabolic process | 5.23E-08 | 1.02E-04 | 6.48 |
| GOTERM_CC_FAT | GO:0042175~nuclear envelope-endoplasmic reticulum network | 1.73E-07 | 4.91E-05 | 3.93 |
| GOTERM_CC_FAT | GO:0005789~endoplasmic reticulum membrane | 3.27E-07 | 9.28E-05 | 3.94 |
| GOTERM_CC_FAT | GO:0000267~cell fraction | 3.45E-07 | 9.79E-05 | 2.25 |
| SP_PIR_KEYWORDS | peroxisome | 4.55E-07 | 1.89E-04 | 5.12 |
| GOTERM_CC_FAT | GO:0005626~insoluble fraction | 5.20E-07 | 1.48E-04 | 2.32 |
| GOTERM_CC_FAT | GO:0005624~membrane fraction | 5.44E-07 | 1.55E-04 | 2.35 |
| GOTERM_CC_FAT | GO:0042579~microbody | 5.93E-07 | 1.68E-04 | 4.67 |
| GOTERM_CC_FAT | GO:0005777~peroxisome | 5.93E-07 | 1.68E-04 | 4.67 |
| UP_SEQ_FEATURE | short sequence motif:Prevents secretion from ER | 8.42E-07 | 0.001241455 | 6.21 |
| GOTERM_BP_FAT | GO:0006575~cellular amino acid derivative metabolic process | 1.10E-06 | 0.002144072 | 4.02 |
| GOTERM_BP_FAT | GO:0006457~protein folding | 1.11E-06 | 0.002179077 | 4.22 |
| GOTERM_BP_FAT | GO:0006775~fat-soluble vitamin metabolic process | 1.22E-06 | 0.002379817 | 8.76 |
| GOTERM_CC_FAT | GO:0042470~melanosome | 1.29E-06 | 3.68E-04 | 5.04 |
| GOTERM_CC_FAT | GO:0048770~pigment granule | 1.29E-06 | 3.68E-04 | 5.04 |
| INTERPRO | IPR012335:Thioredoxin fold | 1.44E-06 | 0.001498747 | 4.70 |
| GOTERM_MF_FAT | GO:0016765~transferase activity, transferring alkyl or aryl (other than methyl) groups | 1.46E-06 | 9.92E-04 | 6.57 |
| SP_PIR_KEYWORDS | Monooxygenase | 2.13E-06 | 8.85E-04 | 4.55 |
| SP_PIR_KEYWORDS | lyase | 2.13E-06 | 8.85E-04 | 4.28 |
| SP_PIR_KEYWORDS | acetylation | 3.23E-06 | 0.001342761 | 1.52 |
| GOTERM_BP_FAT | GO:0008610~lipid biosynthetic process | 3.66E-06 | 0.007144251 | 2.82 |
| SP_PIR_KEYWORDS | heme | 3.69E-06 | 0.00153353 | 3.90 |
| KEGG_PATHWAY | mmu00830:Retinol metabolism | 3.72E-06 | 5.40E-04 | 5.35 |
| SP_PIR_KEYWORDS | lipid synthesis | 3.73E-06 | 0.001551802 | 4.66 |
| INTERPRO | IPR001128:Cytochrome P450 | 3.88E-06 | 0.004038662 | 5.01 |
| GOTERM_BP_FAT | GO:0019725~cellular homeostasis | 4.61E-06 | 0.008978959 | 2.61 |
| INTERPRO | IPR017972:Cytochrome P450, conserved site | 5.02E-06 | 0.005230376 | 4.89 |
| GOTERM_MF_FAT | GO:0016862~intramolecular oxidoreductase activity, interconverting keto- and enol-groups | 5.29E-06 | 0.003596644 | 19.34 |
| SP_PIR_KEYWORDS | membrane | 6.63E-06 | 0.002752873 | 1.28 |
| INTERPRO | IPR000886:Endoplasmic reticulum, targeting sequence | 7.20E-06 | 0.007493231 | 7.23 |
| INTERPRO | IPR017973:Cytochrome P450, C-terminal region | 9.97E-06 | 0.01035736 | 4.99 |
| INTERPRO | IPR002401:Cytochrome P450, E-class, group I | 1.31E-05 | 0.013595744 | 5.33 |
| INTERPRO | IPR001023:Heat shock protein Hsp70 | 1.32E-05 | 0.013641846 | 16.97 |
| INTERPRO | IPR013126:Heat shock protein 70 | 1.32E-05 | 0.013641846 | 16.97 |
| GOTERM_BP_FAT | GO:0042592~homeostatic process | 1.39E-05 | 0.026920803 | 2.09 |
| INTERPRO | IPR003080:Glutathione S-transferase, alpha class | 1.50E-05 | 0.015487746 | 25.92 |
| UP_SEQ_FEATURE | short sequence motif:Microbody targeting signal | 1.91E-05 | 0.027856231 | 6.41 |
| GOTERM_MF_FAT | GO:0004364~glutathione transferase activity | 2.03E-05 | 0.013700251 | 8.93 |
| GOTERM_MF_FAT | GO:0005506~iron ion binding | 2.09E-05 | 0.014136579 | 2.45 |
| GOTERM_MF_FAT | GO:0009055~electron carrier activity | 2.17E-05 | 0.014644904 | 3.02 |
| GOTERM_BP_FAT | GO:0006720~isoprenoid metabolic process | 3.05E-05 | 0.057985259 | 6.08 |
| INTERPRO | IPR018181:Heat shock protein 70, conserved site | 3.47E-05 | 0.035621719 | 14.36 |
| GOTERM_MF_FAT | GO:0020037~heme binding | 3.55E-05 | 0.023875914 | 3.43 |
| GOTERM_BP_FAT | GO:0045454~cell redox homeostasis | 3.62E-05 | 0.06844921 | 5.29 |
| SP_PIR_KEYWORDS | signal | 4.38E-05 | 0.018064095 | 1.39 |
| UP_SEQ_FEATURE | nucleotide phosphate-binding region:NADP | 5.05E-05 | 0.071797687 | 5.09 |
| SP_PIR_KEYWORDS | molecular chaperone | 5.58E-05 | 0.022945458 | 13.17 |
| GOTERM_CC_FAT | GO:0031090~organelle membrane | 5.63E-05 | 0.015871988 | 1.80 |
| GOTERM_MF_FAT | GO:0046906~tetrapyrrole binding | 6.34E-05 | 0.042275034 | 3.27 |
| GOTERM_BP_FAT | GO:0006576~biogenic amine metabolic process | 6.85E-05 | 0.125571868 | 4.47 |
| INTERPRO | IPR017933:Glutathione S-transferase/chloride channel, C-terminal | 8.54E-05 | 0.085334144 | 7.32 |
| GOTERM_MF_FAT | GO:0003756~protein disulfide isomerase activity | 8.66E-05 | 0.057261066 | 18.13 |
| GOTERM_MF_FAT | GO:0016864~intramolecular oxidoreductase activity, transposing S-S bonds | 8.66E-05 | 0.057261066 | 18.13 |
| GOTERM_BP_FAT | GO:0019748~secondary metabolic process | 9.29E-05 | 0.16634458 | 4.75 |
| KEGG_PATHWAY | mmu03320:PPAR signaling pathway | 9.48E-05 | 0.01364933 | 4.25 |
| KEGG_PATHWAY | mmu00980:Metabolism of xenobiotics by cytochrome P450 | 9.58E-05 | 0.013791224 | 4.67 |
| GOTERM_BP_FAT | GO:0010817~regulation of hormone levels | 9.71E-05 | 0.173110483 | 3.49 |
| GOTERM_BP_FAT | GO:0006694~steroid biosynthetic process | 1.19E-04 | 0.207646352 | 4.62 |
| COG_ONTOLOGY | Secondary metabolites biosynthesis, transport, and catabolism | 1.26E-04 | 0.002518607 | 3.40 |
| GOTERM_CC_FAT | GO:0012505~endomembrane system | 1.27E-04 | 0.035473942 | 1.97 |
| SP_PIR_KEYWORDS | Flavoprotein | 1.28E-04 | 0.051720489 | 3.88 |
| GOTERM_BP_FAT | GO:0051186~cofactor metabolic process | 1.32E-04 | 0.228203914 | 2.95 |
| SP_PIR_KEYWORDS | heat shock | 1.33E-04 | 0.05388314 | 30.73 |
| SP_PIR_KEYWORDS | stress-induced protein | 1.33E-04 | 0.05388314 | 30.73 |
| SP_PIR_KEYWORDS | lipid metabolism | 1.39E-04 | 0.05623953 | 3.39 |
| SP_PIR_KEYWORDS | iron | 1.39E-04 | 0.056350511 | 2.39 |
| SMART | SM00387:HATPase_c | 1.40E-04 | 0.025231211 | 11.27 |
| INTERPRO | IPR013766:Thioredoxin domain | 1.41E-04 | 0.137337573 | 8.38 |
| INTERPRO | IPR004045:Glutathione S-transferase, N-terminal | 1.77E-04 | 0.168755702 | 8.07 |
| UP_SEQ_FEATURE | metal ion-binding site:Iron (heme axial ligand) | 1.92E-04 | 0.246884252 | 3.70 |
| GOTERM_BP_FAT | GO:0042445~hormone metabolic process | 2.02E-04 | 0.326122683 | 3.97 |
| INTERPRO | IPR004046:Glutathione S-transferase, C-terminal | 2.19E-04 | 0.204605331 | 7.78 |
| SP_PIR_KEYWORDS | FAD | 2.19E-04 | 0.087217504 | 3.67 |
| SP_PIR_KEYWORDS | Redox-active center | 2.29E-04 | 0.090959842 | 6.30 |
| GOTERM_BP_FAT | GO:0016053~organic acid biosynthetic process | 2.72E-04 | 0.412704641 | 3.17 |
| GOTERM_BP_FAT | GO:0046394~carboxylic acid biosynthetic process | 2.72E-04 | 0.412704641 | 3.17 |
| KEGG_PATHWAY | mmu04610:Complement and coagulation cascades | 2.85E-04 | 0.040446219 | 4.11 |
| GOTERM_BP_FAT | GO:0006732~coenzyme metabolic process | 3.15E-04 | 0.459892267 | 3.13 |
| UP_SEQ_FEATURE | binding site:Substrate | 3.26E-04 | 0.382183825 | 2.36 |
| INTERPRO | IPR010987:Glutathione S-transferase, C-terminal-like | 3.27E-04 | 0.289423415 | 7.26 |
| GOTERM_BP_FAT | GO:0006721~terpenoid metabolic process | 3.36E-04 | 0.482352646 | 7.19 |
| INTERPRO | IPR003594:ATP-binding region, ATPase-like | 3.47E-04 | 0.304174243 | 9.33 |
| UP_SEQ_FEATURE | topological domain:Lumenal | 3.85E-04 | 0.433356979 | 2.04 |
| SP_PIR_KEYWORDS | Isomerase | 4.59E-04 | 0.173771151 | 3.39 |
| GOTERM_BP_FAT | GO:0006631~fatty acid metabolic process | 4.65E-04 | 0.597571494 | 2.75 |
| PIR_SUPERFAMILY | PIRSF000503:glutathione transferase | 5.08E-04 | 0.15098203 | 8.51 |
| GOTERM_BP_FAT | GO:0030003~cellular cation homeostasis | 5.86E-04 | 0.682496522 | 2.94 |
| INTERPRO | IPR005788:Disulphide isomerase | 6.11E-04 | 0.471944823 | 20.74 |
| GOTERM_MF_FAT | GO:0051082~unfolded protein binding | 6.28E-04 | 0.348180498 | 4.14 |
| GOTERM_MF_FAT | GO:0016712~oxidoreductase activity, acting on paired donors, with incorporation or reduction of molecular oxygen, reduced flavin or flavoprotein as one donor, and incorporation of one atom of oxygen | 6.91E-04 | 0.375566655 | 5.28 |
| SMART | SM00028:TPR | 7.52E-04 | 0.127973775 | 3.69 |
| GOTERM_BP_FAT | GO:0046700~heterocycle catabolic process | 7.78E-04 | 0.782315809 | 5.18 |
| GOTERM_BP_FAT | GO:0006695~cholesterol biosynthetic process | 8.41E-04 | 0.80730152 | 7.77 |
| SP_PIR_KEYWORDS | glycoprotein | 8.60E-04 | 0.300825491 | 1.27 |
| GOTERM_BP_FAT | GO:0008202~steroid metabolic process | 0.001034153 | 0.868127492 | 2.78 |
| GOTERM_CC_FAT | GO:0005739~mitochondrion | 0.0011218 | 0.272957305 | 1.47 |
| INTERPRO | IPR016040:NAD(P)-binding domain | 0.001175635 | 0.707147219 | 2.88 |
| GOTERM_BP_FAT | GO:0048878~chemical homeostasis | 0.001306922 | 0.922743738 | 2.04 |
| GOTERM_BP_FAT | GO:0006776~vitamin A metabolic process | 0.001512877 | 0.948411462 | 6.88 |
| UP_SEQ_FEATURE | signal peptide | 0.001540959 | 0.897327401 | 1.28 |
| GOTERM_BP_FAT | GO:0034754~cellular hormone metabolic process | 0.00163766 | 0.959609713 | 4.58 |
| SP_PIR_KEYWORDS | lipid binding | 0.001690338 | 0.505286079 | 15.36 |
| GOTERM_MF_FAT | GO:0016860~intramolecular oxidoreductase activity | 0.001735011 | 0.693509554 | 5.34 |
| GOTERM_CC_FAT | GO:0031300~intrinsic to organelle membrane | 0.001841153 | 0.407479205 | 3.06 |
| GOTERM_BP_FAT | GO:0006875~cellular metal ion homeostasis | 0.001883384 | 0.975056534 | 3.28 |
| INTERPRO | IPR002347:Glucose/ribitol dehydrogenase | 0.002004381 | 0.876889655 | 4.44 |
| GOTERM_BP_FAT | GO:0006986~response to unfolded protein | 0.00204714 | 0.981910229 | 4.41 |
| GOTERM_BP_FAT | GO:0042573~retinoic acid metabolic process | 0.002086816 | 0.983265038 | 8.76 |
| GOTERM_MF_FAT | GO:0048037~cofactor binding | 0.002111296 | 0.762909374 | 2.31 |
| GOTERM_BP_FAT | GO:0001523~retinoid metabolic process | 0.002139348 | 0.984904073 | 6.39 |
| GOTERM_BP_FAT | GO:0016101~diterpenoid metabolic process | 0.002139348 | 0.984904073 | 6.39 |
| UP_SEQ_FEATURE | domain:Thioredoxin 1 | 0.002161436 | 0.958981503 | 14.10 |
| UP_SEQ_FEATURE | domain:Thioredoxin 2 | 0.002161436 | 0.958981503 | 14.10 |
| INTERPRO | IPR011701:Major facilitator superfamily MFS-1 | 0.002201685 | 0.899850718 | 3.50 |
| KEGG_PATHWAY | mmu00480:Glutathione metabolism | 0.002224168 | 0.275927393 | 4.31 |
| SP_PIR_KEYWORDS | Cholesterol biosynthesis | 0.002345578 | 0.623526041 | 8.54 |
| PIR_SUPERFAMILY | PIRSF002581:chaperone HSP70 | 0.002444479 | 0.545285671 | 13.47 |
| GOTERM_BP_FAT | GO:0051789~response to protein stimulus | 0.002483723 | 0.992320327 | 3.43 |
| GOTERM_BP_FAT | GO:0009636~response to toxin | 0.002514996 | 0.992777581 | 6.17 |
| KEGG_PATHWAY | mmu00380:Tryptophan metabolism | 0.00262066 | 0.316476716 | 4.90 |
| INTERPRO | IPR018170:Aldo/keto reductase, conserved site | 0.002769553 | 0.944724483 | 8.19 |
| GOTERM_BP_FAT | GO:0055065~metal ion homeostasis | 0.002902948 | 0.996627961 | 3.09 |
| GOTERM_BP_FAT | GO:0016126~sterol biosynthetic process | 0.002936286 | 0.996841651 | 5.96 |
| GOTERM_MF_FAT | GO:0019842~vitamin binding | 0.003007183 | 0.871390834 | 2.88 |
| GOTERM_BP_FAT | GO:0006873~cellular ion homeostasis | 0.003072644 | 0.997583653 | 2.17 |
| INTERPRO | IPR019734:Tetratricopeptide repeat | 0.003233148 | 0.965982315 | 3.06 |
| GOTERM_BP_FAT | GO:0018401~peptidyl-proline hydroxylation to 4-hydroxy-L-proline | 0.003282061 | 0.998398548 | 29.80 |
| GOTERM_BP_FAT | GO:0019471~4-hydroxyproline metabolic process | 0.003282061 | 0.998398548 | 29.80 |
| SP_PIR_KEYWORDS | chylomicron | 0.003450202 | 0.76254232 | 12.29 |
| SP_PIR_KEYWORDS | carboxypeptidase | 0.003462495 | 0.763757677 | 5.76 |
| GOTERM_CC_FAT | GO:0031410~cytoplasmic vesicle | 0.003559115 | 0.636723712 | 1.74 |
| GOTERM_BP_FAT | GO:0055080~cation homeostasis | 0.003609247 | 0.999157966 | 2.43 |
| GOTERM_BP_FAT | GO:0042572~retinol metabolic process | 0.003754397 | 0.999366939 | 11.92 |
| INTERPRO | IPR011497:Protease inhibitor, Kazal-type | 0.003770809 | 0.98063263 | 5.66 |
| GOTERM_CC_FAT | GO:0031301~integral to organelle membrane | 0.003839879 | 0.664664864 | 3.21 |
| GOTERM_CC_FAT | GO:0016323~basolateral plasma membrane | 0.003886978 | 0.669137596 | 2.63 |
| GOTERM_BP_FAT | GO:0050818~regulation of coagulation | 0.003923611 | 0.999546058 | 7.45 |
| GOTERM_BP_FAT | GO:0055082~cellular chemical homeostasis | 0.004059223 | 0.999652287 | 2.11 |
| INTERPRO | IPR002198:Short-chain dehydrogenase/reductase SDR | 0.004310949 | 0.989005493 | 3.89 |
| INTERPRO | IPR006662:Thioredoxin-like subdomain | 0.004473225 | 0.990725839 | 11.31 |
| GOTERM_CC_FAT | GO:0031982~vesicle | 0.004771513 | 0.742916155 | 1.71 |
| GOTERM_BP_FAT | GO:0033865~nucleoside bisphosphate metabolic process | 0.005034989 | 0.999948986 | 10.84 |
| SP_PIR_KEYWORDS | unfolded protein response | 0.005059376 | 0.878767209 | 6.98 |
| GOTERM_BP_FAT | GO:0030005~cellular di-, tri-valent inorganic cation homeostasis | 0.005367346 | 0.999973479 | 2.67 |
| UP_SEQ_FEATURE | domain:GST C-terminal | 0.00569855 | 0.999782892 | 5.13 |
| GOTERM_CC_FAT | GO:0016023~cytoplasmic membrane-bounded vesicle | 0.00570386 | 0.802996502 | 1.79 |
| INTERPRO | IPR001404:Heat shock protein Hsp90 | 0.005907951 | 0.997942354 | 23.33 |
| INTERPRO | IPR019805:Heat shock protein Hsp90, conserved site | 0.005907951 | 0.997942354 | 23.33 |
| INTERPRO | IPR005792:Protein disulphide isomerase | 0.005907951 | 0.997942354 | 23.33 |
| UP_SEQ_FEATURE | transmembrane region | 0.006270977 | 0.999907204 | 1.19 |
| SP_PIR_KEYWORDS | mitochondrion | 0.006329723 | 0.928748091 | 1.56 |
| GOTERM_BP_FAT | GO:0042359~vitamin D metabolic process | 0.006418616 | 0.999996655 | 22.35 |
| GOTERM_BP_FAT | GO:0018208~peptidyl-proline modification | 0.006418616 | 0.999996655 | 22.35 |
| GOTERM_MF_FAT | GO:0004180~carboxypeptidase activity | 0.006519016 | 0.988367859 | 4.97 |
| GOTERM_MF_FAT | GO:0004740~pyruvate dehydrogenase (acetyl-transferring) kinase activity | 0.006762607 | 0.990156832 | 21.76 |
| GOTERM_MF_FAT | GO:0004656~procollagen-proline 4-dioxygenase activity | 0.006762607 | 0.990156832 | 21.76 |
| GOTERM_MF_FAT | GO:0031545~peptidyl-proline 4-dioxygenase activity | 0.006762607 | 0.990156832 | 21.76 |
| GOTERM_CC_FAT | GO:0031988~membrane-bounded vesicle | 0.006814797 | 0.856587066 | 1.77 |
| UP_SEQ_FEATURE | domain:GST N-terminal | 0.006833928 | 0.999959794 | 6.41 |
| SP_PIR_KEYWORDS | plasma | 0.006982486 | 0.945790688 | 6.40 |
| SP_PIR_KEYWORDS | sterol biosynthesis | 0.006982486 | 0.945790688 | 6.40 |
| INTERPRO | IPR017937:Thioredoxin, conserved site | 0.007764695 | 0.999707772 | 6.22 |
| PIR_SUPERFAMILY | PIRSF002583:Hsp90 | 0.007790275 | 0.919402732 | 20.21 |
| GOTERM_BP_FAT | GO:0030433~ER-associated protein catabolic process | 0.008303273 | 0.999999919 | 9.17 |
| SP_PIR_KEYWORDS | transport | 0.00885712 | 0.975300594 | 1.35 |
| INTERPRO | IPR011990:Tetratricopeptide-like helical | 0.009094935 | 0.999927979 | 2.49 |
| SP_PIR_KEYWORDS | Steroid biosynthesis | 0.009150288 | 0.978160652 | 4.61 |
| GOTERM_CC_FAT | GO:0031227~intrinsic to endoplasmic reticulum membrane | 0.009204738 | 0.927650822 | 3.84 |
| SP_PIR_KEYWORDS | nad | 0.009269389 | 0.979225906 | 2.36 |
| GOTERM_MF_FAT | GO:0050662~coenzyme binding | 0.009270142 | 0.998239945 | 2.36 |
| GOTERM_BP_FAT | GO:0015711~organic anion transport | 0.009277136 | 0.999999988 | 4.58 |
| SP_PIR_KEYWORDS | metalloprotein | 0.009391061 | 0.980260643 | 3.84 |
| SP_PIR_KEYWORDS | carbohydrate metabolism | 0.00942594 | 0.980547668 | 3.37 |
| INTERPRO | IPR011893:SelT/selW/selH selenoprotein | 0.009637783 | 0.999959355 | 18.67 |
| INTERPRO | IPR005467:Signal transduction histidine kinase, core | 0.009637783 | 0.999959355 | 18.67 |
| INTERPRO | IPR018955:Branched-chain alpha-ketoacid dehydrogenase kinase/Pyruvate dehydrogenase kinase, mitochondrial | 0.009637783 | 0.999959355 | 18.67 |
| SP_PIR_KEYWORDS | transmembrane protein | 0.009794145 | 0.983335058 | 1.83 |
| GOTERM_BP_FAT | GO:0050801~ion homeostasis | 0.009962031 | 0.999999997 | 1.93 |
| GOTERM_BP_FAT | GO:0055066~di-, tri-valent inorganic cation homeostasis | 0.010021392 | 0.999999997 | 2.45 |
| GOTERM_MF_FAT | GO:0008238~exopeptidase activity | 0.010081157 | 0.998992323 | 3.32 |
| GOTERM_BP_FAT | GO:0030193~regulation of blood coagulation | 0.010308558 | 0.999999998 | 8.51 |
| GOTERM_BP_FAT | GO:0018106~peptidyl-histidine phosphorylation | 0.010461226 | 0.999999999 | 17.88 |
| SP_PIR_KEYWORDS | lipoprotein | 0.010527867 | 0.987757108 | 1.62 |
| KEGG_PATHWAY | mmu00330:Arginine and proline metabolism | 0.010701003 | 0.789865457 | 3.70 |
| KEGG_PATHWAY | mmu00340:Histidine metabolism | 0.010913428 | 0.796307827 | 5.60 |
| UP_SEQ_FEATURE | active site:Charge relay system | 0.010944393 | 0.999999912 | 2.14 |
| GOTERM_BP_FAT | GO:0009309~amine biosynthetic process | 0.010962845 | 1 | 3.27 |
| INTERPRO | IPR013026:Tetratricopeptide region | 0.01115027 | 0.999991758 | 2.73 |
| INTERPRO | IPR004156:Organic anion transporter polypeptide OATP | 0.011212606 | 0.999992283 | 8.30 |
| INTERPRO | IPR001440:Tetratricopeptide TPR-1 | 0.011319284 | 0.999993105 | 2.95 |
| UP_SEQ_FEATURE | domain:Kazal-like | 0.011626231 | 0.999999968 | 4.34 |
| UP_SEQ_FEATURE | domain:Histidine kinase | 0.011644984 | 0.999999969 | 16.92 |
| KEGG_PATHWAY | mmu00900:Terpenoid backbone biosynthesis | 0.012060472 | 0.827851785 | 8.00 |
| PIR_SUPERFAMILY | PIRSF000718:branched-chain alpha-keto acid dehydrogenase kinase | 0.012667555 | 0.983510114 | 16.16 |
| GOTERM_MF_FAT | GO:0016831~carboxy-lyase activity | 0.012937874 | 0.999859202 | 5.37 |
| SMART | SM00121:IB | 0.013015997 | 0.907861596 | 7.91 |
| GOTERM_BP_FAT | GO:0044271~nitrogen compound biosynthetic process | 0.013507612 | 1 | 1.87 |
| COG_ONTOLOGY | Amino acid transport and metabolism | 0.013631417 | 0.2400507 | 3.43 |
| SP_PIR_KEYWORDS | transferase | 0.013741103 | 0.996836057 | 1.35 |
| SP_PIR_KEYWORDS | fatty acid metabolism | 0.014030265 | 0.997199401 | 3.53 |
| INTERPRO | IPR008067:Cytochrome P450, E-class, group I, CYP2A-like | 0.014150947 | 0.999999655 | 15.55 |
| KEGG_PATHWAY | mmu01040:Biosynthesis of unsaturated fatty acids | 0.01432768 | 0.876626656 | 5.18 |
| GOTERM_BP_FAT | GO:0016042~lipid catabolic process | 0.014609685 | 1 | 2.45 |
| KEGG_PATHWAY | mmu00120:Primary bile acid biosynthesis | 0.014686815 | 0.882976604 | 7.46 |
| GOTERM_BP_FAT | GO:0009110~vitamin biosynthetic process | 0.01518079 | 1 | 5.14 |
| GOTERM_BP_FAT | GO:0014812~muscle cell migration | 0.015345959 | 1 | 14.90 |
| GOTERM_MF_FAT | GO:0019904~protein domain specific binding | 0.015458078 | 0.999975311 | 2.12 |
| GOTERM_MF_FAT | GO:0008035~high-density lipoprotein binding | 0.016148766 | 0.999984691 | 14.51 |
| UP_SEQ_FEATURE | nucleotide phosphate-binding region:FAD | 0.016319972 | 1 | 3.40 |
| SP_PIR_KEYWORDS | duplication | 0.016365001 | 0.998955441 | 2.56 |
| SP_PIR_KEYWORDS | glucose metabolism | 0.01651264 | 0.999018673 | 7.23 |
| SP_PIR_KEYWORDS | hdl | 0.01651264 | 0.999018673 | 7.23 |
| GOTERM_MF_FAT | GO:0016229~steroid dehydrogenase activity | 0.016592965 | 0.999988744 | 5.00 |
| GOTERM_BP_FAT | GO:0051338~regulation of transferase activity | 0.016598505 | 1 | 2.10 |
| COG_ONTOLOGY | Posttranslational modification, protein turnover, chaperones | 0.017256201 | 0.293997494 | 2.29 |
| UP_SEQ_FEATURE | glycosylation site:N-linked (GlcNAc...) | 0.017340783 | 1 | 1.18 |
| GOTERM_CC_FAT | GO:0005829~cytosol | 0.018012528 | 0.994270866 | 1.56 |
| PIR_SUPERFAMILY | PIRSF000126:11beta-hydroxysteroid dehydrogenase | 0.018539965 | 0.99758463 | 13.47 |
| PIR_SUPERFAMILY | PIRSF001487:protein disulfide-isomerase | 0.018539965 | 0.99758463 | 13.47 |
| GOTERM_BP_FAT | GO:0046395~carboxylic acid catabolic process | 0.018664207 | 1 | 2.94 |
| GOTERM_BP_FAT | GO:0016054~organic acid catabolic process | 0.018664207 | 1 | 2.94 |
| SP_PIR_KEYWORDS | isopeptide bond | 0.019269397 | 0.999694728 | 1.89 |
| GOTERM_BP_FAT | GO:0043086~negative regulation of catalytic activity | 0.019960054 | 1 | 2.66 |
| KEGG_PATHWAY | mmu00860:Porphyrin and chlorophyll metabolism | 0.02060092 | 0.95111729 | 4.67 |
| KEGG_PATHWAY | mmu00071:Fatty acid metabolism | 0.020824819 | 0.952711279 | 3.73 |
| GOTERM_CC_FAT | GO:0045177~apical part of cell | 0.020911943 | 0.997526124 | 2.31 |
| GOTERM_BP_FAT | GO:0042219~cellular amino acid derivative catabolic process | 0.020933173 | 1 | 6.62 |
| GOTERM_MF_FAT | GO:0004857~enzyme inhibitor activity | 0.020967513 | 0.999999459 | 1.91 |
| GOTERM_BP_FAT | GO:0018202~peptidyl-histidine modification | 0.021012135 | 1 | 12.77 |
| GOTERM_BP_FAT | GO:0006699~bile acid biosynthetic process | 0.021012135 | 1 | 12.77 |
| GOTERM_BP_FAT | GO:0009408~response to heat | 0.021253737 | 1 | 4.66 |
| GOTERM_BP_FAT | GO:0060191~regulation of lipase activity | 0.021253737 | 1 | 4.66 |
| GOTERM_BP_FAT | GO:0051605~protein maturation by peptide bond cleavage | 0.021329287 | 1 | 3.21 |
| INTERPRO | IPR000867:Insulin-like growth factor-binding protein, IGFBP | 0.021720288 | 1 | 6.55 |
| GOTERM_MF_FAT | GO:0031543~peptidyl-proline dioxygenase activity | 0.02209806 | 0.999999754 | 12.43 |
| GOTERM_MF_FAT | GO:0019798~procollagen-proline dioxygenase activity | 0.02209806 | 0.999999754 | 12.43 |
| GOTERM_BP_FAT | GO:0032101~regulation of response to external stimulus | 0.02218062 | 1 | 2.60 |
| GOTERM_BP_FAT | GO:0043405~regulation of MAP kinase activity | 0.0223434 | 1 | 2.84 |
| GOTERM_CC_FAT | GO:0016324~apical plasma membrane | 0.022445025 | 0.998414749 | 2.60 |
| GOTERM_BP_FAT | GO:0045859~regulation of protein kinase activity | 0.022749128 | 1 | 2.08 |
| UP_SEQ_FEATURE | domain:IGFBP N-terminal | 0.024212617 | 1 | 6.27 |
| SP_PIR_KEYWORDS | disulfide bond | 0.025635805 | 0.999979673 | 1.22 |
| SP_PIR_KEYWORDS | dimer | 0.025902962 | 0.999981865 | 11.52 |
| GOTERM_BP_FAT | GO:0006767~water-soluble vitamin metabolic process | 0.026020728 | 1 | 4.38 |
| BIOCARTA | m_eea1Pathway:The role of FYVE-finger proteins in vesicle transport | 0.02682858 | 0.886460316 | 10.84 |
| GOTERM_CC_FAT | GO:0030176~integral to endoplasmic reticulum membrane | 0.027035373 | 0.99958355 | 4.33 |
| GOTERM_BP_FAT | GO:0030002~cellular anion homeostasis | 0.027402176 | 1 | 11.17 |
| GOTERM_BP_FAT | GO:0055081~anion homeostasis | 0.027402176 | 1 | 11.17 |
| INTERPRO | IPR017936:Thioredoxin-like | 0.027444706 | 1 | 4.32 |
| KEGG_PATHWAY | mmu00590:Arachidonic acid metabolism | 0.027470314 | 0.982383659 | 2.70 |
| GOTERM_CC_FAT | GO:0044429~mitochondrial part | 0.027789351 | 0.999665846 | 1.53 |
| GOTERM_CC_FAT | GO:0031980~mitochondrial lumen | 0.027924901 | 0.99967882 | 2.10 |
| GOTERM_CC_FAT | GO:0005759~mitochondrial matrix | 0.027924901 | 0.99967882 | 2.10 |
| GOTERM_BP_FAT | GO:0010876~lipid localization | 0.028082586 | 1 | 2.33 |
| GOTERM_BP_FAT | GO:0043549~regulation of kinase activity | 0.028230712 | 1 | 2.02 |
| GOTERM_MF_FAT | GO:0070330~aromatase activity | 0.028343422 | 0.999999997 | 4.27 |
| INTERPRO | IPR014716:Fibrinogen, alpha/beta/gamma chain, C-terminal globular, subdomain 1 | 0.028439814 | 1 | 5.93 |
| SMART | SM00327:VWA | 0.029065565 | 0.995338105 | 2.99 |
| GOTERM_BP_FAT | GO:0046486~glycerolipid metabolic process | 0.02933359 | 1 | 2.31 |
| SP_PIR_KEYWORDS | decarboxylase | 0.029352888 | 0.999995855 | 5.85 |
| GOTERM_BP_FAT | GO:0008203~cholesterol metabolic process | 0.029497263 | 1 | 2.98 |
| GOTERM_BP_FAT | GO:0006641~triglyceride metabolic process | 0.03137977 | 1 | 4.14 |
| INTERPRO | IPR016662:Acyl-CoA thioesterase, long chain | 0.031864175 | 1 | 10.37 |
| INTERPRO | IPR006862:Acyl-CoA thioester hydrolase/bile acid-CoA amino acid N-acetyltransferase | 0.031864175 | 1 | 10.37 |
| INTERPRO | IPR002939:Chaperone DnaJ, C-terminal | 0.031864175 | 1 | 10.37 |
| INTERPRO | IPR014940:BAAT/Acyl-CoA thioester hydrolase C-terminal | 0.031864175 | 1 | 10.37 |
| INTERPRO | IPR001395:Aldo/keto reductase | 0.032165502 | 1 | 5.66 |
| GOTERM_BP_FAT | GO:0051336~regulation of hydrolase activity | 0.032381301 | 1 | 1.98 |
| GOTERM_MF_FAT | GO:0004091~carboxylesterase activity | 0.032625222 | 1 | 2.42 |
| GOTERM_CC_FAT | GO:0005903~brush border | 0.032770733 | 0.999922311 | 4.08 |
| GOTERM_BP_FAT | GO:0006874~cellular calcium ion homeostasis | 0.032810551 | 1 | 2.62 |
| SP_PIR_KEYWORDS | acetylated amino end | 0.033188706 | 0.999999202 | 5.59 |
| GOTERM_BP_FAT | GO:0044092~negative regulation of molecular function | 0.033319702 | 1 | 2.26 |
| GOTERM_BP_FAT | GO:0048732~gland development | 0.033483873 | 1 | 1.97 |
| GOTERM_MF_FAT | GO:0050661~NADP or NADPH binding | 0.033927302 | 1 | 5.53 |
| GOTERM_BP_FAT | GO:0006706~steroid catabolic process | 0.03446147 | 1 | 9.93 |
| GOTERM_BP_FAT | GO:0042325~regulation of phosphorylation | 0.03531378 | 1 | 1.75 |
| GOTERM_BP_FAT | GO:0051346~negative regulation of hydrolase activity | 0.035812274 | 1 | 5.42 |
| GOTERM_BP_FAT | GO:0008299~isoprenoid biosynthetic process | 0.035812274 | 1 | 5.42 |
| GOTERM_BP_FAT | GO:0042364~water-soluble vitamin biosynthetic process | 0.035812274 | 1 | 5.42 |
| INTERPRO | IPR000086:NUDIX hydrolase domain | 0.036132779 | 1 | 5.41 |
| GOTERM_MF_FAT | GO:0051087~chaperone binding | 0.036199277 | 1 | 9.67 |
| SP_PIR_KEYWORDS | ubl conjugation | 0.036522406 | 0.99999981 | 1.52 |
| GOTERM_BP_FAT | GO:0055085~transmembrane transport | 0.036608783 | 1 | 1.55 |
| SMART | SM00186:FBG | 0.036985403 | 0.998950017 | 5.37 |
| GOTERM_BP_FAT | GO:0051347~positive regulation of transferase activity | 0.037665651 | 1 | 2.21 |
| GOTERM_MF_FAT | GO:0005520~insulin-like growth factor binding | 0.038309711 | 1 | 5.28 |
| GOTERM_BP_FAT | GO:0009116~nucleoside metabolic process | 0.038908864 | 1 | 3.19 |
| SP_PIR_KEYWORDS | chromoprotein | 0.040051103 | 0.999999959 | 3.84 |
| GOTERM_BP_FAT | GO:0055074~calcium ion homeostasis | 0.040057597 | 1 | 2.51 |
| GOTERM_BP_FAT | GO:0015918~sterol transport | 0.040190352 | 1 | 5.18 |
| GOTERM_BP_FAT | GO:0030301~cholesterol transport | 0.040190352 | 1 | 5.18 |
| SMART | SM00134:LU | 0.040469705 | 0.999457148 | 5.18 |
| SP_PIR_KEYWORDS | tpr repeat | 0.040698608 | 0.999999969 | 2.18 |
| KEGG_PATHWAY | mmu00650:Butanoate metabolism | 0.04100362 | 0.997690806 | 3.78 |
| KEGG_PATHWAY | mmu00051:Fructose and mannose metabolism | 0.04100362 | 0.997690806 | 3.78 |
| PIR_SUPERFAMILY | PIRSF016521:acyl-CoA thioesterase (hydrolase) | 0.041352159 | 0.999998758 | 8.98 |
| PIR_SUPERFAMILY | PIRSF016521:Acyl-CoA_hydro | 0.041352159 | 0.999998758 | 8.98 |
| GOTERM_BP_FAT | GO:0031589~cell-substrate adhesion | 0.041525382 | 1 | 3.14 |
| INTERPRO | IPR015609:Molecular chaperone, heat shock protein, Hsp40, DnaJ | 0.041679865 | 1 | 3.79 |
| GOTERM_BP_FAT | GO:0051604~protein maturation | 0.042019918 | 1 | 2.48 |
| GOTERM_BP_FAT | GO:0015936~coenzyme A metabolic process | 0.042138239 | 1 | 8.94 |
| PIR_SUPERFAMILY | PIRSF000045:cytochrome P450 CYP2D6 | 0.042691786 | 0.999999208 | 3.74 |
| SP_PIR_KEYWORDS | transit peptide | 0.043204063 | 0.99999999 | 1.55 |
| GOTERM_BP_FAT | GO:0016125~sterol metabolic process | 0.044019562 | 1 | 2.71 |
| GOTERM_BP_FAT | GO:0046128~purine ribonucleoside metabolic process | 0.044825614 | 1 | 4.97 |
| GOTERM_BP_FAT | GO:0001818~negative regulation of cytokine production | 0.044825614 | 1 | 4.97 |
| GOTERM_BP_FAT | GO:0042278~purine nucleoside metabolic process | 0.044825614 | 1 | 4.97 |
| SP_PIR_KEYWORDS | pyridoxal phosphate | 0.04499638 | 0.999999995 | 3.07 |
| GOTERM_BP_FAT | GO:0006869~lipid transport | 0.04646694 | 1 | 2.25 |
| INTERPRO | IPR017891:Insulin-like growth factor binding protein, N-terminal | 0.046669176 | 1 | 8.48 |
| GOTERM_BP_FAT | GO:0051174~regulation of phosphorus metabolic process | 0.046805716 | 1 | 1.68 |
| GOTERM_BP_FAT | GO:0019220~regulation of phosphate metabolic process | 0.046805716 | 1 | 1.68 |
| GOTERM_BP_FAT | GO:0043406~positive regulation of MAP kinase activity | 0.047070525 | 1 | 3.03 |
| BIOCARTA | m_fibrinolysisPathway:Fibrinolysis Pathway | 0.047249604 | 0.979187175 | 8.13 |
| SP_PIR_KEYWORDS | vldl | 0.047717469 | 0.999999999 | 8.38 |
| SMART | SM00271:DnaJ | 0.048389732 | 0.999879895 | 3.61 |
| GOTERM_BP_FAT | GO:0030325~adrenal gland development | 0.050383405 | 1 | 8.13 |
| GOTERM_BP_FAT | GO:0015849~organic acid transport | 0.050486962 | 1 | 2.38 |
| BIOCARTA | m_arenrf2Pathway:Oxidative Stress Induced Gene Expression Via Nrf2 | 0.050780351 | 0.98453544 | 4.57 |
| GOTERM_BP_FAT | GO:0044270~nitrogen compound catabolic process | 0.051077652 | 1 | 3.55 |
| UP_SEQ_FEATURE | domain:Nudix hydrolase | 0.051417892 | 1 | 4.70 |
| UP_SEQ_FEATURE | region of interest:Linker | 0.051417892 | 1 | 4.70 |
| SMART | SM00643:C345C | 0.051623499 | 0.99993536 | 8.05 |
| GOTERM_MF_FAT | GO:0031072~heat shock protein binding | 0.051715481 | 1 | 2.95 |
| SP_PIR_KEYWORDS | hydrolase | 0.051883801 | 1 | 1.25 |
| GOTERM_MF_FAT | GO:0000155~two-component sensor activity | 0.052861829 | 1 | 7.91 |
| GOTERM_MF_FAT | GO:0004673~protein histidine kinase activity | 0.052861829 | 1 | 7.91 |
| GOTERM_MF_FAT | GO:0016289~CoA hydrolase activity | 0.053078298 | 1 | 4.64 |
| GOTERM_BP_FAT | GO:0033554~cellular response to stress | 0.053923476 | 1 | 1.55 |
| GOTERM_BP_FAT | GO:0006633~fatty acid biosynthetic process | 0.054021508 | 1 | 2.58 |
| GOTERM_BP_FAT | GO:0006639~acylglycerol metabolic process | 0.054889318 | 1 | 3.46 |
| GOTERM_MF_FAT | GO:0016836~hydro-lyase activity | 0.055337319 | 1 | 3.45 |
| GOTERM_CC_FAT | GO:0005615~extracellular space | 0.056632345 | 0.999999936 | 1.45 |
| GOTERM_BP_FAT | GO:0045860~positive regulation of protein kinase activity | 0.056654595 | 1 | 2.16 |
| GOTERM_BP_FAT | GO:0043085~positive regulation of catalytic activity | 0.056735505 | 1 | 1.71 |
| BIOCARTA | m_nuclearRsPathway:Nuclear Receptors in Lipid Metabolism and Toxicity | 0.057787985 | 0.991451478 | 3.29 |
| GOTERM_BP_FAT | GO:0051085~chaperone mediated protein folding requiring cofactor | 0.059150476 | 1 | 7.45 |
| GOTERM_BP_FAT | GO:0046470~phosphatidylcholine metabolic process | 0.059150476 | 1 | 7.45 |
| INTERPRO | IPR002181:Fibrinogen, alpha/beta/gamma chain, C-terminal globular | 0.059480738 | 1 | 4.44 |
| GOTERM_CC_FAT | GO:0031225~anchored to membrane | 0.059653705 | 0.999999974 | 1.79 |
| UP_SEQ_FEATURE | domain:UPAR/Ly6 | 0.062745082 | 1 | 4.34 |
| UP_SEQ_FEATURE | domain:MARVEL | 0.062745082 | 1 | 4.34 |
| GOTERM_BP_FAT | GO:0009108~coenzyme biosynthetic process | 0.062765888 | 1 | 2.79 |
| GOTERM_BP_FAT | GO:0001666~response to hypoxia | 0.062765888 | 1 | 2.79 |
| GOTERM_BP_FAT | GO:0006662~glycerol ether metabolic process | 0.062959819 | 1 | 3.31 |
| GOTERM_BP_FAT | GO:0006638~neutral lipid metabolic process | 0.062959819 | 1 | 3.31 |
| INTERPRO | IPR019389:Selenoprotein T | 0.063150382 | 1 | 31.11 |
| INTERPRO | IPR013538:Activator of Hsp90 ATPase homologue 1-like | 0.063150382 | 1 | 31.11 |
| INTERPRO | IPR015310:Activator of Hsp90 ATPase, N-terminal | 0.063150382 | 1 | 31.11 |
| KEGG_PATHWAY | mmu00030:Pentose phosphate pathway | 0.063167349 | 0.999922198 | 4.31 |
| INTERPRO | IPR018253:Heat shock protein DnaJ, conserved site | 0.063329084 | 1 | 3.31 |
| INTERPRO | IPR002035:von Willebrand factor, type A | 0.063578875 | 1 | 2.47 |
| SP_PIR_KEYWORDS | myristate | 0.064679163 | 1 | 2.26 |
| INTERPRO | IPR008253:Marvel | 0.064822882 | 1 | 4.29 |
| INTERPRO | IPR016054:Ly-6 antigen / uPA receptor -like | 0.064822882 | 1 | 4.29 |
| UP_SEQ_FEATURE | site:Important for catalytic activity | 0.06526273 | 1 | 7.05 |
| SP_PIR_KEYWORDS | calcium binding | 0.065650517 | 1 | 3.27 |
| SMART | SM00702:P4Hc | 0.065774019 | 0.99999581 | 7.05 |
| GOTERM_BP_FAT | GO:0031669~cellular response to nutrient levels | 0.065843967 | 1 | 4.26 |
| GOTERM_BP_FAT | GO:0010517~regulation of phospholipase activity | 0.065843967 | 1 | 4.26 |
| GOTERM_BP_FAT | GO:0032764~negative regulation of mast cell cytokine production | 0.065851965 | 1 | 29.80 |
| GOTERM_BP_FAT | GO:0015851~nucleobase transport | 0.065851965 | 1 | 29.80 |
| GOTERM_BP_FAT | GO:0007263~nitric oxide mediated signal transduction | 0.065851965 | 1 | 29.80 |
| GOTERM_BP_FAT | GO:0030497~fatty acid elongation | 0.065851965 | 1 | 29.80 |
| GOTERM_BP_FAT | GO:0060318~definitive erythrocyte differentiation | 0.065851965 | 1 | 29.80 |
| GOTERM_BP_FAT | GO:0009310~amine catabolic process | 0.066218939 | 1 | 2.75 |
| GOTERM_BP_FAT | GO:0070482~response to oxygen levels | 0.066218939 | 1 | 2.75 |
| GOTERM_CC_FAT | GO:0032994~protein-lipid complex | 0.066728881 | 0.999999997 | 4.23 |
| GOTERM_CC_FAT | GO:0034358~plasma lipoprotein particle | 0.066728881 | 0.999999997 | 4.23 |
| GOTERM_MF_FAT | GO:0030235~nitric-oxide synthase regulator activity | 0.067603559 | 1 | 29.01 |
| GOTERM_MF_FAT | GO:0001671~ATPase activator activity | 0.067603559 | 1 | 29.01 |
| GOTERM_BP_FAT | GO:0019915~lipid storage | 0.068395422 | 1 | 6.88 |
| UP_SEQ_FEATURE | domain:Fibrinogen C-terminal | 0.068804812 | 1 | 4.18 |
| UP_SEQ_FEATURE | site:Lowers pKa of C-terminal Cys of first active site | 0.069531703 | 1 | 28.21 |
| UP_SEQ_FEATURE | site:Lowers pKa of C-terminal Cys of second active site | 0.069531703 | 1 | 28.21 |
| KEGG_PATHWAY | mmu00520:Amino sugar and nucleotide sugar metabolism | 0.069736596 | 0.999971955 | 3.18 |
| GOTERM_BP_FAT | GO:0006820~anion transport | 0.070621971 | 1 | 2.06 |
| GOTERM_BP_FAT | GO:0033674~positive regulation of kinase activity | 0.070621971 | 1 | 2.06 |
| GOTERM_BP_FAT | GO:0042401~biogenic amine biosynthetic process | 0.071689718 | 1 | 4.11 |
| GOTERM_BP_FAT | GO:0019637~organophosphate metabolic process | 0.072035027 | 1 | 1.86 |
| INTERPRO | IPR018933:Netrin module, non-TIMP type | 0.072511485 | 1 | 6.67 |
| INTERPRO | IPR007123:Gelsolin region | 0.072511485 | 1 | 6.67 |
| PIR_SUPERFAMILY | PIRSF500265:vesicular integral-membrane protein VIP36 | 0.072627804 | 1 | 26.94 |
| KEGG_PATHWAY | mmu00140:Steroid hormone biosynthesis | 0.074508477 | 0.999986695 | 3.11 |
| GOTERM_BP_FAT | GO:0009611~response to wounding | 0.076076276 | 1 | 1.55 |
| SP_PIR_KEYWORDS | Secreted | 0.076089377 | 1 | 1.23 |
| GOTERM_BP_FAT | GO:0018904~organic ether metabolic process | 0.076162848 | 1 | 3.10 |
| GOTERM_MF_FAT | GO:0016620~oxidoreductase activity, acting on the aldehyde or oxo group of donors, NAD or NADP as acceptor | 0.076348256 | 1 | 4.00 |
| GOTERM_BP_FAT | GO:0043161~proteasomal ubiquitin-dependent protein catabolic process | 0.07775918 | 1 | 3.97 |
| GOTERM_BP_FAT | GO:0034311~diol metabolic process | 0.07775918 | 1 | 3.97 |
| GOTERM_BP_FAT | GO:0006584~catecholamine metabolic process | 0.07775918 | 1 | 3.97 |
| GOTERM_BP_FAT | GO:0018958~phenol metabolic process | 0.07775918 | 1 | 3.97 |
| GOTERM_BP_FAT | GO:0010498~proteasomal protein catabolic process | 0.07775918 | 1 | 3.97 |
| GOTERM_BP_FAT | GO:0009712~catechol metabolic process | 0.07775918 | 1 | 3.97 |
| GOTERM_BP_FAT | GO:0006458~'de novo' protein folding | 0.078076566 | 1 | 6.39 |
| GOTERM_BP_FAT | GO:0051084~'de novo' posttranslational protein folding | 0.078076566 | 1 | 6.39 |
| SP_PIR_KEYWORDS | serine esterase | 0.078362575 | 1 | 3.97 |
| UP_SEQ_FEATURE | domain:J | 0.078916371 | 1 | 3.07 |
| GOTERM_CC_FAT | GO:0005902~microvillus | 0.0792668 | 1 | 3.94 |
| GOTERM_CC_FAT | GO:0005794~Golgi apparatus | 0.080590404 | 1 | 1.34 |
| GOTERM_BP_FAT | GO:0016485~protein processing | 0.08117851 | 1 | 2.32 |
| GOTERM_MF_FAT | GO:0008514~organic anion transmembrane transporter activity | 0.081774992 | 1 | 6.22 |
| UP_SEQ_FEATURE | lipid moiety-binding region:N-myristoyl glycine | 0.082044485 | 1 | 2.13 |
| GOTERM_MF_FAT | GO:0016706~oxidoreductase activity, acting on paired donors, with incorporation or reduction of molecular oxygen, 2-oxoglutarate as one donor, and incorporation of one atom each of oxygen into both donors | 0.082760391 | 1 | 3.87 |
| SP_PIR_KEYWORDS | Growth factor binding | 0.083697515 | 1 | 6.15 |
| SP_PIR_KEYWORDS | nucleotidyltransferase | 0.084648653 | 1 | 2.56 |
| GOTERM_BP_FAT | GO:0007596~blood coagulation | 0.085032115 | 1 | 2.55 |
| GOTERM_BP_FAT | GO:0050817~coagulation | 0.085032115 | 1 | 2.55 |
| INTERPRO | IPR001623:Heat shock protein DnaJ, N-terminal | 0.085069597 | 1 | 2.99 |
| GOTERM_BP_FAT | GO:0007160~cell-matrix adhesion | 0.085675489 | 1 | 2.98 |
| UP_SEQ_FEATURE | binding site:2-oxoglutarate | 0.085933116 | 1 | 6.04 |
| GOTERM_BP_FAT | GO:0001558~regulation of cell growth | 0.088085246 | 1 | 2.27 |
| GOTERM_BP_FAT | GO:0042402~biogenic amine catabolic process | 0.088154476 | 1 | 5.96 |
| GOTERM_BP_FAT | GO:0055010~ventricular cardiac muscle morphogenesis | 0.088154476 | 1 | 5.96 |
| GOTERM_BP_FAT | GO:0030539~male genitalia development | 0.088154476 | 1 | 5.96 |
| GOTERM_BP_FAT | GO:0019439~aromatic compound catabolic process | 0.088154476 | 1 | 5.96 |
| GOTERM_BP_FAT | GO:0000186~activation of MAPKK activity | 0.088154476 | 1 | 5.96 |
| GOTERM_BP_FAT | GO:0008206~bile acid metabolic process | 0.088154476 | 1 | 5.96 |
| GOTERM_BP_FAT | GO:0007599~hemostasis | 0.089099248 | 1 | 2.52 |
| GOTERM_MF_FAT | GO:0008430~selenium binding | 0.089395278 | 1 | 3.74 |
| KEGG_PATHWAY | mmu00983:Drug metabolism | 0.089781334 | 0.999998808 | 2.92 |
| GOTERM_BP_FAT | GO:0044093~positive regulation of molecular function | 0.090586361 | 1 | 1.56 |
| GOTERM_BP_FAT | GO:0009266~response to temperature stimulus | 0.090638494 | 1 | 2.92 |
| GOTERM_BP_FAT | GO:0031667~response to nutrient levels | 0.091297369 | 1 | 2.07 |
| UP_SEQ_FEATURE | cross-link:Glycyl lysine isopeptide (Lys-Gly) (interchain with G-Cter in SUMO) | 0.091620345 | 1 | 2.24 |
| INTERPRO | IPR006620:Prolyl 4-hydroxylase, alpha subunit | 0.091722921 | 1 | 5.83 |
| GOTERM_MF_FAT | GO:0016290~palmitoyl-CoA hydrolase activity | 0.092277509 | 1 | 5.80 |
| INTERPRO | IPR013547:Prolyl 4-hydroxylase alpha-subunit, N-terminal | 0.093216498 | 1 | 20.74 |
| INTERPRO | IPR012290:Fibrinogen, alpha/beta/gamma chain, coiled coil | 0.093216498 | 1 | 20.74 |
| INTERPRO | IPR004113:FAD-linked oxidase, C-terminal | 0.093216498 | 1 | 20.74 |
| INTERPRO | IPR000952:Uncharacterised protein family UPF0017, hydrolase-like, conserved site | 0.093216498 | 1 | 20.74 |
| INTERPRO | IPR007599:Der1-like | 0.093216498 | 1 | 20.74 |
| INTERPRO | IPR001526:CD59 antigen | 0.094597635 | 1 | 3.66 |
| GOTERM_CC_FAT | GO:0005740~mitochondrial envelope | 0.094689626 | 1 | 1.46 |
| GOTERM_CC_FAT | GO:0031966~mitochondrial membrane | 0.094919839 | 1 | 1.47 |
| GOTERM_CC_FAT | GO:0005743~mitochondrial inner membrane | 0.096296224 | 1 | 1.54 |
| UP_SEQ_FEATURE | metal ion-binding site:Magnesium or manganese | 0.096907234 | 1 | 5.64 |
| UP_SEQ_FEATURE | domain:IQ 3 | 0.096907234 | 1 | 5.64 |
| GOTERM_MF_FAT | GO:0050660~FAD binding | 0.097101195 | 1 | 2.45 |
| GOTERM_BP_FAT | GO:0048294~negative regulation of isotype switching to IgE isotypes | 0.097136917 | 1 | 19.87 |
| GOTERM_BP_FAT | GO:0030970~retrograde protein transport, ER to cytosol | 0.097136917 | 1 | 19.87 |
| GOTERM_BP_FAT | GO:0002719~negative regulation of cytokine production during immune response | 0.097136917 | 1 | 19.87 |
| GOTERM_BP_FAT | GO:0010273~detoxification of copper ion | 0.097136917 | 1 | 19.87 |
| GOTERM_BP_FAT | GO:0010744~positive regulation of foam cell differentiation | 0.097136917 | 1 | 19.87 |
| GOTERM_BP_FAT | GO:0045829~negative regulation of isotype switching | 0.097136917 | 1 | 19.87 |
| GOTERM_BP_FAT | GO:0014909~smooth muscle cell migration | 0.097136917 | 1 | 19.87 |
| GOTERM_BP_FAT | GO:0002701~negative regulation of production of molecular mediator of immune response | 0.097136917 | 1 | 19.87 |
| GOTERM_BP_FAT | GO:0048012~hepatocyte growth factor receptor signaling pathway | 0.097136917 | 1 | 19.87 |
| GOTERM_BP_FAT | GO:0032763~regulation of mast cell cytokine production | 0.097136917 | 1 | 19.87 |
| GOTERM_BP_FAT | GO:0060192~negative regulation of lipase activity | 0.097136917 | 1 | 19.87 |
| SP_PIR_KEYWORDS | Rotamase | 0.09728183 | 1 | 3.62 |
| GOTERM_BP_FAT | GO:0007242~intracellular signaling cascade | 0.098549885 | 1 | 1.27 |
| GOTERM_BP_FAT | GO:0030512~negative regulation of transforming growth factor beta receptor signaling pathway | 0.09859186 | 1 | 5.59 |
| GOTERM_BP_FAT | GO:0030307~positive regulation of cell growth | 0.09859186 | 1 | 5.59 |
| GOTERM_CC_FAT | GO:0044421~extracellular region part | 0.098991354 | 1 | 1.29 |
| BIOCARTA | m_cblPathway:CBL mediated ligand-induced downregulation of EGF receptors | 0.09925795 | 0.999766635 | 5.42 |
| GOTERM_MF_FAT | GO:0030911~TPR domain binding | 0.099675302 | 1 | 19.34 |
| GOTERM_MF_FAT | GO:0004586~ornithine decarboxylase activity | 0.099675302 | 1 | 19.34 |
| GOTERM_CC_FAT | GO:0031228~intrinsic to Golgi membrane | 0.099811426 | 1 | 3.57 |
| GOTERM_CC_FAT | GO:0030173~integral to Golgi membrane | 0.099811426 | 1 | 3.57 |

**Suppl. Table S12** Functional annotation terms associated with genes exhibiting UPregulation in diabetes relative to control (n = 244; ranked by ascending p-value)

| **Category** | **Term** | **PValue** | **Bonferroni** | **Fold Enrichment** |
| --- | --- | --- | --- | --- |
| KEGG_PATHWAY | mmu00982:Drug metabolism | 3.39E-11 | 2.51E-09 | 14.63 |
| INTERPRO | IPR003080:Glutathione S-transferase, alpha class | 1.17E-07 | 4.62E-05 | 87.07 |
| GOTERM_MF_FAT | GO:0004364~glutathione transferase activity | 2.48E-07 | 8.95E-05 | 25.19 |
| SP_PIR_KEYWORDS | oxidoreductase | 4.58E-07 | 1.11E-04 | 3.88 |
| GOTERM_BP_FAT | GO:0006766~vitamin metabolic process | 7.05E-07 | 7.12E-04 | 12.06 |
| KEGG_PATHWAY | mmu00980:Metabolism of xenobiotics by cytochrome P450 | 7.57E-07 | 5.60E-05 | 11.51 |
| INTERPRO | IPR004045:Glutathione S-transferase, N-terminal | 5.01E-06 | 0.001975568 | 23.22 |
| INTERPRO | IPR004046:Glutathione S-transferase, C-terminal | 6.05E-06 | 0.002386112 | 22.39 |
| GOTERM_BP_FAT | GO:0055114~oxidation reduction | 9.95E-06 | 0.010006266 | 3.03 |
| GOTERM_BP_FAT | GO:0009636~response to toxin | 1.29E-05 | 0.012955928 | 19.12 |
| INTERPRO | IPR017933:Glutathione S-transferase/chloride channel, C-terminal | 1.64E-05 | 0.00643798 | 18.44 |
| GOTERM_MF_FAT | GO:0016765~transferase activity, transferring alkyl or aryl (other than methyl) groups | 1.95E-05 | 0.00703128 | 12.36 |
| SP_PIR_KEYWORDS | lyase | 2.02E-05 | 0.004866743 | 7.79 |
| GOTERM_BP_FAT | GO:0006575~cellular amino acid derivative metabolic process | 2.09E-05 | 0.020943531 | 6.56 |
| GOTERM_BP_FAT | GO:0006576~biogenic amine metabolic process | 2.36E-05 | 0.023597623 | 9.24 |
| GOTERM_BP_FAT | GO:0051186~cofactor metabolic process | 2.67E-05 | 0.026594082 | 5.59 |
| GOTERM_BP_FAT | GO:0006775~fat-soluble vitamin metabolic process | 2.89E-05 | 0.028839203 | 16.31 |
| SP_PIR_KEYWORDS | microsome | 4.43E-05 | 0.010653688 | 8.45 |
| PIR_SUPERFAMILY | PIRSF000503:glutathione transferase | 4.99E-05 | 0.005079604 | 23.27 |
| UP_SEQ_FEATURE | domain:GST N-terminal | 6.24E-05 | 0.030721974 | 22.48 |
| GOTERM_CC_FAT | GO:0005792~microsome | 6.67E-05 | 0.009821123 | 5.64 |
| GOTERM_CC_FAT | GO:0042598~vesicular fraction | 8.64E-05 | 0.012700342 | 5.45 |
| KEGG_PATHWAY | mmu00830:Retinol metabolism | 1.25E-04 | 0.009228222 | 8.69 |
| GOTERM_BP_FAT | GO:0006732~coenzyme metabolic process | 1.54E-04 | 0.143925306 | 5.82 |
| INTERPRO | IPR010987:Glutathione S-transferase, C-terminal-like | 1.79E-04 | 0.068135985 | 17.41 |
| SP_PIR_KEYWORDS | endoplasmic reticulum | 2.30E-04 | 0.05406388 | 2.80 |
| GOTERM_BP_FAT | GO:0016054~organic acid catabolic process | 2.32E-04 | 0.209350725 | 7.99 |
| GOTERM_BP_FAT | GO:0046395~carboxylic acid catabolic process | 2.32E-04 | 0.209350725 | 7.99 |
| SP_PIR_KEYWORDS | peroxisome | 2.92E-04 | 0.068342915 | 7.70 |
| GOTERM_MF_FAT | GO:0009055~electron carrier activity | 3.01E-04 | 0.103047868 | 4.63 |
| KEGG_PATHWAY | mmu00480:Glutathione metabolism | 3.17E-04 | 0.023213573 | 9.74 |
| UP_SEQ_FEATURE | domain:GST C-terminal | 3.20E-04 | 0.148014085 | 14.98 |
| INTERPRO | IPR012335:Thioredoxin fold | 5.28E-04 | 0.188299774 | 6.90 |
| INTERPRO | IPR002401:Cytochrome P450, E-class, group I | 5.40E-04 | 0.19207245 | 8.96 |
| SP_PIR_KEYWORDS | Monooxygenase | 5.50E-04 | 0.124726246 | 6.85 |
| GOTERM_CC_FAT | GO:0042579~microbody | 6.04E-04 | 0.085532777 | 6.68 |
| GOTERM_CC_FAT | GO:0005777~peroxisome | 6.04E-04 | 0.085532777 | 6.68 |
| GOTERM_CC_FAT | GO:0005783~endoplasmic reticulum | 6.37E-04 | 0.090011223 | 2.37 |
| GOTERM_CC_FAT | GO:0044421~extracellular region part | 6.67E-04 | 0.094078208 | 2.44 |
| INTERPRO | IPR018170:Aldo/keto reductase, conserved site | 7.33E-04 | 0.251433813 | 22.00 |
| UP_SEQ_FEATURE | short sequence motif:Microbody targeting signal | 9.75E-04 | 0.386087311 | 11.24 |
| INTERPRO | IPR017973:Cytochrome P450, C-terminal region | 0.00105083 | 0.339855242 | 7.74 |
| SP_PIR_KEYWORDS | transferase | 0.00122337 | 0.256388647 | 1.98 |
| INTERPRO | IPR001128:Cytochrome P450 | 0.001447797 | 0.435770513 | 7.21 |
| GOTERM_BP_FAT | GO:0046700~heterocycle catabolic process | 0.001470395 | 0.77410014 | 10.05 |
| INTERPRO | IPR017972:Cytochrome P450, conserved site | 0.001601836 | 0.469127066 | 7.04 |
| KEGG_PATHWAY | mmu04610:Complement and coagulation cascades | 0.001714004 | 0.119218022 | 6.75 |
| GOTERM_MF_FAT | GO:0019842~vitamin binding | 0.001816365 | 0.481234837 | 5.41 |
| SP_PIR_KEYWORDS | lipid metabolism | 0.001822215 | 0.356850625 | 5.44 |
| SP_PIR_KEYWORDS | glycoprotein | 0.001899006 | 0.368714074 | 1.50 |
| GOTERM_MF_FAT | GO:0016289~CoA hydrolase activity | 0.002267001 | 0.559267102 | 14.97 |
| SP_PIR_KEYWORDS | nadp | 0.002276314 | 0.423912629 | 5.21 |
| SP_PIR_KEYWORDS | heme | 0.002276314 | 0.423912629 | 5.21 |
| GOTERM_BP_FAT | GO:0046486~glycerolipid metabolic process | 0.00267523 | 0.933348803 | 5.02 |
| SP_PIR_KEYWORDS | Flavoprotein | 0.00289747 | 0.504508785 | 6.15 |
| KEGG_PATHWAY | mmu00340:Histidine metabolism | 0.002912512 | 0.194136064 | 13.50 |
| INTERPRO | IPR016662:Acyl-CoA thioesterase, long chain | 0.003100395 | 0.706699724 | 34.83 |
| INTERPRO | IPR014940:BAAT/Acyl-CoA thioester hydrolase C-terminal | 0.003100395 | 0.706699724 | 34.83 |
| INTERPRO | IPR006862:Acyl-CoA thioester hydrolase/bile acid-CoA amino acid N-acetyltransferase | 0.003100395 | 0.706699724 | 34.83 |
| SP_PIR_KEYWORDS | iron | 0.00342455 | 0.564021973 | 3.29 |
| GOTERM_MF_FAT | GO:0005506~iron ion binding | 0.00358564 | 0.726578942 | 3.00 |
| SP_PIR_KEYWORDS | Secreted | 0.003634504 | 0.585694766 | 1.86 |
| GOTERM_BP_FAT | GO:0006631~fatty acid metabolic process | 0.0037244 | 0.977003136 | 4.02 |
| GOTERM_CC_FAT | GO:0005576~extracellular region | 0.00400007 | 0.447442412 | 1.71 |
| PIR_SUPERFAMILY | PIRSF016521:acyl-CoA thioesterase (hydrolase) | 0.004232691 | 0.351212954 | 29.48 |
| PIR_SUPERFAMILY | PIRSF016521:Acyl-CoA_hydro | 0.004232691 | 0.351212954 | 29.48 |
| GOTERM_BP_FAT | GO:0046942~carboxylic acid transport | 0.004259603 | 0.98664222 | 5.60 |
| GOTERM_MF_FAT | GO:0020037~heme binding | 0.004311927 | 0.789857873 | 4.55 |
| GOTERM_BP_FAT | GO:0015849~organic acid transport | 0.004445775 | 0.988943092 | 5.55 |
| GOTERM_CC_FAT | GO:0005624~membrane fraction | 0.005003024 | 0.523985041 | 2.53 |
| COG_ONTOLOGY | Secondary metabolites biosynthesis, transport, and catabolism | 0.005038869 | 0.049261353 | 4.88 |
| GOTERM_CC_FAT | GO:0005615~extracellular space | 0.005080492 | 0.529438845 | 2.52 |
| GOTERM_BP_FAT | GO:0009310~amine catabolic process | 0.005213977 | 0.994933581 | 7.11 |
| GOTERM_MF_FAT | GO:0046906~tetrapyrrole binding | 0.005438078 | 0.860334267 | 4.34 |
| GOTERM_CC_FAT | GO:0005739~mitochondrion | 0.005671157 | 0.569031496 | 1.80 |
| GOTERM_CC_FAT | GO:0005626~insoluble fraction | 0.006551003 | 0.621953513 | 2.44 |
| GOTERM_CC_FAT | GO:0031090~organelle membrane | 0.006692111 | 0.629818306 | 2.09 |
| GOTERM_BP_FAT | GO:0006641~triglyceride metabolic process | 0.00669419 | 0.998875684 | 10.27 |
| GOTERM_BP_FAT | GO:0046470~phosphatidylcholine metabolic process | 0.007051893 | 0.999218832 | 23.11 |
| UP_SEQ_FEATURE | glycosylation site:N-linked (GlcNAc...) | 0.008025606 | 0.98220775 | 1.41 |
| GOTERM_BP_FAT | GO:0042592~homeostatic process | 0.010208455 | 0.999968759 | 2.22 |
| GOTERM_MF_FAT | GO:0048037~cofactor binding | 0.010381975 | 0.976889866 | 3.31 |
| GOTERM_MF_FAT | GO:0016290~palmitoyl-CoA hydrolase activity | 0.010723348 | 0.979596195 | 18.72 |
| GOTERM_BP_FAT | GO:0006639~acylglycerol metabolic process | 0.010956754 | 0.999985457 | 8.60 |
| GOTERM_MF_FAT | GO:0016712~oxidoreductase activity, acting on paired donors, with incorporation or reduction of molecular oxygen, reduced flavin or flavoprotein as one donor, and incorporation of one atom of oxygen | 0.011276362 | 0.983325796 | 8.51 |
| GOTERM_CC_FAT | GO:0031966~mitochondrial membrane | 0.011510776 | 0.819761742 | 2.70 |
| GOTERM_CC_FAT | GO:0045177~apical part of cell | 0.011725475 | 0.825464062 | 4.38 |
| GOTERM_BP_FAT | GO:0006662~glycerol ether metabolic process | 0.012404166 | 0.999996692 | 8.22 |
| GOTERM_BP_FAT | GO:0006638~neutral lipid metabolic process | 0.012404166 | 0.999996692 | 8.22 |
| GOTERM_MF_FAT | GO:0015293~symporter activity | 0.013239154 | 0.99186235 | 4.25 |
| GOTERM_CC_FAT | GO:0016323~basolateral plasma membrane | 0.013542432 | 0.867075649 | 4.22 |
| GOTERM_BP_FAT | GO:0018904~organic ether metabolic process | 0.0147715 | 0.999999708 | 7.70 |
| GOTERM_BP_FAT | GO:0016042~lipid catabolic process | 0.014777788 | 0.99999971 | 4.14 |
| SMART | SM00186:FBG | 0.014842628 | 0.615975311 | 15.78 |
| GOTERM_MF_FAT | GO:0004857~enzyme inhibitor activity | 0.01498954 | 0.995713092 | 3.08 |
| SP_PIR_KEYWORDS | extracellular matrix | 0.015601613 | 0.97774866 | 3.47 |
| GOTERM_BP_FAT | GO:0042439~ethanolamine and derivative metabolic process | 0.015673668 | 0.999999884 | 15.41 |
| GOTERM_CC_FAT | GO:0044429~mitochondrial part | 0.015810025 | 0.905445402 | 2.27 |
| GOTERM_CC_FAT | GO:0000267~cell fraction | 0.016080854 | 0.909219433 | 2.16 |
| GOTERM_CC_FAT | GO:0005740~mitochondrial envelope | 0.016524358 | 0.91507921 | 2.54 |
| INTERPRO | IPR014716:Fibrinogen, alpha/beta/gamma chain, C-terminal globular, subdomain 1 | 0.016783113 | 0.998751223 | 14.93 |
| GOTERM_BP_FAT | GO:0048878~chemical homeostasis | 0.017057376 | 0.999999972 | 2.53 |
| SP_PIR_KEYWORDS | mitochondrion | 0.017263953 | 0.985218227 | 2.01 |
| GOTERM_CC_FAT | GO:0016324~apical plasma membrane | 0.017289516 | 0.924318932 | 5.01 |
| SP_PIR_KEYWORDS | acetylated amino end | 0.017970565 | 0.987579753 | 14.41 |
| INTERPRO | IPR001395:Aldo/keto reductase | 0.018347364 | 0.999334197 | 14.25 |
| GOTERM_MF_FAT | GO:0016291~acyl-CoA thioesterase activity | 0.018745043 | 0.998920425 | 14.04 |
| SP_PIR_KEYWORDS | pyridoxal phosphate | 0.01893134 | 0.990199338 | 7.04 |
| GOTERM_BP_FAT | GO:0006637~acyl-CoA metabolic process | 0.019193747 | 0.999999997 | 13.87 |
| GOTERM_MF_FAT | GO:0070279~vitamin B6 binding | 0.019564116 | 0.999201398 | 6.93 |
| GOTERM_MF_FAT | GO:0030170~pyridoxal phosphate binding | 0.019564116 | 0.999201398 | 6.93 |
| SP_PIR_KEYWORDS | FAD | 0.019619331 | 0.991729592 | 4.85 |
| SP_PIR_KEYWORDS | fatty acid metabolism | 0.019776011 | 0.992043371 | 6.93 |
| GOTERM_BP_FAT | GO:0009063~cellular amino acid catabolic process | 0.020222592 | 0.999999999 | 6.85 |
| UP_SEQ_FEATURE | binding site:Substrate | 0.021258579 | 0.999978423 | 2.88 |
| GOTERM_BP_FAT | GO:0007263~nitric oxide mediated signal transduction | 0.021374882 | 1 | 92.44 |
| GOTERM_CC_FAT | GO:0031226~intrinsic to plasma membrane | 0.022313892 | 0.964558039 | 2.16 |
| UP_SEQ_FEATURE | nucleotide phosphate-binding region:NADP | 0.023441405 | 0.999992934 | 6.48 |
| GOTERM_BP_FAT | GO:0030003~cellular cation homeostasis | 0.024065063 | 1 | 3.65 |
| KEGG_PATHWAY | mmu00360:Phenylalanine metabolism | 0.026695672 | 0.864979275 | 11.51 |
| INTERPRO | IPR012290:Fibrinogen, alpha/beta/gamma chain, coiled coil | 0.028273356 | 0.999987979 | 69.66 |
| INTERPRO | IPR002181:Fibrinogen, alpha/beta/gamma chain, C-terminal globular | 0.028930012 | 0.999990796 | 11.20 |
| GOTERM_CC_FAT | GO:0005578~proteinaceous extracellular matrix | 0.029488168 | 0.988084192 | 2.67 |
| GOTERM_CC_FAT | GO:0005577~fibrinogen complex | 0.029693945 | 0.988452347 | 66.16 |
| UP_SEQ_FEATURE | region of interest:Beta | 0.029847824 | 0.999999737 | 65.93 |
| UP_SEQ_FEATURE | region of interest:Alpha | 0.029847824 | 0.999999737 | 65.93 |
| UP_SEQ_FEATURE | domain:Fibrinogen C-terminal | 0.029888762 | 0.999999743 | 10.99 |
| GOTERM_BP_FAT | GO:0006776~vitamin A metabolic process | 0.031487216 | 1 | 10.67 |
| SP_PIR_KEYWORDS | carbohydrate metabolism | 0.031513497 | 0.999568834 | 5.79 |
| GOTERM_BP_FAT | GO:0060192~negative regulation of lipase activity | 0.031891536 | 1 | 61.62 |
| GOTERM_BP_FAT | GO:0010273~detoxification of copper ion | 0.031891536 | 1 | 61.62 |
| GOTERM_CC_FAT | GO:0031012~extracellular matrix | 0.035427563 | 0.995196504 | 2.57 |
| GOTERM_BP_FAT | GO:0016101~diterpenoid metabolic process | 0.036117057 | 1 | 9.90 |
| GOTERM_BP_FAT | GO:0001523~retinoid metabolic process | 0.036117057 | 1 | 9.90 |
| SP_PIR_KEYWORDS | carboxypeptidase | 0.036301421 | 0.99987005 | 9.90 |
| KEGG_PATHWAY | mmu00030:Pentose phosphate pathway | 0.036456749 | 0.935957272 | 9.74 |
| SP_PIR_KEYWORDS | histidine metabolism | 0.037113774 | 0.99989404 | 52.82 |
| SP_PIR_KEYWORDS | metal-thiolate cluster | 0.037113774 | 0.99989404 | 52.82 |
| INTERPRO | IPR003019:Metallothionein superfamily, eukaryotic | 0.037520079 | 0.999999725 | 52.24 |
| INTERPRO | IPR018064:Metallothionein, vertebrate, metal binding site | 0.037520079 | 0.999999725 | 52.24 |
| INTERPRO | IPR000006:Metallothionein, vertebrate | 0.037520079 | 0.999999725 | 52.24 |
| GOTERM_BP_FAT | GO:0019748~secondary metabolic process | 0.038060573 | 1 | 5.36 |
| SP_PIR_KEYWORDS | membrane | 0.038158896 | 0.999918528 | 1.23 |
| GOTERM_MF_FAT | GO:0008238~exopeptidase activity | 0.038236073 | 0.999999228 | 5.35 |
| GOTERM_BP_FAT | GO:0009110~vitamin biosynthetic process | 0.038524362 | 1 | 9.56 |
| GOTERM_BP_FAT | GO:0006721~terpenoid metabolic process | 0.038524362 | 1 | 9.56 |
| KEGG_PATHWAY | mmu01040:Biosynthesis of unsaturated fatty acids | 0.039081863 | 0.947664919 | 9.38 |
| GOTERM_BP_FAT | GO:0007596~blood coagulation | 0.039461356 | 1 | 5.28 |
| GOTERM_BP_FAT | GO:0050817~coagulation | 0.039461356 | 1 | 5.28 |
| UP_SEQ_FEATURE | metal ion-binding site:Divalent metal cation; cluster B | 0.03959901 | 0.999999998 | 49.45 |
| UP_SEQ_FEATURE | metal ion-binding site:Divalent metal cation; cluster A | 0.03959901 | 0.999999998 | 49.45 |
| GOTERM_BP_FAT | GO:0007599~hemostasis | 0.040888085 | 1 | 5.21 |
| GOTERM_BP_FAT | GO:0006865~amino acid transport | 0.040888085 | 1 | 5.21 |
| GOTERM_BP_FAT | GO:0006584~catecholamine metabolic process | 0.040991162 | 1 | 9.24 |
| GOTERM_BP_FAT | GO:0009712~catechol metabolic process | 0.040991162 | 1 | 9.24 |
| GOTERM_BP_FAT | GO:0018958~phenol metabolic process | 0.040991162 | 1 | 9.24 |
| GOTERM_BP_FAT | GO:0034311~diol metabolic process | 0.040991162 | 1 | 9.24 |
| GOTERM_MF_FAT | GO:0004740~pyruvate dehydrogenase (acetyl-transferring) kinase activity | 0.041778148 | 0.999999796 | 46.79 |
| GOTERM_BP_FAT | GO:0042359~vitamin D metabolic process | 0.042295941 | 1 | 46.22 |
| GOTERM_MF_FAT | GO:0015294~solute:cation symporter activity | 0.042470563 | 0.999999843 | 5.13 |
| GOTERM_BP_FAT | GO:0009309~amine biosynthetic process | 0.043818882 | 1 | 5.06 |
| PIR_SUPERFAMILY | PIRSF002564:metallothionein | 0.044002382 | 0.989846994 | 44.22 |
| GOTERM_CC_FAT | GO:0031967~organelle envelope | 0.044006376 | 0.998719618 | 2.02 |
| GOTERM_CC_FAT | GO:0031975~envelope | 0.044930487 | 0.998890372 | 2.01 |
| GOTERM_BP_FAT | GO:0060191~regulation of lipase activity | 0.046097127 | 1 | 8.67 |
| SP_PIR_KEYWORDS | metal binding | 0.046176232 | 0.999989253 | 42.26 |
| INTERPRO | IPR005467:Signal transduction histidine kinase, core | 0.046679328 | 0.999999994 | 41.80 |
| INTERPRO | IPR014715:Fibrinogen, alpha/beta/gamma chain, C-terminal globular, subdomain 2 | 0.046679328 | 0.999999994 | 41.80 |
| INTERPRO | IPR018955:Branched-chain alpha-ketoacid dehydrogenase kinase/Pyruvate dehydrogenase kinase, mitochondrial | 0.046679328 | 0.999999994 | 41.80 |
| GOTERM_BP_FAT | GO:0032774~RNA biosynthetic process | 0.046892211 | 1 | 3.67 |
| SP_PIR_KEYWORDS | blood coagulation | 0.047286834 | 0.999991893 | 8.57 |
| KEGG_PATHWAY | mmu00860:Porphyrin and chlorophyll metabolism | 0.047369123 | 0.972430787 | 8.44 |
| GOTERM_MF_FAT | GO:0008235~metalloexopeptidase activity | 0.047648423 | 0.999999978 | 8.51 |
| GOTERM_BP_FAT | GO:0055080~cation homeostasis | 0.048434555 | 1 | 3.01 |
| UP_SEQ_FEATURE | domain:Histidine kinase | 0.049252788 | 1 | 39.56 |
| UP_SEQ_FEATURE | active site:Charge relay system | 0.049747976 | 1 | 3.00 |
| GOTERM_BP_FAT | GO:0006767~water-soluble vitamin metabolic process | 0.051422961 | 1 | 8.16 |
| GOTERM_BP_FAT | GO:0006261~DNA-dependent DNA replication | 0.051422961 | 1 | 8.16 |
| SMART | SM00099:btg1 | 0.052230797 | 0.967717591 | 36.82 |
| GOTERM_BP_FAT | GO:0018106~peptidyl-histidine phosphorylation | 0.052589285 | 1 | 36.97 |
| UP_SEQ_FEATURE | metal ion-binding site:Zinc | 0.052805938 | 1 | 3.53 |
| GOTERM_MF_FAT | GO:0008374~O-acyltransferase activity | 0.052968078 | 0.999999997 | 8.02 |
| GOTERM_MF_FAT | GO:0004180~carboxypeptidase activity | 0.052968078 | 0.999999997 | 8.02 |
| GOTERM_CC_FAT | GO:0044459~plasma membrane part | 0.054567443 | 0.999752644 | 1.46 |
| PIR_SUPERFAMILY | PIRSF000718:branched-chain alpha-keto acid dehydrogenase kinase | 0.054700338 | 0.996778269 | 35.37 |
| INTERPRO | IPR008067:Cytochrome P450, E-class, group I, CYP2A-like | 0.055751926 | 1 | 34.83 |
| GOTERM_BP_FAT | GO:0030005~cellular di-, tri-valent inorganic cation homeostasis | 0.056410781 | 1 | 3.45 |
| INTERPRO | IPR015421:Pyridoxal phosphate-dependent transferase, major region, subdomain 1 | 0.057948138 | 1 | 7.65 |
| SMART | SM00209:TSP1 | 0.058920351 | 0.979484201 | 7.49 |
| SP_PIR_KEYWORDS | Acyltransferase | 0.05926643 | 0.999999621 | 3.41 |
| GOTERM_BP_FAT | GO:0055085~transmembrane transport | 0.060079618 | 1 | 2.01 |
| PIR_SUPERFAMILY | PIRSF000045:cytochrome P450 CYP2D6 | 0.060990621 | 0.998369455 | 7.37 |
| KEGG_PATHWAY | mmu03320:PPAR signaling pathway | 0.064256432 | 0.992661506 | 4.27 |
| INTERPRO | IPR008069:Cytochrome P450, E-class, group I, CYP2D-like | 0.064738688 | 1 | 29.85 |
| GOTERM_BP_FAT | GO:0016053~organic acid biosynthetic process | 0.065513405 | 1 | 3.28 |
| GOTERM_BP_FAT | GO:0046394~carboxylic acid biosynthetic process | 0.065513405 | 1 | 3.28 |
| GOTERM_CC_FAT | GO:0005789~endoplasmic reticulum membrane | 0.066001748 | 0.999959148 | 3.26 |
| SP_PIR_KEYWORDS | acetylation | 0.067555417 | 0.999999955 | 1.36 |
| KEGG_PATHWAY | mmu00051:Fructose and mannose metabolism | 0.068871087 | 0.994909903 | 6.84 |
| GOTERM_BP_FAT | GO:0050878~regulation of body fluid levels | 0.070837755 | 1 | 4.15 |
| GOTERM_BP_FAT | GO:0055066~di-, tri-valent inorganic cation homeostasis | 0.072186581 | 1 | 3.17 |
| SP_PIR_KEYWORDS | calcium binding | 0.072259928 | 0.999999987 | 6.74 |
| GOTERM_BP_FAT | GO:0042445~hormone metabolic process | 0.072727691 | 1 | 4.11 |
| GOTERM_BP_FAT | GO:0018202~peptidyl-histidine modification | 0.072847492 | 1 | 26.41 |
| GOTERM_BP_FAT | GO:0006882~cellular zinc ion homeostasis | 0.072847492 | 1 | 26.41 |
| GOTERM_BP_FAT | GO:0046688~response to copper ion | 0.072847492 | 1 | 26.41 |
| SP_PIR_KEYWORDS | dimer | 0.07285801 | 0.999999989 | 26.41 |
| SP_PIR_KEYWORDS | lipid binding | 0.07285801 | 0.999999989 | 26.41 |
| SP_PIR_KEYWORDS | Sodium | 0.073317329 | 0.99999999 | 4.10 |
| INTERPRO | IPR002087:Anti-proliferative protein | 0.07364042 | 1 | 26.12 |
| KEGG_PATHWAY | mmu00350:Tyrosine metabolism | 0.075508323 | 0.997002064 | 6.49 |
| SP_PIR_KEYWORDS | nad | 0.075812994 | 0.999999995 | 3.13 |
| GOTERM_CC_FAT | GO:0005743~mitochondrial inner membrane | 0.076439891 | 0.999992258 | 2.35 |
| GOTERM_CC_FAT | GO:0042175~nuclear envelope-endoplasmic reticulum network | 0.076523582 | 0.999992361 | 3.10 |
| GOTERM_BP_FAT | GO:0015837~amine transport | 0.076574774 | 1 | 4.02 |
| GOTERM_BP_FAT | GO:0019725~cellular homeostasis | 0.076859214 | 1 | 2.16 |
| UP_SEQ_FEATURE | metal ion-binding site:Iron (heme axial ligand) | 0.077855498 | 1 | 4.00 |
| KEGG_PATHWAY | mmu00380:Tryptophan metabolism | 0.078901015 | 0.997716156 | 6.33 |
| BIOCARTA | m_fibrinolysisPathway:Fibrinolysis Pathway | 0.079329252 | 0.892646227 | 22.52 |
| GOTERM_MF_FAT | GO:0008172~S-methyltransferase activity | 0.081822747 | 1 | 23.39 |
| GOTERM_MF_FAT | GO:0016840~carbon-nitrogen lyase activity | 0.081822747 | 1 | 23.39 |
| INTERPRO | IPR002355:Multicopper oxidase, copper-binding site | 0.082457923 | 1 | 23.22 |
| INTERPRO | IPR012269:Aquaporin | 0.082457923 | 1 | 23.22 |
| GOTERM_BP_FAT | GO:0019432~triglyceride biosynthetic process | 0.082814671 | 1 | 23.11 |
| GOTERM_BP_FAT | GO:0055069~zinc ion homeostasis | 0.082814671 | 1 | 23.11 |
| GOTERM_BP_FAT | GO:0017144~drug metabolic process | 0.082814671 | 1 | 23.11 |
| SP_PIR_KEYWORDS | Symport | 0.083640613 | 0.999999999 | 3.88 |
| GOTERM_CC_FAT | GO:0005887~integral to plasma membrane | 0.084517569 | 0.99999789 | 1.87 |
| GOTERM_MF_FAT | GO:0015370~solute:sodium symporter activity | 0.085522816 | 1 | 6.10 |
| UP_SEQ_FEATURE | compositionally biased region:Ala/Pro-rich | 0.086913014 | 1 | 21.98 |
| GOTERM_BP_FAT | GO:0008610~lipid biosynthetic process | 0.087061088 | 1 | 2.27 |
| SP_PIR_KEYWORDS | Sodium transport | 0.0872133 | 1 | 3.81 |
| SP_PIR_KEYWORDS | signal | 0.088921658 | 1 | 1.28 |
| GOTERM_BP_FAT | GO:0006974~response to DNA damage stimulus | 0.089308164 | 1 | 2.25 |
| SP_PIR_KEYWORDS | Schiff base | 0.090232327 | 1 | 21.13 |
| GOTERM_BP_FAT | GO:0006706~steroid catabolic process | 0.092675427 | 1 | 20.54 |
| GOTERM_BP_FAT | GO:0000038~very-long-chain fatty acid metabolic process | 0.092675427 | 1 | 20.54 |
| GOTERM_CC_FAT | GO:0019866~organelle inner membrane | 0.092761705 | 0.999999447 | 2.23 |
| GOTERM_BP_FAT | GO:0006875~cellular metal ion homeostasis | 0.092826417 | 1 | 3.70 |
| GOTERM_BP_FAT | GO:0043086~negative regulation of catalytic activity | 0.094951177 | 1 | 3.66 |
| GOTERM_MF_FAT | GO:0031402~sodium ion binding | 0.096477669 | 1 | 3.63 |
| GOTERM_BP_FAT | GO:0006720~isoprenoid metabolic process | 0.097266988 | 1 | 5.66 |
| GOTERM_MF_FAT | GO:0015171~amino acid transmembrane transporter activity | 0.098535426 | 1 | 5.61 |
| INTERPRO | IPR017891:Insulin-like growth factor binding protein, N-terminal | 0.099843404 | 1 | 19.00 |

**Suppl. Table S13** Functional annotation terms associated with genes exhibiting DOWNregulation in diabetes relative to control (n = 338; ranked by ascending p-value)

| **Category** | **Term** | **PValue** | **Bonferroni** | **Fold Enrichment** |
| --- | --- | --- | --- | --- |
| SP_PIR_KEYWORDS | endoplasmic reticulum | 3.51E-16 | 1.18E-13 | 3.58 |
| GOTERM_CC_FAT | GO:0005783~endoplasmic reticulum | 1.49E-14 | 3.81E-12 | 2.97 |
| SP_PIR_KEYWORDS | stress response | 3.85E-14 | 1.37E-11 | 15.41 |
| GOTERM_CC_FAT | GO:0044432~endoplasmic reticulum part | 9.01E-14 | 2.31E-11 | 5.38 |
| SP_PIR_KEYWORDS | Chaperone | 3.57E-11 | 1.27E-08 | 6.36 |
| GOTERM_CC_FAT | GO:0005788~endoplasmic reticulum lumen | 1.98E-10 | 5.07E-08 | 8.91 |
| GOTERM_BP_FAT | GO:0006457~protein folding | 3.98E-09 | 6.51E-06 | 6.23 |
| GOTERM_CC_FAT | GO:0042470~melanosome | 2.01E-08 | 5.14E-06 | 7.07 |
| GOTERM_CC_FAT | GO:0048770~pigment granule | 2.01E-08 | 5.14E-06 | 7.07 |
| UP_SEQ_FEATURE | short sequence motif:Prevents secretion from ER | 2.22E-08 | 2.45E-05 | 8.69 |
| SP_PIR_KEYWORDS | oxidoreductase | 1.56E-07 | 5.55E-05 | 2.73 |
| GOTERM_BP_FAT | GO:0055114~oxidation reduction | 1.79E-07 | 2.93E-04 | 2.55 |
| INTERPRO | IPR000886:Endoplasmic reticulum, targeting sequence | 3.82E-07 | 2.99E-04 | 10.33 |
| INTERPRO | IPR013126:Heat shock protein 70 | 2.31E-06 | 0.001805504 | 24.22 |
| INTERPRO | IPR001023:Heat shock protein Hsp70 | 2.31E-06 | 0.001805504 | 24.22 |
| GOTERM_CC_FAT | GO:0042175~nuclear envelope-endoplasmic reticulum network | 2.34E-06 | 5.99E-04 | 4.26 |
| GOTERM_CC_FAT | GO:0042598~vesicular fraction | 2.91E-06 | 7.43E-04 | 3.96 |
| SP_PIR_KEYWORDS | nadp | 5.20E-06 | 0.001843366 | 4.58 |
| GOTERM_CC_FAT | GO:0005789~endoplasmic reticulum membrane | 5.71E-06 | 0.001461693 | 4.22 |
| INTERPRO | IPR018181:Heat shock protein 70, conserved site | 6.19E-06 | 0.004838585 | 20.50 |
| SP_PIR_KEYWORDS | molecular chaperone | 1.06E-05 | 0.003770482 | 18.57 |
| GOTERM_CC_FAT | GO:0000267~cell fraction | 1.37E-05 | 0.003495398 | 2.29 |
| SP_PIR_KEYWORDS | acetylation | 1.41E-05 | 0.004983101 | 1.58 |
| SP_PIR_KEYWORDS | lipid synthesis | 1.72E-05 | 0.006103509 | 5.25 |
| GOTERM_MF_FAT | GO:0003756~protein disulfide isomerase activity | 1.99E-05 | 0.010042022 | 26.37 |
| GOTERM_MF_FAT | GO:0016864~intramolecular oxidoreductase activity, transposing S-S bonds | 1.99E-05 | 0.010042022 | 26.37 |
| GOTERM_BP_FAT | GO:0008610~lipid biosynthetic process | 2.74E-05 | 0.043778374 | 3.09 |
| GOTERM_BP_FAT | GO:0006694~steroid biosynthetic process | 3.05E-05 | 0.048586788 | 6.19 |
| GOTERM_CC_FAT | GO:0005792~microsome | 3.33E-05 | 0.008489697 | 3.64 |
| GOTERM_MF_FAT | GO:0016862~intramolecular oxidoreductase activity, interconverting keto- and enol-groups | 3.51E-05 | 0.017670985 | 23.44 |
| GOTERM_BP_FAT | GO:0019725~cellular homeostasis | 3.68E-05 | 0.058407734 | 2.82 |
| SP_PIR_KEYWORDS | heat shock | 4.76E-05 | 0.016762518 | 43.33 |
| SP_PIR_KEYWORDS | stress-induced protein | 4.76E-05 | 0.016762518 | 43.33 |
| SP_PIR_KEYWORDS | membrane | 5.06E-05 | 0.017816015 | 1.30 |
| GOTERM_CC_FAT | GO:0005626~insoluble fraction | 5.43E-05 | 0.013815876 | 2.28 |
| UP_SEQ_FEATURE | topological domain:Lumenal | 5.53E-05 | 0.059377839 | 2.48 |
| SMART | SM00028:TPR | 6.56E-05 | 0.009788648 | 4.98 |
| GOTERM_CC_FAT | GO:0012505~endomembrane system | 6.86E-05 | 0.017406624 | 2.25 |
| GOTERM_CC_FAT | GO:0005624~membrane fraction | 7.34E-05 | 0.018619219 | 2.28 |
| GOTERM_BP_FAT | GO:0045454~cell redox homeostasis | 7.43E-05 | 0.114258675 | 6.38 |
| SP_PIR_KEYWORDS | Isomerase | 8.86E-05 | 0.030978376 | 4.41 |
| GOTERM_BP_FAT | GO:0006695~cholesterol biosynthetic process | 1.39E-04 | 0.203615014 | 11.47 |
| SP_PIR_KEYWORDS | signal | 1.54E-04 | 0.053253125 | 1.43 |
| GOTERM_CC_FAT | GO:0031410~cytoplasmic vesicle | 1.69E-04 | 0.042375831 | 2.21 |
| GOTERM_BP_FAT | GO:0006986~response to unfolded protein | 2.04E-04 | 0.283467356 | 6.51 |
| INTERPRO | IPR005788:Disulphide isomerase | 2.14E-04 | 0.154419355 | 29.61 |
| INTERPRO | IPR019734:Tetratricopeptide repeat | 2.16E-04 | 0.155636555 | 4.36 |
| GOTERM_MF_FAT | GO:0051082~unfolded protein binding | 2.35E-04 | 0.112543065 | 5.42 |
| GOTERM_CC_FAT | GO:0031982~vesicle | 2.38E-04 | 0.059055392 | 2.16 |
| SP_PIR_KEYWORDS | Redox-active center | 2.46E-04 | 0.083626099 | 7.78 |
| INTERPRO | IPR013766:Thioredoxin domain | 2.49E-04 | 0.177051713 | 10.25 |
| SP_PIR_KEYWORDS | microsome | 4.99E-04 | 0.162321698 | 4.33 |
| GOTERM_BP_FAT | GO:0016126~sterol biosynthetic process | 5.18E-04 | 0.57124377 | 8.79 |
| INTERPRO | IPR011990:Tetratricopeptide-like helical | 5.86E-04 | 0.368209514 | 3.55 |
| INTERPRO | IPR016040:NAD(P)-binding domain | 6.19E-04 | 0.38463188 | 3.53 |
| SP_PIR_KEYWORDS | Cholesterol biosynthesis | 6.55E-04 | 0.207660449 | 12.04 |
| GOTERM_BP_FAT | GO:0010817~regulation of hormone levels | 6.63E-04 | 0.661928505 | 3.78 |
| GOTERM_CC_FAT | GO:0016023~cytoplasmic membrane-bounded vesicle | 6.76E-04 | 0.159043482 | 2.23 |
| GOTERM_BP_FAT | GO:0006720~isoprenoid metabolic process | 7.95E-04 | 0.727543962 | 6.28 |
| GOTERM_BP_FAT | GO:0042592~homeostatic process | 8.04E-04 | 0.731243003 | 2.03 |
| UP_SEQ_FEATURE | domain:Thioredoxin 2 | 8.17E-04 | 0.595339295 | 19.73 |
| UP_SEQ_FEATURE | domain:Thioredoxin 1 | 8.17E-04 | 0.595339295 | 19.73 |
| GOTERM_CC_FAT | GO:0031988~membrane-bounded vesicle | 8.17E-04 | 0.188814672 | 2.19 |
| PIR_SUPERFAMILY | PIRSF002581:chaperone HSP70 | 8.51E-04 | 0.182650722 | 19.37 |
| INTERPRO | IPR013026:Tetratricopeptide region | 0.00108178 | 0.571975454 | 3.90 |
| GOTERM_BP_FAT | GO:0008202~steroid metabolic process | 0.00110964 | 0.837025092 | 3.28 |
| GOTERM_CC_FAT | GO:0042579~microbody | 0.001125208 | 0.250399874 | 3.85 |
| GOTERM_CC_FAT | GO:0005777~peroxisome | 0.001125208 | 0.250399874 | 3.85 |
| INTERPRO | IPR001440:Tetratricopeptide TPR-1 | 0.001338996 | 0.650228895 | 4.21 |
| SP_PIR_KEYWORDS | unfolded protein response | 0.001456921 | 0.404041442 | 9.85 |
| GOTERM_BP_FAT | GO:0018401~peptidyl-proline hydroxylation to 4-hydroxy-L-proline | 0.001513424 | 0.915820865 | 43.97 |
| GOTERM_BP_FAT | GO:0019471~4-hydroxyproline metabolic process | 0.001513424 | 0.915820865 | 43.97 |
| INTERPRO | IPR006662:Thioredoxin-like subdomain | 0.00162289 | 0.72011576 | 16.15 |
| SP_PIR_KEYWORDS | peroxisome | 0.001672308 | 0.447977374 | 4.06 |
| SP_PIR_KEYWORDS | heme | 0.001678593 | 0.449209756 | 3.36 |
| GOTERM_MF_FAT | GO:0016860~intramolecular oxidoreductase activity | 0.001889118 | 0.617328959 | 6.66 |
| SP_PIR_KEYWORDS | sterol biosynthesis | 0.002040926 | 0.515805724 | 9.03 |
| SP_PIR_KEYWORDS | Steroid biosynthesis | 0.002138713 | 0.532359842 | 6.50 |
| INTERPRO | IPR017937:Thioredoxin, conserved site | 0.002182591 | 0.819679842 | 8.88 |
| KEGG_PATHWAY | mmu03320:PPAR signaling pathway | 0.002520509 | 0.283321004 | 4.24 |
| SP_PIR_KEYWORDS | lipoprotein | 0.002568056 | 0.598614323 | 1.91 |
| INTERPRO | IPR012335:Thioredoxin fold | 0.002686022 | 0.878601215 | 3.77 |
| GOTERM_BP_FAT | GO:0030433~ER-associated protein catabolic process | 0.002786259 | 0.989528538 | 13.53 |
| INTERPRO | IPR001404:Heat shock protein Hsp90 | 0.002930694 | 0.89984511 | 33.31 |
| INTERPRO | IPR005792:Protein disulphide isomerase | 0.002930694 | 0.89984511 | 33.31 |
| INTERPRO | IPR019805:Heat shock protein Hsp90, conserved site | 0.002930694 | 0.89984511 | 33.31 |
| GOTERM_BP_FAT | GO:0018208~peptidyl-proline modification | 0.002981483 | 0.992395537 | 32.98 |
| GOTERM_MF_FAT | GO:0004656~procollagen-proline 4-dioxygenase activity | 0.003236281 | 0.807314638 | 31.64 |
| GOTERM_MF_FAT | GO:0031545~peptidyl-proline 4-dioxygenase activity | 0.003236281 | 0.807314638 | 31.64 |
| UP_SEQ_FEATURE | signal peptide | 0.003240998 | 0.972501143 | 1.31 |
| GOTERM_MF_FAT | GO:0005506~iron ion binding | 0.003328542 | 0.816165549 | 2.21 |
| INTERPRO | IPR001128:Cytochrome P450 | 0.003424708 | 0.9320905 | 4.08 |
| GOTERM_CC_FAT | GO:0031090~organelle membrane | 0.003430827 | 0.585134909 | 1.68 |
| SP_PIR_KEYWORDS | Monooxygenase | 0.003500974 | 0.712066754 | 3.61 |
| GOTERM_BP_FAT | GO:0051789~response to protein stimulus | 0.003568724 | 0.99709617 | 4.04 |
| UP_SEQ_FEATURE | metal ion-binding site:Iron (heme axial ligand) | 0.003597088 | 0.981484655 | 3.59 |
| PIR_SUPERFAMILY | PIRSF002583:Hsp90 | 0.003808422 | 0.595182602 | 29.06 |
| INTERPRO | IPR017972:Cytochrome P450, conserved site | 0.003887626 | 0.952822524 | 3.99 |
| KEGG_PATHWAY | mmu00900:Terpenoid backbone biosynthesis | 0.003914253 | 0.404107631 | 11.97 |
| INTERPRO | IPR004156:Organic anion transporter polypeptide OATP | 0.004186308 | 0.962707177 | 11.84 |
| GOTERM_BP_FAT | GO:0042445~hormone metabolic process | 0.004308862 | 0.999137688 | 3.91 |
| UP_SEQ_FEATURE | nucleotide phosphate-binding region:NADP | 0.004323754 | 0.991743659 | 4.53 |
| GOTERM_BP_FAT | GO:0019748~secondary metabolic process | 0.004677229 | 0.999528938 | 4.46 |
| GOTERM_BP_FAT | GO:0016053~organic acid biosynthetic process | 0.004830354 | 0.999633651 | 3.12 |
| GOTERM_BP_FAT | GO:0046394~carboxylic acid biosynthetic process | 0.004830354 | 0.999633651 | 3.12 |
| GOTERM_BP_FAT | GO:0008203~cholesterol metabolic process | 0.005020636 | 0.999731962 | 4.40 |
| GOTERM_BP_FAT | GO:0048732~gland development | 0.005336076 | 0.999840345 | 2.68 |
| SP_PIR_KEYWORDS | tpr repeat | 0.005398415 | 0.853631639 | 3.07 |
| GOTERM_BP_FAT | GO:0009408~response to heat | 0.005644055 | 0.999903745 | 6.87 |
| INTERPRO | IPR011497:Protease inhibitor, Kazal-type | 0.006131803 | 0.991950398 | 6.73 |
| GOTERM_CC_FAT | GO:0005794~Golgi apparatus | 0.006339351 | 0.803685957 | 1.70 |
| SMART | SM00387:HATPase_c | 0.006606651 | 0.630012369 | 10.15 |
| GOTERM_CC_FAT | GO:0031301~integral to organelle membrane | 0.006654302 | 0.818988439 | 3.60 |
| GOTERM_CC_FAT | GO:0031300~intrinsic to organelle membrane | 0.006803411 | 0.825812822 | 3.22 |
| GOTERM_MF_FAT | GO:0020037~heme binding | 0.007199626 | 0.974539819 | 2.93 |
| GOTERM_BP_FAT | GO:0016125~sterol metabolic process | 0.007963342 | 0.99999788 | 4.00 |
| INTERPRO | IPR002347:Glucose/ribitol dehydrogenase | 0.008332649 | 0.998584351 | 4.76 |
| INTERPRO | IPR017936:Thioredoxin-like | 0.008374261 | 0.998630166 | 6.17 |
| GOTERM_MF_FAT | GO:0000166~nucleotide binding | 0.008622211 | 0.987711304 | 1.33 |
| PIR_SUPERFAMILY | PIRSF001487:protein disulfide-isomerase | 0.009202377 | 0.888201851 | 19.37 |
| PIR_SUPERFAMILY | PIRSF000126:11beta-hydroxysteroid dehydrogenase | 0.009202377 | 0.888201851 | 19.37 |
| INTERPRO | IPR003594:ATP-binding region, ATPase-like | 0.009653446 | 0.999502079 | 8.88 |
| GOTERM_MF_FAT | GO:0046906~tetrapyrrole binding | 0.009691541 | 0.992897547 | 2.79 |
| INTERPRO | IPR017973:Cytochrome P450, C-terminal region | 0.009756731 | 0.999541173 | 3.84 |
| GOTERM_CC_FAT | GO:0005829~cytosol | 0.010279032 | 0.928998192 | 1.75 |
| UP_SEQ_FEATURE | binding site:Substrate | 0.01045787 | 0.999991174 | 2.15 |
| GOTERM_MF_FAT | GO:0031543~peptidyl-proline dioxygenase activity | 0.010806276 | 0.995992007 | 18.08 |
| GOTERM_MF_FAT | GO:0019798~procollagen-proline dioxygenase activity | 0.010806276 | 0.995992007 | 18.08 |
| GOTERM_BP_FAT | GO:0015711~organic anion transport | 0.011409753 | 0.999999993 | 5.64 |
| GOTERM_MF_FAT | GO:0031072~heat shock protein binding | 0.012615141 | 0.998418303 | 4.29 |
| SP_PIR_KEYWORDS | myristate | 0.013034975 | 0.990513068 | 3.18 |
| SP_PIR_KEYWORDS | FAD | 0.013034975 | 0.990513068 | 3.18 |
| INTERPRO | IPR015609:Molecular chaperone, heat shock protein, Hsp40, DnaJ | 0.013191843 | 0.999969907 | 5.42 |
| UP_SEQ_FEATURE | transmembrane region | 0.013300919 | 0.999999635 | 1.20 |
| INTERPRO | IPR002198:Short-chain dehydrogenase/reductase SDR | 0.014390573 | 0.999988396 | 4.16 |
| COG_ONTOLOGY | Posttranslational modification, protein turnover, chaperones | 0.014896612 | 0.213481393 | 2.68 |
| GOTERM_BP_FAT | GO:0006575~cellular amino acid derivative metabolic process | 0.015015335 | 1 | 2.81 |
| INTERPRO | IPR011701:Major facilitator superfamily MFS-1 | 0.015064631 | 0.999993213 | 3.49 |
| GOTERM_BP_FAT | GO:0006873~cellular ion homeostasis | 0.016109293 | 1 | 2.19 |
| INTERPRO | IPR002939:Chaperone DnaJ, C-terminal | 0.016321653 | 0.999997506 | 14.80 |
| GOTERM_BP_FAT | GO:0001818~negative regulation of cytokine production | 0.016410086 | 1 | 7.33 |
| UP_SEQ_FEATURE | domain:Kazal-like | 0.01647541 | 0.99999999 | 5.06 |
| SP_PIR_KEYWORDS | iron | 0.016995132 | 0.997723528 | 2.02 |
| GOTERM_MF_FAT | GO:0051087~chaperone binding | 0.01795566 | 0.9998994 | 14.06 |
| UP_SEQ_FEATURE | lipid moiety-binding region:N-myristoyl glycine | 0.017995842 | 0.999999998 | 2.98 |
| SMART | SM00271:DnaJ | 0.01861285 | 0.940288899 | 4.88 |
| GOTERM_BP_FAT | GO:0055082~cellular chemical homeostasis | 0.019307403 | 1 | 2.13 |
| SP_PIR_KEYWORDS | ubl conjugation | 0.019389932 | 0.999042357 | 1.74 |
| GOTERM_BP_FAT | GO:0006766~vitamin metabolic process | 0.019999084 | 1 | 3.82 |
| KEGG_PATHWAY | mmu04612:Antigen processing and presentation | 0.020421727 | 0.934360241 | 3.22 |
| INTERPRO | IPR002401:Cytochrome P450, E-class, group I | 0.020503032 | 0.999999912 | 3.81 |
| COG_ONTOLOGY | Secondary metabolites biosynthesis, transport, and catabolism | 0.020568312 | 0.282888951 | 2.77 |
| INTERPRO | IPR018253:Heat shock protein DnaJ, conserved site | 0.020930749 | 0.999999937 | 4.72 |
| SP_PIR_KEYWORDS | chylomicron | 0.021051148 | 0.999475434 | 13.00 |
| GOTERM_BP_FAT | GO:0008104~protein localization | 0.021597663 | 1 | 1.58 |
| GOTERM_MF_FAT | GO:0009055~electron carrier activity | 0.021840905 | 0.999986572 | 2.30 |
| KEGG_PATHWAY | mmu00830:Retinol metabolism | 0.021976366 | 0.946775183 | 3.70 |
| GOTERM_BP_FAT | GO:0030003~cellular cation homeostasis | 0.022523238 | 1 | 2.60 |
| SP_PIR_KEYWORDS | lyase | 0.022813714 | 0.999723329 | 2.84 |
| GOTERM_BP_FAT | GO:0015031~protein transport | 0.023585002 | 1 | 1.62 |
| UP_SEQ_FEATURE | cross-link:Glycyl lysine isopeptide (Lys-Gly) (interchain with G-Cter in SUMO) | 0.024011462 | 1 | 3.14 |
| GOTERM_MF_FAT | GO:0017076~purine nucleotide binding | 0.024576034 | 0.999996762 | 1.31 |
| GOTERM_BP_FAT | GO:0030325~adrenal gland development | 0.024608316 | 1 | 11.99 |
| GOTERM_BP_FAT | GO:0033865~nucleoside bisphosphate metabolic process | 0.024608316 | 1 | 11.99 |
| UP_SEQ_FEATURE | short sequence motif:Microbody targeting signal | 0.024656844 | 1 | 4.48 |
| GOTERM_MF_FAT | GO:0016831~carboxy-lyase activity | 0.025139056 | 0.999997585 | 6.25 |
| GOTERM_BP_FAT | GO:0045184~establishment of protein localization | 0.025370578 | 1 | 1.61 |
| GOTERM_BP_FAT | GO:0006875~cellular metal ion homeostasis | 0.026110138 | 1 | 3.08 |
| SP_PIR_KEYWORDS | transport | 0.026346903 | 0.999923527 | 1.35 |
| SP_PIR_KEYWORDS | isopeptide bond | 0.027078107 | 0.999941429 | 2.03 |
| GOTERM_BP_FAT | GO:0006721~terpenoid metabolic process | 0.027274929 | 1 | 6.07 |
| GOTERM_BP_FAT | GO:0009266~response to temperature stimulus | 0.028098933 | 1 | 4.31 |
| UP_SEQ_FEATURE | domain:J | 0.028505838 | 1 | 4.29 |
| SMART | SM00327:VWA | 0.029060326 | 0.988009504 | 3.46 |
| GOTERM_BP_FAT | GO:0051085~chaperone mediated protein folding requiring cofactor | 0.029094057 | 1 | 10.99 |
| INTERPRO | IPR001623:Heat shock protein DnaJ, N-terminal | 0.02913362 | 1 | 4.27 |
| SMART | SM00643:C345C | 0.029792876 | 0.989292974 | 10.87 |
| GOTERM_BP_FAT | GO:0043161~proteasomal ubiquitin-dependent protein catabolic process | 0.029810323 | 1 | 5.86 |
| GOTERM_BP_FAT | GO:0010498~proteasomal protein catabolic process | 0.029810323 | 1 | 5.86 |
| GOTERM_BP_FAT | GO:0034754~cellular hormone metabolic process | 0.029915355 | 1 | 4.23 |
| UP_SEQ_FEATURE | repeat:TPR 3 | 0.03022515 | 1 | 2.68 |
| GOTERM_MF_FAT | GO:0016229~steroid dehydrogenase activity | 0.030343021 | 0.999999841 | 5.82 |
| SP_PIR_KEYWORDS | golgi apparatus | 0.031635466 | 0.999988939 | 1.65 |
| SP_PIR_KEYWORDS | Flavoprotein | 0.031752847 | 0.999989405 | 2.95 |
| GOTERM_BP_FAT | GO:0055065~metal ion homeostasis | 0.03345055 | 1 | 2.90 |
| GOTERM_BP_FAT | GO:0019915~lipid storage | 0.033877293 | 1 | 10.15 |
| GOTERM_CC_FAT | GO:0070013~intracellular organelle lumen | 0.034997081 | 0.999890522 | 1.38 |
| GOTERM_BP_FAT | GO:0050801~ion homeostasis | 0.035317556 | 1 | 1.95 |
| GOTERM_BP_FAT | GO:0043085~positive regulation of catalytic activity | 0.035988628 | 1 | 2.02 |
| KEGG_PATHWAY | mmu00330:Arginine and proline metabolism | 0.036137518 | 0.992237707 | 3.95 |
| GOTERM_MF_FAT | GO:0016765~transferase activity, transferring alkyl or aryl (other than methyl) groups | 0.03622002 | 0.999999993 | 3.98 |
| GOTERM_MF_FAT | GO:0050662~coenzyme binding | 0.03628511 | 0.999999993 | 2.37 |
| GOTERM_CC_FAT | GO:0043233~organelle lumen | 0.036439042 | 0.999925342 | 1.38 |
| GOTERM_BP_FAT | GO:0006633~fatty acid biosynthetic process | 0.036724603 | 1 | 3.26 |
| GOTERM_CC_FAT | GO:0005625~soluble fraction | 0.036875796 | 0.999933524 | 2.83 |
| GOTERM_BP_FAT | GO:0051338~regulation of transferase activity | 0.037744468 | 1 | 2.21 |
| GOTERM_CC_FAT | GO:0031974~membrane-enclosed lumen | 0.037832141 | 0.999948451 | 1.37 |
| SP_PIR_KEYWORDS | lipid metabolism | 0.038024681 | 0.999998945 | 2.55 |
| SMART | SM00702:P4Hc | 0.038298067 | 0.997141927 | 9.51 |
| INTERPRO | IPR007123:Gelsolin region | 0.038322594 | 1 | 9.52 |
| INTERPRO | IPR018933:Netrin module, non-TIMP type | 0.038322594 | 1 | 9.52 |
| GOTERM_MF_FAT | GO:0019904~protein domain specific binding | 0.038710384 | 0.999999998 | 2.20 |
| GOTERM_BP_FAT | GO:0006458~'de novo' protein folding | 0.038942326 | 1 | 9.42 |
| GOTERM_BP_FAT | GO:0051084~'de novo' posttranslational protein folding | 0.038942326 | 1 | 9.42 |
| GOTERM_BP_FAT | GO:0048878~chemical homeostasis | 0.038958137 | 1 | 1.81 |
| SP_PIR_KEYWORDS | metalloprotein | 0.03979771 | 0.999999452 | 3.87 |
| GOTERM_CC_FAT | GO:0005739~mitochondrion | 0.039933928 | 0.99997055 | 1.33 |
| GOTERM_CC_FAT | GO:0031227~intrinsic to endoplasmic reticulum membrane | 0.039962332 | 0.999970772 | 3.85 |
| GOTERM_BP_FAT | GO:0009968~negative regulation of signal transduction | 0.041123561 | 1 | 2.31 |
| GOTERM_BP_FAT | GO:0006775~fat-soluble vitamin metabolic process | 0.041143794 | 1 | 5.17 |
| GOTERM_BP_FAT | GO:0043405~regulation of MAP kinase activity | 0.041932397 | 1 | 3.14 |
| GOTERM_MF_FAT | GO:0008514~organic anion transmembrane transporter activity | 0.042003417 | 1 | 9.04 |
| SP_PIR_KEYWORDS | Rotamase | 0.042807603 | 0.99999982 | 5.10 |
| GOTERM_MF_FAT | GO:0042802~identical protein binding | 0.04346889 | 1 | 1.89 |
| INTERPRO | IPR019389:Selenoprotein T | 0.04442152 | 1 | 44.41 |
| INTERPRO | IPR015310:Activator of Hsp90 ATPase, N-terminal | 0.04442152 | 1 | 44.41 |
| INTERPRO | IPR013538:Activator of Hsp90 ATPase homologue 1-like | 0.04442152 | 1 | 44.41 |
| GOTERM_CC_FAT | GO:0030173~integral to Golgi membrane | 0.044436074 | 0.999991159 | 5.01 |
| GOTERM_CC_FAT | GO:0031228~intrinsic to Golgi membrane | 0.044436074 | 0.999991159 | 5.01 |
| GOTERM_BP_FAT | GO:0032764~negative regulation of mast cell cytokine production | 0.044821953 | 1 | 43.97 |
| SP_PIR_KEYWORDS | transmembrane protein | 0.046679701 | 0.999999957 | 1.76 |
| GOTERM_MF_FAT | GO:0030235~nitric-oxide synthase regulator activity | 0.04670403 | 1 | 42.18 |
| GOTERM_MF_FAT | GO:0001671~ATPase activator activity | 0.04670403 | 1 | 42.18 |
| SP_PIR_KEYWORDS | glycoprotein | 0.046979986 | 0.999999962 | 1.18 |
| UP_SEQ_FEATURE | repeat:TPR 1 | 0.047091099 | 1 | 2.43 |
| UP_SEQ_FEATURE | repeat:TPR 2 | 0.047091099 | 1 | 2.43 |
| UP_SEQ_FEATURE | binding site:2-oxoglutarate | 0.047468959 | 1 | 8.46 |
| GOTERM_CC_FAT | GO:0030176~integral to endoplasmic reticulum membrane | 0.048009722 | 0.999996612 | 4.86 |
| INTERPRO | IPR002035:von Willebrand factor, type A | 0.048113709 | 1 | 3.03 |
| GOTERM_MF_FAT | GO:0003755~peptidyl-prolyl cis-trans isomerase activity | 0.049049755 | 1 | 4.82 |
| INTERPRO | IPR006620:Prolyl 4-hydroxylase, alpha subunit | 0.049075325 | 1 | 8.33 |
| COG_ONTOLOGY | Amino acid transport and metabolism | 0.049270327 | 0.554433244 | 3.50 |
| GOTERM_MF_FAT | GO:0032555~purine ribonucleotide binding | 0.049540539 | 1 | 1.27 |
| GOTERM_MF_FAT | GO:0032553~ribonucleotide binding | 0.049540539 | 1 | 1.27 |
| GOTERM_BP_FAT | GO:0030307~positive regulation of cell growth | 0.049857745 | 1 | 8.25 |
| GOTERM_BP_FAT | GO:0030512~negative regulation of transforming growth factor beta receptor signaling pathway | 0.049857745 | 1 | 8.25 |
| UP_SEQ_FEATURE | site:Lowers pKa of C-terminal Cys of second active site | 0.049921136 | 1 | 39.46 |
| UP_SEQ_FEATURE | site:Lowers pKa of C-terminal Cys of first active site | 0.049921136 | 1 | 39.46 |
| PIR_SUPERFAMILY | PIRSF500265:vesicular integral-membrane protein VIP36 | 0.050719786 | 0.99999561 | 38.74 |
| GOTERM_CC_FAT | GO:0031980~mitochondrial lumen | 0.050955389 | 0.999998468 | 2.21 |
| GOTERM_CC_FAT | GO:0005759~mitochondrial matrix | 0.050955389 | 0.999998468 | 2.21 |
| GOTERM_CC_FAT | GO:0005903~brush border | 0.055564749 | 0.999999559 | 4.58 |
| GOTERM_BP_FAT | GO:0042573~retinoic acid metabolic process | 0.055679441 | 1 | 7.76 |
| GOTERM_BP_FAT | GO:0070085~glycosylation | 0.055738153 | 1 | 2.90 |
| GOTERM_BP_FAT | GO:0006874~cellular calcium ion homeostasis | 0.055738153 | 1 | 2.90 |
| GOTERM_BP_FAT | GO:0043413~biopolymer glycosylation | 0.055738153 | 1 | 2.90 |
| GOTERM_BP_FAT | GO:0006486~protein amino acid glycosylation | 0.055738153 | 1 | 2.90 |
| GOTERM_MF_FAT | GO:0016859~cis-trans isomerase activity | 0.056282496 | 1 | 4.56 |
| KEGG_PATHWAY | mmu00650:Butanoate metabolism | 0.056363179 | 0.999527672 | 4.53 |
| SP_PIR_KEYWORDS | hdl | 0.057254718 | 0.999999999 | 7.65 |
| GOTERM_BP_FAT | GO:0055080~cation homeostasis | 0.058536226 | 1 | 2.15 |
| GOTERM_BP_FAT | GO:0006631~fatty acid metabolic process | 0.058536226 | 1 | 2.15 |
| UP_SEQ_FEATURE | nucleotide phosphate-binding region:FAD | 0.058746631 | 1 | 3.40 |
| GOTERM_BP_FAT | GO:0051605~protein maturation by peptide bond cleavage | 0.059752402 | 1 | 3.38 |
| GOTERM_MF_FAT | GO:0004653~polypeptide N-acetylgalactosaminyltransferase activity | 0.059945204 | 1 | 7.44 |
| GOTERM_MF_FAT | GO:0016878~acid-thiol ligase activity | 0.059945204 | 1 | 7.44 |
| BIOCARTA | m_cblPathway:CBL mediated ligand-induced downregulation of EGF receptors | 0.060495285 | 0.982684595 | 7.14 |
| GOTERM_BP_FAT | GO:0010648~negative regulation of cell communication | 0.06156674 | 1 | 2.13 |
| GOTERM_BP_FAT | GO:0045859~regulation of protein kinase activity | 0.06156674 | 1 | 2.13 |
| KEGG_PATHWAY | mmu05215:Prostate cancer | 0.061672158 | 0.999775711 | 2.79 |
| GOTERM_MF_FAT | GO:0030145~manganese ion binding | 0.061710379 | 1 | 2.28 |
| SP_PIR_KEYWORDS | vitamin c | 0.063456381 | 1 | 7.22 |
| GOTERM_BP_FAT | GO:0055074~calcium ion homeostasis | 0.064672714 | 1 | 2.78 |
| SP_PIR_KEYWORDS | disulfide bond | 0.064994312 | 1 | 1.21 |
| INTERPRO | IPR004113:FAD-linked oxidase, C-terminal | 0.065888544 | 1 | 29.61 |
| INTERPRO | IPR007599:Der1-like | 0.065888544 | 1 | 29.61 |
| INTERPRO | IPR000952:Uncharacterised protein family UPF0017, hydrolase-like, conserved site | 0.065888544 | 1 | 29.61 |
| INTERPRO | IPR013547:Prolyl 4-hydroxylase alpha-subunit, N-terminal | 0.065888544 | 1 | 29.61 |
| GOTERM_BP_FAT | GO:0042325~regulation of phosphorylation | 0.066210522 | 1 | 1.82 |
| GOTERM_BP_FAT | GO:0002719~negative regulation of cytokine production during immune response | 0.066476218 | 1 | 29.32 |
| GOTERM_BP_FAT | GO:0032763~regulation of mast cell cytokine production | 0.066476218 | 1 | 29.32 |
| GOTERM_BP_FAT | GO:0045829~negative regulation of isotype switching | 0.066476218 | 1 | 29.32 |
| GOTERM_BP_FAT | GO:0010744~positive regulation of foam cell differentiation | 0.066476218 | 1 | 29.32 |
| GOTERM_BP_FAT | GO:0030970~retrograde protein transport, ER to cytosol | 0.066476218 | 1 | 29.32 |
| GOTERM_BP_FAT | GO:0002701~negative regulation of production of molecular mediator of immune response | 0.066476218 | 1 | 29.32 |
| GOTERM_BP_FAT | GO:0048294~negative regulation of isotype switching to IgE isotypes | 0.066476218 | 1 | 29.32 |
| SP_PIR_KEYWORDS | gpi-anchor | 0.067013555 | 1 | 2.45 |
| GOTERM_BP_FAT | GO:0051604~protein maturation | 0.067024794 | 1 | 2.75 |
| GOTERM_BP_FAT | GO:0045793~positive regulation of cell size | 0.067982895 | 1 | 6.94 |
| GOTERM_BP_FAT | GO:0045428~regulation of nitric oxide biosynthetic process | 0.067982895 | 1 | 6.94 |
| KEGG_PATHWAY | mmu00380:Tryptophan metabolism | 0.068137397 | 0.999909957 | 4.19 |
| GOTERM_MF_FAT | GO:0030911~TPR domain binding | 0.069234132 | 1 | 28.12 |
| GOTERM_MF_FAT | GO:0004586~ornithine decarboxylase activity | 0.069234132 | 1 | 28.12 |
| GOTERM_BP_FAT | GO:0022604~regulation of cell morphogenesis | 0.069424019 | 1 | 2.72 |
| GOTERM_BP_FAT | GO:0010876~lipid localization | 0.071104162 | 1 | 2.40 |
| GOTERM_BP_FAT | GO:0043549~regulation of kinase activity | 0.071212389 | 1 | 2.06 |
| INTERPRO | IPR001134:Netrin domain | 0.073303434 | 1 | 6.66 |
| GOTERM_MF_FAT | GO:0030554~adenyl nucleotide binding | 0.073822231 | 1 | 1.26 |
| GOTERM_CC_FAT | GO:0031225~anchored to membrane | 0.074222968 | 0.999999997 | 1.94 |
| GOTERM_BP_FAT | GO:0001764~neuron migration | 0.074272874 | 1 | 3.14 |
| GOTERM_BP_FAT | GO:0050818~regulation of coagulation | 0.074438949 | 1 | 6.60 |
| UP_SEQ_FEATURE | region of interest:Catalytic subdomain A | 0.074736022 | 1 | 6.58 |
| UP_SEQ_FEATURE | region of interest:Catalytic subdomain B | 0.074736022 | 1 | 6.58 |
| PIR_SUPERFAMILY | PIRSF000320:procollagen-proline,2-oxoglutarate 4-dioxygenase alpha chain | 0.075111176 | 0.999999991 | 25.83 |
| GOTERM_BP_FAT | GO:0006820~anion transport | 0.075391074 | 1 | 2.37 |
| GOTERM_BP_FAT | GO:0051336~regulation of hydrolase activity | 0.078183709 | 1 | 2.02 |
| GOTERM_MF_FAT | GO:0031418~L-ascorbic acid binding | 0.079995787 | 1 | 6.33 |
| GOTERM_BP_FAT | GO:0030518~steroid hormone receptor signaling pathway | 0.081081528 | 1 | 6.28 |
| GOTERM_BP_FAT | GO:0019220~regulation of phosphate metabolic process | 0.081668006 | 1 | 1.75 |
| GOTERM_BP_FAT | GO:0051174~regulation of phosphorus metabolic process | 0.081668006 | 1 | 1.75 |
| GOTERM_MF_FAT | GO:0001883~purine nucleoside binding | 0.08206996 | 1 | 1.25 |
| GOTERM_BP_FAT | GO:0044271~nitrogen compound biosynthetic process | 0.083225518 | 1 | 1.75 |
| SP_PIR_KEYWORDS | decarboxylase | 0.083294843 | 1 | 6.19 |
| SP_PIR_KEYWORDS | nucleotidyltransferase | 0.08425719 | 1 | 3.01 |
| GOTERM_BP_FAT | GO:0030005~cellular di-, tri-valent inorganic cation homeostasis | 0.084396307 | 1 | 2.30 |
| SP_PIR_KEYWORDS | phosphoprotein | 0.08440291 | 1 | 1.10 |
| GOTERM_BP_FAT | GO:0032101~regulation of response to external stimulus | 0.084799158 | 1 | 2.56 |
| GOTERM_MF_FAT | GO:0016712~oxidoreductase activity, acting on paired donors, with incorporation or reduction of molecular oxygen, reduced flavin or flavoprotein as one donor, and incorporation of one atom of oxygen | 0.085227275 | 1 | 3.83 |
| KEGG_PATHWAY | mmu00520:Amino sugar and nucleotide sugar metabolism | 0.085412188 | 0.999992384 | 3.81 |
| GOTERM_BP_FAT | GO:0051347~positive regulation of transferase activity | 0.086736898 | 1 | 2.28 |
| INTERPRO | IPR005052:Legume-like lectin | 0.086874494 | 1 | 22.20 |
| INTERPRO | IPR002433:Ornithine decarboxylase | 0.086874494 | 1 | 22.20 |
| INTERPRO | IPR000074:Apolipoprotein A1/A4/E | 0.086874494 | 1 | 22.20 |
| INTERPRO | IPR016168:FAD-linked oxidase, FAD-binding, subdomain 2 | 0.086874494 | 1 | 22.20 |
| INTERPRO | IPR013326:Apolipoprotein/apolipophorin | 0.086874494 | 1 | 22.20 |
| INTERPRO | IPR012020:AB-hydrolase YheT, putative | 0.086874494 | 1 | 22.20 |
| INTERPRO | IPR004821:Cytidyltransferase-related | 0.086874494 | 1 | 22.20 |
| GOTERM_MF_FAT | GO:0050661~NADP or NADPH binding | 0.087081706 | 1 | 6.03 |
| SP_PIR_KEYWORDS | nucleotide-binding | 0.087353674 | 1 | 1.25 |
| GOTERM_BP_FAT | GO:0002829~negative regulation of T-helper 2 type immune response | 0.087641129 | 1 | 21.99 |
| GOTERM_BP_FAT | GO:0010884~positive regulation of lipid storage | 0.087641129 | 1 | 21.99 |
| GOTERM_BP_FAT | GO:0010743~regulation of foam cell differentiation | 0.087641129 | 1 | 21.99 |
| GOTERM_BP_FAT | GO:0030300~regulation of intestinal cholesterol absorption | 0.087641129 | 1 | 21.99 |
| GOTERM_BP_FAT | GO:0008299~isoprenoid biosynthetic process | 0.087898924 | 1 | 6.00 |
| GOTERM_MF_FAT | GO:0048037~cofactor binding | 0.088315759 | 1 | 1.87 |
| GOTERM_MF_FAT | GO:0001882~nucleoside binding | 0.088841657 | 1 | 1.25 |
| GOTERM_BP_FAT | GO:0044093~positive regulation of molecular function | 0.089241827 | 1 | 1.72 |
| UP_SEQ_FEATURE | domain:NTR | 0.089830649 | 1 | 5.92 |
| KEGG_PATHWAY | mmu00071:Fatty acid metabolism | 0.089996405 | 0.999996076 | 3.72 |
| INTERPRO | IPR000086:NUDIX hydrolase domain | 0.093463462 | 1 | 5.79 |
| GOTERM_BP_FAT | GO:0042157~lipoprotein metabolic process | 0.093819374 | 1 | 2.89 |
| SP_PIR_KEYWORDS | nad | 0.096153091 | 1 | 2.05 |
| UP_SEQ_FEATURE | domain:IQ 6 | 0.097355998 | 1 | 19.73 |
| UP_SEQ_FEATURE | domain:L-type lectin-like | 0.097355998 | 1 | 19.73 |
| UP_SEQ_FEATURE | domain:Fe2OG dioxygenase | 0.097685684 | 1 | 5.64 |
| PIR_SUPERFAMILY | PIRSF005211:alpha/beta hydrolase, YheT type | 0.09887876 | 1 | 19.37 |
| PIR_SUPERFAMILY | PIRSF005211:Ab_hydro_YheT | 0.09887876 | 1 | 19.37 |
| PIR_SUPERFAMILY | PIRSF017635:L-type lectin, animal type | 0.09887876 | 1 | 19.37 |

**Suppl. Table S14** Functional annotation terms associated with genes exhibiting DYSregulation in diabetes relative to control, and amelioration (reversion toward control expression level) with losartan treatment (n = 52 annotation terms; ranked by ascending p-value)

| **Category** | **Term** | **PValue** | **Bonferroni** | **Fold Enrichment** |
| --- | --- | --- | --- | --- |
| SP_PIR_KEYWORDS | stress response | 1.99E-09 | 3.23E-07 | 36.07 |
| GOTERM_CC_FAT | GO:0005788~endoplasmic reticulum lumen | 1.55E-06 | 1.86E-04 | 18.99 |
| INTERPRO | IPR013126:Heat shock protein 70 | 1.70E-05 | 0.0037777 | 75.11 |
| INTERPRO | IPR001023:Heat shock protein Hsp70 | 1.70E-05 | 0.0037777 | 75.11 |
| INTERPRO | IPR018181:Heat shock protein 70, conserved site | 2.92E-05 | 0.006493991 | 63.55 |
| GOTERM_MF_FAT | GO:0019904~protein domain specific binding | 5.97E-05 | 0.011154506 | 7.91 |
| UP_SEQ_FEATURE | short sequence motif:Prevents secretion from ER | 2.94E-04 | 0.087661261 | 15.43 |
| GOTERM_MF_FAT | GO:0051082~unfolded protein binding | 4.72E-04 | 0.084871031 | 13.56 |
| SP_PIR_KEYWORDS | Chaperone | 8.32E-04 | 0.126113309 | 8.12 |
| PIR_SUPERFAMILY | PIRSF002581:chaperone HSP70 | 9.33E-04 | 0.057992221 | 62.27 |
| GOTERM_CC_FAT | GO:0044432~endoplasmic reticulum part | 0.001051164 | 0.118566753 | 5.92 |
| INTERPRO | IPR000886:Endoplasmic reticulum, targeting sequence | 0.001136617 | 0.224005411 | 19.21 |
| GOTERM_CC_FAT | GO:0070013~intracellular organelle lumen | 0.001265354 | 0.140959029 | 2.59 |
| GOTERM_CC_FAT | GO:0043233~organelle lumen | 0.001298106 | 0.144332905 | 2.58 |
| SP_PIR_KEYWORDS | acetylation | 0.001436158 | 0.207707706 | 2.01 |
| SP_PIR_KEYWORDS | endoplasmic reticulum | 0.001675237 | 0.237853142 | 3.29 |
| GOTERM_CC_FAT | GO:0031974~membrane-enclosed lumen | 0.001779049 | 0.192389991 | 2.50 |
| SP_PIR_KEYWORDS | molecular chaperone | 0.00205625 | 0.283556207 | 43.48 |
| GOTERM_CC_FAT | GO:0005783~endoplasmic reticulum | 0.002844546 | 0.289532388 | 2.80 |
| GOTERM_BP_FAT | GO:0006457~protein folding | 0.004818544 | 0.967906301 | 7.23 |
| KEGG_PATHWAY | mmu04612:Antigen processing and presentation | 0.005431568 | 0.195757859 | 10.51 |
| GOTERM_CC_FAT | GO:0048770~pigment granule | 0.008977972 | 0.661158231 | 9.19 |
| GOTERM_CC_FAT | GO:0042470~melanosome | 0.008977972 | 0.661158231 | 9.19 |
| SP_PIR_KEYWORDS | phosphoprotein | 0.015402577 | 0.919107445 | 1.35 |
| SP_PIR_KEYWORDS | heat shock | 0.019351038 | 0.957811042 | 101.44 |
| SP_PIR_KEYWORDS | stress-induced protein | 0.019351038 | 0.957811042 | 101.44 |
| GOTERM_MF_FAT | GO:0015293~symporter activity | 0.031213932 | 0.997424614 | 5.75 |
| GOTERM_MF_FAT | GO:0004857~enzyme inhibitor activity | 0.037437583 | 0.999233286 | 3.91 |
| SP_PIR_KEYWORDS | ubl conjugation | 0.042716988 | 0.999151724 | 2.71 |
| GOTERM_BP_FAT | GO:0030003~cellular cation homeostasis | 0.048386653 | 1 | 4.83 |
| GOTERM_MF_FAT | GO:0008238~exopeptidase activity | 0.051189869 | 0.999948748 | 8.14 |
| GOTERM_BP_FAT | GO:0060284~regulation of cell development | 0.054000137 | 1 | 4.62 |
| GOTERM_CC_FAT | GO:0005829~cytosol | 0.057465767 | 0.999176515 | 2.49 |
| GOTERM_BP_FAT | GO:0030036~actin cytoskeleton organization | 0.05905062 | 1 | 4.45 |
| GOTERM_BP_FAT | GO:0051085~chaperone mediated protein folding requiring cofactor | 0.062622032 | 1 | 30.60 |
| GOTERM_BP_FAT | GO:0033554~cellular response to stress | 0.065899107 | 1 | 2.73 |
| GOTERM_BP_FAT | GO:0030029~actin filament-based process | 0.068866232 | 1 | 4.17 |
| GOTERM_BP_FAT | GO:0006458~'de novo' protein folding | 0.072676117 | 1 | 26.23 |
| GOTERM_BP_FAT | GO:0051084~'de novo' posttranslational protein folding | 0.072676117 | 1 | 26.23 |
| GOTERM_BP_FAT | GO:0055080~cation homeostasis | 0.076441884 | 1 | 3.99 |
| UP_SEQ_FEATURE | cross-link:Glycyl lysine isopeptide (Lys-Gly) (interchain with G-Cter in SUMO) | 0.082714523 | 1 | 6.21 |
| SMART | SM00387:HATPase_c | 0.084145506 | 0.982460463 | 22.27 |
| GOTERM_CC_FAT | GO:0045120~pronucleus | 0.086966072 | 0.999981869 | 21.71 |
| KEGG_PATHWAY | mmu03040:Spliceosome | 0.087296182 | 0.974106266 | 5.78 |
| GOTERM_MF_FAT | GO:0000166~nucleotide binding | 0.091293443 | 0.999999985 | 1.48 |
| INTERPRO | IPR003594:ATP-binding region, ATPase-like | 0.091523029 | 0.999999999 | 20.65 |
| SP_PIR_KEYWORDS | methylation | 0.093383925 | 0.999999873 | 3.67 |
| SP_PIR_KEYWORDS | atp-binding | 0.09533277 | 0.999999911 | 1.73 |
| SP_PIR_KEYWORDS | nucleotide-binding | 0.097255735 | 0.999999937 | 1.62 |
| GOTERM_BP_FAT | GO:0045428~regulation of nitric oxide biosynthetic process | 0.097348357 | 1 | 19.33 |
| GOTERM_BP_FAT | GO:0007010~cytoskeleton organization | 0.098124703 | 1 | 2.82 |
| SP_PIR_KEYWORDS | Symport | 0.098976483 | 0.999999954 | 5.58 |

**Suppl. Table S15** Functional annotation terms associated with genes exhibiting DYSregulation in diabetes relative to control, and unaffected by losartan treatment (n = 219 annotation terms; ranked by ascending p-value)

| **Category** | **Term** | **PValue** | **Bonferroni** | **Fold Enrichment** |
| --- | --- | --- | --- | --- |
| KEGG_PATHWAY | mmu00982:Drug metabolism | 2.60E-09 | 2.16E-07 | 11.92 |
| SP_PIR_KEYWORDS | oxidoreductase | 1.58E-07 | 4.35E-05 | 3.67 |
| INTERPRO | IPR003080:Glutathione S-transferase, alpha class | 2.08E-07 | 9.70E-05 | 75.52 |
| GOTERM_BP_FAT | GO:0055114~oxidation reduction | 1.53E-06 | 0.001523006 | 3.08 |
| INTERPRO | IPR004045:Glutathione S-transferase, N-terminal | 1.00E-05 | 0.00468109 | 20.14 |
| SP_PIR_KEYWORDS | lyase | 1.07E-05 | 0.00294115 | 7.17 |
| INTERPRO | IPR004046:Glutathione S-transferase, C-terminal | 1.21E-05 | 0.005645419 | 19.42 |
| GOTERM_MF_FAT | GO:0004364~glutathione transferase activity | 1.27E-05 | 0.005123781 | 19.05 |
| GOTERM_CC_FAT | GO:0005792~microsome | 1.91E-05 | 0.003096827 | 5.79 |
| KEGG_PATHWAY | mmu00980:Metabolism of xenobiotics by cytochrome P450 | 2.25E-05 | 0.001867613 | 9.03 |
| GOTERM_CC_FAT | GO:0042598~vesicular fraction | 2.56E-05 | 0.004135341 | 5.60 |
| KEGG_PATHWAY | mmu00830:Retinol metabolism | 2.74E-05 | 0.002275713 | 8.77 |
| INTERPRO | IPR017933:Glutathione S-transferase/chloride channel, C-terminal | 3.25E-05 | 0.015080108 | 15.99 |
| GOTERM_BP_FAT | GO:0016054~organic acid catabolic process | 5.19E-05 | 0.050214764 | 8.18 |
| GOTERM_BP_FAT | GO:0046395~carboxylic acid catabolic process | 5.19E-05 | 0.050214764 | 8.18 |
| GOTERM_BP_FAT | GO:0006631~fatty acid metabolic process | 7.48E-05 | 0.071542111 | 4.95 |
| PIR_SUPERFAMILY | PIRSF000503:glutathione transferase | 8.43E-05 | 0.010143397 | 20.39 |
| GOTERM_MF_FAT | GO:0019842~vitamin binding | 1.05E-04 | 0.041626763 | 6.14 |
| UP_SEQ_FEATURE | domain:GST N-terminal | 1.36E-04 | 0.077557811 | 18.39 |
| SP_PIR_KEYWORDS | microsome | 1.45E-04 | 0.039130944 | 7.00 |
| GOTERM_BP_FAT | GO:0006766~vitamin metabolic process | 1.75E-04 | 0.159126933 | 8.41 |
| KEGG_PATHWAY | mmu00340:Histidine metabolism | 2.91E-04 | 0.023879276 | 14.90 |
| INTERPRO | IPR010987:Glutathione S-transferase, C-terminal-like | 3.09E-04 | 0.134240299 | 15.10 |
| GOTERM_BP_FAT | GO:0051186~cofactor metabolic process | 3.48E-04 | 0.292055706 | 4.55 |
| GOTERM_BP_FAT | GO:0006576~biogenic amine metabolic process | 3.93E-04 | 0.322867559 | 7.25 |
| GOTERM_MF_FAT | GO:0016765~transferase activity, transferring alkyl or aryl (other than methyl) groups | 4.28E-04 | 0.158459734 | 9.34 |
| SP_PIR_KEYWORDS | pyridoxal phosphate | 5.95E-04 | 0.150926102 | 8.75 |
| UP_SEQ_FEATURE | domain:GST C-terminal | 6.86E-04 | 0.333981212 | 12.26 |
| GOTERM_BP_FAT | GO:0006775~fat-soluble vitamin metabolic process | 6.99E-04 | 0.500654681 | 12.18 |
| SP_PIR_KEYWORDS | peroxisome | 7.96E-04 | 0.196617772 | 6.38 |
| GOTERM_CC_FAT | GO:0042579~microbody | 8.70E-04 | 0.131563103 | 6.23 |
| GOTERM_CC_FAT | GO:0005777~peroxisome | 8.70E-04 | 0.131563103 | 6.23 |
| INTERPRO | IPR002401:Cytochrome P450, E-class, group I | 0.001028606 | 0.381591491 | 7.77 |
| GOTERM_BP_FAT | GO:0009310~amine catabolic process | 0.001080101 | 0.658060064 | 7.65 |
| INTERPRO | IPR018170:Aldo/keto reductase, conserved site | 0.001108885 | 0.40437059 | 19.08 |
| INTERPRO | IPR012335:Thioredoxin fold | 0.001113647 | 0.405695268 | 5.98 |
| GOTERM_CC_FAT | GO:0005624~membrane fraction | 0.001131379 | 0.167554173 | 2.72 |
| SP_PIR_KEYWORDS | Monooxygenase | 0.001468236 | 0.332396929 | 5.67 |
| GOTERM_CC_FAT | GO:0005576~extracellular region | 0.001484649 | 0.213916202 | 1.76 |
| GOTERM_BP_FAT | GO:0006575~cellular amino acid derivative metabolic process | 0.001549145 | 0.785510251 | 4.70 |
| GOTERM_CC_FAT | GO:0005626~insoluble fraction | 0.001570789 | 0.224826084 | 2.63 |
| GOTERM_MF_FAT | GO:0048037~cofactor binding | 0.001662347 | 0.488538752 | 3.65 |
| GOTERM_BP_FAT | GO:0006732~coenzyme metabolic process | 0.001679623 | 0.811615071 | 4.64 |
| GOTERM_MF_FAT | GO:0016712~oxidoreductase activity, acting on paired donors, with incorporation or reduction of molecular oxygen, reduced flavin or flavoprotein as one donor, and incorporation of one atom of oxygen | 0.001889321 | 0.533322339 | 9.38 |
| INTERPRO | IPR017973:Cytochrome P450, C-terminal region | 0.001976335 | 0.603016941 | 6.71 |
| UP_SEQ_FEATURE | short sequence motif:Microbody targeting signal | 0.002049462 | 0.703149468 | 9.19 |
| GOTERM_BP_FAT | GO:0046700~heterocycle catabolic process | 0.002200447 | 0.887797666 | 9.01 |
| GOTERM_BP_FAT | GO:0008610~lipid biosynthetic process | 0.002316674 | 0.90005413 | 3.20 |
| SMART | SM00099:btg1 | 0.002366357 | 0.197747607 | 39.36 |
| INTERPRO | IPR001128:Cytochrome P450 | 0.002703664 | 0.71756899 | 6.25 |
| INTERPRO | IPR017972:Cytochrome P450, conserved site | 0.002984268 | 0.752349467 | 6.11 |
| SP_PIR_KEYWORDS | Secreted | 0.002984461 | 0.560428097 | 1.79 |
| GOTERM_MF_FAT | GO:0009055~electron carrier activity | 0.003077173 | 0.711197662 | 3.68 |
| INTERPRO | IPR002087:Anti-proliferative protein | 0.003214267 | 0.777644996 | 33.99 |
| GOTERM_MF_FAT | GO:0016289~CoA hydrolase activity | 0.003243753 | 0.730006473 | 13.21 |
| GOTERM_CC_FAT | GO:0045177~apical part of cell | 0.00341443 | 0.425401735 | 4.77 |
| GOTERM_BP_FAT | GO:0034754~cellular hormone metabolic process | 0.003453271 | 0.96777563 | 7.97 |
| GOTERM_CC_FAT | GO:0005783~endoplasmic reticulum | 0.003591361 | 0.441693754 | 2.10 |
| GOTERM_BP_FAT | GO:0006776~vitamin A metabolic process | 0.003597817 | 0.972098485 | 12.75 |
| GOTERM_CC_FAT | GO:0044421~extracellular region part | 0.003719894 | 0.453240631 | 2.15 |
| KEGG_PATHWAY | mmu03320:PPAR signaling pathway | 0.00375226 | 0.268035536 | 5.66 |
| GOTERM_CC_FAT | GO:0044459~plasma membrane part | 0.003845931 | 0.464332645 | 1.70 |
| GOTERM_BP_FAT | GO:0009063~cellular amino acid catabolic process | 0.003960078 | 0.980554973 | 7.67 |
| GOTERM_MF_FAT | GO:0030170~pyridoxal phosphate binding | 0.004010957 | 0.802035176 | 7.64 |
| GOTERM_MF_FAT | GO:0070279~vitamin B6 binding | 0.004010957 | 0.802035176 | 7.64 |
| INTERPRO | IPR006862:Acyl-CoA thioester hydrolase/bile acid-CoA amino acid N-acetyltransferase | 0.004102847 | 0.853388788 | 30.21 |
| INTERPRO | IPR014940:BAAT/Acyl-CoA thioester hydrolase C-terminal | 0.004102847 | 0.853388788 | 30.21 |
| INTERPRO | IPR016662:Acyl-CoA thioesterase, long chain | 0.004102847 | 0.853388788 | 30.21 |
| GOTERM_CC_FAT | GO:0016324~apical plasma membrane | 0.004196487 | 0.494021596 | 5.61 |
| GOTERM_BP_FAT | GO:0016101~diterpenoid metabolic process | 0.004454543 | 0.988124081 | 11.84 |
| GOTERM_BP_FAT | GO:0001523~retinoid metabolic process | 0.004454543 | 0.988124081 | 11.84 |
| GOTERM_BP_FAT | GO:0042445~hormone metabolic process | 0.004523993 | 0.988918937 | 5.52 |
| SP_PIR_KEYWORDS | lipid metabolism | 0.004645651 | 0.722110274 | 4.50 |
| GOTERM_CC_FAT | GO:0000267~cell fraction | 0.004709324 | 0.534532262 | 2.33 |
| SP_PIR_KEYWORDS | endoplasmic reticulum | 0.00474298 | 0.729483618 | 2.19 |
| KEGG_PATHWAY | mmu00480:Glutathione metabolism | 0.004757494 | 0.326868298 | 7.17 |
| GOTERM_BP_FAT | GO:0006721~terpenoid metabolic process | 0.004925246 | 0.992574629 | 11.43 |
| GOTERM_BP_FAT | GO:0009636~response to toxin | 0.004925246 | 0.992574629 | 11.43 |
| GOTERM_BP_FAT | GO:0009110~vitamin biosynthetic process | 0.004925246 | 0.992574629 | 11.43 |
| KEGG_PATHWAY | mmu01040:Biosynthesis of unsaturated fatty acids | 0.005197463 | 0.351124508 | 11.04 |
| PIR_SUPERFAMILY | PIRSF016521:acyl-CoA thioesterase (hydrolase) | 0.005494867 | 0.486606308 | 25.83 |
| PIR_SUPERFAMILY | PIRSF016521:Acyl-CoA_hydro | 0.005494867 | 0.486606308 | 25.83 |
| SP_PIR_KEYWORDS | carboxypeptidase | 0.005635171 | 0.788610902 | 10.94 |
| SP_PIR_KEYWORDS | heme | 0.00572059 | 0.793546309 | 4.31 |
| GOTERM_CC_FAT | GO:0031090~organelle membrane | 0.005779862 | 0.609002502 | 2.06 |
| SP_PIR_KEYWORDS | Flavoprotein | 0.006441744 | 0.830890676 | 5.10 |
| UP_SEQ_FEATURE | binding site:Substrate | 0.007073148 | 0.985037115 | 2.94 |
| GOTERM_BP_FAT | GO:0006261~DNA-dependent DNA replication | 0.007721194 | 0.999545766 | 9.75 |
| SP_PIR_KEYWORDS | transferase | 0.007892636 | 0.886855067 | 1.71 |
| GOTERM_MF_FAT | GO:0020037~heme binding | 0.007965043 | 0.960155227 | 4.01 |
| GOTERM_MF_FAT | GO:0004180~carboxypeptidase activity | 0.008455409 | 0.967353497 | 9.43 |
| GOTERM_MF_FAT | GO:0005506~iron ion binding | 0.008618377 | 0.96944599 | 2.65 |
| GOTERM_BP_FAT | GO:0046470~phosphatidylcholine metabolic process | 0.008723038 | 0.99983341 | 20.71 |
| GOTERM_CC_FAT | GO:0005615~extracellular space | 0.0087491 | 0.759152402 | 2.36 |
| COG_ONTOLOGY | Secondary metabolites biosynthesis, transport, and catabolism | 0.008942731 | 0.085913046 | 4.32 |
| GOTERM_BP_FAT | GO:0019748~secondary metabolic process | 0.009427777 | 0.999917787 | 6.00 |
| GOTERM_MF_FAT | GO:0046906~tetrapyrrole binding | 0.009944003 | 0.982181022 | 3.83 |
| SP_PIR_KEYWORDS | glycoprotein | 0.01021067 | 0.940535043 | 1.36 |
| INTERPRO | IPR015421:Pyridoxal phosphate-dependent transferase, major region, subdomain 1 | 0.010223539 | 0.991761738 | 8.84 |
| SP_PIR_KEYWORDS | iron | 0.011387091 | 0.957122818 | 2.73 |
| SP_PIR_KEYWORDS | nad | 0.012907829 | 0.971921781 | 3.63 |
| GOTERM_MF_FAT | GO:0016290~palmitoyl-CoA hydrolase activity | 0.013650154 | 0.996069159 | 16.51 |
| GOTERM_MF_FAT | GO:0016836~hydro-lyase activity | 0.01395191 | 0.996525176 | 7.86 |
| GOTERM_CC_FAT | GO:0031226~intrinsic to plasma membrane | 0.015406153 | 0.919155028 | 2.18 |
| KEGG_PATHWAY | mmu00380:Tryptophan metabolism | 0.015528519 | 0.7271898 | 7.45 |
| SP_PIR_KEYWORDS | calcium binding | 0.016272222 | 0.989019878 | 7.45 |
| KEGG_PATHWAY | mmu04610:Complement and coagulation cascades | 0.017006613 | 0.759176152 | 4.97 |
| GOTERM_BP_FAT | GO:0042573~retinoic acid metabolic process | 0.017283695 | 0.99999997 | 14.62 |
| GOTERM_CC_FAT | GO:0016323~basolateral plasma membrane | 0.017802363 | 0.945522222 | 3.94 |
| GOTERM_BP_FAT | GO:0032774~RNA biosynthetic process | 0.017864999 | 0.999999983 | 3.95 |
| GOTERM_BP_FAT | GO:0010817~regulation of hormone levels | 0.018991406 | 0.999999995 | 3.88 |
| GOTERM_BP_FAT | GO:0042439~ethanolamine and derivative metabolic process | 0.019292738 | 0.999999996 | 13.81 |
| GOTERM_BP_FAT | GO:0019637~organophosphate metabolic process | 0.019414749 | 0.999999996 | 3.30 |
| GOTERM_BP_FAT | GO:0046486~glycerolipid metabolic process | 0.019571755 | 0.999999997 | 3.85 |
| GOTERM_BP_FAT | GO:0006720~isoprenoid metabolic process | 0.020869609 | 0.999999999 | 6.76 |
| SP_PIR_KEYWORDS | nadp | 0.023202052 | 0.998428541 | 3.70 |
| GOTERM_BP_FAT | GO:0006637~acyl-CoA metabolic process | 0.023587158 | 1 | 12.43 |
| GOTERM_MF_FAT | GO:0016291~acyl-CoA thioesterase activity | 0.023750143 | 0.999937902 | 12.38 |
| INTERPRO | IPR001395:Aldo/keto reductase | 0.023978215 | 0.999988044 | 12.36 |
| GOTERM_BP_FAT | GO:0016053~organic acid biosynthetic process | 0.027457711 | 1 | 3.53 |
| GOTERM_BP_FAT | GO:0046394~carboxylic acid biosynthetic process | 0.027457711 | 1 | 3.53 |
| GOTERM_MF_FAT | GO:0015297~antiporter activity | 0.028483611 | 0.999991242 | 6.00 |
| SP_PIR_KEYWORDS | Aminotransferase | 0.03013112 | 0.999778139 | 10.94 |
| GOTERM_BP_FAT | GO:0015918~sterol transport | 0.030682947 | 1 | 10.81 |
| GOTERM_BP_FAT | GO:0030301~cholesterol transport | 0.030682947 | 1 | 10.81 |
| GOTERM_BP_FAT | GO:0048878~chemical homeostasis | 0.031953821 | 1 | 2.27 |
| SP_PIR_KEYWORDS | fatty acid metabolism | 0.032198509 | 0.999876625 | 5.74 |
| GOTERM_BP_FAT | GO:0006875~cellular metal ion homeostasis | 0.032223706 | 1 | 4.14 |
| GOTERM_CC_FAT | GO:0031300~intrinsic to organelle membrane | 0.03225657 | 0.995066473 | 4.13 |
| GOTERM_BP_FAT | GO:0019216~regulation of lipid metabolic process | 0.032343018 | 1 | 5.71 |
| GOTERM_MF_FAT | GO:0008483~transaminase activity | 0.033439053 | 0.999998885 | 10.32 |
| KEGG_PATHWAY | mmu00360:Phenylalanine metabolism | 0.033706041 | 0.941914335 | 10.16 |
| GOTERM_BP_FAT | GO:0060192~negative regulation of lipase activity | 0.035560245 | 1 | 55.24 |
| SP_PIR_KEYWORDS | FAD | 0.035866979 | 0.999956583 | 4.01 |
| GOTERM_BP_FAT | GO:0030003~cellular cation homeostasis | 0.036237895 | 1 | 3.27 |
| GOTERM_BP_FAT | GO:0006260~DNA replication | 0.036237895 | 1 | 3.27 |
| UP_SEQ_FEATURE | compositionally biased region:Pro-rich (proline/acidic region (PAR)) | 0.036439587 | 1 | 53.94 |
| GOTERM_BP_FAT | GO:0055065~metal ion homeostasis | 0.038664532 | 1 | 3.91 |
| UP_SEQ_FEATURE | nucleotide phosphate-binding region:NADP | 0.039170998 | 1 | 5.31 |
| GOTERM_CC_FAT | GO:0005739~mitochondrion | 0.042328693 | 0.999094123 | 1.54 |
| SP_PIR_KEYWORDS | histidine metabolism | 0.044713898 | 0.999996559 | 43.76 |
| KEGG_PATHWAY | mmu00030:Pentose phosphate pathway | 0.0458449 | 0.979658874 | 8.60 |
| GOTERM_BP_FAT | GO:0006644~phospholipid metabolic process | 0.046581057 | 1 | 3.05 |
| GOTERM_BP_FAT | GO:0050873~brown fat cell differentiation | 0.047000551 | 1 | 8.57 |
| GOTERM_MF_FAT | GO:0033765~steroid dehydrogenase activity, acting on the CH-CH group of donors | 0.047306057 | 0.999999997 | 41.27 |
| GOTERM_MF_FAT | GO:0004366~glycerol-3-phosphate O-acyltransferase activity | 0.047306057 | 0.999999997 | 41.27 |
| GOTERM_MF_FAT | GO:0016229~steroid dehydrogenase activity | 0.047313803 | 0.999999997 | 8.54 |
| GOTERM_BP_FAT | GO:0042592~homeostatic process | 0.048609947 | 1 | 1.84 |
| GOTERM_BP_FAT | GO:0009712~catechol metabolic process | 0.049970244 | 1 | 8.29 |
| GOTERM_BP_FAT | GO:0018958~phenol metabolic process | 0.049970244 | 1 | 8.29 |
| GOTERM_BP_FAT | GO:0006584~catecholamine metabolic process | 0.049970244 | 1 | 8.29 |
| GOTERM_BP_FAT | GO:0034311~diol metabolic process | 0.049970244 | 1 | 8.29 |
| GOTERM_MF_FAT | GO:0008238~exopeptidase activity | 0.052239684 | 1 | 4.72 |
| GOTERM_BP_FAT | GO:0006730~one-carbon metabolic process | 0.052251809 | 1 | 3.54 |
| GOTERM_BP_FAT | GO:0006869~lipid transport | 0.054968779 | 1 | 3.48 |
| GOTERM_CC_FAT | GO:0044429~mitochondrial part | 0.054987608 | 0.999895076 | 1.94 |
| GOTERM_BP_FAT | GO:0060191~regulation of lipase activity | 0.05610541 | 1 | 7.77 |
| GOTERM_MF_FAT | GO:0016769~transferase activity, transferring nitrogenous groups | 0.056474812 | 1 | 7.74 |
| UP_SEQ_FEATURE | glycosylation site:N-linked (GlcNAc...) | 0.057640086 | 1 | 1.25 |
| GOTERM_BP_FAT | GO:0006974~response to DNA damage stimulus | 0.057824228 | 1 | 2.31 |
| GOTERM_CC_FAT | GO:0005887~integral to plasma membrane | 0.059086326 | 0.999948112 | 1.92 |
| GOTERM_BP_FAT | GO:0006351~transcription, DNA-dependent | 0.059185196 | 1 | 3.40 |
| KEGG_PATHWAY | mmu00860:Porphyrin and chlorophyll metabolism | 0.059329371 | 0.993758062 | 7.45 |
| PIR_SUPERFAMILY | PIRSF018524:Noelin/Myocilin | 0.06231518 | 0.999584201 | 30.99 |
| INTERPRO | IPR008067:Cytochrome P450, E-class, group I, CYP2A-like | 0.064094563 | 1 | 30.21 |
| GOTERM_MF_FAT | GO:0008374~O-acyltransferase activity | 0.066196105 | 1 | 7.07 |
| SP_PIR_KEYWORDS | transcription termination | 0.066319024 | 0.999999994 | 29.17 |
| GOTERM_BP_FAT | GO:0010876~lipid localization | 0.068120286 | 1 | 3.24 |
| GOTERM_BP_FAT | GO:0006641~triglyceride metabolic process | 0.069104526 | 1 | 6.90 |
| GOTERM_BP_FAT | GO:0042981~regulation of apoptosis | 0.069199701 | 1 | 1.80 |
| GOTERM_BP_FAT | GO:0035238~vitamin A biosynthetic process | 0.069863441 | 1 | 27.62 |
| GOTERM_BP_FAT | GO:0042905~9-cis-retinoic acid metabolic process | 0.069863441 | 1 | 27.62 |
| GOTERM_BP_FAT | GO:0042904~9-cis-retinoic acid biosynthetic process | 0.069863441 | 1 | 27.62 |
| GOTERM_BP_FAT | GO:0006353~transcription termination | 0.069863441 | 1 | 27.62 |
| KEGG_PATHWAY | mmu00150:Androgen and estrogen metabolism | 0.070223802 | 0.997626391 | 6.77 |
| GOTERM_CC_FAT | GO:0031301~integral to organelle membrane | 0.070347686 | 0.999992622 | 4.16 |
| GOTERM_BP_FAT | GO:0055080~cation homeostasis | 0.070780164 | 1 | 2.70 |
| SMART | SM00399:ZnF_C4 | 0.072173339 | 0.999057206 | 6.70 |
| GOTERM_BP_FAT | GO:0002526~acute inflammatory response | 0.073497355 | 1 | 4.09 |
| GOTERM_BP_FAT | GO:0043067~regulation of programmed cell death | 0.074177 | 1 | 1.78 |
| INTERPRO | IPR008069:Cytochrome P450, E-class, group I, CYP2D-like | 0.074372288 | 1 | 25.89 |
| GOTERM_BP_FAT | GO:0010941~regulation of cell death | 0.076378543 | 1 | 1.77 |
| PIR_SUPERFAMILY | PIRSF000045:cytochrome P450 CYP2D6 | 0.077002268 | 0.999938443 | 6.46 |
| SMART | SM00430:HOLI | 0.077586933 | 0.9994529 | 6.43 |
| GOTERM_BP_FAT | GO:0030005~cellular di-, tri-valent inorganic cation homeostasis | 0.077712958 | 1 | 3.09 |
| GOTERM_BP_FAT | GO:0016042~lipid catabolic process | 0.077712958 | 1 | 3.09 |
| GOTERM_BP_FAT | GO:0008654~phospholipid biosynthetic process | 0.077821048 | 1 | 3.99 |
| GOTERM_MF_FAT | GO:0046914~transition metal ion binding | 0.080187705 | 1 | 1.27 |
| BIOCARTA | m_nuclearRsPathway:Nuclear Receptors in Lipid Metabolism and Toxicity | 0.080470289 | 0.958742861 | 5.91 |
| GOTERM_CC_FAT | GO:0005789~endoplasmic reticulum membrane | 0.080612165 | 0.999998779 | 3.05 |
| GOTERM_BP_FAT | GO:0042362~fat-soluble vitamin biosynthetic process | 0.081026176 | 1 | 23.67 |
| SP_PIR_KEYWORDS | transport | 0.084079477 | 1 | 1.39 |
| INTERPRO | IPR016040:NAD(P)-binding domain | 0.084698757 | 1 | 3.00 |
| GOTERM_BP_FAT | GO:0030001~metal ion transport | 0.084915762 | 1 | 1.87 |
| SP_PIR_KEYWORDS | metalloprotease | 0.085385305 | 1 | 3.00 |
| KEGG_PATHWAY | mmu00051:Fructose and mannose metabolism | 0.085665395 | 0.999408809 | 6.04 |
| GOTERM_BP_FAT | GO:0006811~ion transport | 0.086431154 | 1 | 1.63 |
| SP_PIR_KEYWORDS | hydrolase | 0.08695903 | 1 | 1.40 |
| SP_PIR_KEYWORDS | lipid binding | 0.087437873 | 1 | 21.88 |
| SP_PIR_KEYWORDS | dimer | 0.087437873 | 1 | 21.88 |
| GOTERM_BP_FAT | GO:0006812~cation transport | 0.091345187 | 1 | 1.77 |
| GOTERM_BP_FAT | GO:0017144~drug metabolic process | 0.092055756 | 1 | 20.71 |
| GOTERM_MF_FAT | GO:0016840~carbon-nitrogen lyase activity | 0.092387578 | 1 | 20.63 |
| GOTERM_CC_FAT | GO:0042175~nuclear envelope-endoplasmic reticulum network | 0.093080674 | 0.999999866 | 2.89 |
| KEGG_PATHWAY | mmu00350:Tyrosine metabolism | 0.093737459 | 0.999716806 | 5.73 |
| GOTERM_BP_FAT | GO:0006479~protein amino acid methylation | 0.093888375 | 1 | 5.78 |
| GOTERM_BP_FAT | GO:0006639~acylglycerol metabolic process | 0.093888375 | 1 | 5.78 |
| GOTERM_BP_FAT | GO:0008213~protein amino acid alkylation | 0.093888375 | 1 | 5.78 |
| INTERPRO | IPR001628:Zinc finger, nuclear hormone receptor-type | 0.094049377 | 1 | 5.78 |
| GOTERM_MF_FAT | GO:0015300~solute:solute antiporter activity | 0.094479112 | 1 | 5.76 |
| INTERPRO | IPR012269:Aquaporin | 0.094592079 | 1 | 20.14 |
| INTERPRO | IPR002355:Multicopper oxidase, copper-binding site | 0.094592079 | 1 | 20.14 |
| GOTERM_BP_FAT | GO:0006874~cellular calcium ion homeostasis | 0.096182436 | 1 | 3.64 |
| SP_PIR_KEYWORDS | extracellular matrix | 0.096512402 | 1 | 2.47 |
| KEGG_PATHWAY | mmu00590:Arachidonic acid metabolism | 0.096890634 | 0.999787945 | 3.59 |
| INTERPRO | IPR001723:Steroid hormone receptor | 0.097456386 | 1 | 5.66 |
| UP_SEQ_FEATURE | active site:Charge relay system | 0.097829014 | 1 | 2.45 |
| GOTERM_BP_FAT | GO:0055066~di-, tri-valent inorganic cation homeostasis | 0.098671497 | 1 | 2.84 |
| GOTERM_CC_FAT | GO:0005578~proteinaceous extracellular matrix | 0.09987083 | 0.99999996 | 2.18 |

**Suppl. Table S16** Functional annotation terms associated with genes exhibiting UPregulation in diabetes relative to control, and ameliorated by losartan treatment (n = 9 annotation terms; ranked by ascending p-value)

| **Category** | **Term** | **PValue** | **Bonferroni** | **Fold Enrichment** |
| --- | --- | --- | --- | --- |
| GOTERM_MF_FAT | GO:0019904~protein domain specific binding | 0.004806661 | 0.391208692 | 11.07 |
| GOTERM_MF_FAT | GO:0015293~symporter activity | 0.023434051 | 0.913051428 | 12.08 |
| GOTERM_MF_FAT | GO:0030165~PDZ domain binding | 0.044227389 | 0.990526252 | 42.52 |
| GOTERM_MF_FAT | GO:0008235~metalloexopeptidase activity | 0.057979793 | 0.997870868 | 32.21 |
| GOTERM_MF_FAT | GO:0004857~enzyme inhibitor activity | 0.070638422 | 0.999471524 | 6.56 |
| GOTERM_MF_FAT | GO:0015370~solute:sodium symporter activity | 0.079923866 | 0.999812134 | 23.11 |
| GOTERM_MF_FAT | GO:0015171~amino acid transmembrane transporter activity | 0.086576745 | 0.999911034 | 21.26 |
| SP_PIR_KEYWORDS | sh3-binding | 0.091543424 | 0.999764244 | 20.21 |
| GOTERM_BP_FAT | GO:0044271~nitrogen compound biosynthetic process | 0.091765247 | 1 | 5.62 |

**Suppl. Table S17** Functional annotation terms associated with genes exhibiting DOWNregulation in diabetes relative to control, and ameliorated by losartan treatment (n = 70 annotation terms; ranked by ascending p-value)

| **Category** | **Term** | **PValue** | **Bonferroni** | **Fold Enrichment** |
| --- | --- | --- | --- | --- |
| SP_PIR_KEYWORDS | stress response | 5.36E-09 | 7.12E-07 | 48.72 |
| GOTERM_CC_FAT | GO:0005788~endoplasmic reticulum lumen | 8.28E-08 | 9.11E-06 | 30.39 |
| SP_PIR_KEYWORDS | acetylation | 3.26E-06 | 4.33E-04 | 2.96 |
| INTERPRO | IPR001023:Heat shock protein Hsp70 | 4.55E-06 | 7.10E-04 | 115.34 |
| INTERPRO | IPR013126:Heat shock protein 70 | 4.55E-06 | 7.10E-04 | 115.34 |
| INTERPRO | IPR018181:Heat shock protein 70, conserved site | 7.86E-06 | 0.001225293 | 97.60 |
| UP_SEQ_FEATURE | short sequence motif:Prevents secretion from ER | 5.28E-05 | 0.012751715 | 23.82 |
| GOTERM_CC_FAT | GO:0044432~endoplasmic reticulum part | 7.30E-05 | 0.007994487 | 9.47 |
| GOTERM_MF_FAT | GO:0051082~unfolded protein binding | 8.18E-05 | 0.010331368 | 21.09 |
| SP_PIR_KEYWORDS | Chaperone | 1.06E-04 | 0.013988042 | 12.53 |
| SP_PIR_KEYWORDS | phosphoprotein | 2.64E-04 | 0.034558578 | 1.69 |
| INTERPRO | IPR000886:Endoplasmic reticulum, targeting sequence | 3.18E-04 | 0.04833638 | 29.51 |
| PIR_SUPERFAMILY | PIRSF002581:chaperone HSP70 | 4.14E-04 | 0.01722115 | 92.45 |
| GOTERM_CC_FAT | GO:0070013~intracellular organelle lumen | 4.99E-04 | 0.053473989 | 3.31 |
| GOTERM_CC_FAT | GO:0043233~organelle lumen | 5.11E-04 | 0.054664553 | 3.30 |
| GOTERM_CC_FAT | GO:0031974~membrane-enclosed lumen | 6.76E-04 | 0.071693438 | 3.20 |
| SP_PIR_KEYWORDS | molecular chaperone | 8.58E-04 | 0.107922704 | 67.12 |
| GOTERM_BP_FAT | GO:0006457~protein folding | 0.00111394 | 0.421460977 | 10.70 |
| SP_PIR_KEYWORDS | endoplasmic reticulum | 0.001176684 | 0.144946364 | 4.16 |
| KEGG_PATHWAY | mmu04612:Antigen processing and presentation | 0.001529405 | 0.04341603 | 15.76 |
| GOTERM_CC_FAT | GO:0042470~melanosome | 0.002322164 | 0.225652902 | 14.71 |
| GOTERM_CC_FAT | GO:0048770~pigment granule | 0.002322164 | 0.225652902 | 14.71 |
| GOTERM_CC_FAT | GO:0005783~endoplasmic reticulum | 0.003765367 | 0.339641315 | 3.36 |
| GOTERM_CC_FAT | GO:0005829~cytosol | 0.006614908 | 0.518118585 | 3.99 |
| SP_PIR_KEYWORDS | stress-induced protein | 0.012488348 | 0.812017934 | 156.61 |
| SP_PIR_KEYWORDS | heat shock | 0.012488348 | 0.812017934 | 156.61 |
| GOTERM_BP_FAT | GO:0030003~cellular cation homeostasis | 0.017370693 | 0.999816621 | 7.15 |
| SP_PIR_KEYWORDS | atp-binding | 0.017931767 | 0.909875847 | 2.43 |
| GOTERM_MF_FAT | GO:0000166~nucleotide binding | 0.02124421 | 0.934590364 | 1.89 |
| GOTERM_BP_FAT | GO:0030036~actin cytoskeleton organization | 0.021544477 | 0.999977318 | 6.59 |
| GOTERM_BP_FAT | GO:0030029~actin filament-based process | 0.025469602 | 0.999996848 | 6.18 |
| GOTERM_MF_FAT | GO:0019904~protein domain specific binding | 0.025494317 | 0.962362935 | 6.15 |
| GOTERM_BP_FAT | GO:0055080~cation homeostasis | 0.028550947 | 0.999999334 | 5.91 |
| SP_PIR_KEYWORDS | nucleotide-binding | 0.028887721 | 0.979730818 | 2.11 |
| GOTERM_MF_FAT | GO:0032555~purine ribonucleotide binding | 0.029117187 | 0.976547711 | 1.97 |
| GOTERM_MF_FAT | GO:0032553~ribonucleotide binding | 0.029117187 | 0.976547711 | 1.97 |
| GOTERM_BP_FAT | GO:0007010~cytoskeleton organization | 0.029576937 | 0.999999604 | 4.17 |
| UP_SEQ_FEATURE | cross-link:Glycyl lysine isopeptide (Lys-Gly) (interchain with G-Cter in SUMO) | 0.037931284 | 0.999917 | 9.58 |
| GOTERM_MF_FAT | GO:0017076~purine nucleotide binding | 0.037963905 | 0.99266663 | 1.89 |
| GOTERM_CC_FAT | GO:0016023~cytoplasmic membrane-bounded vesicle | 0.03913362 | 0.987614038 | 3.78 |
| KEGG_PATHWAY | mmu03040:Spliceosome | 0.040482429 | 0.698327976 | 8.68 |
| GOTERM_CC_FAT | GO:0031988~membrane-bounded vesicle | 0.04091615 | 0.989902274 | 3.72 |
| GOTERM_BP_FAT | GO:0051085~chaperone mediated protein folding requiring cofactor | 0.042442281 | 0.999999999 | 45.29 |
| GOTERM_MF_FAT | GO:0005524~ATP binding | 0.044010572 | 0.996707374 | 2.05 |
| GOTERM_MF_FAT | GO:0032559~adenyl ribonucleotide binding | 0.046889968 | 0.997755259 | 2.02 |
| GOTERM_BP_FAT | GO:0006458~'de novo' protein folding | 0.049342307 | 1 | 38.82 |
| GOTERM_BP_FAT | GO:0051084~'de novo' posttranslational protein folding | 0.049342307 | 1 | 38.82 |
| GOTERM_BP_FAT | GO:0006875~cellular metal ion homeostasis | 0.050424149 | 1 | 8.15 |
| GOTERM_BP_FAT | GO:0045664~regulation of neuron differentiation | 0.052235718 | 1 | 7.99 |
| GOTERM_CC_FAT | GO:0045120~pronucleus | 0.05471432 | 0.997949127 | 34.73 |
| GOTERM_BP_FAT | GO:0055065~metal ion homeostasis | 0.055928032 | 1 | 7.69 |
| GOTERM_BP_FAT | GO:0033554~cellular response to stress | 0.057206376 | 1 | 3.36 |
| GOTERM_MF_FAT | GO:0030554~adenyl nucleotide binding | 0.061129832 | 0.999668179 | 1.92 |
| GOTERM_MF_FAT | GO:0001883~purine nucleoside binding | 0.063859867 | 0.99977076 | 1.91 |
| GOTERM_MF_FAT | GO:0001882~nucleoside binding | 0.066013654 | 0.999828901 | 1.90 |
| GOTERM_BP_FAT | GO:0045428~regulation of nitric oxide biosynthetic process | 0.066379987 | 1 | 28.61 |
| GOTERM_BP_FAT | GO:0006873~cellular ion homeostasis | 0.067689622 | 1 | 4.16 |
| GOTERM_BP_FAT | GO:0055082~cellular chemical homeostasis | 0.072055578 | 1 | 4.06 |
| GOTERM_CC_FAT | GO:0031410~cytoplasmic vesicle | 0.072355732 | 0.999741797 | 3.08 |
| GOTERM_BP_FAT | GO:0030199~collagen fibril organization | 0.073110986 | 1 | 25.88 |
| GOTERM_CC_FAT | GO:0031982~vesicle | 0.076972606 | 0.999850856 | 3.01 |
| SP_PIR_KEYWORDS | viral nucleoprotein | 0.081379246 | 0.999987494 | 23.20 |
| SP_PIR_KEYWORDS | ubl conjugation | 0.081711046 | 0.999988081 | 2.99 |
| GOTERM_BP_FAT | GO:0050767~regulation of neurogenesis | 0.081969823 | 1 | 6.18 |
| GOTERM_BP_FAT | GO:0001818~negative regulation of cytokine production | 0.083118441 | 1 | 22.65 |
| GOTERM_BP_FAT | GO:0030005~cellular di-, tri-valent inorganic cation homeostasis | 0.084105488 | 1 | 6.08 |
| GOTERM_BP_FAT | GO:0050801~ion homeostasis | 0.088649519 | 1 | 3.71 |
| GOTERM_BP_FAT | GO:0031669~cellular response to nutrient levels | 0.096297223 | 1 | 19.41 |
| GOTERM_BP_FAT | GO:0055066~di-, tri-valent inorganic cation homeostasis | 0.097266988 | 1 | 5.58 |
| GOTERM_BP_FAT | GO:0051960~regulation of nervous system development | 0.09951536 | 1 | 5.51 |

**Suppl. Table S18** Genes unaffected by diabetes (i.e., relative to control) but UPregulated with losartan treatment (relative to untreated diabetes, n = 104; ranked alphabetically)

| **Gene_symbol** | **Gene_name** |
| --- | --- |
| 1600029D21Rik | RIKEN cDNA 1600029D21 gene |
| 2810055F11Rik | RIKEN cDNA 2810055F11 gene |
| 4930402H24Rik | RIKEN cDNA 4930402H24 gene |
| 4933407C03Rik | RIKEN cDNA 4933407C03 gene |
| Actb | actin, beta |
| Actn4 | actinin alpha 4 |
| Add3 | adducin 3 (gamma) |
| Alas1 | aminolevulinic acid synthase 1 |
| Aldoart2 | aldolase 1, A isoform, retrogene 2 |
| Alpl | alkaline phosphatase, liver/bone/kidney |
| Amd1 | S-adenosylmethionine decarboxylase 1 |
| Aqp2 | aquaporin 2 |
| Armc6 | armadillo repeat containing 6 |
| Aup1 | ancient ubiquitous protein 1 |
| Avpr2 | arginine vasopressin receptor 2 |
| AW555464 | expressed sequence AW555464 |
| Btc | betacellulin, epidermal growth factor family member |
| C3 | complement component 3; similar to complement component C3 prepropeptide, last |
| C4b | Complement component 4B (Chido blood group) |
| Ccdc141 | RIKEN cDNA 2610301F02 gene |
| Ccnd1 | cyclin D1 |
| Cd63 | CD63 antigen |
| Cdkn1a | cyclin-dependent kinase inhibitor 1A (P21) |
| Cnnm4 | cyclin M4 |
| Cpn1 | carboxypeptidase N, polypeptide 1 |
| Creg1 | cellular repressor of E1A-stimulated genes 1 |
| Dap | death-associated protein |
| Dhrs3 | dehydrogenase/reductase (SDR family) member 3 |
| Dynll2 | dynein light chain LC8-type 2 |
| Eef1e1 | eukaryotic translation elongation factor 1 epsilon 1 |
| Eif1 | Eukaryotic translation initiation factor 1 |
| Epb4.1l3 | erythrocyte protein band 4.1-like 3 |
| Fam107b | family with sequence similarity 107, member B |
| Fam129a | family with sequence similarity 129, member A |
| Fam176a | family with sequence similarity 176, member A |
| Fam20c | family with sequence similarity 20, member C |
| Fgb | fibrinogen beta chain |
| Fmo1 | flavin containing monooxygenase 1 |
| Ftl1 | Ferritin light chain 1 |
| Gldc | glycine decarboxylase |
| Gm8615 | predicted gene 8615 |
| Gna14 | guanine nucleotide binding protein, alpha 14 |
| Gnpda1 | Glucosamine-6-phosphate deaminase 1 |
| Gpatch4 | G patch domain containing 4 |
| Grem2 | gremlin 2 homolog, cysteine knot superfamily (Xenopus laevis) |
| Gstp1 | glutathione S-transferase, pi 2; glutathione S-transferase, pi 1 |
| H2-Ab1 | histocompatibility 2, class II antigen A, beta 1 |
| H2-D1 | histocompatibility 2, D region; histocompatibility 2, D region locus 1 |
| Hbxip | hepatitis B virus x interacting protein |
| Hexa | hexosaminidase A |
| Hmgn1 | High mobility group nucleosomal binding domain 1 |
| Id2 | inhibitor of DNA binding 2 |
| Insc | inscuteable homolog (Drosophila) |
| Irak2 | interleukin-1 receptor-associated kinase 2 |
| Jub | ajuba |
| Krt19 | keratin 19 |
| Lad1 | ladinin |
| Ltbr | lymphotoxin B receptor |
| Lyz2 | lysozyme 2 |
| Mcoln3 | mucolipin 3 |
| Mdm2 | transformed mouse 3T3 cell double minute 2 |
| Med29 | mediator complex subunit 29; predicted gene 9450 |
| Mkks | McKusick-Kaufman syndrome protein |
| Myc | myelocytomatosis oncogene |
| Ncbp2 | nuclear cap binding protein subunit 2 |
| Ncoa7 | nuclear receptor coactivator 7 |
| Ndrg1 | N-myc downstream regulated gene 1 |
| Nop2 | NOP2 nucleolar protein homolog (yeast) |
| Obfc2a | oligonucleotide/oligosaccharide-binding fold containing 2A |
| Pdrg1 | p53 and DNA damage regulated 1 |
| Phf5a | PHD finger protein 5A |
| Plk2 | polo-like kinase 2 (Drosophila) |
| Ppcs | predicted gene 8465; phosphopantothenoylcysteine synthetase |
| Rcan1 | regulator of calcineurin 1 |
| Ren1 | renin 1 structural |
| Rorc | RAR-related orphan receptor gamma |
| Rps8 | Ribosomal protein S8 |
| Scd2 | stearoyl-Coenzyme A desaturase 2 |
| Sh3tc1 | SH3 domain and tetratricopeptide repeats 1 |
| Sh3tc2 | SH3 domain and tetratricopeptide repeats 2 |
| Slc16a1 | solute carrier family 16 (monocarboxylic acid transporters), member 1 |
| Slc16a6 | solute carrier family 16 (monocarboxylic acid transporters), member 6 |
| Slc22a23 | solute carrier family 22, member 23 |
| Slc25a25 | solute carrier family 25 (mitochondrial carrier, phosphate carrier), member 25 |
| Slc25a45 | solute carrier family 25, member 45 |
| Slc3a2 | solute carrier family 3 (activators of dibasic and neutral amino acid transport), member 2 |
| Slc7a8 | solute carrier family 7 (cationic amino acid transporter, y+ system), member 8 |
| Smcp | sperm mitochondria-associated cysteine-rich protein |
| Spns2 | spinster homolog 2 (Drosophila) |
| Tacstd2 | tumor-associated calcium signal transducer 2 |
| Tbc1d1 | TBC1 domain family, member 1; similar to TBC1 domain family member 1 |
| Tchhl1 | trichohyalin-like 1 |
| Thbd | thrombomodulin |
| Timp3 | tissue inhibitor of metalloproteinase 3 |
| Tmco4 | transmembrane and coiled-coil domains 4 |
| Tmem37 | transmembrane protein 37 |
| Tmem52 | transmembrane protein 52 |
| Tmem82 | transmembrane protein 82 |
| Tomm5 | translocase of outer mitochondrial membrane 5 homolog (yeast) |
| Tubb2a | tubulin, beta 2A |
| Vcl | vinculin |
| Wfdc2 | WAP four-disulfide core domain 2 |
| Wsb1 | WD repeat and SOCS box-containing 1 |

**Suppl. Table S19** Genes unaffected by diabetes (i.e., relative to control) but DOWNregulated with losartan treatment (relative to untreated diabetes, n = 85; ranked alphabetically)

| **Gene symbol** | **Gene name** |
| --- | --- |
| 2210404J11Rik | RIKEN cDNA 9030025P20 gene; predicted gene 3435; RIKEN cDNA 2210404J11 gene |
| 4933407H18Rik | RIKEN cDNA 4933407H18 gene |
| A930001N09Rik | RIKEN cDNA A930001N09 gene |
| Ace2 | angiotensin I converting enzyme (peptidyl-dipeptidase A) 2 |
| Adck3 | aarF domain containing kinase 3 |
| Afap1l1 | actin filament associated protein 1-like 1 |
| Afm | afamin |
| Apom | apolipoprotein M |
| Asb9 | ankyrin repeat and SOCS box-containing 9 |
| BC024386 | cDNA sequence BC023486 |
| Bcat1 | branched chain aminotransferase 1, cytosolic; similar to branched chain aminotransferase 1, cytosolic |
| Btg1 | B-cell translocation gene 1, anti-proliferative; similar to myocardial vascular inhibition factor |
| C8g | complement component 8, gamma polypeptide |
| Calb1 | calbindin 1 |
| Car3 | carbonic anhydrase 3 |
| Ccbl1 | cysteine conjugate-beta lyase 1 |
| Ccdc148 | coiled-coil domain containing 148 |
| Ccdc28a | coiled-coil domain containing 28A |
| Cdkn2c | cyclin-dependent kinase inhibitor 2C (p18, inhibits CDK4) |
| Clip4 | CAP-GLY domain containing linker protein family, member 4; similar to CAP-GLY domain containing linker protein family, member 4 |
| Cndp1 | carnosine dipeptidase 1 (metallopeptidase M20 family) |
| Ctbs | chitobiase, di-N-acetyl- |
| Cyp2j9 | cytochrome P450, family 2, subfamily j, polypeptide 9 |
| D4Wsu53e | DNA segment, Chr 4, Wayne State University 53, expressed |
| D630029K05Rik | RIKEN cDNA D630029K05 gene |
| Dbp | D site albumin promoter binding protein |
| Dnajc12 | DnaJ (Hsp40) homolog, subfamily C, member 12 |
| Dusp1 | dual specificity phosphatase 1 |
| Erbb3 | v-erb-b2 erythroblastic leukemia viral oncogene homolog 3 (avian) |
| Fam81a | family with sequence similarity 81, member A |
| Fnip2 | folliculin interacting protein 2 |
| Gatsl3 | GATS protein-like 3 |
| Gcom1 | GRINL1A complex locus |
| Gem | GTP binding protein (gene overexpressed in skeletal muscle) |
| Gm11992 | predicted gene 11992 |
| Gpam | glycerol-3-phosphate acyltransferase, mitochondrial |
| Gpm6a | glycoprotein m6a |
| Hbp1 | high mobility group box transcription factor 1 |
| Itih5 | inter-alpha (globulin) inhibitor H5 |
| Kcnj2 | potassium inwardly-rectifying channel, subfamily J, member 2 |
| Klf12 | Kruppel-like factor 12 |
| Klhl24 | kelch-like 24 (Drosophila) |
| Lbr | lamin B receptor |
| Lrrc29 | leucine rich repeat containing 29 |
| Mcm10 | minichromosome maintenance deficient 10 (S. cerevisiae) |
| Mfsd9 | major facilitator superfamily domain containing 9 |
| Nr1d1 | nuclear receptor subfamily 1, group D, member 1 |
| Nsdhl | NAD(P) dependent steroid dehydrogenase-like |
| Olfm4 | olfactomedin 4 |
| Olfml3 | olfactomedin-like 3 |
| Osbpl9 | oxysterol binding protein-like 9 |
| Pank3 | pantothenate kinase 3 |
| Phtf2 | putative homeodomain transcription factor 2 |
| Ppara | peroxisome proliferator activated receptor alpha |
| Ppm1k | protein phosphatase 1K (PP2C domain containing) |
| Prss35 | protease, serine, 35 |
| Rab43 | RAB43, member RAS oncogene family |
| Rasa3 | RAS p21 protein activator 3 |
| Rdh16 | retinol dehydrogenase 16 |
| Rhebl1 | Ras homolog enriched in brain like 1 |
| Rpain | RPA interacting protein |
| Scd1 | stearoyl-Coenzyme A desaturase 1 |
| Sgms2 | sphingomyelin synthase 2 |
| Slc22a22 | solute carrier family 22, member 22 |
| Slc41a2 | solute carrier family 41, member 2 |
| Slc8a1 | solute carrier family 8 (sodium/calcium exchanger), member 1 |
| Slco4c1 | solute carrier organic anion transporter family, member 4C1 |
| Srd5a1 | steroid 5 alpha-reductase 1 |
| Sucnr1 | succinate receptor 1 |
| Suv39h1 | suppressor of variegation 3-9 homolog 1 (Drosophila) |
| Tcea3 | transcription elongation factor A (SII), 3 |
| Tcp11l2 | t-complex 11 (mouse) like 2 |
| Tef | thyrotroph embryonic factor |
| Tgds | TDP-glucose 4,6-dehydratase |
| Tmem174 | transmembrane protein 174 |
| Tmem220 | transmembrane protein 220 |
| Tmem26 | transmembrane protein 26 |
| Tmem53 | transmembrane protein 53 |
| Trim7 | tripartite motif-containing 7 |
| Ttr | transthyretin |
| Vdr | vitamin D receptor |
| Xrn2 | 5'-3' exoribonuclease 2 |
| Ypel5 | yippee-like 5 (Drosophila) |
| Zfand1 | zinc finger, AN1-type domain 1 |

**Suppl. Table S20** Functional annotation terms for genes unaffected by diabetes (i.e., relative to control) but UPregulated with losartan treatment (relative to untreated diabetes, n = 89 terms; ranked by ascending p-value)

| **Category** | **Term** | **PValue** | **Bonferroni** | **Fold Enrichment** |
| --- | --- | --- | --- | --- |
| BIOCARTA | m_p53Pathway:p53 Signaling Pathway | 4.28E-04 | 0.015292475 | 22.52 |
| GOTERM_BP_FAT | GO:0009628~response to abiotic stimulus | 4.60E-04 | 0.315494921 | 5.70 |
| KEGG_PATHWAY | mmu05219:Bladder cancer | 0.002201403 | 0.141066877 | 14.77 |
| INTERPRO | IPR001134:Netrin domain | 0.004644672 | 0.704692756 | 28.96 |
| GOTERM_BP_FAT | GO:0048535~lymph node development | 0.004837265 | 0.981603188 | 28.23 |
| UP_SEQ_FEATURE | domain:NTR | 0.006420896 | 0.890945762 | 24.52 |
| GOTERM_BP_FAT | GO:0051726~regulation of cell cycle | 0.006537515 | 0.995504139 | 5.01 |
| SP_PIR_KEYWORDS | plasma | 0.007459224 | 0.798558696 | 22.77 |
| GOTERM_BP_FAT | GO:0009314~response to radiation | 0.008025792 | 0.998692887 | 6.25 |
| INTERPRO | IPR011701:Major facilitator superfamily MFS-1 | 0.010755516 | 0.941176214 | 8.68 |
| GOTERM_BP_FAT | GO:0007605~sensory perception of sound | 0.011178022 | 0.999905088 | 8.51 |
| KEGG_PATHWAY | mmu04610:Complement and coagulation cascades | 0.011240666 | 0.541593105 | 8.27 |
| KEGG_PATHWAY | mmu05220:Chronic myeloid leukemia | 0.01165271 | 0.554589202 | 8.16 |
| GOTERM_BP_FAT | GO:0055085~transmembrane transport | 0.013337095 | 0.999984329 | 3.11 |
| GOTERM_BP_FAT | GO:0016485~protein processing | 0.013462369 | 0.999985886 | 7.95 |
| GOTERM_BP_FAT | GO:0050954~sensory perception of mechanical stimulus | 0.014278595 | 0.999992864 | 7.77 |
| GOTERM_BP_FAT | GO:0045807~positive regulation of endocytosis | 0.015045748 | 0.999996243 | 15.78 |
| GOTERM_BP_FAT | GO:0007346~regulation of mitotic cell cycle | 0.015993967 | 0.999998301 | 7.45 |
| GOTERM_BP_FAT | GO:0051604~protein maturation | 0.015993967 | 0.999998301 | 7.45 |
| UP_SEQ_FEATURE | domain:Anaphylatoxin-like | 0.018055029 | 0.998103383 | 108.99 |
| KEGG_PATHWAY | mmu05215:Prostate cancer | 0.018343829 | 0.721260395 | 6.89 |
| GOTERM_BP_FAT | GO:0009612~response to mechanical stimulus | 0.019524649 | 0.999999912 | 13.75 |
| INTERPRO | IPR018081:Anaphylatoxin | 0.020336804 | 0.995406754 | 96.54 |
| INTERPRO | IPR001840:Complement C3a/C4a/C5a anaphylatoxin | 0.020336804 | 0.995406754 | 96.54 |
| KEGG_PATHWAY | mmu05416:Viral myocarditis | 0.020576576 | 0.761788043 | 6.60 |
| GOTERM_CC_FAT | GO:0005615~extracellular space | 0.020625809 | 0.959626825 | 2.84 |
| GOTERM_BP_FAT | GO:0010033~response to organic substance | 0.021171763 | 0.999999978 | 2.83 |
| GOTERM_BP_FAT | GO:0051270~regulation of cell motion | 0.021286321 | 0.99999998 | 6.68 |
| GOTERM_CC_FAT | GO:0044421~extracellular region part | 0.02365835 | 0.974957382 | 2.34 |
| SP_PIR_KEYWORDS | acetylation | 0.023765116 | 0.994183993 | 1.65 |
| GOTERM_BP_FAT | GO:0033554~cellular response to stress | 0.023958041 | 0.999999998 | 3.10 |
| GOTERM_BP_FAT | GO:0042060~wound healing | 0.023972426 | 0.999999998 | 6.39 |
| KEGG_PATHWAY | mmu05322:Systemic lupus erythematosus | 0.026127849 | 0.839070988 | 6.02 |
| GOTERM_MF_FAT | GO:0004857~enzyme inhibitor activity | 0.028047331 | 0.990034275 | 4.27 |
| SMART | SM00104:ANATO | 0.029527343 | 0.748097801 | 65.22 |
| GOTERM_BP_FAT | GO:0051130~positive regulation of cellular component organization | 0.029870976 | 1 | 5.86 |
| GOTERM_CC_FAT | GO:0005938~cell cortex | 0.031159166 | 0.992363841 | 5.75 |
| GOTERM_BP_FAT | GO:0030100~regulation of endocytosis | 0.033373384 | 1 | 10.31 |
| INTERPRO | IPR000020:Anaphylatoxin/fibulin | 0.035320466 | 0.999919023 | 55.16 |
| GOTERM_BP_FAT | GO:0051050~positive regulation of transport | 0.036466875 | 1 | 5.42 |
| SP_PIR_KEYWORDS | thioester bond | 0.037422605 | 0.999714747 | 52.05 |
| INTERPRO | IPR019565:Alpha-2-macroglobulin, thiol-ester bond-forming | 0.040264476 | 0.999978928 | 48.27 |
| GOTERM_MF_FAT | GO:0004866~endopeptidase inhibitor activity | 0.040871397 | 0.998841093 | 5.16 |
| UP_SEQ_FEATURE | cross-link:Isoglutamyl cysteine thioester (Cys-Gln) | 0.041627439 | 0.999999556 | 46.71 |
| GOTERM_BP_FAT | GO:0009611~response to wounding | 0.042643758 | 1 | 3.09 |
| INTERPRO | IPR011625:Alpha-2-macroglobulin, N-terminal 2 | 0.045183424 | 0.999994517 | 42.91 |
| INTERPRO | IPR011626:A-macroglobulin complement component | 0.045183424 | 0.999994517 | 42.91 |
| INTERPRO | IPR001599:Alpha-2-macroglobulin | 0.045183424 | 0.999994517 | 42.91 |
| INTERPRO | IPR009048:Alpha-macroglobulin, receptor-binding | 0.045183424 | 0.999994517 | 42.91 |
| KEGG_PATHWAY | mmu04110:Cell cycle | 0.045362186 | 0.959368235 | 4.85 |
| GOTERM_MF_FAT | GO:0003779~actin binding | 0.047546046 | 0.999626125 | 3.60 |
| GOTERM_CC_FAT | GO:0000307~cyclin-dependent protein kinase holoenzyme complex | 0.047908046 | 0.999479345 | 40.27 |
| GOTERM_BP_FAT | GO:0050910~detection of mechanical stimulus involved in sensory perception of sound | 0.048607424 | 1 | 39.73 |
| GOTERM_BP_FAT | GO:0008217~regulation of blood pressure | 0.050017418 | 1 | 8.25 |
| GOTERM_BP_FAT | GO:0051605~protein maturation by peptide bond cleavage | 0.050017418 | 1 | 8.25 |
| INTERPRO | IPR002890:Alpha-2-macroglobulin, N-terminal | 0.050077438 | 0.999998573 | 38.62 |
| GOTERM_MF_FAT | GO:0030414~peptidase inhibitor activity | 0.050903994 | 0.999789019 | 4.72 |
| INTERPRO | IPR019742:Alpha-2-macroglobulin, conserved site | 0.05494664 | 0.999999629 | 35.10 |
| PIR_SUPERFAMILY | PIRSF001635:alpha-2-macroglobulin | 0.055048261 | 0.981003482 | 34.77 |
| GOTERM_BP_FAT | GO:0030031~cell projection assembly | 0.057067602 | 1 | 7.66 |
| SP_PIR_KEYWORDS | thiolester bond | 0.058181112 | 0.999997315 | 33.12 |
| SMART | SM00643:C345C | 0.058204533 | 0.936612532 | 32.61 |
| KEGG_PATHWAY | mmu05214:Glioma | 0.060539043 | 0.986552771 | 7.27 |
| GOTERM_MF_FAT | GO:0005198~structural molecule activity | 0.061756275 | 0.999967256 | 2.77 |
| SP_PIR_KEYWORDS | ubl conjugation | 0.065844162 | 0.999999533 | 2.43 |
| GOTERM_MF_FAT | GO:0016538~cyclin-dependent protein kinase regulator activity | 0.068839469 | 0.999990406 | 27.68 |
| KEGG_PATHWAY | mmu04115:p53 signaling pathway | 0.069101602 | 0.992850799 | 6.74 |
| INTERPRO | IPR018933:Netrin module, non-TIMP type | 0.069406625 | 0.999999993 | 27.58 |
| KEGG_PATHWAY | mmu05218:Melanoma | 0.072633582 | 0.994500234 | 6.55 |
| GOTERM_BP_FAT | GO:0006974~response to DNA damage stimulus | 0.0740417 | 1 | 3.11 |
| GOTERM_BP_FAT | GO:0060627~regulation of vesicle-mediated transport | 0.076858101 | 1 | 6.46 |
| SP_PIR_KEYWORDS | immune response | 0.078778151 | 0.999999976 | 3.96 |
| GOTERM_BP_FAT | GO:0050974~detection of mechanical stimulus involved in sensory perception | 0.0797095 | 1 | 23.84 |
| GOTERM_CC_FAT | GO:0005856~cytoskeleton | 0.0806725 | 0.999997632 | 1.78 |
| KEGG_PATHWAY | mmu04520:Adherens junction | 0.081713858 | 0.997210771 | 6.12 |
| SP_PIR_KEYWORDS | transport | 0.082721903 | 0.999999991 | 1.62 |
| SP_PIR_KEYWORDS | surface antigen | 0.083507166 | 0.999999992 | 22.77 |
| GOTERM_BP_FAT | GO:0044036~cell wall macromolecule metabolic process | 0.084794727 | 1 | 22.35 |
| GOTERM_BP_FAT | GO:0030032~lamellipodium assembly | 0.084794727 | 1 | 22.35 |
| GOTERM_BP_FAT | GO:0016998~cell wall macromolecule catabolic process | 0.084794727 | 1 | 22.35 |
| GOTERM_BP_FAT | GO:0050878~regulation of body fluid levels | 0.08662466 | 1 | 6.03 |
| SP_PIR_KEYWORDS | inflammation | 0.088490898 | 0.999999998 | 21.43 |
| SP_PIR_KEYWORDS | cytoplasm | 0.088974285 | 0.999999998 | 1.38 |
| GOTERM_CC_FAT | GO:0043232~intracellular non-membrane-bounded organelle | 0.089355913 | 0.999999451 | 1.51 |
| GOTERM_CC_FAT | GO:0043228~non-membrane-bounded organelle | 0.089355913 | 0.999999451 | 1.51 |
| GOTERM_BP_FAT | GO:0050982~detection of mechanical stimulus | 0.089852228 | 1 | 21.03 |
| GOTERM_CC_FAT | GO:0005886~plasma membrane | 0.090939589 | 0.99999958 | 1.37 |
| SP_PIR_KEYWORDS | phosphoprotein | 0.093982831 | 0.999999999 | 1.21 |
| GOTERM_BP_FAT | GO:0051336~regulation of hydrolase activity | 0.094110551 | 1 | 3.65 |

**Suppl. Table S21** Functional annotation terms for genes unaffected by diabetes (i.e., relative to control) but DOWNregulated with losartan treatment (relative to untreated diabetes, n = 40 terms; ranked by ascending p-value)

| **Category** | **Term** | **PValue** | **Bonferroni** | **Fold Enrichment** |
| --- | --- | --- | --- | --- |
| GOTERM_BP_FAT | GO:0008610~lipid biosynthetic process | 0.001054263 | 0.484401256 | 4.95 |
| GOTERM_MF_FAT | GO:0008238~exopeptidase activity | 0.008909301 | 0.888369424 | 9.26 |
| SP_PIR_KEYWORDS | carboxypeptidase | 0.016258177 | 0.951007577 | 15.22 |
| GOTERM_MF_FAT | GO:0004180~carboxypeptidase activity | 0.019172909 | 0.991287337 | 13.89 |
| UP_SEQ_FEATURE | compositionally biased region:Pro-rich (proline/acidic region (PAR)) | 0.020458132 | 0.998316907 | 96.22 |
| SP_PIR_KEYWORDS | lipid synthesis | 0.022742357 | 0.985489917 | 6.56 |
| INTERPRO | IPR010600:Inter-alpha-trypsin inhibitor heavy chain, C-terminal | 0.027010376 | 0.999417316 | 72.50 |
| INTERPRO | IPR001628:Zinc finger, nuclear hormone receptor-type | 0.027206934 | 0.999448473 | 11.57 |
| GOTERM_MF_FAT | GO:0008237~metallopeptidase activity | 0.027311314 | 0.998868651 | 4.33 |
| INTERPRO | IPR001723:Steroid hormone receptor | 0.028290495 | 0.9995927 | 11.33 |
| INTERPRO | IPR008946:Nuclear hormone receptor, ligand-binding | 0.029391177 | 0.999700752 | 11.10 |
| INTERPRO | IPR000536:Nuclear hormone receptor, ligand-binding, core | 0.029391177 | 0.999700752 | 11.10 |
| PIR_SUPERFAMILY | PIRSF018524:Noelin/Myocilin | 0.031558504 | 0.894041138 | 61.40 |
| SMART | SM00399:ZnF_C4 | 0.033487537 | 0.916795952 | 10.23 |
| INTERPRO | IPR013088:Zinc finger, NHR/GATA-type | 0.03396083 | 0.999917097 | 10.26 |
| GOTERM_BP_FAT | GO:0006022~aminoglycan metabolic process | 0.034192871 | 1 | 10.18 |
| GOTERM_MF_FAT | GO:0003707~steroid hormone receptor activity | 0.035880597 | 0.999870565 | 9.92 |
| SMART | SM00430:HOLI | 0.036149257 | 0.931967817 | 9.81 |
| GOTERM_BP_FAT | GO:0030212~hyaluronan metabolic process | 0.038509689 | 1 | 50.42 |
| SP_PIR_KEYWORDS | lyase | 0.038747916 | 0.999304876 | 5.32 |
| UP_SEQ_FEATURE | zinc finger region:NR C4-type | 0.039746766 | 0.999996393 | 9.41 |
| UP_SEQ_FEATURE | DNA-binding region:Nuclear receptor | 0.039746766 | 0.999996393 | 9.41 |
| INTERPRO | IPR013694:Vault protein inter-alpha-trypsin | 0.042868692 | 0.999993328 | 45.31 |
| GOTERM_MF_FAT | GO:0004879~ligand-dependent nuclear receptor activity | 0.045726987 | 0.999989534 | 8.68 |
| UP_SEQ_FEATURE | domain:VIT | 0.04709183 | 0.999999664 | 41.24 |
| GOTERM_BP_FAT | GO:0050892~intestinal absorption | 0.049241075 | 1 | 39.22 |
| GOTERM_BP_FAT | GO:0006354~RNA elongation | 0.049241075 | 1 | 39.22 |
| SP_PIR_KEYWORDS | metalloprotease | 0.060140615 | 0.999988945 | 4.45 |
| GOTERM_MF_FAT | GO:0004857~enzyme inhibitor activity | 0.061010855 | 0.9999998 | 3.33 |
| GOTERM_MF_FAT | GO:0046914~transition metal ion binding | 0.061995689 | 0.999999845 | 1.43 |
| GOTERM_BP_FAT | GO:0009309~amine biosynthetic process | 0.062902021 | 1 | 7.25 |
| GOTERM_MF_FAT | GO:0003711~transcription elongation regulator activity | 0.065069965 | 0.999999931 | 29.46 |
| GOTERM_BP_FAT | GO:0046513~ceramide biosynthetic process | 0.065116901 | 1 | 29.41 |
| SP_PIR_KEYWORDS | zinc | 0.065196393 | 0.999995902 | 1.55 |
| INTERPRO | IPR003112:Olfactomedin-like | 0.068732691 | 0.999999996 | 27.89 |
| GOTERM_BP_FAT | GO:0046520~sphingoid biosynthetic process | 0.070350483 | 1 | 27.15 |
| UP_SEQ_FEATURE | domain:Olfactomedin-like | 0.073007764 | 1 | 26.24 |
| KEGG_PATHWAY | mmu00770:Pantothenate and CoA biosynthesis | 0.073272504 | 0.975975719 | 25.50 |
| GOTERM_BP_FAT | GO:0022600~digestive system process | 0.075555151 | 1 | 25.21 |
| SMART | SM00284:OLF | 0.076908253 | 0.997096811 | 24.65 |
| INTERPRO | IPR011700:Basic leucine zipper | 0.078883523 | 1 | 24.17 |
| GOTERM_MF_FAT | GO:0008270~zinc ion binding | 0.081248209 | 0.999999999 | 1.46 |
| SP_PIR_KEYWORDS | Secreted | 0.091908292 | 0.99999998 | 1.60 |
| GOTERM_BP_FAT | GO:0005976~polysaccharide metabolic process | 0.093718579 | 1 | 5.75 |
| INTERPRO | IPR000324:Vitamin D receptor | 0.098857809 | 1 | 19.08 |

**Suppl. Table S22.** Functional annotation terms associated with genes exhibiting UPregulation in losartan treatment relative to diabetes (n = 193 annotation terms; ranked by ascending p-value)

| **Category** | **Term** | **PValue** | **Bonferroni** | **Fold Enrichment** |
| --- | --- | --- | --- | --- |
| SP_PIR_KEYWORDS | stress response | 1.58E-07 | 3.97E-05 | 19.35 |
| SP_PIR_KEYWORDS | acetylation | 5.13E-07 | 1.29E-04 | 2.15 |
| GOTERM_CC_FAT | GO:0005788~endoplasmic reticulum lumen | 5.57E-05 | 0.010480784 | 10.30 |
| INTERPRO | IPR013126:Heat shock protein 70 | 1.06E-04 | 0.040639522 | 40.88 |
| INTERPRO | IPR001023:Heat shock protein Hsp70 | 1.06E-04 | 0.040639522 | 40.88 |
| INTERPRO | IPR018181:Heat shock protein 70, conserved site | 1.82E-04 | 0.068525697 | 34.59 |
| GOTERM_BP_FAT | GO:0009628~response to abiotic stimulus | 1.90E-04 | 0.185629328 | 4.41 |
| INTERPRO | IPR012290:Fibrinogen, alpha/beta/gamma chain, coiled coil | 2.32E-04 | 0.086560257 | 112.42 |
| GOTERM_CC_FAT | GO:0005577~fibrinogen complex | 2.59E-04 | 0.047749347 | 105.97 |
| GOTERM_MF_FAT | GO:0051082~unfolded protein binding | 3.87E-04 | 0.086326899 | 9.57 |
| SP_PIR_KEYWORDS | Chaperone | 4.59E-04 | 0.10916127 | 5.81 |
| GOTERM_BP_FAT | GO:0033554~cellular response to stress | 6.50E-04 | 0.505374065 | 3.24 |
| GOTERM_BP_FAT | GO:0042060~wound healing | 8.37E-04 | 0.59578609 | 6.29 |
| KEGG_PATHWAY | mmu04610:Complement and coagulation cascades | 8.91E-04 | 0.06881612 | 7.78 |
| GOTERM_CC_FAT | GO:0005615~extracellular space | 9.68E-04 | 0.167272792 | 2.90 |
| SP_PIR_KEYWORDS | phosphoprotein | 0.001122394 | 0.24648184 | 1.35 |
| GOTERM_CC_FAT | GO:0005938~cell cortex | 0.001167743 | 0.198148949 | 5.89 |
| SP_PIR_KEYWORDS | plasma | 0.001313238 | 0.281905117 | 18.14 |
| KEGG_PATHWAY | mmu04612:Antigen processing and presentation | 0.002122804 | 0.156339233 | 6.41 |
| GOTERM_CC_FAT | GO:0044421~extracellular region part | 0.002250218 | 0.346733714 | 2.33 |
| BIOCARTA | m_p53Pathway:p53 Signaling Pathway | 0.002399063 | 0.108895329 | 13.31 |
| UP_SEQ_FEATURE | short sequence motif:Prevents secretion from ER | 0.003042621 | 0.816826887 | 8.28 |
| PIR_SUPERFAMILY | PIRSF002581:chaperone HSP70 | 0.003244879 | 0.302858744 | 33.53 |
| GOTERM_BP_FAT | GO:0030003~cellular cation homeostasis | 0.003939826 | 0.986036541 | 4.64 |
| GOTERM_CC_FAT | GO:0070013~intracellular organelle lumen | 0.004046544 | 0.535293116 | 1.96 |
| GOTERM_CC_FAT | GO:0043233~organelle lumen | 0.00416897 | 0.545965602 | 1.96 |
| GOTERM_BP_FAT | GO:0006873~cellular ion homeostasis | 0.004332602 | 0.990887061 | 3.47 |
| GOTERM_BP_FAT | GO:0045807~positive regulation of endocytosis | 0.004488177 | 0.992304643 | 11.84 |
| GOTERM_BP_FAT | GO:0055082~cellular chemical homeostasis | 0.005071914 | 0.995920475 | 3.38 |
| GOTERM_BP_FAT | GO:0051726~regulation of cell cycle | 0.005299197 | 0.996813961 | 3.76 |
| GOTERM_CC_FAT | GO:0044432~endoplasmic reticulum part | 0.005968588 | 0.677432543 | 3.67 |
| GOTERM_CC_FAT | GO:0031974~membrane-enclosed lumen | 0.006007454 | 0.679807497 | 1.90 |
| INTERPRO | IPR000886:Endoplasmic reticulum, targeting sequence | 0.006453198 | 0.920450578 | 10.46 |
| SP_PIR_KEYWORDS | molecular chaperone | 0.007014445 | 0.830324724 | 23.33 |
| GOTERM_BP_FAT | GO:0051258~protein polymerization | 0.007099085 | 0.999551052 | 10.07 |
| GOTERM_BP_FAT | GO:0051130~positive regulation of cellular component organization | 0.007151543 | 0.999575998 | 4.95 |
| GOTERM_BP_FAT | GO:0006461~protein complex assembly | 0.007252959 | 0.999620365 | 3.55 |
| GOTERM_BP_FAT | GO:0070271~protein complex biogenesis | 0.007252959 | 0.999620365 | 3.55 |
| GOTERM_BP_FAT | GO:0009611~response to wounding | 0.007431461 | 0.999687488 | 2.90 |
| INTERPRO | IPR011701:Major facilitator superfamily MFS-1 | 0.008018361 | 0.957054197 | 6.32 |
| GOTERM_CC_FAT | GO:0048770~pigment granule | 0.008239361 | 0.7906379 | 6.23 |
| GOTERM_CC_FAT | GO:0042470~melanosome | 0.008239361 | 0.7906379 | 6.23 |
| GOTERM_BP_FAT | GO:0006457~protein folding | 0.008437175 | 0.999895649 | 4.76 |
| KEGG_PATHWAY | mmu05219:Bladder cancer | 0.008510299 | 0.495272922 | 9.26 |
| GOTERM_BP_FAT | GO:0050801~ion homeostasis | 0.008523972 | 0.99990508 | 3.09 |
| SP_PIR_KEYWORDS | ubl conjugation | 0.008776669 | 0.891551903 | 2.49 |
| GOTERM_BP_FAT | GO:0060627~regulation of vesicle-mediated transport | 0.009115869 | 0.999950255 | 6.06 |
| SP_PIR_KEYWORDS | endoplasmic reticulum | 0.009390462 | 0.907224927 | 2.25 |
| GOTERM_BP_FAT | GO:0007605~sensory perception of sound | 0.009500034 | 0.999967301 | 5.99 |
| GOTERM_BP_FAT | GO:0055080~cation homeostasis | 0.009786907 | 0.999976099 | 3.83 |
| GOTERM_BP_FAT | GO:0030005~cellular di-, tri-valent inorganic cation homeostasis | 0.010487735 | 0.99998889 | 4.51 |
| GOTERM_BP_FAT | GO:0030168~platelet activation | 0.010581447 | 0.999989972 | 18.87 |
| GOTERM_BP_FAT | GO:0010033~response to organic substance | 0.011305276 | 0.999995457 | 2.39 |
| GOTERM_BP_FAT | GO:0050878~regulation of body fluid levels | 0.011578806 | 0.999996633 | 5.65 |
| KEGG_PATHWAY | mmu05215:Prostate cancer | 0.012605037 | 0.637529548 | 5.40 |
| GOTERM_BP_FAT | GO:0050954~sensory perception of mechanical stimulus | 0.012955986 | 0.999999255 | 5.47 |
| INTERPRO | IPR001134:Netrin domain | 0.013286971 | 0.994646628 | 16.86 |
| GOTERM_CC_FAT | GO:0005829~cytosol | 0.013744007 | 0.926878541 | 2.32 |
| GOTERM_MF_FAT | GO:0003779~actin binding | 0.014297011 | 0.965099223 | 3.10 |
| GOTERM_BP_FAT | GO:0030100~regulation of endocytosis | 0.014579064 | 0.999999874 | 7.74 |
| KEGG_PATHWAY | mmu05416:Viral myocarditis | 0.014603162 | 0.691757064 | 5.17 |
| GOTERM_BP_FAT | GO:0055066~di-, tri-valent inorganic cation homeostasis | 0.014777788 | 0.999999899 | 4.14 |
| GOTERM_BP_FAT | GO:0048535~lymph node development | 0.014790787 | 0.999999901 | 15.89 |
| GOTERM_BP_FAT | GO:0051604~protein maturation | 0.014948917 | 0.999999916 | 5.24 |
| GOTERM_BP_FAT | GO:0055085~transmembrane transport | 0.015657728 | 0.999999962 | 2.41 |
| UP_SEQ_FEATURE | domain:NTR | 0.017334411 | 0.999941115 | 14.65 |
| SP_PIR_KEYWORDS | cell motility | 0.018176371 | 0.990172638 | 108.87 |
| SP_PIR_KEYWORDS | microfilament | 0.018176371 | 0.990172638 | 108.87 |
| SP_PIR_KEYWORDS | liver | 0.018483081 | 0.990916711 | 14.20 |
| GOTERM_BP_FAT | GO:0007263~nitric oxide mediated signal transduction | 0.019626752 | 1 | 100.65 |
| GOTERM_BP_FAT | GO:0019725~cellular homeostasis | 0.020333812 | 1 | 2.64 |
| GOTERM_MF_FAT | GO:0004857~enzyme inhibitor activity | 0.021236165 | 0.993270946 | 3.22 |
| GOTERM_BP_FAT | GO:0051270~regulation of cell motion | 0.021391567 | 1 | 4.70 |
| INTERPRO | IPR002067:Mitochondrial carrier protein | 0.021956831 | 0.999830181 | 12.97 |
| PIR_SUPERFAMILY | PIRSF005695:dynein light chain | 0.022002872 | 0.915380331 | 89.41 |
| GOTERM_BP_FAT | GO:0043623~cellular protein complex assembly | 0.022049385 | 1 | 4.66 |
| GOTERM_BP_FAT | GO:0030036~actin cytoskeleton organization | 0.02372269 | 1 | 3.66 |
| GOTERM_BP_FAT | GO:0008217~regulation of blood pressure | 0.026282226 | 1 | 6.19 |
| GOTERM_BP_FAT | GO:0034976~response to endoplasmic reticulum stress | 0.026879996 | 1 | 11.61 |
| SP_PIR_KEYWORDS | structural protein | 0.027141039 | 0.999025974 | 72.58 |
| GOTERM_BP_FAT | GO:0048878~chemical homeostasis | 0.028135844 | 1 | 2.48 |
| GOTERM_BP_FAT | GO:0010273~detoxification of copper ion | 0.029296266 | 1 | 67.10 |
| UP_SEQ_FEATURE | domain:POLO box 1 | 0.030214819 | 0.999999962 | 65.13 |
| UP_SEQ_FEATURE | domain:POLO box 2 | 0.030214819 | 0.999999962 | 65.13 |
| UP_SEQ_FEATURE | domain:Anaphylatoxin-like | 0.030214819 | 0.999999962 | 65.13 |
| UP_SEQ_FEATURE | region of interest:Beta | 0.030214819 | 0.999999962 | 65.13 |
| UP_SEQ_FEATURE | region of interest:Alpha | 0.030214819 | 0.999999962 | 65.13 |
| GOTERM_BP_FAT | GO:0030029~actin filament-based process | 0.030220539 | 1 | 3.43 |
| GOTERM_BP_FAT | GO:0007596~blood coagulation | 0.031796871 | 1 | 5.75 |
| GOTERM_BP_FAT | GO:0050817~coagulation | 0.031796871 | 1 | 5.75 |
| SP_PIR_KEYWORDS | blocked amino end | 0.032398777 | 0.999751404 | 10.54 |
| GOTERM_BP_FAT | GO:0007599~hemostasis | 0.032966926 | 1 | 5.67 |
| GOTERM_MF_FAT | GO:0030674~protein binding, bridging | 0.034516951 | 0.999721064 | 10.15 |
| INTERPRO | IPR018081:Anaphylatoxin | 0.034891327 | 0.999999068 | 56.21 |
| INTERPRO | IPR000006:Metallothionein, vertebrate | 0.034891327 | 0.999999068 | 56.21 |
| INTERPRO | IPR003019:Metallothionein superfamily, eukaryotic | 0.034891327 | 0.999999068 | 56.21 |
| INTERPRO | IPR015728:Polo-like kinase-related | 0.034891327 | 0.999999068 | 56.21 |
| INTERPRO | IPR019763:Dynein light chain, type 1 and 2, conserved site | 0.034891327 | 0.999999068 | 56.21 |
| INTERPRO | IPR001840:Complement C3a/C4a/C5a anaphylatoxin | 0.034891327 | 0.999999068 | 56.21 |
| INTERPRO | IPR001372:Dynein light chain, type 1 and 2 | 0.034891327 | 0.999999068 | 56.21 |
| INTERPRO | IPR018064:Metallothionein, vertebrate, metal binding site | 0.034891327 | 0.999999068 | 56.21 |
| INTERPRO | IPR017450:POLO box | 0.034891327 | 0.999999068 | 56.21 |
| GOTERM_MF_FAT | GO:0042801~polo kinase kinase activity | 0.035054323 | 0.999754999 | 55.83 |
| GOTERM_BP_FAT | GO:0051100~negative regulation of binding | 0.035072781 | 1 | 10.07 |
| SP_PIR_KEYWORDS | cytoplasm | 0.035341913 | 0.999884628 | 1.37 |
| KEGG_PATHWAY | mmu03040:Spliceosome | 0.035985055 | 0.946703177 | 3.92 |
| SP_PIR_KEYWORDS | heat shock | 0.036024351 | 0.999903473 | 54.43 |
| SP_PIR_KEYWORDS | metal-thiolate cluster | 0.036024351 | 0.999903473 | 54.43 |
| SP_PIR_KEYWORDS | stress-induced protein | 0.036024351 | 0.999903473 | 54.43 |
| GOTERM_BP_FAT | GO:0042359~vitamin D metabolic process | 0.038871113 | 1 | 50.33 |
| KEGG_PATHWAY | mmu04110:Cell cycle | 0.039733283 | 0.960974352 | 3.80 |
| UP_SEQ_FEATURE | metal ion-binding site:Divalent metal cation; cluster A | 0.040083404 | 1 | 48.84 |
| UP_SEQ_FEATURE | metal ion-binding site:Divalent metal cation; cluster B | 0.040083404 | 1 | 48.84 |
| KEGG_PATHWAY | mmu05220:Chronic myeloid leukemia | 0.040981769 | 0.964831887 | 5.12 |
| KEGG_PATHWAY | mmu04520:Adherens junction | 0.040981769 | 0.964831887 | 5.12 |
| GOTERM_BP_FAT | GO:0051050~positive regulation of transport | 0.041603607 | 1 | 3.81 |
| GOTERM_BP_FAT | GO:0043627~response to estrogen stimulus | 0.041767624 | 1 | 9.15 |
| INTERPRO | IPR000959:POLO box duplicated region | 0.043423455 | 0.999999971 | 44.97 |
| INTERPRO | IPR014715:Fibrinogen, alpha/beta/gamma chain, C-terminal globular, subdomain 2 | 0.043423455 | 0.999999971 | 44.97 |
| PIR_SUPERFAMILY | PIRSF002564:metallothionein | 0.043526879 | 0.992843883 | 44.70 |
| GOTERM_BP_FAT | GO:0045787~positive regulation of cell cycle | 0.04409759 | 1 | 8.88 |
| SP_PIR_KEYWORDS | blood coagulation | 0.044791762 | 0.999990346 | 8.83 |
| SP_PIR_KEYWORDS | metal binding | 0.044827042 | 0.999990435 | 43.55 |
| GOTERM_BP_FAT | GO:0048002~antigen processing and presentation of peptide antigen | 0.046474782 | 1 | 8.63 |
| GOTERM_MF_FAT | GO:0005198~structural molecule activity | 0.047071977 | 0.999986787 | 2.23 |
| GOTERM_CC_FAT | GO:0005783~endoplasmic reticulum | 0.04932809 | 0.999929564 | 1.77 |
| GOTERM_BP_FAT | GO:0065003~macromolecular complex assembly | 0.050035386 | 1 | 2.38 |
| GOTERM_BP_FAT | GO:0009314~response to radiation | 0.053026989 | 1 | 3.52 |
| SMART | SM00104:ANATO | 0.053193669 | 0.976984545 | 36.23 |
| GOTERM_BP_FAT | GO:0051789~response to protein stimulus | 0.054655801 | 1 | 4.63 |
| GOTERM_CC_FAT | GO:0043034~costamere | 0.054855714 | 0.999976604 | 35.32 |
| GOTERM_MF_FAT | GO:0004866~endopeptidase inhibitor activity | 0.055200662 | 0.999998205 | 3.47 |
| GOTERM_BP_FAT | GO:0009612~response to mechanical stimulus | 0.056433223 | 1 | 7.74 |
| SP_PIR_KEYWORDS | actin-binding | 0.056520996 | 0.999999571 | 2.89 |
| GOTERM_CC_FAT | GO:0044429~mitochondrial part | 0.057017953 | 0.999984824 | 2.02 |
| GOTERM_CC_FAT | GO:0031966~mitochondrial membrane | 0.057314458 | 0.9999857 | 2.30 |
| GOTERM_BP_FAT | GO:0016485~protein processing | 0.059324441 | 1 | 4.47 |
| INTERPRO | IPR000020:Anaphylatoxin/fibulin | 0.060263507 | 1 | 32.12 |
| GOTERM_MF_FAT | GO:0005159~insulin-like growth factor receptor binding | 0.060542934 | 0.999999521 | 31.90 |
| GOTERM_CC_FAT | GO:0030863~cortical cytoskeleton | 0.061141334 | 0.999993371 | 7.39 |
| SP_PIR_KEYWORDS | thioester bond | 0.062193461 | 0.999999906 | 31.10 |
| GOTERM_CC_FAT | GO:0005576~extracellular region | 0.062405555 | 0.999994861 | 1.45 |
| GOTERM_CC_FAT | GO:0005770~late endosome | 0.063671855 | 0.99999602 | 7.22 |
| GOTERM_BP_FAT | GO:0006974~response to DNA damage stimulus | 0.064760179 | 1 | 2.45 |
| GOTERM_BP_FAT | GO:0006882~cellular zinc ion homeostasis | 0.06703681 | 1 | 28.76 |
| GOTERM_BP_FAT | GO:0046688~response to copper ion | 0.06703681 | 1 | 28.76 |
| INTERPRO | IPR019565:Alpha-2-macroglobulin, thiol-ester bond-forming | 0.068572733 | 1 | 28.11 |
| UP_SEQ_FEATURE | cross-link:Isoglutamyl cysteine thioester (Cys-Gln) | 0.06909428 | 1 | 27.91 |
| UP_SEQ_FEATURE | region of interest:Interaction with calmodulin | 0.06909428 | 1 | 27.91 |
| GOTERM_BP_FAT | GO:0007346~regulation of mitotic cell cycle | 0.069202565 | 1 | 4.19 |
| GOTERM_MF_FAT | GO:0005509~calcium ion binding | 0.069395556 | 0.999999947 | 1.73 |
| INTERPRO | IPR001993:Mitochondrial substrate carrier | 0.069703528 | 1 | 6.88 |
| GOTERM_BP_FAT | GO:0043933~macromolecular complex subunit organization | 0.071161789 | 1 | 2.19 |
| GOTERM_MF_FAT | GO:0030414~peptidase inhibitor activity | 0.071647837 | 0.99999997 | 3.17 |
| KEGG_PATHWAY | mmu00520:Amino sugar and nucleotide sugar metabolism | 0.072506638 | 0.997574145 | 6.63 |
| GOTERM_BP_FAT | GO:0031668~cellular response to extracellular stimulus | 0.072601325 | 1 | 6.71 |
| UP_SEQ_FEATURE | repeat:Solcar 3 | 0.073733564 | 1 | 6.66 |
| GOTERM_CC_FAT | GO:0005740~mitochondrial envelope | 0.074072132 | 0.999999518 | 2.17 |
| GOTERM_MF_FAT | GO:0008092~cytoskeletal protein binding | 0.075652947 | 0.999999989 | 2.16 |
| GOTERM_BP_FAT | GO:0006875~cellular metal ion homeostasis | 0.076175242 | 1 | 4.03 |
| GOTERM_BP_FAT | GO:0006417~regulation of translation | 0.076175242 | 1 | 4.03 |
| GOTERM_BP_FAT | GO:0055069~zinc ion homeostasis | 0.076242102 | 1 | 25.16 |
| GOTERM_BP_FAT | GO:0009651~response to salt stress | 0.076242102 | 1 | 25.16 |
| INTERPRO | IPR011626:A-macroglobulin complement component | 0.076808953 | 1 | 24.98 |
| INTERPRO | IPR009048:Alpha-macroglobulin, receptor-binding | 0.076808953 | 1 | 24.98 |
| INTERPRO | IPR001599:Alpha-2-macroglobulin | 0.076808953 | 1 | 24.98 |
| INTERPRO | IPR011625:Alpha-2-macroglobulin, N-terminal 2 | 0.076808953 | 1 | 24.98 |
| INTERPRO | IPR018108:Mitochondrial substrate/solute carrier | 0.077276822 | 1 | 6.49 |
| GOTERM_CC_FAT | GO:0043228~non-membrane-bounded organelle | 0.080229088 | 0.999999863 | 1.38 |
| GOTERM_CC_FAT | GO:0043232~intracellular non-membrane-bounded organelle | 0.080229088 | 0.999999863 | 1.38 |
| GOTERM_CC_FAT | GO:0005771~multivesicular body | 0.081153982 | 0.999999887 | 23.55 |
| GOTERM_CC_FAT | GO:0000307~cyclin-dependent protein kinase holoenzyme complex | 0.081153982 | 0.999999887 | 23.55 |
| UP_SEQ_FEATURE | repeat:Solcar 1 | 0.082632652 | 1 | 6.24 |
| UP_SEQ_FEATURE | repeat:Solcar 2 | 0.082632652 | 1 | 6.24 |
| INTERPRO | IPR002890:Alpha-2-macroglobulin, N-terminal | 0.084972803 | 1 | 22.48 |
| GOTERM_BP_FAT | GO:0050910~detection of mechanical stimulus involved in sensory perception of sound | 0.085357239 | 1 | 22.37 |
| KEGG_PATHWAY | mmu05322:Systemic lupus erythematosus | 0.085399068 | 0.999208354 | 3.78 |
| GOTERM_BP_FAT | GO:0055065~metal ion homeostasis | 0.087188603 | 1 | 3.80 |
| SP_PIR_KEYWORDS | methylated amino acid | 0.087656397 | 1 | 21.77 |
| SP_PIR_KEYWORDS | Schiff base | 0.087656397 | 1 | 21.77 |
| SP_PIR_KEYWORDS | immune response | 0.088081245 | 1 | 2.96 |
| GOTERM_BP_FAT | GO:0051325~interphase | 0.090052311 | 1 | 5.92 |
| GOTERM_MF_FAT | GO:0019842~vitamin binding | 0.092842864 | 1 | 3.69 |
| INTERPRO | IPR019742:Alpha-2-macroglobulin, conserved site | 0.093064916 | 1 | 20.44 |
| GOTERM_BP_FAT | GO:0030866~cortical actin cytoskeleton organization | 0.094383097 | 1 | 20.13 |
| GOTERM_BP_FAT | GO:0040012~regulation of locomotion | 0.094883978 | 1 | 3.66 |
| PIR_SUPERFAMILY | PIRSF001635:alpha-2-macroglobulin | 0.095308969 | 0.999985157 | 19.87 |
| SP_PIR_KEYWORDS | thiolester bond | 0.095990403 | 1 | 19.79 |
| GOTERM_BP_FAT | GO:0010038~response to metal ion | 0.096119348 | 1 | 5.70 |
| GOTERM_BP_FAT | GO:0042981~regulation of apoptosis | 0.096277869 | 1 | 1.82 |
| GOTERM_BP_FAT | GO:0003013~circulatory system process | 0.096850293 | 1 | 3.63 |
| GOTERM_BP_FAT | GO:0008015~blood circulation | 0.096850293 | 1 | 3.63 |
| GOTERM_BP_FAT | GO:0006986~response to unfolded protein | 0.099195774 | 1 | 5.59 |

**Suppl. Table S23** Functional annotation terms associated with genes exhibiting DOWNregulation with losartan treatment relative to untreated diabetes (n = 45 annotation terms; ranked by ascending p-value)

| **Category** | **Term** | **PValue** | **Bonferroni** | **Fold Enrichment** |
| --- | --- | --- | --- | --- |
| SP_PIR_KEYWORDS | stress response | 5.36E-09 | 7.12E-07 | 48.72 |
| GOTERM_CC_FAT | GO:0005788~endoplasmic reticulum lumen | 8.28E-08 | 9.11E-06 | 30.39 |
| SP_PIR_KEYWORDS | acetylation | 3.26E-06 | 4.33E-04 | 2.96 |
| INTERPRO | IPR001023:Heat shock protein Hsp70 | 4.55E-06 | 7.10E-04 | 115.34 |
| INTERPRO | IPR013126:Heat shock protein 70 | 4.55E-06 | 7.10E-04 | 115.34 |
| INTERPRO | IPR018181:Heat shock protein 70, conserved site | 7.86E-06 | 0.001225293 | 97.60 |
| UP_SEQ_FEATURE | short sequence motif:Prevents secretion from ER | 5.28E-05 | 0.012751715 | 23.82 |
| GOTERM_CC_FAT | GO:0044432~endoplasmic reticulum part | 7.30E-05 | 0.007994487 | 9.47 |
| GOTERM_MF_FAT | GO:0051082~unfolded protein binding | 8.18E-05 | 0.010331368 | 21.09 |
| SP_PIR_KEYWORDS | Chaperone | 1.06E-04 | 0.013988042 | 12.53 |
| SP_PIR_KEYWORDS | phosphoprotein | 2.64E-04 | 0.034558578 | 1.69 |
| INTERPRO | IPR000886:Endoplasmic reticulum, targeting sequence | 3.18E-04 | 0.04833638 | 29.51 |
| PIR_SUPERFAMILY | PIRSF002581:chaperone HSP70 | 4.14E-04 | 0.01722115 | 92.45 |
| GOTERM_CC_FAT | GO:0070013~intracellular organelle lumen | 4.99E-04 | 0.053473989 | 3.31 |
| GOTERM_CC_FAT | GO:0043233~organelle lumen | 5.11E-04 | 0.054664553 | 3.30 |
| GOTERM_CC_FAT | GO:0031974~membrane-enclosed lumen | 6.76E-04 | 0.071693438 | 3.20 |
| SP_PIR_KEYWORDS | molecular chaperone | 8.58E-04 | 0.107922704 | 67.12 |
| GOTERM_BP_FAT | GO:0006457~protein folding | 0.00111394 | 0.421460977 | 10.70 |
| SP_PIR_KEYWORDS | endoplasmic reticulum | 0.001176684 | 0.144946364 | 4.16 |
| KEGG_PATHWAY | mmu04612:Antigen processing and presentation | 0.001529405 | 0.04341603 | 15.76 |
| GOTERM_CC_FAT | GO:0042470~melanosome | 0.002322164 | 0.225652902 | 14.71 |
| GOTERM_CC_FAT | GO:0048770~pigment granule | 0.002322164 | 0.225652902 | 14.71 |
| GOTERM_CC_FAT | GO:0005783~endoplasmic reticulum | 0.003765367 | 0.339641315 | 3.36 |
| GOTERM_CC_FAT | GO:0005829~cytosol | 0.006614908 | 0.518118585 | 3.99 |
| SP_PIR_KEYWORDS | stress-induced protein | 0.012488348 | 0.812017934 | 156.61 |
| SP_PIR_KEYWORDS | heat shock | 0.012488348 | 0.812017934 | 156.61 |
| GOTERM_BP_FAT | GO:0030003~cellular cation homeostasis | 0.017370693 | 0.999816621 | 7.15 |
| SP_PIR_KEYWORDS | atp-binding | 0.017931767 | 0.909875847 | 2.43 |
| GOTERM_MF_FAT | GO:0000166~nucleotide binding | 0.02124421 | 0.934590364 | 1.89 |
| GOTERM_BP_FAT | GO:0030036~actin cytoskeleton organization | 0.021544477 | 0.999977318 | 6.59 |
| GOTERM_BP_FAT | GO:0030029~actin filament-based process | 0.025469602 | 0.999996848 | 6.18 |
| GOTERM_MF_FAT | GO:0019904~protein domain specific binding | 0.025494317 | 0.962362935 | 6.15 |
| GOTERM_BP_FAT | GO:0055080~cation homeostasis | 0.028550947 | 0.999999334 | 5.91 |
| SP_PIR_KEYWORDS | nucleotide-binding | 0.028887721 | 0.979730818 | 2.11 |
| GOTERM_MF_FAT | GO:0032555~purine ribonucleotide binding | 0.029117187 | 0.976547711 | 1.97 |
| GOTERM_MF_FAT | GO:0032553~ribonucleotide binding | 0.029117187 | 0.976547711 | 1.97 |
| GOTERM_BP_FAT | GO:0007010~cytoskeleton organization | 0.029576937 | 0.999999604 | 4.17 |
| UP_SEQ_FEATURE | cross-link:Glycyl lysine isopeptide (Lys-Gly) (interchain with G-Cter in SUMO) | 0.037931284 | 0.999917 | 9.58 |
| GOTERM_MF_FAT | GO:0017076~purine nucleotide binding | 0.037963905 | 0.99266663 | 1.89 |
| GOTERM_CC_FAT | GO:0016023~cytoplasmic membrane-bounded vesicle | 0.03913362 | 0.987614038 | 3.78 |
| KEGG_PATHWAY | mmu03040:Spliceosome | 0.040482429 | 0.698327976 | 8.68 |
| GOTERM_CC_FAT | GO:0031988~membrane-bounded vesicle | 0.04091615 | 0.989902274 | 3.72 |
| GOTERM_BP_FAT | GO:0051085~chaperone mediated protein folding requiring cofactor | 0.042442281 | 0.999999999 | 45.29 |
| GOTERM_MF_FAT | GO:0005524~ATP binding | 0.044010572 | 0.996707374 | 2.05 |
| GOTERM_MF_FAT | GO:0032559~adenyl ribonucleotide binding | 0.046889968 | 0.997755259 | 2.02 |
| GOTERM_BP_FAT | GO:0006458~'de novo' protein folding | 0.049342307 | 1 | 38.82 |
| GOTERM_BP_FAT | GO:0051084~'de novo' posttranslational protein folding | 0.049342307 | 1 | 38.82 |
| GOTERM_BP_FAT | GO:0006875~cellular metal ion homeostasis | 0.050424149 | 1 | 8.15 |
| GOTERM_BP_FAT | GO:0045664~regulation of neuron differentiation | 0.052235718 | 1 | 7.99 |
| GOTERM_CC_FAT | GO:0045120~pronucleus | 0.05471432 | 0.997949127 | 34.73 |
| GOTERM_BP_FAT | GO:0055065~metal ion homeostasis | 0.055928032 | 1 | 7.69 |
| GOTERM_BP_FAT | GO:0033554~cellular response to stress | 0.057206376 | 1 | 3.36 |
| GOTERM_MF_FAT | GO:0030554~adenyl nucleotide binding | 0.061129832 | 0.999668179 | 1.92 |
| GOTERM_MF_FAT | GO:0001883~purine nucleoside binding | 0.063859867 | 0.99977076 | 1.91 |
| GOTERM_MF_FAT | GO:0001882~nucleoside binding | 0.066013654 | 0.999828901 | 1.90 |
| GOTERM_BP_FAT | GO:0045428~regulation of nitric oxide biosynthetic process | 0.066379987 | 1 | 28.61 |
| GOTERM_BP_FAT | GO:0006873~cellular ion homeostasis | 0.067689622 | 1 | 4.16 |
| GOTERM_BP_FAT | GO:0055082~cellular chemical homeostasis | 0.072055578 | 1 | 4.06 |
| GOTERM_CC_FAT | GO:0031410~cytoplasmic vesicle | 0.072355732 | 0.999741797 | 3.08 |
| GOTERM_BP_FAT | GO:0030199~collagen fibril organization | 0.073110986 | 1 | 25.88 |
| GOTERM_CC_FAT | GO:0031982~vesicle | 0.076972606 | 0.999850856 | 3.01 |
| SP_PIR_KEYWORDS | viral nucleoprotein | 0.081379246 | 0.999987494 | 23.20 |
| SP_PIR_KEYWORDS | ubl conjugation | 0.081711046 | 0.999988081 | 2.99 |
| GOTERM_BP_FAT | GO:0050767~regulation of neurogenesis | 0.081969823 | 1 | 6.18 |
| GOTERM_BP_FAT | GO:0001818~negative regulation of cytokine production | 0.083118441 | 1 | 22.65 |
| GOTERM_BP_FAT | GO:0030005~cellular di-, tri-valent inorganic cation homeostasis | 0.084105488 | 1 | 6.08 |
| GOTERM_BP_FAT | GO:0050801~ion homeostasis | 0.088649519 | 1 | 3.71 |
| GOTERM_BP_FAT | GO:0031669~cellular response to nutrient levels | 0.096297223 | 1 | 19.41 |
| GOTERM_BP_FAT | GO:0055066~di-, tri-valent inorganic cation homeostasis | 0.097266988 | 1 | 5.58 |
| GOTERM_BP_FAT | GO:0051960~regulation of nervous system development | 0.09951536 | 1 | 5.51 |

**Suppl. Table S24** Functional annotation terms associated with genes exhibiting DYSregulation (up- OR down-regulation) with losartan treatment relative to untreated diabetes (n = 209 annotation terms; ranked by ascending p-value)

| **Category** | **Term** | **PValue** | **Bonferroni** | **Fold Enrichment** |
| --- | --- | --- | --- | --- |
| SP_PIR_KEYWORDS | stress response | 3.56E-07 | 1.13E-04 | 13.03 |
| GOTERM_CC_FAT | GO:0005788~endoplasmic reticulum lumen | 9.38E-05 | 0.019965278 | 7.43 |
| INTERPRO | IPR001023:Heat shock protein Hsp70 | 4.43E-04 | 0.230055848 | 25.23 |
| INTERPRO | IPR013126:Heat shock protein 70 | 4.43E-04 | 0.230055848 | 25.23 |
| SP_PIR_KEYWORDS | plasma | 4.46E-04 | 0.1319896 | 13.58 |
| INTERPRO | IPR012290:Fibrinogen, alpha/beta/gamma chain, coiled coil | 6.10E-04 | 0.302338138 | 69.39 |
| GOTERM_CC_FAT | GO:0005615~extracellular space | 6.13E-04 | 0.123435979 | 2.49 |
| GOTERM_CC_FAT | GO:0005577~fibrinogen complex | 6.54E-04 | 0.131178877 | 66.87 |
| INTERPRO | IPR018181:Heat shock protein 70, conserved site | 7.52E-04 | 0.358334406 | 21.35 |
| KEGG_PATHWAY | mmu04610:Complement and coagulation cascades | 9.46E-04 | 0.092838737 | 6.02 |
| GOTERM_MF_FAT | GO:0004857~enzyme inhibitor activity | 0.001137926 | 0.343788537 | 3.26 |
| SP_PIR_KEYWORDS | carboxypeptidase | 0.001372599 | 0.353002443 | 10.18 |
| GOTERM_BP_FAT | GO:0033554~cellular response to stress | 0.001632494 | 0.896286188 | 2.54 |
| INTERPRO | IPR011701:Major facilitator superfamily MFS-1 | 0.001774787 | 0.649380816 | 5.46 |
| GOTERM_BP_FAT | GO:0042060~wound healing | 0.001802417 | 0.918096096 | 4.58 |
| GOTERM_MF_FAT | GO:0004180~carboxypeptidase activity | 0.001809653 | 0.488382529 | 9.44 |
| GOTERM_BP_FAT | GO:0009628~response to abiotic stimulus | 0.001899474 | 0.928429758 | 3.06 |
| SP_PIR_KEYWORDS | Chaperone | 0.002190026 | 0.500925372 | 3.91 |
| GOTERM_BP_FAT | GO:0030003~cellular cation homeostasis | 0.002572511 | 0.971919243 | 3.80 |
| GOTERM_CC_FAT | GO:0044421~extracellular region part | 0.002588652 | 0.427236146 | 1.99 |
| INTERPRO | IPR001134:Netrin domain | 0.00278206 | 0.806736876 | 13.88 |
| SP_PIR_KEYWORDS | acetylation | 0.002905457 | 0.60242456 | 1.49 |
| GOTERM_MF_FAT | GO:0051082~unfolded protein binding | 0.004028983 | 0.775467466 | 5.67 |
| GOTERM_MF_FAT | GO:0008238~exopeptidase activity | 0.004028983 | 0.775467466 | 5.67 |
| UP_SEQ_FEATURE | domain:NTR | 0.004547166 | 0.972563894 | 11.65 |
| GOTERM_BP_FAT | GO:0030005~cellular di-, tri-valent inorganic cation homeostasis | 0.004911064 | 0.99891744 | 3.83 |
| SP_PIR_KEYWORDS | liver | 0.004993266 | 0.795426981 | 11.33 |
| GOTERM_CC_FAT | GO:0070013~intracellular organelle lumen | 0.00513069 | 0.669099579 | 1.71 |
| GOTERM_CC_FAT | GO:0043233~organelle lumen | 0.005318412 | 0.682256301 | 1.71 |
| BIOCARTA | m_p53Pathway:p53 Signaling Pathway | 0.006162698 | 0.305611844 | 9.76 |
| GOTERM_BP_FAT | GO:0006873~cellular ion homeostasis | 0.007689424 | 0.9999776 | 2.70 |
| GOTERM_BP_FAT | GO:0055066~di-, tri-valent inorganic cation homeostasis | 0.007770536 | 0.999980001 | 3.51 |
| PIR_SUPERFAMILY | PIRSF002581:chaperone HSP70 | 0.008013642 | 0.753400746 | 21.19 |
| GOTERM_BP_FAT | GO:0055080~cation homeostasis | 0.008050734 | 0.999986483 | 3.14 |
| GOTERM_CC_FAT | GO:0031974~membrane-enclosed lumen | 0.008241335 | 0.831231755 | 1.65 |
| GOTERM_BP_FAT | GO:0055082~cellular chemical homeostasis | 0.009169312 | 0.999997174 | 2.63 |
| GOTERM_MF_FAT | GO:0019842~vitamin binding | 0.009924425 | 0.975037489 | 3.82 |
| GOTERM_MF_FAT | GO:0004866~endopeptidase inhibitor activity | 0.010976324 | 0.983154858 | 3.28 |
| SP_PIR_KEYWORDS | lyase | 0.011189124 | 0.971758005 | 3.74 |
| GOTERM_CC_FAT | GO:0005938~cell cortex | 0.011311162 | 0.913339603 | 3.71 |
| KEGG_PATHWAY | mmu04612:Antigen processing and presentation | 0.012525448 | 0.726996027 | 4.25 |
| GOTERM_BP_FAT | GO:0055085~transmembrane transport | 0.012737541 | 0.999999981 | 2.09 |
| UP_SEQ_FEATURE | region of interest:Ligand-binding | 0.013018785 | 0.999967662 | 8.04 |
| KEGG_PATHWAY | mmu04110:Cell cycle | 0.013365439 | 0.749907085 | 3.53 |
| GOTERM_CC_FAT | GO:0005576~extracellular region | 0.014349204 | 0.955284144 | 1.47 |
| SP_PIR_KEYWORDS | ubl conjugation | 0.015242493 | 0.992319523 | 1.99 |
| GOTERM_BP_FAT | GO:0045807~positive regulation of endocytosis | 0.01550612 | 1 | 7.54 |
| SMART | SM00643:C345C | 0.015892047 | 0.862817107 | 15.17 |
| GOTERM_BP_FAT | GO:0050801~ion homeostasis | 0.016311897 | 1 | 2.41 |
| INTERPRO | IPR018933:Netrin module, non-TIMP type | 0.016672455 | 0.999950803 | 14.87 |
| GOTERM_BP_FAT | GO:0051604~protein maturation | 0.016842686 | 1 | 4.01 |
| GOTERM_CC_FAT | GO:0005829~cytosol | 0.01706939 | 0.975316037 | 1.95 |
| GOTERM_MF_FAT | GO:0030414~peptidase inhibitor activity | 0.017192703 | 0.998365828 | 3.00 |
| UP_SEQ_FEATURE | short sequence motif:Prevents secretion from ER | 0.01826108 | 0.999999516 | 4.94 |
| GOTERM_BP_FAT | GO:0008217~regulation of blood pressure | 0.018310241 | 1 | 4.93 |
| SP_PIR_KEYWORDS | molecular chaperone | 0.018779347 | 0.997545077 | 13.96 |
| GOTERM_BP_FAT | GO:0006875~cellular metal ion homeostasis | 0.01974947 | 1 | 3.85 |
| GOTERM_BP_FAT | GO:0009611~response to wounding | 0.019789196 | 1 | 2.22 |
| KEGG_PATHWAY | mmu00770:Pantothenate and CoA biosynthesis | 0.021449692 | 0.892831966 | 12.89 |
| GOTERM_CC_FAT | GO:0044432~endoplasmic reticulum part | 0.022250041 | 0.992075381 | 2.61 |
| INTERPRO | IPR000886:Endoplasmic reticulum, targeting sequence | 0.023661657 | 0.999999269 | 6.45 |
| GOTERM_BP_FAT | GO:0051258~protein polymerization | 0.023929579 | 1 | 6.41 |
| GOTERM_BP_FAT | GO:0055065~metal ion homeostasis | 0.024696529 | 1 | 3.63 |
| GOTERM_BP_FAT | GO:0030168~platelet activation | 0.024959681 | 1 | 12.02 |
| GOTERM_BP_FAT | GO:0051270~regulation of cell motion | 0.025591601 | 1 | 3.59 |
| KEGG_PATHWAY | mmu05219:Bladder cancer | 0.026023795 | 0.933857637 | 6.14 |
| GOTERM_MF_FAT | GO:0019904~protein domain specific binding | 0.026211439 | 0.999946058 | 2.75 |
| SMART | SM00399:ZnF_C4 | 0.027880665 | 0.96999262 | 6.02 |
| INTERPRO | IPR017153:Glutathione degradosome, DUG1 | 0.028506077 | 0.999999961 | 69.39 |
| SP_PIR_KEYWORDS | phosphoprotein | 0.028738163 | 0.999903258 | 1.17 |
| INTERPRO | IPR001628:Zinc finger, nuclear hormone receptor-type | 0.02982472 | 0.999999983 | 5.91 |
| SP_PIR_KEYWORDS | cell motility | 0.03034842 | 0.999942828 | 65.16 |
| SP_PIR_KEYWORDS | microfilament | 0.03034842 | 0.999942828 | 65.16 |
| GOTERM_BP_FAT | GO:0007263~nitric oxide mediated signal transduction | 0.030816809 | 1 | 64.09 |
| SMART | SM00430:HOLI | 0.031050025 | 0.979984422 | 5.78 |
| INTERPRO | IPR001723:Steroid hormone receptor | 0.031485096 | 0.999999994 | 5.78 |
| SP_PIR_KEYWORDS | endoplasmic reticulum | 0.032005328 | 0.999966755 | 1.73 |
| INTERPRO | IPR008946:Nuclear hormone receptor, ligand-binding | 0.033193016 | 0.999999998 | 5.66 |
| INTERPRO | IPR000536:Nuclear hormone receptor, ligand-binding, core | 0.033193016 | 0.999999998 | 5.66 |
| GOTERM_BP_FAT | GO:0048535~lymph node development | 0.034504704 | 1 | 10.12 |
| PIR_SUPERFAMILY | PIRSF037242:CNDP_dipeptidase | 0.034845609 | 0.997911466 | 56.50 |
| PIR_SUPERFAMILY | PIRSF005695:dynein light chain | 0.034845609 | 0.997911466 | 56.50 |
| PIR_SUPERFAMILY | PIRSF037242:CNDP dipeptidase | 0.034845609 | 0.997911466 | 56.50 |
| GOTERM_BP_FAT | GO:0006974~response to DNA damage stimulus | 0.035387511 | 1 | 2.23 |
| GOTERM_MF_FAT | GO:0003707~steroid hormone receptor activity | 0.037313561 | 0.999999225 | 5.40 |
| GOTERM_CC_FAT | GO:0048770~pigment granule | 0.037831904 | 0.999749415 | 3.93 |
| GOTERM_CC_FAT | GO:0042470~melanosome | 0.037831904 | 0.999749415 | 3.93 |
| GOTERM_MF_FAT | GO:0015171~amino acid transmembrane transporter activity | 0.039268768 | 0.999999635 | 5.29 |
| GOTERM_BP_FAT | GO:0060627~regulation of vesicle-mediated transport | 0.04016522 | 1 | 3.86 |
| INTERPRO | IPR013088:Zinc finger, NHR/GATA-type | 0.040495813 | 1 | 5.24 |
| GOTERM_BP_FAT | GO:0051130~positive regulation of cellular component organization | 0.041548656 | 1 | 3.15 |
| GOTERM_BP_FAT | GO:0019725~cellular homeostasis | 0.041558229 | 1 | 2.06 |
| GOTERM_BP_FAT | GO:0007605~sensory perception of sound | 0.041680251 | 1 | 3.82 |
| GOTERM_CC_FAT | GO:0005783~endoplasmic reticulum | 0.043523685 | 0.999930023 | 1.60 |
| UP_SEQ_FEATURE | zinc finger region:NR C4-type | 0.043806602 | 1 | 5.07 |
| UP_SEQ_FEATURE | DNA-binding region:Nuclear receptor | 0.043806602 | 1 | 5.07 |
| SP_PIR_KEYWORDS | acetylated amino end | 0.044015183 | 0.999999365 | 8.89 |
| SP_PIR_KEYWORDS | Secreted | 0.044382835 | 0.999999438 | 1.42 |
| SP_PIR_KEYWORDS | structural protein | 0.045176724 | 0.999999568 | 43.44 |
| GOTERM_BP_FAT | GO:0010273~detoxification of copper ion | 0.045868895 | 1 | 42.73 |
| GOTERM_BP_FAT | GO:0051789~response to protein stimulus | 0.046417453 | 1 | 3.68 |
| GOTERM_BP_FAT | GO:0007242~intracellular signaling cascade | 0.04663275 | 1 | 1.54 |
| GOTERM_BP_FAT | GO:0030100~regulation of endocytosis | 0.046810078 | 1 | 4.93 |
| GOTERM_BP_FAT | GO:0006022~aminoglycan metabolic process | 0.046810078 | 1 | 4.93 |
| GOTERM_BP_FAT | GO:0006457~protein folding | 0.047953284 | 1 | 3.03 |
| KEGG_PATHWAY | mmu05215:Prostate cancer | 0.04874098 | 0.994181981 | 3.58 |
| UP_SEQ_FEATURE | region of interest:Hinge | 0.049435002 | 1 | 8.32 |
| GOTERM_BP_FAT | GO:0051726~regulation of cell cycle | 0.049670845 | 1 | 2.40 |
| GOTERM_BP_FAT | GO:0050878~regulation of body fluid levels | 0.049735402 | 1 | 3.60 |
| UP_SEQ_FEATURE | domain:Anaphylatoxin-like | 0.050438263 | 1 | 38.84 |
| UP_SEQ_FEATURE | domain:POLO box 2 | 0.050438263 | 1 | 38.84 |
| UP_SEQ_FEATURE | domain:POLO box 1 | 0.050438263 | 1 | 38.84 |
| UP_SEQ_FEATURE | region of interest:Beta | 0.050438263 | 1 | 38.84 |
| UP_SEQ_FEATURE | region of interest:Alpha | 0.050438263 | 1 | 38.84 |
| UP_SEQ_FEATURE | compositionally biased region:Pro-rich (proline/acidic region (PAR)) | 0.050438263 | 1 | 38.84 |
| GOTERM_BP_FAT | GO:0006986~response to unfolded protein | 0.051383205 | 1 | 4.75 |
| GOTERM_BP_FAT | GO:0016485~protein processing | 0.051442177 | 1 | 3.56 |
| PIR_SUPERFAMILY | PIRSF000344:acyl-CoA desaturase | 0.051813531 | 0.999904608 | 37.67 |
| PIR_SUPERFAMILY | PIRSF009270:carboxypeptidase, M type | 0.051813531 | 0.999904608 | 37.67 |
| GOTERM_MF_FAT | GO:0004879~ligand-dependent nuclear receptor activity | 0.052061419 | 0.999999997 | 4.72 |
| INTERPRO | IPR002067:Mitochondrial carrier protein | 0.053228404 | 1 | 8.01 |
| SMART | SM00103:ALBUMIN | 0.054913475 | 0.999091186 | 35.39 |
| GOTERM_BP_FAT | GO:0050954~sensory perception of mechanical stimulus | 0.054950979 | 1 | 3.48 |
| KEGG_PATHWAY | mmu05416:Viral myocarditis | 0.055587959 | 0.997235376 | 3.43 |
| INTERPRO | IPR017450:POLO box | 0.056202654 | 1 | 34.69 |
| INTERPRO | IPR019763:Dynein light chain, type 1 and 2, conserved site | 0.056202654 | 1 | 34.69 |
| INTERPRO | IPR015876:Fatty acid desaturase, type 1, core | 0.056202654 | 1 | 34.69 |
| INTERPRO | IPR003019:Metallothionein superfamily, eukaryotic | 0.056202654 | 1 | 34.69 |
| INTERPRO | IPR018081:Anaphylatoxin | 0.056202654 | 1 | 34.69 |
| INTERPRO | IPR001840:Complement C3a/C4a/C5a anaphylatoxin | 0.056202654 | 1 | 34.69 |
| INTERPRO | IPR014760:Serum albumin, N-terminal | 0.056202654 | 1 | 34.69 |
| INTERPRO | IPR001261:ArgE/DapE/ACY1/CPG2/YscS, conserved site | 0.056202654 | 1 | 34.69 |
| INTERPRO | IPR000006:Metallothionein, vertebrate | 0.056202654 | 1 | 34.69 |
| INTERPRO | IPR015728:Polo-like kinase-related | 0.056202654 | 1 | 34.69 |
| INTERPRO | IPR001372:Dynein light chain, type 1 and 2 | 0.056202654 | 1 | 34.69 |
| INTERPRO | IPR018064:Metallothionein, vertebrate, metal binding site | 0.056202654 | 1 | 34.69 |
| INTERPRO | IPR001522:Fatty acid desaturase, type 1, C-terminal | 0.056202654 | 1 | 34.69 |
| GOTERM_BP_FAT | GO:0009991~response to extracellular stimulus | 0.05636975 | 1 | 2.89 |
| GOTERM_MF_FAT | GO:0005499~vitamin D binding | 0.058865551 | 1 | 33.05 |
| GOTERM_MF_FAT | GO:0042801~polo kinase kinase activity | 0.058865551 | 1 | 33.05 |
| GOTERM_BP_FAT | GO:0048878~chemical homeostasis | 0.058886388 | 1 | 1.93 |
| SP_PIR_KEYWORDS | complement pathway | 0.059523228 | 0.999999996 | 7.52 |
| SP_PIR_KEYWORDS | stress-induced protein | 0.059779086 | 0.999999997 | 32.58 |
| SP_PIR_KEYWORDS | heat shock | 0.059779086 | 0.999999997 | 32.58 |
| SP_PIR_KEYWORDS | metal-thiolate cluster | 0.059779086 | 0.999999997 | 32.58 |
| SP_PIR_KEYWORDS | vitamin d | 0.059779086 | 0.999999997 | 32.58 |
| GOTERM_BP_FAT | GO:0042359~vitamin D metabolic process | 0.060688304 | 1 | 32.05 |
| GOTERM_BP_FAT | GO:0034976~response to endoplasmic reticulum stress | 0.061125969 | 1 | 7.40 |
| GOTERM_BP_FAT | GO:0006461~protein complex assembly | 0.063557999 | 1 | 2.26 |
| GOTERM_BP_FAT | GO:0070271~protein complex biogenesis | 0.063557999 | 1 | 2.26 |
| GOTERM_MF_FAT | GO:0008237~metallopeptidase activity | 0.06355954 | 1 | 2.47 |
| SP_PIR_KEYWORDS | pyridoxal phosphate | 0.064041592 | 0.999999999 | 4.34 |
| SP_PIR_KEYWORDS | lipid synthesis | 0.065396985 | 1 | 3.29 |
| UP_SEQ_FEATURE | domain:Albumin 3 | 0.066681235 | 1 | 29.13 |
| UP_SEQ_FEATURE | metal ion-binding site:Divalent metal cation; cluster A | 0.066681235 | 1 | 29.13 |
| UP_SEQ_FEATURE | domain:Albumin 1 | 0.066681235 | 1 | 29.13 |
| UP_SEQ_FEATURE | metal ion-binding site:Divalent metal cation; cluster B | 0.066681235 | 1 | 29.13 |
| UP_SEQ_FEATURE | domain:Albumin 2 | 0.066681235 | 1 | 29.13 |
| PIR_SUPERFAMILY | PIRSF002564:metallothionein | 0.068485197 | 0.999995645 | 28.25 |
| GOTERM_MF_FAT | GO:0005275~amine transmembrane transporter activity | 0.069193401 | 1 | 4.20 |
| INTERPRO | IPR002933:Peptidase M20 | 0.069754562 | 1 | 27.75 |
| INTERPRO | IPR000264:Serum albumin | 0.069754562 | 1 | 27.75 |
| INTERPRO | IPR014715:Fibrinogen, alpha/beta/gamma chain, C-terminal globular, subdomain 2 | 0.069754562 | 1 | 27.75 |
| INTERPRO | IPR011650:Peptidase M20, dimerisation | 0.069754562 | 1 | 27.75 |
| INTERPRO | IPR010600:Inter-alpha-trypsin inhibitor heavy chain, C-terminal | 0.069754562 | 1 | 27.75 |
| INTERPRO | IPR000959:POLO box duplicated region | 0.069754562 | 1 | 27.75 |
| GOTERM_MF_FAT | GO:0003779~actin binding | 0.069923832 | 1 | 2.07 |
| GOTERM_MF_FAT | GO:0046983~protein dimerization activity | 0.07197944 | 1 | 1.94 |
| GOTERM_BP_FAT | GO:0009314~response to radiation | 0.072169505 | 1 | 2.69 |
| KEGG_PATHWAY | mmu05322:Systemic lupus erythematosus | 0.07279203 | 0.999583853 | 3.13 |
| SP_PIR_KEYWORDS | metalloprotease | 0.073730749 | 1 | 2.68 |
| SP_PIR_KEYWORDS | metal binding | 0.074158935 | 1 | 26.06 |
| GOTERM_BP_FAT | GO:0008610~lipid biosynthetic process | 0.076847833 | 1 | 2.02 |
| GOTERM_BP_FAT | GO:0001666~response to hypoxia | 0.077281263 | 1 | 4.01 |
| GOTERM_BP_FAT | GO:0006958~complement activation, classical pathway | 0.078615711 | 1 | 6.41 |
| GOTERM_BP_FAT | GO:0051100~negative regulation of binding | 0.078615711 | 1 | 6.41 |
| GOTERM_BP_FAT | GO:0051605~protein maturation by peptide bond cleavage | 0.080134699 | 1 | 3.94 |
| GOTERM_BP_FAT | GO:0070482~response to oxygen levels | 0.080134699 | 1 | 3.94 |
| SP_PIR_KEYWORDS | blocked amino end | 0.081077729 | 1 | 6.31 |
| GOTERM_MF_FAT | GO:0016769~transferase activity, transferring nitrogenous groups | 0.083311678 | 1 | 6.20 |
| PIR_SUPERFAMILY | PIRSF018524:Noelin/Myocilin | 0.084865745 | 0.999999801 | 22.60 |
| INTERPRO | IPR013781:Glycoside hydrolase, subgroup, catalytic core | 0.085341469 | 1 | 6.12 |
| KEGG_PATHWAY | mmu00260:Glycine, serine and threonine metabolism | 0.085777079 | 0.999902643 | 6.04 |
| GOTERM_CC_FAT | GO:0043034~costamere | 0.086014009 | 0.999999996 | 22.29 |
| GOTERM_MF_FAT | GO:0004768~stearoyl-CoA 9-desaturase activity | 0.086992158 | 1 | 22.04 |
| GOTERM_BP_FAT | GO:0043623~cellular protein complex assembly | 0.087480381 | 1 | 2.97 |
| GOTERM_MF_FAT | GO:0030674~protein binding, bridging | 0.087838218 | 1 | 6.01 |
| GOTERM_MF_FAT | GO:0008235~metalloexopeptidase activity | 0.087838218 | 1 | 6.01 |
| SMART | SM00253:SOCS | 0.090233879 | 0.999991922 | 5.90 |
| SP_PIR_KEYWORDS | amino-acid transport | 0.090277893 | 1 | 5.92 |
| GOTERM_CC_FAT | GO:0044429~mitochondrial part | 0.091504912 | 0.999999999 | 1.66 |
| GOTERM_BP_FAT | GO:0006766~vitamin metabolic process | 0.091994421 | 1 | 3.72 |
| GOTERM_BP_FAT | GO:0040012~regulation of locomotion | 0.092083228 | 1 | 2.91 |
| GOTERM_BP_FAT | GO:0043627~response to estrogen stimulus | 0.092624196 | 1 | 5.83 |
| INTERPRO | IPR001496:SOCS protein, C-terminal | 0.094096422 | 1 | 5.78 |
| SMART | SM00104:ANATO | 0.094125272 | 0.999995253 | 20.22 |
| GOTERM_BP_FAT | GO:0008015~blood circulation | 0.094427322 | 1 | 2.89 |
| GOTERM_BP_FAT | GO:0003013~circulatory system process | 0.094427322 | 1 | 2.89 |
| GOTERM_BP_FAT | GO:0007049~cell cycle | 0.09446861 | 1 | 1.57 |
| GOTERM_BP_FAT | GO:0050817~coagulation | 0.095067271 | 1 | 3.66 |
| GOTERM_BP_FAT | GO:0007596~blood coagulation | 0.095067271 | 1 | 3.66 |
| GOTERM_BP_FAT | GO:0010033~response to organic substance | 0.095514256 | 1 | 1.65 |
| INTERPRO | IPR000020:Anaphylatoxin/fibulin | 0.096279623 | 1 | 19.82 |
| GOTERM_MF_FAT | GO:0003777~microtubule motor activity | 0.097437821 | 1 | 3.62 |
| GOTERM_BP_FAT | GO:0006775~fat-soluble vitamin metabolic process | 0.097444076 | 1 | 5.66 |
| GOTERM_BP_FAT | GO:0045787~positive regulation of cell cycle | 0.097444076 | 1 | 5.66 |
| GOTERM_BP_FAT | GO:0007599~hemostasis | 0.098181826 | 1 | 3.61 |
| GOTERM_BP_FAT | GO:0006865~amino acid transport | 0.098181826 | 1 | 3.61 |
| GOTERM_CC_FAT | GO:0031966~mitochondrial membrane | 0.098692682 | 1 | 1.82 |

**Suppl. Table S25** Expression of genes matching cancer-related annotation terms (from **Suppl. Table S24**) in clear-cell renal cell carcinoma (ccRCC) and in adjacent normal tissue in 66 human tumor/kidney samples from The Cancer Genome Atlas KCC database (http://cancergenome.nih.gov/).

| **Gene Symbol** | **Normalized Average Gene Expression in ccRCC (n=66)** | **Normalized Average Gene Expression in Normal Tissue (adjacent to ccRCC; n=66)** | **Fold Change ccRCC/Normal** | **Student's T-test (p value)** | **Direction of Expression in ccRCC** |
| --- | --- | --- | --- | --- | --- |
|  |  |  |  |  |  |
| *Ccnd1* | 176.01 | 32.76 | 5.37 | 3.09E-14 | up |
| *Myc* | 39.58 | 16.06 | 2.46 | 1.15E-09 | up |
| *Obfc2a* | 3.29 | 1.96 | 1.68 | 6.97E-05 | up |
| *Mdm2* | 18.12 | 10.95 | 1.66 | 7.27E-07 | up |
| *Cdkn1a* | 69.76 | 44.71 | 1.56 | 0.00064 | up |
| *Thbd* | 24.07 | 20.17 | 1.19 | 0.064 | up (nominally) |
| *Timp3* | 128.37 | 182.62 | 0.70 | 8.17E-05 | down |
| *Btc* | 0.76 | 2.78 | 0.27 | 1.21E-21 | down |
